# Tomato lncRNA23468 functions as a competing endogenous RNA to modulate *NBS-LRR* genes by decoying miR482b in the tomato*-Phytophthora infestans* interaction

**DOI:** 10.1038/s41438-018-0096-0

**Published:** 2019-02-01

**Authors:** Ning Jiang, Jun Cui, Yunsheng Shi, Guanglei Yang, Xiaoxu Zhou, Xinxin Hou, Jun Meng, Yushi Luan

**Affiliations:** 1 School of Life Science and Biotechnology, Dalian University of Technology, 116024 Dalian, China; 2 School of Computer Science and Technology, Dalian University of Technology, 116024 Dalian, China

**Keywords:** Biotic, Non-coding RNAs

## Abstract

Our previous studies indicated that tomato miR482b could negatively regulate the resistance of tomato to *Phytophthora infestans* and the expression of miR482b was decreased after inoculation with *P. infestans*. However, the mechanism by which the accumulation of miR482b is suppressed remains unclear. In this study, we wrote a program to identify 89 long noncoding RNA (lncRNA)-originated endogenous target mimics (eTMs) for 46 miRNAs from our RNA-Seq data. Three tomato lncRNAs, lncRNA23468, lncRNA01308 and lncRNA13262, contained conserved eTM sites for miR482b. When lncRNA23468 was overexpressed in tomato, miR482b expression was significantly decreased, and the expression of the target genes, *NBS-LRRs*, was significantly increased, resulting in enhanced resistance to *P. infestans*. Silencing lncRNA23468 in tomato led to the increased accumulation of miR482b and decreased accumulation of NBS-LRRs, as well as reduced resistance to *P. infestans*. In addition, the accumulation of both miR482b and NBS-LRRs was not significantly changed in tomato plants that overexpressed lncRNA23468 with a mutated eTM site. Based on the VIGS system, a target gene of miR482b, *Solyc02g036270.2*, was silenced. The disease symptoms of the VIGS-*Solyc02g036270.2* tomato plants were in accordance with those of tomato plants in which lncRNA23468 was silenced after inoculation with *P. infestans*. More severe disease symptoms were found in the modified plants than in the control plants. Our results demonstrate that lncRNAs functioning as eTMs may modulate the effects of miRNAs in tomato and provide insight into how the lncRNA23468-miR482b-NBS-LRR module regulates tomato resistance to *P. infestans*.

## A barrier against blight

The interplay between various non-protein-coding RNA molecules plays a critical role in managing tomato plants’ defenses against infection. Long non-coding RNA (lncRNA) molecules can modulate gene function by binding to other, complementary RNA molecules. Researchers led by Jun Meng and Yushi Luan at the Dalian University of Technology recently set out to identify lncRNAs that contribute to tomato resistance to the pathogen that causes late blight. Their team specifically sought lncRNAs targeting another RNA molecule known as miR482b, which they had previously found to weaken plant resistance to infection. They found that one such molecule, lncRNA23468, can counter the effects of miR482b and protect tomatoes against late blight by boosting the activity of genes involved in the immune response. These results thus offer important insights into the mechanisms underlying disease resistance in this important crop.

## Introduction

MicroRNAs (miRNAs) are noncoding RNAs of 20–24 nucleotides^1^ that transcriptionally and posttranscriptionally regulate gene expression in various biological processes of plants^2^. Many miRNAs act in plant responses to various biotic challenges. For example, miR482, belonging to the miR482/2118 family, functions in various plants^3,4^. After *Verticillium dahliae* infection, the accumulation of miR482e in potato significantly decreases^5^. Similarly, after infection with cucumber mosaic virus and *V. dahliae*, the expression of miR482 is suppressed in tomato and cotton, respectively^6,7^.

To show the biological function of miRNAs, identification of their target genes is necessary and important^8^. MiR482 can silence members of the NBS-LRR gene family^4^. An NBS-LRR protein is a disease resistance (R) protein that is involved in the effector-triggered immunity (ETI) of the plant innate immune system^9^. An NBS-LRR protein contains a nucleotide-binding site (NBS), a leucine-rich repeat (LRR) and a toll-interleukin receptor-like (TIR) domain or a coiled coil (CC) domain^10^. An NBS-LRR gene, *GbRVd*, was cloned and characterized in *Gossypium barbadense*, and its silencing enhances the susceptibility of *G. barbadense* to Verticillium wilt^11^. The resistance of *Nicotiana benthamiana* to *Phytophthora parasitica* is effectively enhanced by the overexpression of the grapevine TIR-NB-LRR gene *VaRGA1*^12^. Plants infected with pathogens show an increased level of *NBS-LRR* transcripts and reduced level of miR482^7,13^. Potato miR482 acts by suppressing *NBS-LRR* genes to regulate the potato resistance against *V. dahliae* infection^5^.

Recently, long noncoding RNA (lncRNA), another type of ncRNA, has been identified and analyzed in various biological processes^14^. LncRNAs are a set of RNA transcripts (>200 nt length) that have no protein-coding ability^15^. LncRNAs play important roles in flowering time regulation^16^, fruit development^17,18^, photomorphogenesis^19^, gene silencing^20,21^ and biotic and abiotic stress responses^22,23^. In plant-pathogen interactions, using an RNA sequencing approach, 125 putative stress-responsive lncRNAs that are induced by powdery mildew infection have been identified in wheat ^24^. A number of lncRNAs have been identified in Paulownia witches’ broom-infected *Paulownia tomentosa* by high-throughput sequencing^25^. In addition, *Arabidopsis thaliana* lncRNAs have been found to act in *Arabidopsis* resistance to *Fusarium oxysporum*^22^. In tomato, a comprehensive set of lncRNAs was identified^26–28^. These lncRNAs were primarily involved in the regulation of tomato fruit ripening^18,28–30^, the ethylene signal transduction pathway^31^, chilling injury^32^, and the tomato-potato spindle tuber viroid (PSTVd)/tomato yellow leaf curl virus (TYLCV) interaction^33–35^.

Tomato is the second most important vegetable crop in the world, constituting a major agricultural industry^36^. Late blight (LB), caused by *Phytophthora infestans*, is one of the most serious diseases of tomato. In the early 2000s, LB occurred and hindered tomato production, causing serious economic losses in the USA and China^37^. In our previous work, we focused on the effects of lncRNAs and miRNAs during tomato resistance to *P. infestans*. A number of miRNAs were identified by next-generation sequencing^38^, including miR482b. MiR482b was downregulated after infection with *P. infestans* and negatively regulated tomato resistance^8^. Tomato lncRNA16397 was found to induce *glutaredoxin* expression to enhance resistance to *P. infestans*^39^. However, the mechanisms by which lncRNAs, as competing endogenous RNAs **(**ceRNAs), suppress the accumulation of miRNAs in the tomato-*P. infestans* interaction are unknown. To determine whether lncRNAs can suppress miR482b accumulation in tomato resistance to pathogen infection, RNA-Seq data were used to identify and characterize a number of lncRNAs, and bioinformatics analysis was used to predict the endogenous target mimics (eTMs) of these lncRNAs. We found that lncRNA23468 modulated the accumulation of *NBS-LRRs* by suppressing miR482b expression in tomato plants infected with *P. infestans*. These results will improve our understanding of the regulatory mechanism of miR482b in the response of tomato to *P. infestans* infection and help future molecular-based breeding approaches of pathogen resistance.

## Materials and methods

### Bioinformatics pipeline for identifying lncRNAs

To identify the lncRNAs, we used two RNA-Seq datasets obtained from our previous studies. These two datasets were constructed by LC Biotech, Hangzhou, China, using the leaves of miR482b-overexpressing and Zaofen No. 2 tomatoes. The clean reads were *de novo* assembled using Cufflinks. TopHat was used to align assembled transcripts to the tomato genome iTAGv2.3 (http://phytozome.jgi.doe.gov/pz/portal.html#!info?alias = Org_Slycopersicum). All transcripts were required to be more than 200 bp in length. The lncRNAs were identified according to the method of Cui et al.^39^. According to their genomic locations, the lncRNAs were classified into four categories. The fragments per kilobaseof exon per million fragments mapped (FPKM) value was applied to represent the normalized expression value of the lncRNAs.

### Prediction of ceRNAs

All tomato miRNAs were collected from miRBase (http://www.mirbase.org/), and the lncRNAs identified above were used as the ceRNA prediction libraries. CeRNAs for the selected tomato miRNAs were predicted using RNAhybrid software with the following rules: (i) *P*-value < 0.05 and minimum free energy (mfe) <−25 kcal/mol; (ii) bulges were only permitted at the ninth to 12th positions of the 5’ end of a miRNA sequence; (iii) the bulge in ceRNAs should be composed of 2–4 nucleotides; (iv) G/U pairs were allowed with the ceRNA and miRNA pairing region, and perfect nucleotide pairing was required at the second to eighth positions of the 5’ end of the miRNA sequence; and (v) except for the central bulge, the total mismatches within the ceRNA and miRNA pairing regions should be no more than four, with no more than two consecutive mismatches. Interaction networks among the ceRNAs and miRNAs were constructed using the software Cytoscape. WebLogo software was used to analyze the conserved residues (http://weblogo.berkeley.edu/logo.cgi).

### Tomato material and *P. infestans* inoculation

Tomato Zaofen No. 2, bred by the Institute of Vegetables and Flowers, Chinese Academy of Agricultural Sciences, Beijing, China, is an accession susceptible to *P. infestans*. The tomato was grown in a greenhouse under 16 h light within a temperature range of 22–28 °C. *P. infestans* strain P12103 was cultured in oat medium in the dark at 20 °C. The tomato plants (4–5-leaf stage) were inoculated with *P. infestans* spores according to the method of Jiang et al.^8^. The whole fifth leaves of each sample were collected at the indicated times (0, 1, 2, 3 and 4 dpi). All samples were quickly frozen in liquid nitrogen and stored at −80 °C until RNA isolation.

### Cloning of lncRNA23468, mutation of lncRNA23468 and construction of the overexpression plasmid

According to the tomato genome and lncRNA prediction results, a pair of primers (l23468F and l23468R) were designed and used to clone lncRNA23468 from tomato plant (Table S1).

We introduced six point mutations to lncRNA23468 within sequences pairing with miR482b. LncRNA23468 mutation was generated by PCR, which involved amplification and mutagenesis using lncRNA23468 as the backbone. Two more primers were used for this: ml23468-1R and ml23468-2F (Table S1). Three rounds of PCR were performed to amplify the mutated lncRNA23468 (mlncRNA23468). First, the primers l23468F and ml23468-1R were used to amplify a fragment containing mutation points, and then ml23468-2F and l23468R were used to amplify another fragment. Finally, the PCR products of the first and second rounds were used as the template along with l23468F and l23468R.

The PCR fragments of lncRNA23468 or mlncRNA23468 were subcloned into binary vector pBI121, replacing the *GUS* gene. In plasmids, lncRNA23468 and mlncRNA23468 were controlled by the Cauliflower mosaic virus (CaMV) 35S promoter.

### Virus-induced gene silencing (VIGS) constructs

TVR-based vectors (pTRV1 and pTRV2), which were provided by Prof. Liu from Tsinghua University of China, were used for VIGS. The VIGS sequence was designed according to the SGN VIGS Tool (http://vigs.solgenomics.net/) and cloned into the pTRV2 vector by the ligation-independent cloning method^40,41^.

### Agrobacteria infiltration

All the plasmids were transformed into *A. tumefaciens* strain GV3101 by the freeze-thaw method^37^. *Agrobacteria* infiltration was performed according to Jiang’s method^8^.

*A. tumefaciens* containing pBI-121-lncRNA23468 or pBI-121-mlncRNA23468 plasmids was introduced into the leaves of Zaofen No. 2 tomato by infiltration. *A. tumefaciens* with an empty vector was used as a control. The leaves were harvested for the next experiments at 3 dpi.

In the tobacco (*N. tabacum*) system, we introduced *Agrobacterium* harboring pBI121-miR482b into tobacco leaf cells. After 3 days, the *Agrobacterium* harboring pBI121-lncRNA23468 and mlncRNA23468 were introduced into the tobacco leaves that expressed miR482b. The accumulation of miR482b was examined.

Agrobacterium cultures containing pTRV2 derivatives and pTRV1 were mixed at a 1:1 ratio and then infiltrated into a 2-3-leaf-stage tomato. The pTRV2 empty vector was used as the negative control. The plants were maintained for 3 weeks in a greenhouse under 16 h light within 20 °C, and leaflets were harvested from several plants for the isolation of RNA and qRT-PCR analysis to assess the degree of silencing.

### *P. infestans* resistance analysis

For the tomato plants that overexpressed lncRNA23468 or mlncRNA23468, the infiltrated leaf regions were inoculated with 20 μl of *P. infestans* (10^6^ zoospores/ml) and then were placed at 20 ± 1 °C in a 100%-relative-humidity environment without light. The lesions were observed at the fifth day, and the sizes of the lesions were also calculated.

3 weeks after VIGS, detached leaves from the VIGS tomato plants were inoculated with 20 μl of the *P. infestans* zoospore suspension (1 × 10^6^ zoospores/ml) according to Jiang’s method^8^. The whole plants were sprayed to run-off with the same zoospore suspension by the method described above. At 5 dpi, the diameters of the lesions and the abundance of *P. infestans* were calculated according to Jiang’s method^8^.

The disease index (DI) was calculated according to disease grade (DG). The DGs were sorted from 0 to 6 on the basis of the lesion area (Table S2). The DI was calculated according to the following formula:

$${\mathrm{DI}}({\mathrm{\% }}) = \frac{{{\sum} {\left( {DG_i \times n_i} \right) \times 100} }}{{n \times DG_{imax}}}$$

Where DGi is the value of DG, *n*_i_ is the number of plants in each DG, and *n* is the total number of plants. Each experiment was carried out at least three times.

### RNA isolation, reverse transcription, and qRT-PCR analysis

Total RNA was extracted using RNAiso Plus (TaKaRa, Dalian, China). Reverse transcription and the qRT-PCR reactions of miRNAs were performed with TransScript Green miRNA Two-Step qRT-PCR SuperMix (Transgen Biotech, Beijing, China) according to the manufacturer’s instructions. We used PrimeScript^TM^ RT Master Mix (TaKaRa, Dalian, China) to synthesize the cDNAs of the mRNAs and lncRNAs. The qRT-PCR reactions were performed by using a SYBR Premix Ex Taq^TM^ II kit (TaKaRa, Dalian, China). The qRT-PCR reactions of the selected genes, miRNAs and lncRNAs were performed on an ABI7500. The tomato *actin* gene was used as an internal reference gene. All primer sequences are shown in Table S1. The gene sample *Ct* values were standardized, and the 2^–ΔΔCt^ method was used to analyze the relative changes in expression. Of the nine leaves sampled in each experiment, three leaves were pooled into one biological replicate, resulting in three biological replicates.

### Statistical analysis

All statistical analyses of the data were performed with SPSS19.0, and all data were expressed as the means ± SEs from three independent experiments. We used the Tukey method to estimate significance.

## Results

### Identification of lncRNAs

**Fig. 1:**
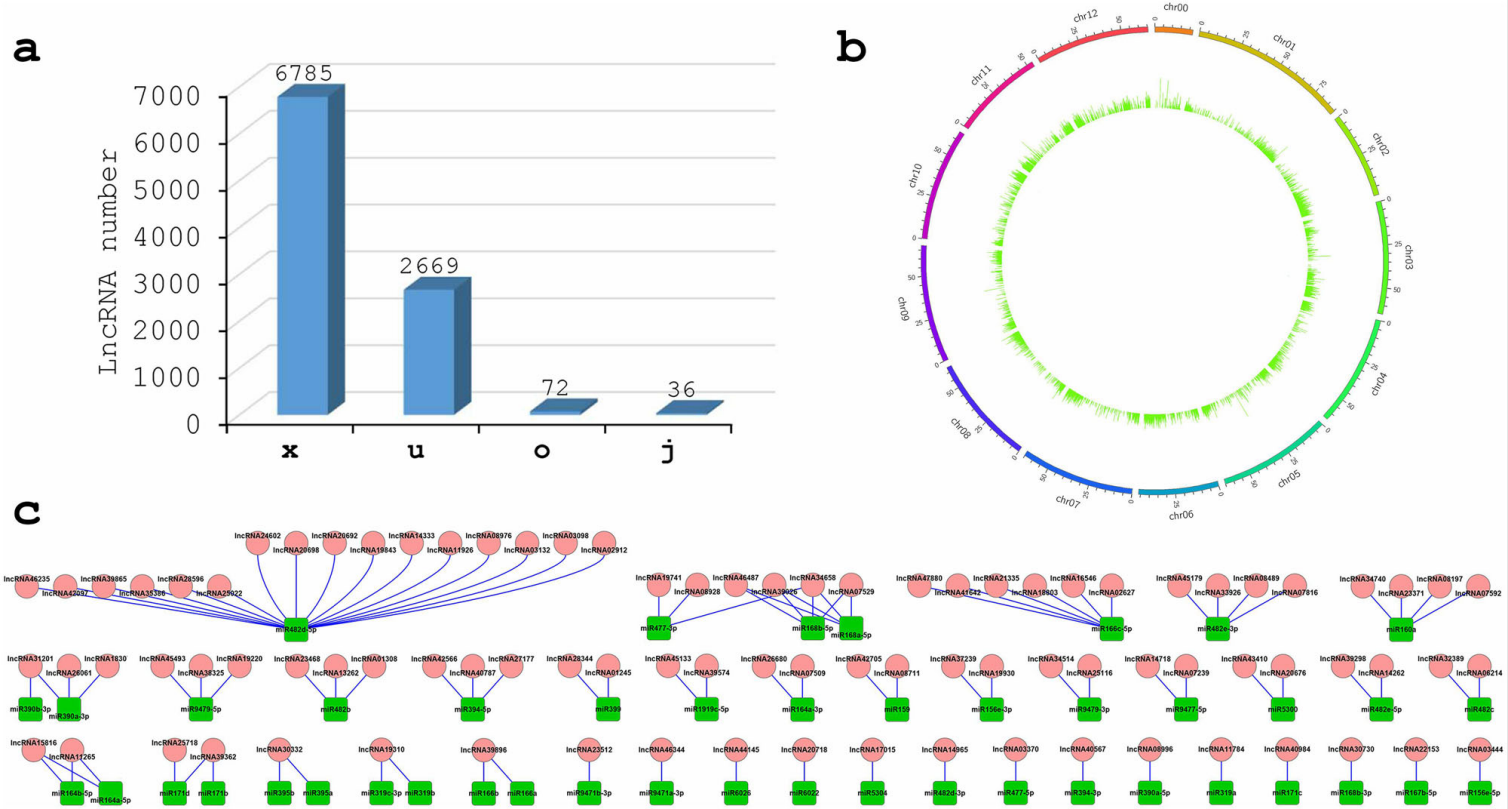
Identification and characterization of lncRNAs. **a** Composition of different types of lncRNAs. **b** The expression levels of the lncRNAs (log_10_ FPKM) along 12 tomato chromosomes. **c** Cytoscape results of lncRNAs and miRNAs. The 89 lncRNAs that may act as ceRNAs could be bound by 46 miRNAs. The red round and green square nodes represent lncRNAs and miRNAs, respectively

Two of our group’s RNA-Seq datasets, miR482b-overexpressing (OE482) and Zaofen No. 2 tomatoes (Slz), were used to identify the expressed lncRNAs. After assembly and mapping to the tomato genome, the noncoding transcripts were considered “putative” lncRNAs and filtered according to length, coding potentials and coverage of reads. From these analyses, approximately 9742 unique lncRNAs were obtained in two samples, and 9562 of these lncRNAs were expressed in the OE482 sample (Table S3). Among these lncRNAs, 6,785 were natural antisense transcripts (x); 2,669 were long intergenic noncoding RNAs (u); 72 were generic exonic overlaps with a reference transcript (o); and 36 were potentially novel isoforms (j) in the OE482 sample (Fig. 1a). These lncRNAs were evenly distributed across the 12 chromosomes in tomato (Fig. 1b).

### Identification of ceRNAs

LncRNAs may act as ceRNAs via the eTMs of miRNAs. Therefore, a program was written to identify the lncRNAs acting as eTMs of miRNAs in tomato. In total, 89 lncRNAs were found to be bound by 46 miRNAs (Table S4). Interaction networks showed that 38 miRNA-lncRNA duplexes were formed (Fig. 1c). In these duplexes, miR482d-5p was putatively sequestered by 16 lncRNAs, and lncRNA34658 decoyed three miRNAs, including miR168a-5p, miR168b-5p, and miR477-3p. In addition, more than one-third of the regulated relationships had only two nodes, as shown in the networks in Fig. 1c.

### The eTMs of miR482b and expression analysis of miR482b ceRNAs after infection with *P. infestans*

**Fig. 2:**
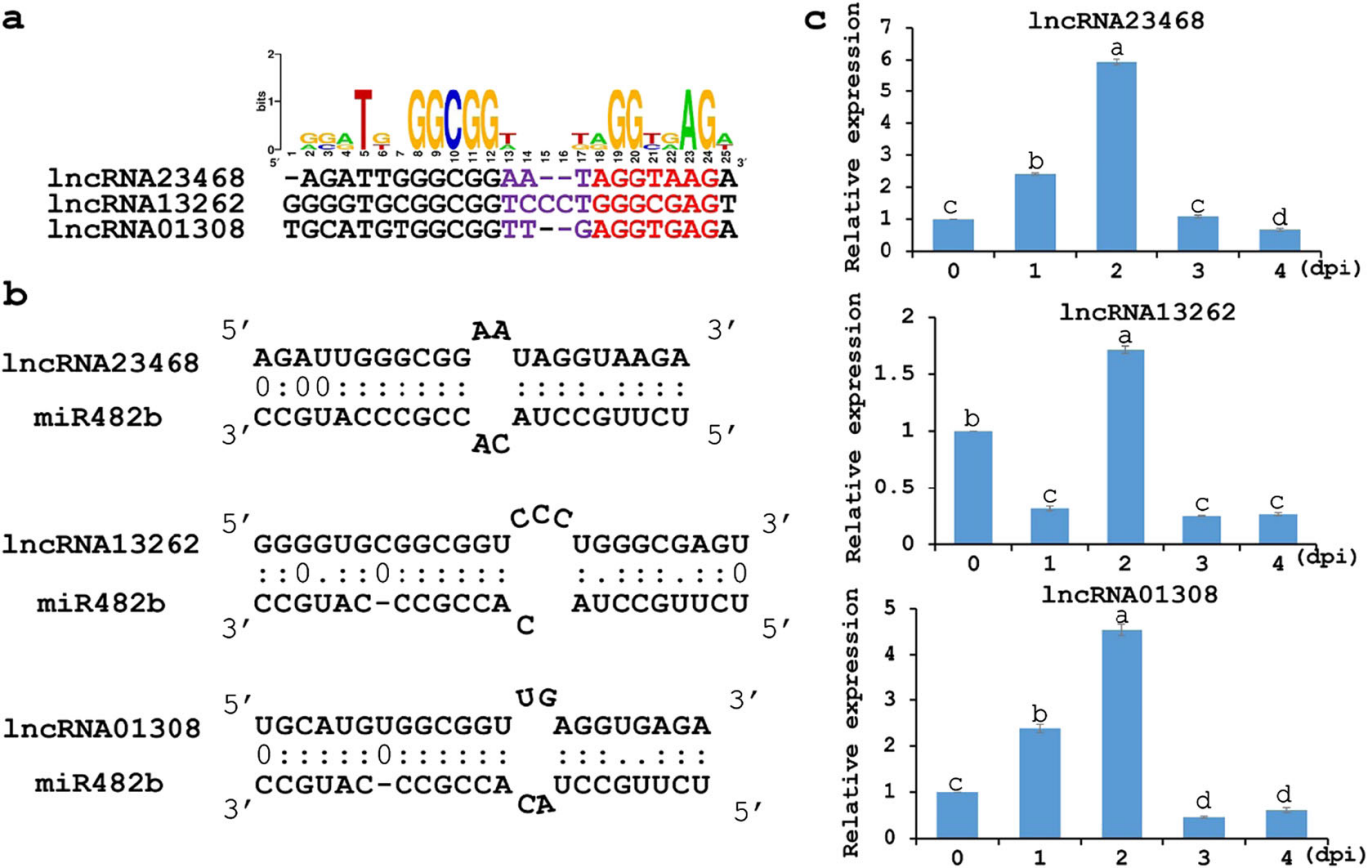
Three lncRNAs decoy miR482b in tomato. **a** Conservation analysis of the eTM sites of three lncRNAs that decoy miR482b. The conservation status of the sequence was analyzed by WebLogo. The red letters represent the conserved sequence at the second to eighth positions of the 5’ end of the miRNA sequence. The purple letters represent bulges permitted at the ninth to 12th positions of the 5’ end of a miRNA sequence. **b** Predicted base-pairing interactions between miR482b and the eTM sites of three lncRNAs. **c** Expression of the three lncRNAs in tomato inoculated with *P. infestans*. All data are the means ± SE of three independent experiments. Different letters among the groups indicate a significant difference at the *P* = 0.05 level

The predicted binding sites of miR482b among these lncRNAs (lncRNA23468, lncRNA13262 and lncRNA01308) were well conserved (Fig. 2a). In our prediction, a two or three-nucleotide bulge on the eTMs located between the ninth and 12th positions at the 5’ end of miR482b was required for the eTMs to decoy miR482b (Fig. 2b). The minimum free energies (mfes) were −33.8 kcal/mol, −35.8 kcal/mol and −33.2 kcal/mol in lncRNA13262-miR482b, lncRNA01308-miR482b and lncRNA23468-miR482b, respectively. The values of the mfes in lncRNA13262-miR482b and lncRNA01308-miR482b were close to that in lncRNA23468-miR482b. This suggests that the abilities of the three lncRNAs to suppress miR482b were not significantly different.

To explore whether the ceRNAs of the miR482b, lncRNA23468, lncRNA13262 and lncRNA01308 had an effect on tomato response to *P*. *infestans*, the relative levels of accumulation were examined in tomato leaves infected by *P*. *infestans*. These three lncRNAs responded to *P. infestans* infection. The changes in the expression of both lncRNA23468 and lncRNA01308 followed the same pattern. Both first increased and then decreased, reaching a peak at 2 days after *P*. *infestans* infection. LncRNA13262 was downregulated from 0 dpi to 1 dpi and then reached a peak at 2 dpi (Fig. 2c).

### Construction of the lncRNA23468 mutant

**Fig. 3:**
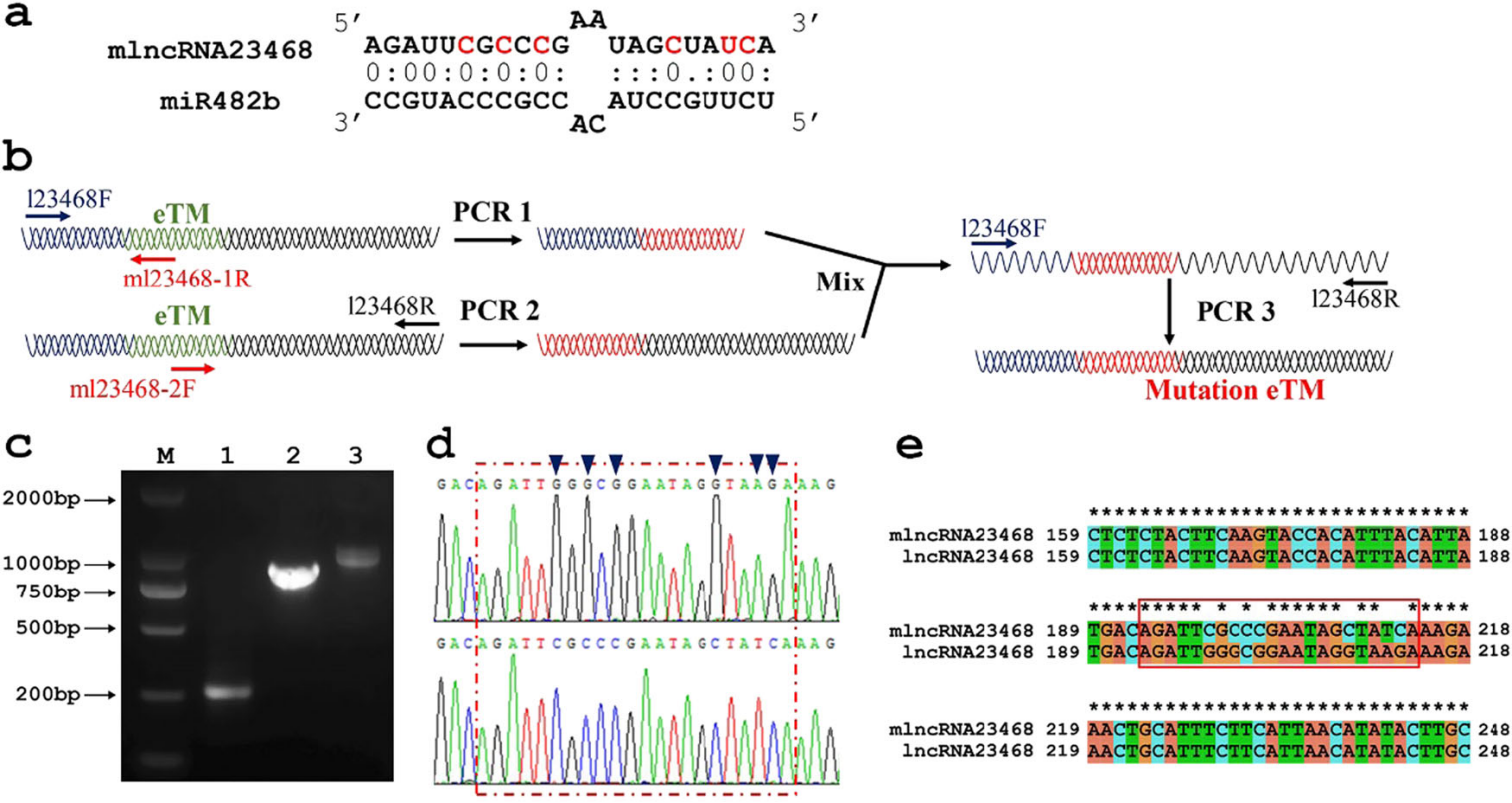
Construction of the mutant eTM of lncRNA23468. **a** Predicted base-pairing interactions between miR482b and the eTM of lncRNA23468 with the designed mutations. **b** Strategy for introducing mutations by PCR. **c** PCR products from the different stages of the mutant eTM of lncRNA23468. M, DL2000 DNA Marker; 1, 2, and 3, products from the first, second and third rounds of PCR, respectively. **d** The sequencing results of the eTM regions of lncRNA23468 (top) and its mutated sequence (bottom). The red box represents the eTM region. Blue triangles represent the six-point mutation. **e** Sequence alignment of lncRNA23468 and mlncRNA23468 performed with ClustalX. The red box represents the eTM region

To examine whether lncRNA23468 indeed functioned via pairing with miR482b, a six-point mutation was introduced into lncRNA23468 by using sequences that paired with miR482b (mlncRNA23468; Fig. 3a). The design of the mlncRNA23468 is shown in Fig. 3b. The eTM of miR482b (5′-AGAUUGGGCGGAAUAGGUAAGA-3′) in lncRNA23468 was replaced with the mutation sequence (5′-AGAUUCGCCCGAAUAGCUAUCA-3′) by oligonucleotide-directed mutagenesis. After three rounds of PCR, the sequence of mlncRNA23468 was amplified (Fig. 3c). As shown in Fig. 3d, the sequencing results showed that six point mutations were introduced into the eTM from lncRNA23468. In addition, except for the eTM region of miR482b, the lncRNA23468 and mlncRNA23468 sequences were conserved after sequence alignment analysis (Fig. 3e). Overall, mlncRNA23468 was successfully constructed by using lncRNA23468 as the backbone.

### LncRNA23468 as a miRNA decoy suppresses the expression of miR482b

**Fig. 4:**
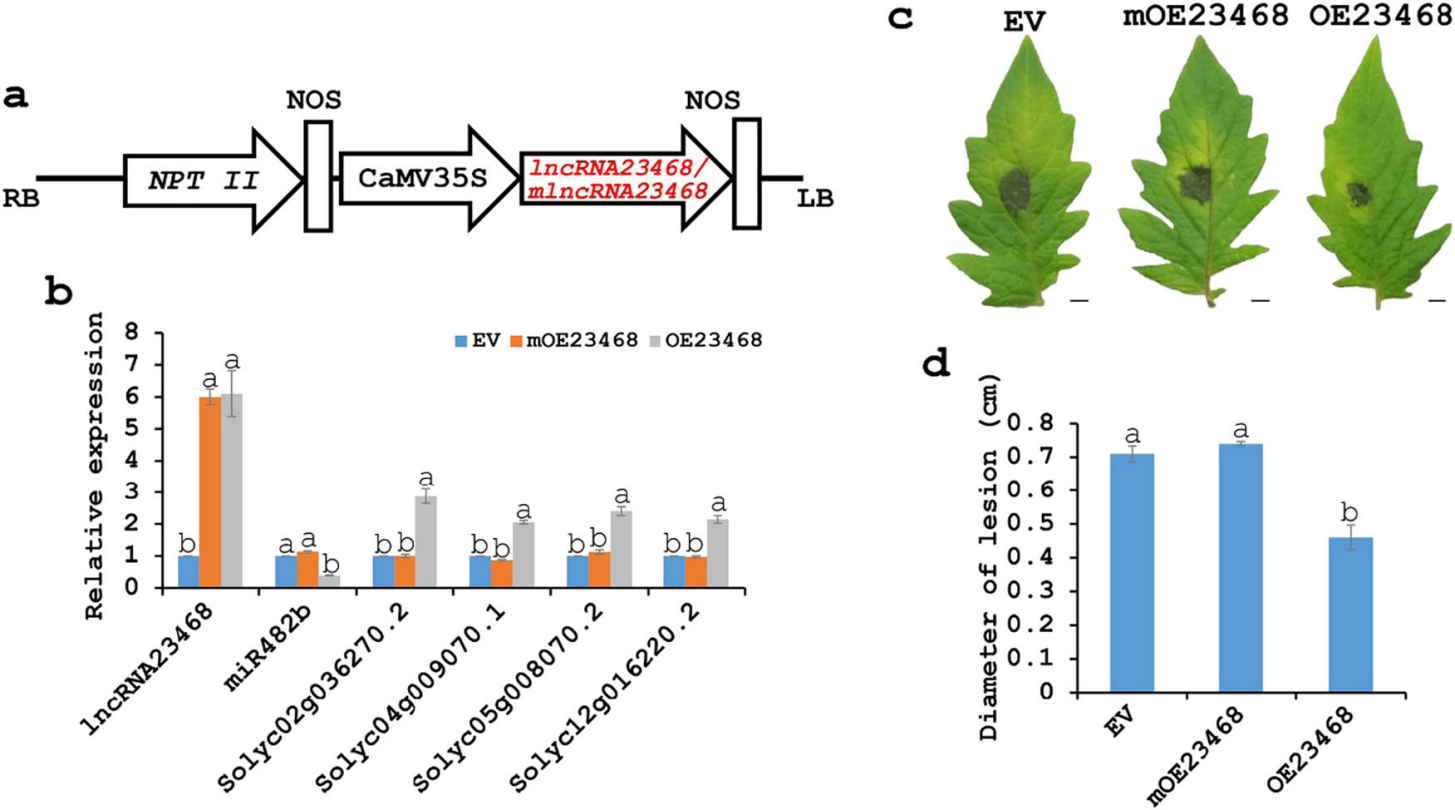
lncRNA23468 functions to modulate *NBS-LRR* genes by decoying miR482b. **a** Schematic diagram of the gene cassette containing lncRNA23468 and mlncRNA23468. **b** qRT-PCR analysis of lncRNA23468, miR482b and the target genes in the EV, mOE23468 and OE23468 tomato plants. **c** Disease signs on the detached leaves from the EV, mOE23468 and OE23468 tomato plants at 5 dpi. Scale bars = 0.5 cm. **d** The diameter of the lesion of the detached leaves from EV, mOE23468 and OE23468 tomato plants at 5 dpi. All data are the means ± SE of three independent experiments. Different letters among the groups indicate a significant difference at the *P* = 0.05 level

We investigated the possible function of lncRNA23468 using infiltration to upregulate lncRNA23468 expression in Zaofen No. 2 tomato. The plasmids for the overexpression of lncRNA23468 and mlncRNA23468 were carried out on the basis of the pBI121 vector (Fig. 4a). The introduction of *A. tumefaciens* harboring pBI121-lncRNA23468 and pBI121-mlncRNA23468 into tomato leaf cells resulted in significant upregulation of lncRNA23468 and mlncRNA23468 expression at 3 days: the expression levels of lncRNA23468 and mlncRNA23468 were approximately 6.1-fold and 6.0-fold greater than the tomato leaves that overexpressed empty vector (EV), respectively (Fig. 4b). The expression of miR482b was dramatically suppressed to approximately 40% in the tomato plants that overexpressed lncRNA23468 (OE23468), and the target genes of miR482b, *NBS-LRRs*, were significantly increased (Fig. 4b). The levels of the transcripts of *Solyc02g036270.2*, *Solyc04g009070.1*, *Solyc05g008070.2* and *Solyc12g016220.2* in the leaves of the OE23468 tomato plants were approximately 2.9-fold, 2.1-fold, 2.4-fold, and 2.1-fold, respectively, to the values in the leaves of the EV tomato plants. The overexpression of mlncRNA23468 in tomato (mOE23468) did not cause expression changes in either miR482b or its target genes (Fig. 4b).

In our previous work, miR172 and miR396 were identified to play important roles in tomato resistance to *P. infestans*^42,43^. We examined the accumulation of miR172, miR396 and their target genes in the tomato leaves that overexpressed lncRNA23468 by using qRT-PCR. The overexpression of lncRNA23468 did not cause expression changes in miR172, miR396 and their target genes compared to the control samples (Fig. S1).

Disease resistance tests were performed on the OE23468, mOE23468 and EV plants using the oomycete *P. infestans*. Five days after infection, the physical appearance of these tomato plants was assessed. Compared with the severe disease symptoms that appeared on the leaves of EV plants, the disease symptoms on the leaves of mOE23468 plants were similar, while fewer disease symptoms were exhibited on the leaves of OE23468 plants (Fig. 4c). The diameter of the lesion was significantly smaller in the leaves of *P. infestans*-infected OE23468 plants than in those of EV or mOE23468 plants (Fig. 4d).

**Fig. 5:**
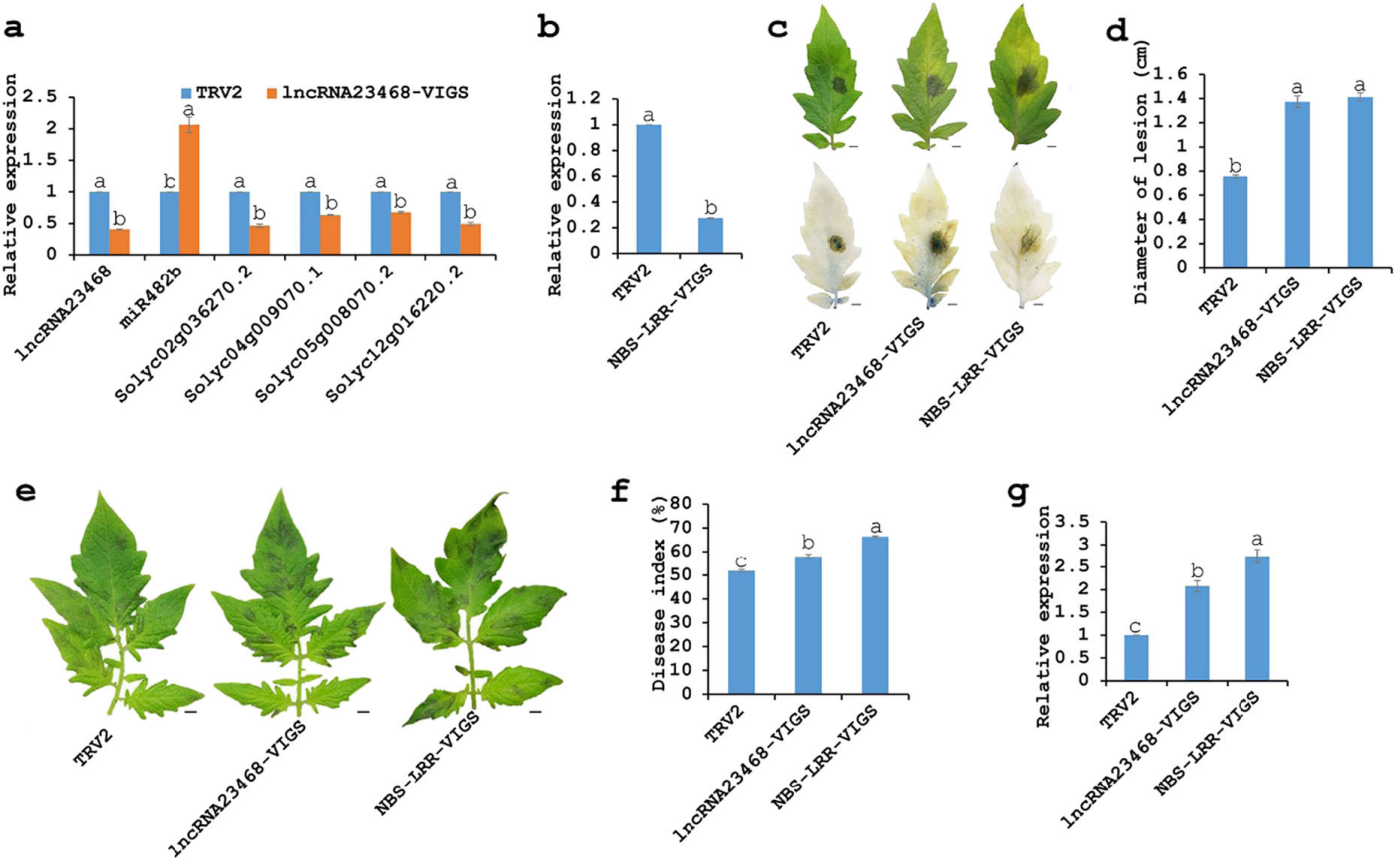
Silencing of lncRNA23468 and *NBS-LRR* gene decreases tomato resistance to *P. infestans*. **a** Relative expression levels of lncRNA23468, miR482b and the target genes of miR482b in the TRV2 and lncRNA23468-VIGS tomato plants. **b** The silencing efficiency of *NBS-LRR* in NBS-LRR-VIGS tomato plants. **c** Phenotypes of the detached leaves from TRV2, lncRNA23468-VIGS and NBS-LRR-VIGS tomato plants at 5 dpi. Scale bars = 0.5 cm. Top: disease symptoms; Bottom: trypan blue staining for detection of dead cells. **d** The diameter of the lesion of the detached leaves. **e** Leaf phenotypes at 5 dpi after whole-plant inoculation with *P. infestans*. Scale bars = 0.5 cm. **f** Disease index of VIGS-tomato plants at 5 dpi. **g** Transcript accumulation of the *P. infestans actin* gene in these inoculated plants at 5 dpi. All data are the means ± SE of three independent experiments. Different letters among the groups indicate a significant difference at the *P* = 0.05 level

As overexpression is not enough to fully understand lncRNA functions, lncRNA silencing should be performed to explore these biological functions. LncRNA23468 was silenced in Zaofen No. 2 tomato by VIGS. The empty TRV2 vector and *PHYTOENE DESATURASE* (PDS)*-*VIGS were used as the negative control and the positive control, respectively. At 21 days of VIGS treatment, the *PDS-*VIGS tomato showed visible bleaching, indicating that the *lncRNA23468* gene was also silenced (Fig. S2). The expression of lncRNA23468 was suppressed to approximately 40%, and the accumulation of miR482b was upregulated approximately 2-fold, while the four target genes showed decreased abundance (Fig. 5a).

The disease phenotypes for the control (TRV2) and lncRNA23468-VIGS tomato plants were assessed after infection with *P. infestans*. Compared to the leaves of TRV2 tomato plants, the detached leaves of the lncRNA23468-VIGS tomato plants showed more serious symptoms of the disease. The decreased resistance to *P. infestans* in the leaves of lncRNA23468-VIGS tomato plants was also revealed by trypan blue staining, as shown by the increased number of necrotic cells relative to those in the leaves of TRV2 tomato plants (Fig. 5c). In addition, Fig. 5d shows that the leaves of the lncRNA23468-VIGS tomato plants had larger lesions than did the leaves of the TRV2 tomato plants.

The leaves of the lncRNA23468-VIGS tomato plants also exhibited more serious symptoms of the disease after whole-plant inoculation with *P. infestans* (Fig. 5e), with a higher disease index than that observed for the TRV2 tomato plants (Fig. 5f). In addition, there were significant increases in the abundance of *P. infestans* in the lncRNA23468-VIGS tomato plants compared to the TRV2 tomato plants (Fig. 5g). These results suggest that lncRNA23468 suppressed the accumulation of miR482b, resulting in an increased expression of *NBS-LRRs* and enhanced tomato resistance to *P. infestans*.

The regulation between lncRNA23468 and miR482b was also validated using the *Nicotiana* system. We introduced *Agrobacterium* harboring pBI121-miR482b into tobacco leaf cells, which led to a significant increase in miR482b expression at 3 days compared to tobacco leaves overexpressing the empty vector (Fig. S3a). Then, the *Agrobacterium* harboring pBI121-lncRNA23468 and mlncRNA23468 were introduced into the tobacco leaves that overexpressed miR482b. The accumulation of miR482b was significantly decreased after the overexpression of lncRNA23468. The overexpression of mlncRNA23468 did not cause expression changes in miR482b (Fig. S3b). These results suggested that lncRNA23468 as a ceRNA may decoy miR482b.

### Silencing of *NBS-LRR* enhances tomato susceptibility to *P. infestans*

We also employed the VIGS system to test the role of the target gene of miR482b (Solyc02g036270.2) in tomato resistance to *P. infestans*. At 21 days after hand-infiltration, a further examination of *Solyc02g036270.2* indicated that the abundance of transcripts was significantly suppressed in the tomato plants that silenced *Solyc02g036270.2* (*NBS-LRR-*VIGS) (Fig. 5b).

More serious disease symptoms were shown in the detached leaves of the *NBS-LRR*-VIGS plants than in the TRV2 plants after infection with *P. infestans* (Fig. 5c). The darker blue marks from trypan blue staining in the detached leaves indicated that a greater number of dead cells occurred in the *NBS-LRR-VIGS* tomato plants than in the TRV2 tomato plants (Fig. 5c). Furthermore, these *NBS-LRR*-VIGS tomato leaves also had larger diameter lesions (approximately 1.91-fold increase compared to TRV2 tomato plants infected with *P. infestans*) (Fig. 5d). In the whole-plant inoculation assay, the susceptibility of the silenced plants manifested as more serious disease symptoms (Fig. 5e), higher DI (Fig. 5f) and an increased abundance of *P*. *infestans* (Fig. 5g).

## Discussion

### LncRNA as ceRNA regulates the accumulation of miRNA

MicroRNAs (miRNAs), a class of approximately 22 nt endogenous small noncoding RNAs, can direct the posttranscriptional repression of target genes by base-pairing to mRNAs. The mature miRNA is separated from the miRNA/miRNA* duplex, which is processed from a pre-miRNA^1^.

One focus of miRNA research is the regulation of miRNA accumulation. Various transcription factors can promote the expression of pre-miRNAs at the transcriptional level. For example, in *A. thaliana*, transcription factor APETALA2 (AP2) positively or negatively regulates the expression of miR156 or miR172 by binding to these miRNA genes^44^. Another *Arabidopsis* transcription factor, *At*MYB2, has been identified for its roles in the activation of miR399f expression in the context of phosphate homeostasis through sequence-specific interactions with a MYB-binding site in the promoter of the miR399f precursor^45^. In addition, tomato ripening inhibitor (RIN) binds to a RIN-binding site in the promoter of *miR172a* to regulate its accumulation^46^.

Another regulatory mechanism has been proposed to underlie the accumulation of miRNA. During plant-pathogen interactions, pathogen effectors can suppress host defense^47^. The effectors, RNA-silencing suppressors from the pathogens, affect the resistance of plants by suppressing the accumulation of host miRNAs. *P. sojae* encoded two RNA-silencing suppressors (*Ps*PSR1 and *Ps*PSR2) that enhance plant susceptibility by impairing the small RNA-mediated defense of the host^48^. The *Ps*PSR1 virulence target acts in the assembly of sRNA-processing complexes in *Arabidopsis* and soybean^49^. PSR2 is involved in modifying plant gene regulation early during *Phytophthora* infection, and the overexpression of *PsPSR2* in *Arabidopsis* enhances hypersusceptibility to *P. capsici*^50^.

Some specific endogenous lncRNAs can act as ceRNAs to interfere with miRNA pathways^51^. Acting as a ceRNA is an effective posttranscriptional regulatory mechanism by which lncRNAs interfere with target transcripts. The disruption of the equilibrium between ceRNAs and miRNAs could be important to ceRNA activity in diseases such as cancer^52^. For example, the lncRNA HOTAIR decoys miR-152-3p to promote malignant melanoma progression^53^. In addition, the lncRNA CRNDE prevents miR-136-5p-mediated downregulation of Bcl-2 and Wnt2 to promote glioma malignancy^54^. However, studies on ceRNA in plants are limited. In maize, a number of lncRNAs have been identified as miRNA decoys by using computational methods^55^. Tobacco eTMX27 inhibits the expression of miRX27, resulting in enhanced nicotine biosynthesis^56^. *Arabidopsis* IPS1 contains the eTM of ath-miR399 and inhibits the expression of ath-miR399^20^, and PDIL1 reduces the accumulation of miR399, thus regulating Pi-related pathways in *Medicago truncatula*^57^. Wang et al.^21^ reported that the eTMs of miR160 from lncRNAs can serve as decoys for miR160 and function as the regulator in rice development.

In this study, we wrote a program that systematically identified 89 lncRNAs as 46 miRNA decoys in tomato (Fig. 1c and Table S4). Three of these lncRNAs, lncRNA23468, lncRNA01308 and lncRNA13262, contained an eTM site of miR482b (Fig. 2b). The expression of these three lncRNAs was initially increased but then decreased with *P. infestans* infection (Fig. 2c). This result was in contrast to the expression trend of miR482b upon infection by *P. infestans*, which was detected in our previous study^8^. The expression of miR482b was decreased in the tomato plants that overexpressed lncRNA23468 (Fig. 4a), while silencing lncRNA23468 led to increased miR482b accumulation (Fig. 5a). In addition, the accumulation of miR482b was not significantly changed in the tomato plants that overexpressed mlncRNA23468 with a mutated eTM site (Fig. 4a). These results suggest that the lncRNAs functioned as an eTM that may modulate the effects of miRNAs in tomato.

### The lncRNA23468-miR482b-*NBS-LRR* network in tomato resistance to *P. infestans*

The miR482b family is involved in various plant interactions with pathogens^6,58^. The overexpression of miR482e in potato decreased plant resistance to *V. dahliae* infection^5^. Similar results were reported in our previous study, showing that miR482b negatively regulates tomato resistance to *P. infestans*^8^. To understand the biological functions of miRNAs, the identification of their target genes is an important step. In our previous study, four members of the NBS-LRR family were identified as the target genes of miR482b using the degradome data of tomato infected with *P. infestans*^8^. NBS-LRRs are active in the resistance of other plants to pathogens, such as tobacco resistance to *P. parasitica*^12^, wheat resistance to powdery mildew^59^, and rice resistance to blast^60^. In this study, NBS-LRR accumulation was suppressed by silencing lncRNA23468 and VIGS technology in tomato, resulting in the enhancement of tomato susceptibility to *P. infestans* (Figs. 4 and 5). Similarly, cotton susceptibility is also increased by silencing a *CC-NBS-LRR* gene^11^.

LncRNAs are involved in many biological processes, including biotic stresses. For example, Joshi et al.^61^ found that 13 lncRNAs were predicted to be the precursors of 96 miRNAs that affect the *Brassica napus* and *Sclerotinia sclerotiorum* interaction. The silencing of GhlncNAT-ANX2 and GhlncNAT-RLP7 in cotton seedlings can induce *LOX1* and *LOX2* expression to increase the resistance to *V. dahliae* and *Botrytis cinerea* infection^62^. In addition, many lncRNAs act in the response of *Arabidopsis* to *F. oxysporum* and *Pseudomonas syringe* pv. *tomato* DC3000, wheat response to powdery mildew pathogen, tomato response to TYLCV and *P. tomentosa* response to Paulownia witches’ broom^22,24,25,33,35^. The overexpression of lncRNA16397 in tomato induced glutaredoxin expression to increase the resistance to *P. infestans*^39^. Another tomato lncRNA, lncRNA23468, was also responsive to *P. infestans* infection (Fig. 2c). In addition, the overexpression of lncRNA23468 in tomato positively modulated the *P. infestans* defense response (Fig. 4), while the resistance was impaired after lncRNA23468 silencing (Fig. 5).

The overexpression of lncRNA23468 in tomato suppressed the accumulation of miR482b, resulting in the increased abundance of NBS-LRRs, and after lncRNA23468 silencing, the opposite was observed (Figs. 4a and 5a). In other words, lncRNA23468 functions as a ceRNA to modulate *NBS-LRR* genes by decoying miR482b, which enhances tomato resistance to *P. infestans*. Similarly, the lncRNA Slylnc0195 might function as a ceRNA to protect miR166 targets, class III HD-Zip transcription factor genes, by binding to miR166 via target mimicry in the tomato response to TYLCV^33^.

In summary, lncRNA23468 increases resistance to *P. infestans* infection in tomato. From our results in the lncRNA23468-silencing, lncRNA23468-overexpressing and mlncRNA23468-overexpressing tomato, we propose that lncRNA23468 increases the expression levels of *NBS-LRRs* by the repression of miR482b. This mechanism would allow for a response to *P. infestans* stress through lncRNA23468-induced downregulation of miR482b, which increases NBS-LRRs, thus promoting tomato resistance to *P. infestans*. Our results provide insight into an effective posttranscriptional regulation mechanism of lncRNA and demonstrate that the lncRNA23468-miR482b-NBS-LRR network is an important component of the *P. infestans* network in tomato.

## Electronic supplementary material

SUPPLEMENTAL MATERIAL
