# Supplementary material for: Tomato lncRNA23468 functions as a competing endogenous RNA to modulate NBS-LRR genes by decoying miR482b in the tomato-Phytophthora infestans interaction
Source: Hortic Res. 2019 Feb 1;6:28. doi: 10.1038/s41438-018-0096-0 (PMC6355781; doi:10.1038/s41438-018-0096-0)
Supplement: Supplementary file 1 — SUPPLEMENTAL MATERIAL [file 41438_2018_96_MOESM1_ESM.docx]

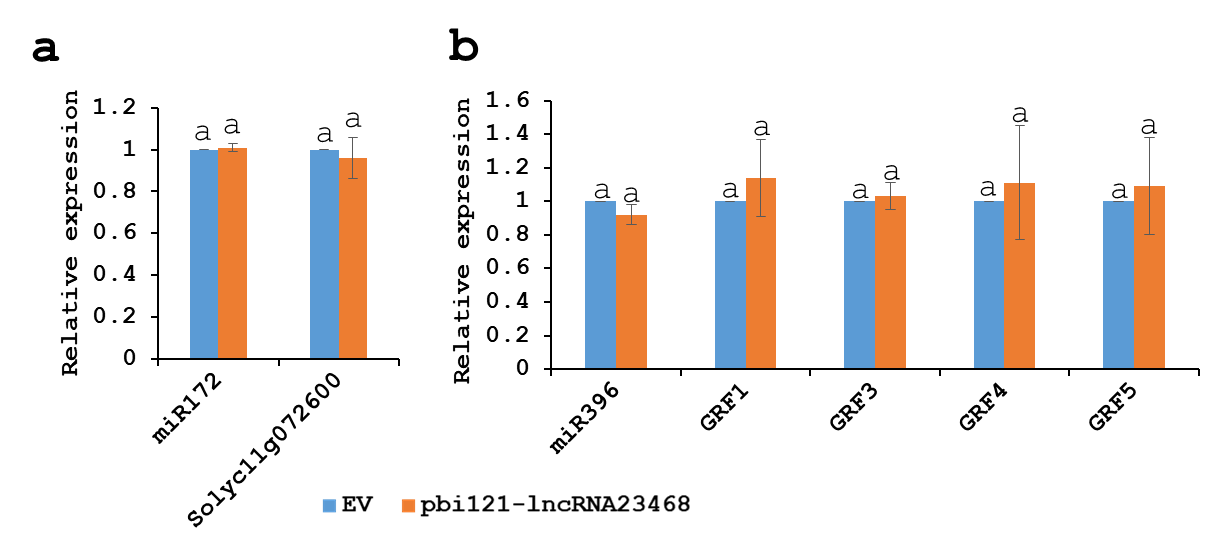


**Fig S1** The expression levels of miR172, miR396 and their target genes after overexpression of lncRNA23468 in tomato leaves. **a** miR172 and its target gene. **b** miR396 and its targets


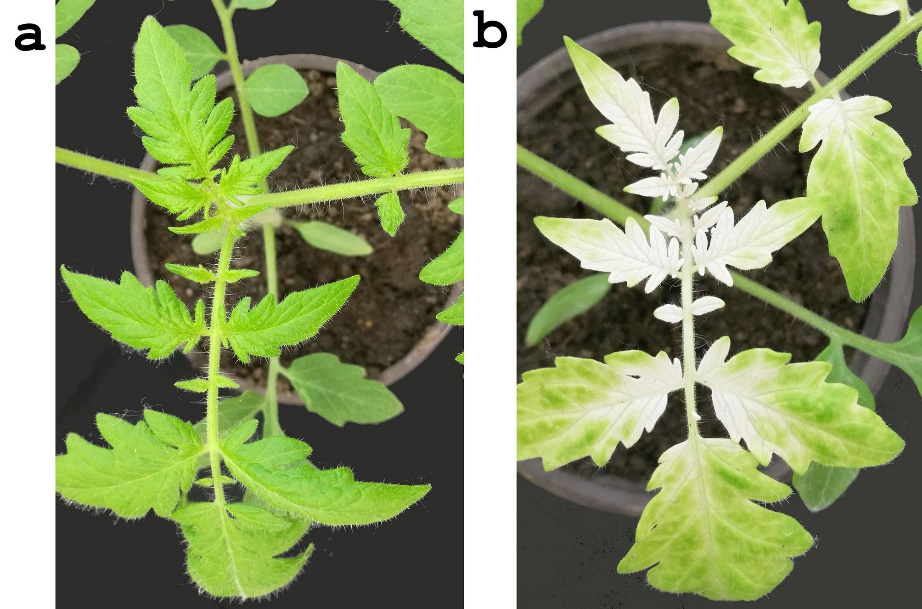


**Fig. S2** PDS as visual maker for silencing efficiency. **a** Control tomato plants. **b** Tomato plants treated with the PDS gene silencing constructs pTRV1 and pTRV2-PDS


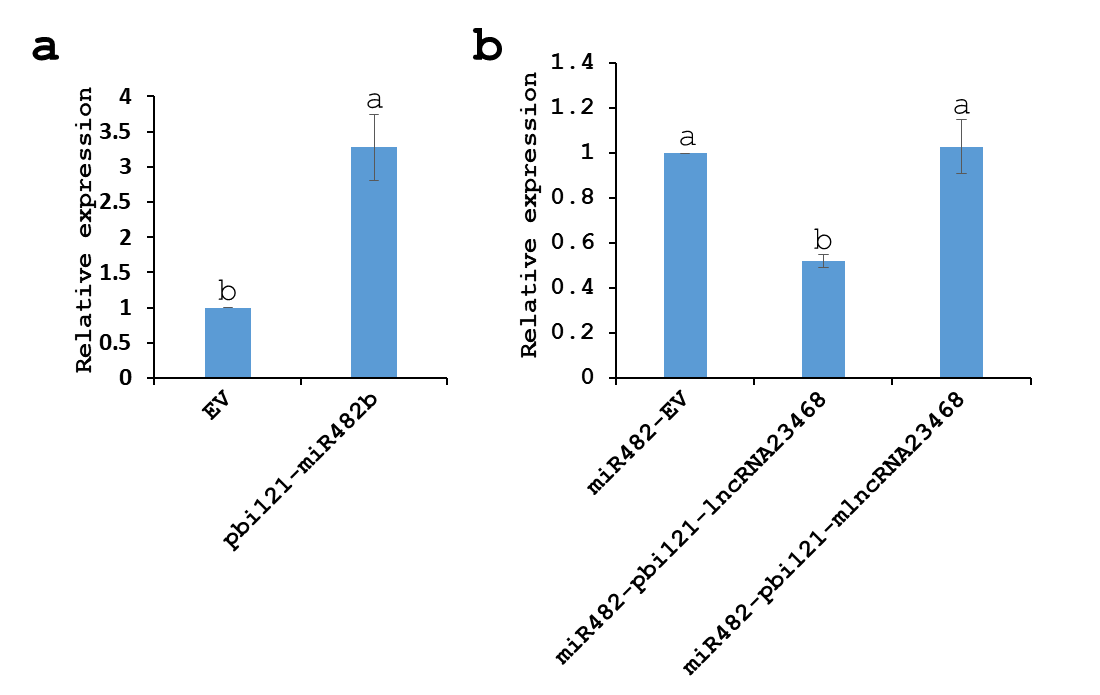


**Fig. S3** LncRNA23468 suppresses the expression of miR482b in *Nicotiana* systems. **a** The expression levels of miR482b in the tobacco leaves that overexpressed miR482b. **b** the expression levels of the miR482b after *Agrobacterium* harboring pBI121-lncRNA23468 and mlncRNA23468 were introduced into the tobacco leaves that expressed miR482b.

**Table S1** Primers in this study

| **Gene name** | **primer sequence (5'-3')** | **Function** |
| --- | --- | --- |
| l23468F | CGGGATCCAAAAATAAAAGAAAGCTTGCACG | lncRNA23468 Cloning* |
| l23468R | CGAGCTCGGACCGGATAATGAAGATGGT |
| ml23468-1R | GATAGCTATTCGGGCGAATCTGTCATAATGTAAATGTGG | construction of the mutation ^#^ |
| ml23468-2F | GATTCGCCCGAATAGCTATCAAAGAAACTGCATTTC | construction of the mutation ^#^ |
| vlncRNA23468F | CGACGACAAGACCCTGACAGATTGGGCGGAATAGG | VIGS |
| vlncRNA23468R | GAGGAGAAGAGCCCTTTCATGCCGACATGCTTCTC |
| vNBS-LRRF | CGACGACAAGACCCTTGCGCAACAGATTGGGTATT | VIGS |
| vNBS-LRRR | GAGGAGAAGAGCCCTTTCAACTACAGGTGGTGCAG |
| vPDS | CGACGACAAGACCCTGGCATCAACTTTATAAACC | VIGS |
| vPDS | GAGGAGAAGAGCCCTTTCAGTTTTCTGTCAAACC |
| qlncRNA23468F | GGTGCAATTAGCCAAAGGAGG | qRT-PCR |
| qlncRNA23468R | GAGGATGAGAGCTGGAAGCT |
| qlncRNA01308F | TACTGCCTCAGAGTTCCATTTG | qRT-PCR |
| qlncRNA01308R | CCTTTATATGGAGGTCGCAATGA |
| qlncRNA13262F | TTCCTGTATGATGTGGGTTTCA | qRT-PCR |
| qlncRNA13262R | TCTCCATGTCCTAGGCAAATTC |
| *P. infestans* actin F | GTTCCTGAGTTTTTGCTCCATC | qRT-PCR |
| *P. infestans* actin R | GCAGACCCTTTGCTACTACCTT |
| Tomato actin F | TGTGTTGGACTCTGGTGATGGTGT | qRT-PCR |
| Tomato actin R | ATCCAAACGAAGAATGGCATGCGG |
| miR172 | AGAATCTTGATGATGCTGCAT | qRT-PCR |
| miR396 | CGTTCCACAGCTTTCTTGAACTG | qRT-PCR |
| Solyc11g072600F | GCATAGTCAGGTCGGAACAA | qRT-PCR |
| Solyc11g072600R | GTTGGGACCAGAGATTTGAGAG |
| GRF1F | TCCTCAAAGCTCCTCATTCATC | qRT-PCR |
| GRF1R | TCCATCTGTTCTTCTGCATCTC |
| GRF3F | ATATTGCGAGCGGCACAT | qRT-PCR |
| GRF3R | TCCCGAATTAGCAACAGAATACA |
| GRF4F | CTGGAGAGGCAAGCTATGATTT | qRT-PCR |
| GRF4R | TCCAGTAGCCGATGTAGTATGT |
| GRF5F | TCCATGGAATGACTGCTGATG | qRT-PCR |
| GRF5R | ACCACATGCTGTCTGTGTTAG |

*PCR amplification was performed as follows: one cycle at 94 ℃ for 5 min; 35 cycles at 94 ℃ for 30 s, 60 ℃ for 30 s, and 72 ℃ for 1 min 30 s; and a final extension at 72 ℃ for 7 min

^#^ Both the first and the second round of PCR amplification were performed as follows: one cycle at 94 ℃ for 5 min; 35 cycles at 94 ℃ for 30 s, 60 ℃ for 30 s, and 72 ℃ for 1 min; and a final extension at 72 ℃ for 7 min

**Table S2** Disease grade of the VIGS tomato after infection with *Phytophthora infestans*

| **Disease grade** | **leaf** | **stem** |
| --- | --- | --- |
| 0 | lesion area = 0 | lesion area = 0 |
| 1 | lesion area ≤5% | lesion area = 0 |
| 2 | 5% < lesion area ≤ 15% | lesion area = 0 |
| 3 | 15% < lesion area ≤ 30% | lesion area = 0 |
| 4 | 30% < lesion area ≤ 60% | 10% < lesion area ≤ 30% |
| 5 | 60% < lesion area ≤ 90% | 30% < lesion area ≤ 50% |
| 6 | 90% < lesion area | 50% < lesion area |

**Table S3** LncRNAs from the OE482 and Slz samples

| **LncRNA name** | **Class code** | **Locus** | **OE482 FPKM** | **Slz FPKM** |
| --- | --- | --- | --- | --- |
| lncRNA02356 | "u" | chr01:71612477-71617923 | 3.34276 | 38.3411 |
| lncRNA09205 | "u" | chr02:8767119-8770231 | 12.398 | 1.00316 |
| lncRNA11846 | "u" | chr03:8640901-8641955 | 1.42964 | 0 |
| lncRNA18648 | "u" | chr04:25305418-25307457 | 1.12426 | 0 |
| lncRNA22366 | "o" | chr05:26874203-26884273 | 6.63026 | 0.289123 |
| lncRNA22375 | "u" | chr05:26986197-26988606 | 1.84244 | 0 |
| lncRNA23502 | "o" | chr06:8156978-8160867 | 2.07435 | 14.1597 |
| lncRNA29518 | "u" | chr07:50894303-50896783 | 11.379 | 43.3957 |
| lncRNA30729 | "u" | chr08:3346073-3350651 | 15.7443 | 0.544344 |
| lncRNA32893 | "u" | chr08:47779393-47784409 | 2.56725 | 22.7442 |
| lncRNA37657 | "u" | chr10:6518260-6519950 | 33.9325 | 0 |
| lncRNA38123 | "x" | chr10:45829633-45832992 | 3.45381 | 29.3275 |
| lncRNA39492 | "u" | chr10:6608599-6610747 | 0 | 0.750767 |
| lncRNA40885 | "x" | chr10:64119317-64122790 | 0 | 2.23954 |
| lncRNA41861 | "u" | chr11:17601674-17604431 | 0.760631 | 0 |
| lncRNA41867 | "u" | chr11:17642122-17646109 | 0.987147 | 0 |
| lncRNA43655 | "u" | chr11:17300905-17302097 | 2.08762 | 0 |
| lncRNA43657 | "u" | chr11:17304539-17306455 | 3.02561 | 0 |
| lncRNA43658 | "u" | chr11:17306573-17308556 | 1.83587 | 0 |
| lncRNA43980 | "u" | chr11:41062003-41062929 | 0 | 3.7626 |
| lncRNA45266 | "u" | chr12:7725752-7727707 | 1.06209 | 0 |
| lncRNA45739 | "x" | chr12:43667109-43672072 | 1.64922 | 0 |
| lncRNA09219 | "x" | chr02:9865099-9867410 | 1.15525 | 10.5893 |
| lncRNA20708 | "u" | chr05:28583765-28585143 | 0 | 1.1032 |
| lncRNA24909 | "x" | chr06:43627762-43628351 | 7.28282 | 104.1 |
| lncRNA25076 | "u" | chr06:45307481-45311639 | 3.55588 | 12.5574 |
| lncRNA44607 | "u" | chr11:53264511-53265257 | 5.326 | 37.0543 |
| lncRNA45011 | "x" | chr12:3460051-3461846 | 1.43909 | 13.3209 |
| lncRNA45738 | "x" | chr12:43667109-43672072 | 1.40554 | 0 |
| lncRNA18796 | "x" | chr04:39051199-39055010 | 1.35519 | 6.06465 |
| lncRNA25525 | "u" | chr06:15465756-15466297 | 6.10057 | 0 |
| lncRNA40666 | "x" | chr10:62204302-62205882 | 0 | 0.701634 |
| lncRNA46584 | "x" | chr12:541919-547222 | 2.29321 | 12.2502 |
| lncRNA20835 | "u" | chr05:41171075-41172320 | 1.05939 | 0 |
| lncRNA22369 | "u" | chr05:26888635-26889507 | 1.65694 | 0 |
| lncRNA25526 | "u" | chr06:15470483-15471583 | 1.16912 | 0 |
| lncRNA30728 | "u" | chr08:3343956-3344569 | 3.49603 | 0 |
| lncRNA34456 | "u" | chr09:16982111-16983645 | 0 | 0.953663 |
| lncRNA41860 | "u" | chr11:17596929-17598477 | 0.776493 | 0 |
| lncRNA00542 | "x" | chr00:4196742-4198543 | 46879.7 | 144729 |
| lncRNA23645 | "u" | chr06:21234643-21234930 | 48.559 | 0 |
| lncRNA41865 | "u" | chr11:17623122-17624297 | 1.0648 | 0 |
| lncRNA47336 | "u" | chr12:29061620-29062510 | 1.53968 | 0 |
| lncRNA23442 | "u" | chr06:4232429-4233395 | 0 | 1.66561 |
| lncRNA27434 | "u" | chr07:7607939-7608831 | 0 | 1.44613 |
| lncRNA27605 | "u" | chr07:26004018-26005227 | 0.827377 | 0 |
| lncRNA30959 | "u" | chr08:26066003-26066273 | 0 | 77.4483 |
| lncRNA37796 | "x" | chr10:20841244-20846139 | 0.69627 | 3.70459 |
| lncRNA43668 | "u" | chr11:18012764-18013485 | 2.07463 | 0 |
| lncRNA38671 | "x" | chr10:60978907-60979637 | 43.442 | 142.183 |
| lncRNA14959 | "u" | chr03:49414952-49417161 | 2.56209 | 11.9966 |
| lncRNA32685 | "u" | chr08:27239532-27241317 | 0.980916 | 0 |
| lncRNA41812 | "u" | chr11:15200943-15201214 | 904.052 | 3080.51 |
| lncRNA00637 | "o" | chr00:9795292-9833981 | 15582.6 | 46382.1 |
| lncRNA18769 | "u" | chr04:37561909-37562968 | 0 | 0.909073 |
| lncRNA46405 | "u" | chr12:64659974-64660372 | 0 | 8.21171 |
| lncRNA04865 | "u" | chr01:57683417-57683863 | 0 | 5.32729 |
| lncRNA16937 | "x" | chr04:47172967-47174993 | 1.4101 | 0 |
| lncRNA27745 | "u" | chr07:46362945-46363963 | 0 | 0.965116 |
| lncRNA32370 | "u" | chr08:2759045-2759544 | 0 | 3.65846 |
| lncRNA11688 | "x" | chr03:4354971-4357698 | 0.712876 | 5.71795 |
| lncRNA18623 | "u" | chr04:24650570-24652926 | 2.10492 | 9.29105 |
| lncRNA22164 | "x" | chr05:10567963-10570967 | 11.5853 | 32.3351 |
| lncRNA11680 | "u" | chr03:3685527-3686259 | 1.69407 | 0 |
| lncRNA41864 | "u" | chr11:17621923-17622853 | 1.14222 | 0 |
| lncRNA14614 | "u" | chr03:37281049-37281693 | 2.01059 | 0 |
| lncRNA17148 | "u" | chr04:53840122-53840996 | 1.24309 | 0 |
| lncRNA36212 | "u" | chr09:23275646-23276154 | 3.68813 | 0 |
| lncRNA29504 | "u" | chr07:49633718-49637170 | 9.18544 | 26.3634 |
| lncRNA03075 | "x" | chr01:80773724-80784683 | 0.960985 | 4.61048 |
| lncRNA20676 | "u" | chr05:27521494-27522294 | 1.27168 | 0 |
| lncRNA43857 | "x" | chr11:35149007-35149884 | 0.919772 | 0 |
| lncRNA14550 | "u" | chr03:30514188-30514698 | 740.828 | 2078.69 |
| lncRNA18985 | "x" | chr04:50279182-50283154 | 2.07056 | 21.2739 |
| lncRNA40669 | "x" | chr10:62218430-62223678 | 41.5088 | 118.267 |
| lncRNA22458 | "u" | chr05:34100171-34100423 | 40170.1 | 119457 |
| lncRNA31565 | "u" | chr08:56364579-56368874 | 0.975525 | 3.72738 |
| lncRNA32553 | "u" | chr08:10580512-10582309 | 2.39242 | 9.41055 |
| lncRNA41701 | "u" | chr11:10078331-10080798 | 2.8529 | 10.2511 |
| lncRNA17203 | "u" | chr04:54728518-54729269 | 1.1955 | 0 |
| lncRNA18651 | "u" | chr04:25312667-25313478 | 1.0574 | 0 |
| lncRNA38646 | "u" | chr10:60819341-60820186 | 1.0296 | 0 |
| lncRNA43654 | "u" | chr11:17299439-17300036 | 1.89779 | 0 |
| lncRNA45523 | "u" | chr12:32458083-32458527 | 4.20416 | 0 |
| lncRNA46069 | "u" | chr12:56885118-56891798 | 1.8896 | 0.444552 |
| lncRNA18133 | "u" | chr04:2640422-2641312 | 0.980076 | 0 |
| lncRNA27606 | "u" | chr07:26005911-26006489 | 2.00842 | 0 |
| lncRNA33093 | "u" | chr08:53269049-53269981 | 0.852601 | 0 |
| lncRNA41629 | "u" | chr11:8231157-8232059 | 1.01282 | 0 |
| lncRNA07019 | "x" | chr02:15054082-15057417 | 1.19868 | 4.98737 |
| lncRNA28146 | "u" | chr07:58348972-58351088 | 0.929193 | 5.31262 |
| lncRNA07657 | "x" | chr02:34275085-34275924 | 14.8783 | 48.7881 |
| lncRNA23493 | "x" | chr06:7557407-7560259 | 0.604046 | 4.05171 |
| lncRNA45503 | "x" | chr12:30898738-30902533 | 0.937498 | 3.62806 |
| lncRNA14549 | "u" | chr03:30513154-30513437 | 6313.06 | 16834.6 |
| lncRNA08352 | "x" | chr02:41925428-41948678 | 0.617954 | 1.73776 |
| lncRNA14960 | "u" | chr03:49434807-49437447 | 4.6831 | 13.7119 |
| lncRNA34664 | "x" | chr09:43130709-43133920 | 3.21787 | 9.43935 |
| lncRNA41189 | "u" | chr11:2007400-2007713 | 459.278 | 1271.46 |
| lncRNA17202 | "x" | chr04:54722696-54728243 | 1.72089 | 0.285301 |
| lncRNA04140 | "j" | chr01:1897088-1903877 | 128.888 | 433.263 |
| lncRNA23224 | "x" | chr06:714188-716146 | 31.3531 | 95.2137 |
| lncRNA43669 | "u" | chr11:18016410-18016851 | 3.44192 | 0 |
| lncRNA46444 | "x" | chr12:64967477-64968224 | 9.06702 | 44.3117 |
| lncRNA30833 | "u" | chr08:7906730-7909250 | 4.97469 | 14.1171 |
| lncRNA05741 | "x" | chr01:78285183-78287680 | 2.90219 | 38.9653 |
| lncRNA12185 | "x" | chr03:25046073-25049407 | 14.3234 | 35.0315 |
| lncRNA30673 | "u" | chr08:2620472-2620697 | 1038.1 | 3562.76 |
| lncRNA32515 | "x" | chr08:7482953-7485450 | 0.584372 | 6.95754 |
| lncRNA22549 | "u" | chr05:46935916-46936235 | 0 | 12.1933 |
| lncRNA31590 | "u" | chr08:56724562-56726577 | 4.9805 | 0.459396 |
| lncRNA36314 | "u" | chr09:35878443-35878862 | 1172.12 | 2914.58 |
| lncRNA16083 | "x" | chr04:323027-323963 | 5.35791 | 44.9407 |
| lncRNA37697 | "u" | chr10:8881177-8886438 | 0.319492 | 1.62808 |
| lncRNA07083 | "x" | chr02:17631337-17633397 | 4.53114 | 13.0969 |
| lncRNA46435 | "x" | chr12:64903430-64906621 | 1.55659 | 8.5207 |
| lncRNA27873 | "x" | chr07:53217866-53220281 | 1.03579 | 8.32711 |
| lncRNA47437 | "x" | chr12:35134469-35138297 | 6.79833 | 1.70717 |
| lncRNA41817 | "u" | chr11:15257615-15257836 | 13307.2 | 32365.5 |
| lncRNA21371 | "u" | chr05:63260371-63265148 | 0.933062 | 3.06491 |
| lncRNA29633 | "x" | chr07:54691601-54697262 | 0.358794 | 3.07052 |
| lncRNA26273 | "u" | chr06:37646896-37650664 | 15.8323 | 0.443677 |
| lncRNA28414 | "x" | chr07:61773086-61774158 | 1.01317 | 8.50173 |
| lncRNA39550 | "u" | chr10:10808705-10811548 | 6.08029 | 14.9829 |
| lncRNA30781 | "x" | chr08:5087905-5090801 | 2.22852 | 6.44909 |
| lncRNA38152 | "x" | chr10:46963490-46964619 | 1.08549 | 7.02918 |
| lncRNA29519 | "u" | chr07:50897678-50898817 | 0.941229 | 7.88292 |
| lncRNA34504 | "u" | chr09:20180877-20183297 | 3.24232 | 0.213517 |
| lncRNA18588 | "u" | chr04:21529333-21531573 | 0.544954 | 3.3215 |
| lncRNA15765 | "x" | chr03:61676280-61677216 | 20.6134 | 84.8332 |
| lncRNA29654 | "u" | chr07:55282888-55283719 | 35.1843 | 82.8046 |
| lncRNA12629 | "u" | chr03:49515500-49519687 | 1.35796 | 3.85043 |
| lncRNA20542 | "u" | chr05:12361262-12362618 | 1.95713 | 7.64817 |
| lncRNA36130 | "u" | chr09:15047095-15049228 | 0.585031 | 3.39618 |
| lncRNA14823 | "x" | chr03:46562094-46564618 | 6.22461 | 2.14406 |
| lncRNA20674 | "u" | chr05:27110376-27113911 | 1.83713 | 0.234826 |
| lncRNA20673 | "x" | chr05:27105705-27110273 | 1.08598 | 0.159488 |
| lncRNA40966 | "x" | chr10:64782861-64789180 | 0.183204 | 3.04 |
| lncRNA02614 | "x" | chr01:75096579-75102455 | 0.799093 | 7.16019 |
| lncRNA45215 | "x" | chr12:6328023-6333883 | 2.65544 | 0.0742034 |
| lncRNA15743 | "x" | chr03:61383764-61386408 | 12.0594 | 42.4592 |
| lncRNA16936 | "x" | chr04:47172967-47174993 | 1.50837 | 0 |
| lncRNA09758 | "x" | chr02:31309551-31310294 | 18.2971 | 46.8429 |
| lncRNA31746 | "x" | chr08:58824854-58826185 | 0.762039 | 8.54656 |
| lncRNA09225 | "x" | chr02:10982790-10987713 | 0.591196 | 3.95712 |
| lncRNA48003 | "x" | chr12:62086486-62092749 | 2.26671 | 8.12334 |
| lncRNA25529 | "u" | chr06:15476486-15480121 | 1.78072 | 0.124619 |
| lncRNA43614 | "u" | chr11:14692799-14693846 | 11.7018 | 29.1669 |
| lncRNA21045 | "u" | chr05:57485278-57489098 | 4.20403 | 1.49593 |
| lncRNA41820 | "x" | chr11:15308250-15340263 | 333.784 | 1013 |
| lncRNA12562 | "u" | chr03:47621943-47622450 | 1.73117 | 0 |
| lncRNA26345 | "x" | chr06:38377439-38378810 | 8.32027 | 37.8944 |
| lncRNA41821 | "o" | chr11:15308250-15340263 | 548.015 | 2113.35 |
| lncRNA46441 | "u" | chr12:64948903-64950000 | 2.41684 | 8.25424 |
| lncRNA21370 | "x" | chr05:63256743-63259307 | 20.7615 | 58.2767 |
| lncRNA18401 | "u" | chr04:6108896-6112164 | 44.0948 | 94.8302 |
| lncRNA42959 | "x" | chr11:1345706-1348486 | 4.7796 | 1.00449 |
| lncRNA06482 | "x" | chr01:86643324-86647899 | 2.91359 | 15.1357 |
| lncRNA37370 | "x" | chr10:1163273-1165705 | 3.5246 | 10.2684 |
| lncRNA02166 | "x" | chr01:67873051-67875885 | 1.88743 | 6.24548 |
| lncRNA04049 | "u" | chr01:906484-907372 | 31.1553 | 67.9394 |
| lncRNA23824 | "x" | chr06:29430734-29434224 | 1.93744 | 7.9657 |
| lncRNA28339 | "x" | chr07:60916507-60919031 | 2.34402 | 9.75812 |
| lncRNA19998 | "x" | chr05:1028560-1029190 | 6.02751 | 22.4623 |
| lncRNA25170 | "x" | chr06:411510-413206 | 1.23983 | 4.28433 |
| lncRNA05581 | "x" | chr01:76330160-76349305 | 0.00350679 | 4.50905 |
| lncRNA29134 | "x" | chr07:7609378-7611423 | 2.16461 | 5.92998 |
| lncRNA43859 | "u" | chr11:35151690-35154480 | 1.90514 | 0.495419 |
| lncRNA19805 | "x" | chr04:63070049-63071183 | 9.70335 | 31.1103 |
| lncRNA33763 | "x" | chr08:62171933-62173538 | 40.85 | 292.907 |
| lncRNA19601 | "u" | chr04:60900296-60901482 | 4.37997 | 0.644095 |
| lncRNA31693 | "x" | chr08:58133141-58134069 | 1.90504 | 12.789 |
| lncRNA32906 | "x" | chr08:48053463-48059479 | 5.6728 | 2.54161 |
| lncRNA16611 | "u" | chr04:9360920-9362126 | 3.92498 | 0.478373 |
| lncRNA30683 | "o" | chr08:2722852-2724468 | 39.2537 | 19.2934 |
| lncRNA03643 | "u" | chr01:86595981-86596431 | 3.38648 | 24.2749 |
| lncRNA26783 | "x" | chr06:43287460-43290800 | 1.95707 | 0.433717 |
| lncRNA21002 | "u" | chr05:56135347-56137872 | 4.67033 | 0.00138088 |
| lncRNA45655 | "x" | chr12:40295365-40299365 | 1.46291 | 4.73768 |
| lncRNA45823 | "x" | chr12:45744236-45745480 | 6.66081 | 1.26114 |
| lncRNA37604 | "x" | chr10:4409839-4412664 | 0.821491 | 5.37289 |
| lncRNA01422 | "u" | chr01:5184107-5185313 | 5.79706 | 1.62169 |
| lncRNA15609 | "x" | chr03:59947284-59948577 | 3.83132 | 11.771 |
| lncRNA10804 | "x" | chr02:42931627-42936171 | 1.26953 | 4.62081 |
| lncRNA12884 | "x" | chr03:55819510-55820894 | 1.88982 | 21.1311 |
| lncRNA33473 | "x" | chr08:58707666-58711970 | 0.988219 | 4.99404 |
| lncRNA44058 | "x" | chr11:45816412-45818558 | 0.174328 | 2.81264 |
| lncRNA03076 | "x" | chr01:80773724-80784683 | 0.926948 | 2.71791 |
| lncRNA28967 | "x" | chr07:2646085-2647739 | 17.5472 | 40.3734 |
| lncRNA07882 | "x" | chr02:36900694-36901485 | 13.8606 | 47.5777 |
| lncRNA34719 | "x" | chr09:49172212-49173725 | 23.8992 | 9.7415 |
| lncRNA32492 | "x" | chr08:6854442-6855225 | 1.01998 | 7.23896 |
| lncRNA30974 | "x" | chr08:27458007-27462628 | 7.00386 | 17.2705 |
| lncRNA19319 | "u" | chr04:57173009-57173510 | 2.99282 | 15.069 |
| lncRNA10596 | "x" | chr02:40875351-40878689 | 0.575646 | 3.6248 |
| lncRNA32468 | "x" | chr08:5693335-5695525 | 3.01585 | 7.08428 |
| lncRNA38776 | "x" | chr10:61932878-61937470 | 1.16674 | 9.42565 |
| lncRNA12192 | "u" | chr03:25230500-25232857 | 2.14708 | 0.658202 |
| lncRNA27240 | "x" | chr07:2239341-2241090 | 31.921 | 86.225 |
| lncRNA35261 | "u" | chr09:64436810-64437061 | 31.1708 | 0 |
| lncRNA46904 | "x" | chr12:3892564-3893880 | 1.5654 | 9.33651 |
| lncRNA32585 | "u" | chr08:16759992-16760248 | 0 | 22.8033 |
| lncRNA34934 | "u" | chr09:59202515-59205989 | 0.195446 | 0.885444 |
| lncRNA37023 | "u" | chr09:64621821-64622117 | 0 | 9.19787 |
| lncRNA39931 | "x" | chr10:45390725-45391315 | 6.07848 | 17.7462 |
| lncRNA23386 | "x" | chr06:3053411-3057986 | 0.748581 | 3.4825 |
| lncRNA39588 | "u" | chr10:14029061-14033288 | 0.687303 | 0.127734 |
| lncRNA06533 | "u" | chr01:87173562-87176285 | 2.79294 | 1.07184 |
| lncRNA02061 | "u" | chr01:62507318-62510781 | 1.01379 | 0.292738 |
| lncRNA20539 | "u" | chr05:12107889-12109744 | 2.39337 | 5.8157 |
| lncRNA14589 | "x" | chr03:32616137-32618774 | 1.25953 | 3.23532 |
| lncRNA00322 | "x" | chr00:15165397-15166578 | 3.66171 | 0.832611 |
| lncRNA23816 | "x" | chr06:29256146-29260955 | 0.75386 | 0.0020234 |
| lncRNA17072 | "u" | chr04:52098297-52100313 | 2.0002 | 0.545015 |
| lncRNA47692 | "x" | chr12:45417432-45419715 | 0.00479264 | 4.89976 |
| lncRNA13491 | "x" | chr03:62685075-62686696 | 1.28189 | 3.73427 |
| lncRNA39051 | "x" | chr10:64482457-64484213 | 4.36023 | 21.4327 |
| lncRNA48014 | "x" | chr12:62213767-62215934 | 2.41805 | 13.5494 |
| lncRNA26609 | "x" | chr06:41319073-41320092 | 8.23612 | 18.6285 |
| lncRNA32804 | "u" | chr08:42000802-42003299 | 0.925795 | 2.49013 |
| lncRNA27150 | "x" | chr07:1194127-1196139 | 62.4969 | 22.0218 |
| lncRNA28126 | "u" | chr07:58040080-58040390 | 25.2566 | 96.3557 |
| lncRNA31985 | "x" | chr08:61456274-61458026 | 8.01016 | 39.1819 |
| lncRNA35894 | "x" | chr09:4230818-4251046 | 3.10095 | 1.67587 |
| lncRNA13367 | "x" | chr03:61377398-61378263 | 0.862446 | 4.60382 |
| lncRNA01782 | "u" | chr01:38140011-38140212 | 533.073 | 1975.64 |
| lncRNA22004 | "x" | chr05:6480259-6484488 | 0.213665 | 2.6471 |
| lncRNA08730 | "x" | chr02:45946476-45949249 | 5.82974 | 56.2779 |
| lncRNA19797 | "x" | chr04:63003944-63007770 | 2.0145 | 0.000589174 |
| lncRNA42467 | "x" | chr11:49761083-49762307 | 42.3765 | 10.6645 |
| lncRNA21281 | "x" | chr05:62083439-62086301 | 1.31523 | 3.60004 |
| lncRNA04442 | "x" | chr01:11691047-11695017 | 0.789472 | 0.232759 |
| lncRNA11059 | "x" | chr02:45475663-45480704 | 2.83107 | 1.26882 |
| lncRNA08342 | "o" | chr02:41829797-41904084 | 0 | 2.52256 |
| lncRNA39528 | "u" | chr10:8711275-8714068 | 1.24149 | 3.25773 |
| lncRNA32594 | "u" | chr08:16971930-16977614 | 0.996271 | 2.17452 |
| lncRNA05460 | "u" | chr01:74651812-74652504 | 1.23453 | 5.86491 |
| lncRNA18939 | "x" | chr04:48879772-48894993 | 0.272913 | 1.14879 |
| lncRNA05630 | "u" | chr01:76844074-76845064 | 4.90664 | 11.6581 |
| lncRNA33211 | "x" | chr08:55113673-55114740 | 1.73663 | 5.21709 |
| lncRNA14512 | "u" | chr03:29426060-29427404 | 0.936705 | 3.08596 |
| lncRNA20744 | "x" | chr05:30890663-30891656 | 3.19299 | 0.408017 |
| lncRNA16972 | "u" | chr04:49220263-49221654 | 0.769351 | 2.70611 |
| lncRNA27079 | "x" | chr07:234854-236052 | 27.6613 | 14.5576 |
| lncRNA39746 | "u" | chr10:29760928-29762093 | 0.421595 | 2.4145 |
| lncRNA19220 | "u" | chr04:55610090-55610990 | 23.1252 | 10.7269 |
| lncRNA37693 | "u" | chr10:8711275-8714068 | 1.15502 | 4.96985 |
| lncRNA20503 | "u" | chr05:10522633-10523715 | 0.352136 | 2.94473 |
| lncRNA45571 | "x" | chr12:35520254-35523028 | 2.28157 | 4.75476 |
| lncRNA20554 | "u" | chr05:14557792-14559783 | 0.85792 | 2.39561 |
| lncRNA06100 | "u" | chr01:82365342-82367926 | 3.53799 | 1.5153 |
| lncRNA22631 | "x" | chr05:55347630-55350234 | 0.764264 | 2.03559 |
| lncRNA38051 | "x" | chr10:41774498-41777578 | 0.914767 | 4.40398 |
| lncRNA46764 | "x" | chr12:2502479-2504956 | 6.71942 | 66.3118 |
| lncRNA18223 | "u" | chr04:3577412-3577650 | 27.6052 | 0 |
| lncRNA26988 | "j" | chr06:45263576-45269128 | 6.74623 | 0.0133288 |
| lncRNA12233 | "u" | chr03:29611902-29614016 | 0.197058 | 1.02613 |
| lncRNA17360 | "u" | chr04:57227466-57231033 | 2.60322 | 1.16055 |
| lncRNA08425 | "x" | chr02:42815857-42821280 | 11.7524 | 21.1547 |
| lncRNA18587 | "x" | chr04:21512730-21514390 | 0.582966 | 1.93244 |
| lncRNA32934 | "u" | chr08:49491039-49492086 | 3.54594 | 8.28783 |
| lncRNA19671 | "x" | chr04:61699525-61701530 | 0.42547 | 6.16134 |
| lncRNA03099 | "x" | chr01:80976339-80981277 | 1.17186 | 5.05405 |
| lncRNA07356 | "u" | chr02:30120523-30120770 | 142.786 | 439.94 |
| lncRNA25815 | "u" | chr06:30436565-30437534 | 1.11892 | 3.75067 |
| lncRNA35386 | "x" | chr09:65813909-65825428 | 13.863 | 3.69223 |
| lncRNA04085 | "u" | chr01:1393444-1394767 | 3.08216 | 6.93375 |
| lncRNA41940 | "u" | chr11:24820303-24826896 | 0.284978 | 1.25466 |
| lncRNA13019 | "x" | chr03:57498269-57504777 | 3.72365 | 0.485955 |
| lncRNA19505 | "x" | chr04:59767189-59767752 | 94.4218 | 171.434 |
| lncRNA46332 | "x" | chr12:64024999-64026610 | 13.4346 | 26.935 |
| lncRNA38637 | "x" | chr10:60742952-60747382 | 3.14312 | 7.07691 |
| lncRNA44753 | "x" | chr12:902665-908840 | 12.8479 | 43.9548 |
| lncRNA34653 | "u" | chr09:42295699-42296699 | 1.17522 | 3.84293 |
| lncRNA08408 | "x" | chr02:42621388-42622136 | 2.37485 | 8.98808 |
| lncRNA18542 | "u" | chr04:17311255-17313659 | 0.839563 | 0.187377 |
| lncRNA46763 | "x" | chr12:2502479-2504956 | 0.476458 | 9.88989 |
| lncRNA25531 | "x" | chr06:15679585-15681618 | 4.39249 | 8.52771 |
| lncRNA33044 | "u" | chr08:52252293-52254924 | 2.3882 | 4.87973 |
| lncRNA22394 | "u" | chr05:28301602-28301803 | 1853.62 | 4039.72 |
| lncRNA39964 | "u" | chr10:46357323-46358953 | 2.43884 | 5.36552 |
| lncRNA39569 | "u" | chr10:12065516-12067200 | 5.48722 | 10.6508 |
| lncRNA25220 | "x" | chr06:1076020-1078334 | 0.912112 | 0.0984952 |
| lncRNA31869 | "x" | chr08:60152857-60158284 | 3.74934 | 0.0196661 |
| lncRNA23538 | "u" | chr06:13152473-13160195 | 3.55412 | 0.0278951 |
| lncRNA29857 | "x" | chr07:58547668-58548690 | 29.0117 | 7.05322 |
| lncRNA46062 | "u" | chr12:55882814-55885965 | 2.66338 | 1.24808 |
| lncRNA21372 | "u" | chr05:63265205-63266628 | 0.614806 | 2.13437 |
| lncRNA31627 | "x" | chr08:57214925-57218896 | 1.19108 | 3.71512 |
| lncRNA39482 | "x" | chr10:6075873-6078934 | 5.93621 | 14.2909 |
| lncRNA42070 | "x" | chr11:35656919-35659559 | 4.07425 | 1.76076 |
| lncRNA45055 | "x" | chr12:3827847-3830773 | 5.26805 | 1.96862 |
| lncRNA47875 | "x" | chr12:48580799-48581476 | 14.5613 | 65.0809 |
| lncRNA11671 | "x" | chr03:2471903-2478161 | 0.393585 | 3.78403 |
| lncRNA47170 | "x" | chr12:10619412-10622877 | 0.000298427 | 0.646479 |
| lncRNA22181 | "x" | chr05:11856765-11860993 | 9.22578E-05 | 0.91942 |
| lncRNA15521 | "x" | chr03:59116973-59119928 | 0.999371 | 0.000190049 |
| lncRNA29872 | "x" | chr07:58743767-58744578 | 6.07527 | 13.1519 |
| lncRNA04516 | "u" | chr01:18235806-18238024 | 3.01943 | 6.00403 |
| lncRNA21140 | "x" | chr05:60131927-60136497 | 6.72819 | 32.0673 |
| lncRNA38005 | "u" | chr10:38507717-38509933 | 0.880309 | 0.100634 |
| lncRNA36416 | "u" | chr09:46073712-46076684 | 0.503095 | 1.29859 |
| lncRNA12121 | "u" | chr03:20440788-20444989 | 1.46596 | 0.662111 |
| lncRNA29849 | "x" | chr07:58478186-58501747 | 0.774204 | 0 |
| lncRNA26329 | "u" | chr06:38179144-38181178 | 0.344355 | 1.19859 |
| lncRNA12948 | "x" | chr03:56796655-56798610 | 5.73944 | 11.9037 |
| lncRNA10348 | "x" | chr02:38092291-38092723 | 2.67895 | 13.3268 |
| lncRNA20843 | "x" | chr05:41381950-41385231 | 13.1184 | 22.043 |
| lncRNA48017 | "x" | chr12:62241229-62242087 | 5.23526 | 11.5701 |
| lncRNA48384 | "x" | chr12:65379979-65380464 | 0.628415 | 0 |
| lncRNA34161 | "u" | chr09:3910919-3911719 | 1.19604 | 4.09978 |
| lncRNA11499 | "x" | chr03:210556-211788 | 0.876686 | 3.39925 |
| lncRNA14638 | "x" | chr03:41192651-41197317 | 1.07669 | 0.476042 |
| lncRNA07176 | "u" | chr02:21295102-21296725 | 3.47819 | 7.0037 |
| lncRNA26736 | "u" | chr06:42760056-42761338 | 41.4308 | 70.4537 |
| lncRNA31270 | "o" | chr08:50894362-50897010 | 0.219156 | 0.956153 |
| lncRNA04798 | "u" | chr01:52335185-52337100 | 0.163829 | 0.893457 |
| lncRNA47257 | "x" | chr12:17602668-17603003 | 544.185 | 1065.16 |
| lncRNA21046 | "u" | chr05:57490403-57491540 | 2.33167 | 0.646053 |
| lncRNA33041 | "x" | chr08:52209115-52215073 | 0.857865 | 2.65822 |
| lncRNA37584 | "x" | chr10:3983197-3987001 | 2.9219 | 12.5763 |
| lncRNA26089 | "u" | chr06:35439841-35440164 | 15.73 | 55.2315 |
| lncRNA21632 | "x" | chr05:1369867-1373977 | 6.13865 | 3.31875 |
| lncRNA23826 | "u" | chr06:29435893-29436877 | 1.03634 | 3.20265 |
| lncRNA24715 | "u" | chr06:41525347-41526197 | 2.76508 | 6.97286 |
| lncRNA44308 | "x" | chr11:50253545-50259742 | 1.68228 | 6.00422 |
| lncRNA11214 | "x" | chr02:47144005-47144841 | 0.79289 | 9.00436 |
| lncRNA29856 | "x" | chr07:58542009-58546861 | 2.87776 | 9.91799 |
| lncRNA06421 | "x" | chr01:85988825-85990630 | 0.877148 | 3.41091 |
| lncRNA40867 | "x" | chr10:63936549-63944280 | 7.57517 | 46.5001 |
| lncRNA43505 | "u" | chr11:9484589-9484947 | 7.99177 | 28.7948 |
| lncRNA46645 | "x" | chr12:1195795-1196489 | 8.29609 | 17.8861 |
| lncRNA39147 | "x" | chr10:733291-738148 | 0.129218 | 2.94143 |
| lncRNA05317 | "x" | chr01:72724332-72727265 | 0.460906 | 1.87686 |
| lncRNA43648 | "u" | chr11:17042794-17049029 | 14.026 | 8.30071 |
| lncRNA36107 | "u" | chr09:13496849-13498250 | 1.33906 | 3.43647 |
| lncRNA34914 | "u" | chr09:58814639-58817325 | 0.74133 | 0.186167 |
| lncRNA25741 | "x" | chr06:28279397-28280268 | 156.409 | 85.9661 |
| lncRNA08368 | "u" | chr02:42102660-42103114 | 5.3483 | 15.5214 |
| lncRNA00125 | "o" | chr00:10682530-10686239 | 0.762126 | 2.1611 |
| lncRNA39748 | "u" | chr10:29766383-29767418 | 1.77881 | 4.53082 |
| lncRNA11419 | "x" | chr02:49243309-49244057 | 1.29651 | 48.2674 |
| lncRNA23780 | "x" | chr06:27979944-27980604 | 1.85215 | 5.63713 |
| lncRNA27739 | "x" | chr07:45034948-45038999 | 4.58796 | 2.56816 |
| lncRNA06195 | "x" | chr01:83415884-83422712 | 1.99182 | 0.966393 |
| lncRNA32839 | "u" | chr08:45101267-45101780 | 1.53528 | 6.7776 |
| lncRNA47348 | "u" | chr12:29144752-29145941 | 1.48565 | 0.221895 |
| lncRNA25501 | "u" | chr06:13171771-13173940 | 0.734956 | 0.105478 |
| lncRNA07082 | "u" | chr02:17630203-17631268 | 2.43234 | 5.555 |
| lncRNA02092 | "u" | chr01:64788642-64790788 | 0.176154 | 0.75787 |
| lncRNA01817 | "u" | chr01:41730077-41732111 | 0.714017 | 0.116732 |
| lncRNA02183 | "u" | chr01:68128969-68129470 | 65.0376 | 114.593 |
| lncRNA27871 | "u" | chr07:53201289-53203567 | 0.892118 | 2.05328 |
| lncRNA24673 | "x" | chr06:41205363-41207945 | 0 | 1.55576 |
| lncRNA23654 | "x" | chr06:21427578-21428630 | 0 | 16.9037 |
| lncRNA11030 | "x" | chr02:45301572-45302284 | 4.46922 | 11.8926 |
| lncRNA39736 | "x" | chr10:28841569-28845152 | 1.47113 | 3.99292 |
| lncRNA14168 | "o" | chr03:9695210-9696126 | 6.10795 | 11.9704 |
| lncRNA45318 | "x" | chr12:11158125-11160494 | 3.20875 | 5.84619 |
| lncRNA10273 | "x" | chr02:37316258-37319906 | 1.53161 | 3.63455 |
| lncRNA28413 | "x" | chr07:61773086-61774158 | 0 | 7.80375 |
| lncRNA37807 | "x" | chr10:21044741-21078139 | 0 | 1.36154 |
| lncRNA41339 | "o" | chr11:3713758-3714507 | 7.3361 | 35.3576 |
| lncRNA10887 | "x" | chr02:43880801-43884639 | 0.577922 | 8.74948 |
| lncRNA37218 | "u" | chr09:66990613-66992085 | 10.5386 | 5.75626 |
| lncRNA34783 | "u" | chr09:53892524-53894770 | 2.57492 | 4.89692 |
| lncRNA36108 | "u" | chr09:13498345-13499727 | 0.580109 | 1.77227 |
| lncRNA28038 | "x" | chr07:56334685-56340326 | 5.51222 | 12.5811 |
| lncRNA21103 | "x" | chr05:59397891-59402194 | 0 | 0.999859 |
| lncRNA10186 | "x" | chr02:36228647-36237360 | 4.96273 | 2.76131 |
| lncRNA26727 | "u" | chr06:42684237-42686228 | 0.930893 | 0.272957 |
| lncRNA32883 | "x" | chr08:47337913-47339861 | 0.921643 | 0.257551 |
| lncRNA05971 | "u" | chr01:80950527-80951921 | 0.196926 | 1.15404 |
| lncRNA45271 | "x" | chr12:8877607-8878239 | 2.9132 | 8.62981 |
| lncRNA31923 | "u" | chr08:60692720-60694597 | 1.03438 | 0.286916 |
| lncRNA40143 | "x" | chr10:52372077-52383992 | 0 | 1.17446 |
| lncRNA14745 | "o" | chr03:45657120-45668274 | 0 | 1.16647 |
| lncRNA39377 | "u" | chr10:3434692-3435850 | 1.4637 | 0.31624 |
| lncRNA39574 | "x" | chr10:13229462-13230046 | 7.68214 | 0 |
| lncRNA14963 | "u" | chr03:49513121-49513691 | 0.817349 | 4.51557 |
| lncRNA36184 | "u" | chr09:20084601-20085445 | 0.413727 | 2.28903 |
| lncRNA23880 | "x" | chr06:30774363-30780559 | 9.69165 | 0 |
| lncRNA36787 | "x" | chr09:61224705-61225683 | 2.72464 | 0 |
| lncRNA39488 | "x" | chr10:6316477-6321985 | 4.24856 | 7.05776 |
| lncRNA46555 | "o" | chr12:298219-301809 | 1.91764 | 0.941745 |
| lncRNA36116 | "u" | chr09:13905314-13906468 | 1.36536 | 0.232204 |
| lncRNA20327 | "o" | chr05:5799500-5800416 | 262.445 | 442.667 |
| lncRNA19150 | "u" | chr04:54510954-54512864 | 45.0029 | 27.2954 |
| lncRNA36810 | "x" | chr09:61771569-61773997 | 1.18414 | 0.468268 |
| lncRNA06483 | "x" | chr01:86650093-86653299 | 1.87395 | 5.9368 |
| lncRNA12862 | "u" | chr03:54518571-54519240 | 0.628138 | 3.24814 |
| lncRNA12053 | "u" | chr03:15009129-15010656 | 1.01467 | 2.47611 |
| lncRNA15520 | "x" | chr03:59116973-59119928 | 0.294525 | 1.23541 |
| lncRNA09831 | "x" | chr02:32326545-32327386 | 0.425254 | 3.59582 |
| lncRNA39628 | "x" | chr10:18289417-18295394 | 0 | 5.01898 |
| lncRNA09481 | "u" | chr02:21909434-21911179 | 0.776417 | 0.143243 |
| lncRNA46962 | "u" | chr12:4623067-4623750 | 0.718611 | 3.37414 |
| lncRNA30727 | "u" | chr08:3341870-3343234 | 1.03584 | 0.19321 |
| lncRNA44430 | "x" | chr11:51612236-51617104 | 6.34531 | 3.79339 |
| lncRNA40757 | "x" | chr10:63001704-63003502 | 0 | 6.94426 |
| lncRNA02158 | "x" | chr01:67703304-67706749 | 8.13062 | 4.83376 |
| lncRNA41318 | "x" | chr11:3465993-3466654 | 28.5444 | 76.108 |
| lncRNA02840 | "x" | chr01:78183567-78186950 | 1.86663 | 3.43739 |
| lncRNA47980 | "x" | chr12:61766314-61778223 | 9.92271 | 5.23926 |
| lncRNA10646 | "x" | chr02:41380883-41385012 | 0.614258 | 1.36982 |
| lncRNA01061 | "u" | chr00:21592713-21594127 | 0.889045 | 0.172572 |
| lncRNA09678 | "x" | chr02:29992546-29993398 | 4.00611 | 13.8807 |
| lncRNA10581 | "x" | chr02:40801303-40804388 | 13.6965 | 35.1293 |
| lncRNA14916 | "u" | chr03:48491154-48491849 | 0.414992 | 5.21697 |
| lncRNA02088 | "x" | chr01:64680931-64684810 | 1.90362 | 0.300989 |
| lncRNA34109 | "x" | chr09:3296634-3297580 | 7.32574 | 1.5908 |
| lncRNA25403 | "x" | chr06:3339557-3340984 | 19.5398 | 0 |
| lncRNA32532 | "u" | chr08:9091502-9093431 | 0.657231 | 1.65764 |
| lncRNA38606 | "x" | chr10:60410500-60414275 | 1.39518 | 3.55077 |
| lncRNA33284 | "u" | chr08:56169923-56171243 | 0.151125 | 1.6452 |
| lncRNA08348 | "x" | chr02:41829797-41904084 | 2.19981 | 9.85822 |
| lncRNA12212 | "x" | chr03:27077049-27080500 | 2.17206 | 0.613406 |
| lncRNA23797 | "x" | chr06:28759228-28760112 | 6.43192 | 34.1796 |
| lncRNA20667 | "u" | chr05:26919218-26921547 | 1.65523 | 0.178797 |
| lncRNA23540 | "u" | chr06:13160740-13164929 | 1.6468 | 0 |
| lncRNA30958 | "u" | chr08:26065290-26065825 | 0.729748 | 6.99876 |
| lncRNA44932 | "x" | chr12:2734834-2737802 | 5.85006 | 1.72201 |
| lncRNA37229 | "j" | chr09:67076449-67077702 | 0.987592 | 3.59309 |
| lncRNA23511 | "u" | chr06:9559353-9561028 | 0.824926 | 0.204315 |
| lncRNA03468 | "u" | chr01:84895860-84897307 | 1.33605 | 0.38264 |
| lncRNA34650 | "u" | chr09:42247264-42248632 | 0.262791 | 1.15759 |
| lncRNA29331 | "x" | chr07:30493328-30496781 | 7.71978 | 4.63206 |
| lncRNA04277 | "x" | chr01:4205343-4209004 | 1.28596 | 3.69135 |
| lncRNA28084 | "x" | chr07:57174888-57179154 | 2.32242 | 8.60844 |
| lncRNA08830 | "x" | chr02:47064809-47066611 | 1.00306 | 2.33935 |
| lncRNA46853 | "x" | chr12:3450318-3452069 | 0.233408 | 2.83748 |
| lncRNA43716 | "u" | chr11:21625874-21626197 | 4.25461 | 34.9568 |
| lncRNA46873 | "u" | chr12:3602314-3605856 | 1.7726 | 0.905111 |
| lncRNA11078 | "u" | chr02:45791487-45791799 | 52.772 | 18.005 |
| lncRNA39568 | "x" | chr10:12062103-12063761 | 0.332938 | 1.09193 |
| lncRNA42016 | "u" | chr11:32856291-32857728 | 0.230281 | 0.963862 |
| lncRNA23616 | "u" | chr06:20458608-20459587 | 0.249143 | 1.96527 |
| lncRNA22587 | "x" | chr05:51253194-51263703 | 0 | 0.726631 |
| lncRNA28296 | "x" | chr07:60285912-60291302 | 0.407927 | 3.21214 |
| lncRNA36940 | "x" | chr09:63444404-63445334 | 3.34383 | 12.165 |
| lncRNA19796 | "x" | chr04:63003944-63007770 | 0.0318184 | 2.04514 |
| lncRNA30903 | "u" | chr08:17279385-17280204 | 2.34056 | 0.639507 |
| lncRNA12211 | "x" | chr03:27076341-27076912 | 5.15785 | 1.29744 |
| lncRNA22155 | "x" | chr05:10448357-10450024 | 1.33059 | 0 |
| lncRNA08732 | "x" | chr02:45991463-45992145 | 5.50392 | 12.0458 |
| lncRNA26798 | "x" | chr06:43427532-43430452 | 0.936938 | 11.3616 |
| lncRNA19399 | "u" | chr04:58249763-58250804 | 7.37353 | 13.4143 |
| lncRNA23136 | "u" | chr05:64562930-64563824 | 2.19769 | 0.662109 |
| lncRNA21001 | "u" | chr05:56135347-56137872 | 4.5335 | 8.25045 |
| lncRNA22371 | "u" | chr05:26919218-26921547 | 0.98727 | 0 |
| lncRNA24538 | "o" | chr06:39866795-39869543 | 0.545681 | 1.4306 |
| lncRNA18033 | "x" | chr04:1262091-1262802 | 0 | 11.3119 |
| lncRNA30009 | "x" | chr07:60389210-60392375 | 0.358258 | 1.08303 |
| lncRNA05913 | "x" | chr01:80331659-80334218 | 0 | 4.03753 |
| lncRNA10775 | "u" | chr02:42712628-42713745 | 17.745 | 10.375 |
| lncRNA43473 | "x" | chr11:8325083-8330973 | 14.0276 | 0 |
| lncRNA07239 | "u" | chr02:25517454-25518361 | 0.276148 | 1.92668 |
| lncRNA34392 | "u" | chr09:11630716-11631219 | 0.802562 | 5.61099 |
| lncRNA02799 | "u" | chr01:77694648-77695108 | 14.5667 | 5.6274 |
| lncRNA15026 | "u" | chr03:51577259-51578055 | 5.8364 | 11.3432 |
| lncRNA13847 | "x" | chr03:1938145-1944988 | 2.0452 | 3.5252 |
| lncRNA14590 | "x" | chr03:32630818-32633611 | 0.552361 | 1.24429 |
| lncRNA20955 | "u" | chr05:53682767-53683211 | 3.17385 | 10.2054 |
| lncRNA36266 | "u" | chr09:29712331-29715036 | 2.8245 | 1.4878 |
| lncRNA33036 | "x" | chr08:52091230-52092726 | 0.255242 | 0.93246 |
| lncRNA00239 | "u" | chr00:12632396-12635202 | 1.23107 | 2.6328 |
| lncRNA27041 | "x" | chr06:45809711-45812769 | 0.948266 | 0 |
| lncRNA03804 | "x" | chr01:88339901-88340412 | 9.40856 | 1.41268 |
| lncRNA05201 | "u" | chr01:70651688-70654035 | 0.830552 | 0.307838 |
| lncRNA20617 | "u" | chr05:20729509-20734443 | 1.62294 | 2.80595 |
| lncRNA31998 | "u" | chr08:61678990-61679411 | 2.57561 | 9.49494 |
| lncRNA19439 | "u" | chr04:58786444-58787449 | 1.94152 | 0.606023 |
| lncRNA11560 | "u" | chr03:984158-987419 | 0.960417 | 0.415139 |
| lncRNA33611 | "x" | chr08:60343824-60344745 | 4.57398 | 20.0791 |
| lncRNA22598 | "x" | chr05:52917390-52924690 | 1.12466 | 0.42477 |
| lncRNA43259 | "u" | chr11:4612052-4614192 | 2.73282 | 5.00973 |
| lncRNA47324 | "u" | chr12:28178255-28180956 | 0.651018 | 0.217567 |
| lncRNA31297 | "x" | chr08:51400701-51401412 | 171.34 | 279.147 |
| lncRNA21918 | "x" | chr05:5491137-5495631 | 0.958208 | 2.12247 |
| lncRNA23649 | "x" | chr06:21356929-21358920 | 4.96281 | 12.097 |
| lncRNA20113 | "x" | chr05:2627533-2628438 | 0.998136 | 3.36821 |
| lncRNA26095 | "x" | chr06:35526430-35531314 | 1.5782 | 0 |
| lncRNA16020 | "u" | chr03:64393452-64393822 | 3.10904 | 14.1488 |
| lncRNA28522 | "x" | chr07:62979494-62988936 | 2.07337 | 7.6898 |
| lncRNA23927 | "u" | chr06:31783847-31785025 | 0.235311 | 1.06773 |
| lncRNA32186 | "x" | chr08:477230-479985 | 7.19991 | 30.1844 |
| lncRNA01816 | "u" | chr01:41726909-41729595 | 1.04006 | 0.0661341 |
| lncRNA18649 | "u" | chr04:25307516-25311601 | 0.842893 | 0.0341891 |
| lncRNA20671 | "u" | chr05:27100491-27102707 | 0.86829 | 0.0660446 |
| lncRNA25502 | "u" | chr06:13174191-13176877 | 0.675655 | 0.0496378 |
| lncRNA41642 | "u" | chr11:8378626-8379292 | 4.02687 | 0.3637 |
| lncRNA15317 | "x" | chr03:56820716-56822367 | 11.5104 | 35.5039 |
| lncRNA41645 | "x" | chr11:8421678-8423170 | 5.60402 | 14.138 |
| lncRNA01355 | "u" | chr01:3276331-3277204 | 5.29957 | 10.188 |
| lncRNA20704 | "u" | chr05:28337606-28338339 | 3.36679 | 0.349516 |
| lncRNA32575 | "u" | chr08:15756780-15758930 | 1.16429 | 0.466227 |
| lncRNA19529 | "x" | chr04:60169704-60170500 | 6.56919 | 14.3808 |
| lncRNA11333 | "j" | chr02:48380826-48386599 | 3.89627 | 9.71262E-05 |
| lncRNA04361 | "u" | chr01:6654573-6655502 | 1.35869 | 3.41524 |
| lncRNA03791 | "x" | chr01:88141507-88144096 | 1.90661 | 4.20594 |
| lncRNA31265 | "x" | chr08:50647220-50651909 | 0.25511 | 1.78731 |
| lncRNA28956 | "u" | chr07:2407669-2408345 | 4.95236 | 1.89591 |
| lncRNA43811 | "x" | chr11:32178557-32186986 | 3.94493 | 2.31379 |
| lncRNA24759 | "x" | chr06:41978252-41979234 | 0.791399 | 4.1527 |
| lncRNA25748 | "u" | chr06:28420608-28421371 | 2.56225 | 0.329596 |
| lncRNA00806 | "u" | chr00:13811669-13812661 | 0.303436 | 1.28436 |
| lncRNA09245 | "u" | chr02:12514602-12515887 | 0.208719 | 0.884283 |
| lncRNA21803 | "x" | chr05:3661820-3663363 | 5.22287 | 11.7312 |
| lncRNA23994 | "x" | chr06:32954971-32960911 | 0.635564 | 3.69875 |
| lncRNA43355 | "x" | chr11:5550817-5554345 | 1.90451 | 3.46884 |
| lncRNA42896 | "x" | chr11:809686-816308 | 8.90038 | 19.1683 |
| lncRNA34906 | "u" | chr09:58561812-58563476 | 0.268427 | 0.885488 |
| lncRNA27826 | "u" | chr07:51471122-51472534 | 1.15804 | 0.375665 |
| lncRNA16430 | "x" | chr04:4642330-4650161 | 10.983 | 3.32961 |
| lncRNA15340 | "u" | chr03:57080126-57081096 | 1.73246 | 0.236297 |
| lncRNA28085 | "x" | chr07:57174888-57179154 | 0.190549 | 1.23775 |
| lncRNA17337 | "u" | chr04:56988059-56988782 | 0.416677 | 2.36988 |
| lncRNA45719 | "u" | chr12:43221012-43222439 | 0.142859 | 0.808651 |
| lncRNA44883 | "x" | chr12:2208960-2213073 | 13.3591 | 1.74195 |
| lncRNA01820 | "u" | chr01:41740063-41742631 | 0.710667 | 0.102681 |
| lncRNA07432 | "x" | chr02:31286097-31288095 | 2.81748 | 9.63664 |
| lncRNA45319 | "x" | chr12:11179365-11181852 | 7.62758 | 12.0818 |
| lncRNA17943 | "x" | chr04:272480-276895 | 0.512726 | 1.33288 |
| lncRNA01818 | "u" | chr01:41732321-41733496 | 0.892289 | 0.135532 |
| lncRNA40659 | "x" | chr10:62125510-62134770 | 8.37129 | 19.3691 |
| lncRNA24826 | "u" | chr06:42742275-42743823 | 2.3886 | 0.761764 |
| lncRNA47615 | "u" | chr12:43592513-43595101 | 1.80071 | 0.926847 |
| lncRNA47256 | "x" | chr12:17501785-17505067 | 4.31332 | 2.57518 |
| lncRNA07188 | "u" | chr02:21494787-21495086 | 11.1883 | 44.0433 |
| lncRNA17055 | "u" | chr04:51473199-51473778 | 1.01986 | 3.98612 |
| lncRNA27642 | "u" | chr07:30164421-30166858 | 5.54137 | 3.30351 |
| lncRNA34536 | "u" | chr09:23992561-23993634 | 1.8634 | 4.01648 |
| lncRNA29668 | "o" | chr07:55642717-55645751 | 5.43943 | 9.73936 |
| lncRNA29458 | "u" | chr07:45106435-45108574 | 0.770745 | 1.65084 |
| lncRNA17681 | "u" | chr04:61365864-61367337 | 0.472848 | 5.11291 |
| lncRNA05141 | "u" | chr01:69559300-69560319 | 0.207584 | 1.0796 |
| lncRNA29401 | "u" | chr07:38880628-38881695 | 0.220208 | 1.13683 |
| lncRNA43858 | "u" | chr11:35150296-35151620 | 0.801856 | 0.129232 |
| lncRNA07592 | "u" | chr02:33389832-33390893 | 1.07148 | 0.173506 |
| lncRNA15944 | "x" | chr03:63527013-63528365 | 7.70439 | 19.6272 |
| lncRNA40388 | "x" | chr10:59685255-59685740 | 214.576 | 136.69 |
| lncRNA30173 | "u" | chr07:62299418-62299670 | 80.5658 | 199.725 |
| lncRNA24239 | "x" | chr06:36344390-36356737 | 1.02401 | 2.60544 |
| lncRNA26429 | "x" | chr06:39258750-39259562 | 4.17624 | 1.51076 |
| lncRNA13239 | "x" | chr03:59978698-59982625 | 1.08636 | 4.06882 |
| lncRNA20710 | "u" | chr05:28878196-28880268 | 0.363562 | 0.953218 |
| lncRNA31156 | "u" | chr08:47326466-47334218 | 1.11465 | 0.642941 |
| lncRNA16397 | "x" | chr04:4279626-4280350 | 10.0933 | 19.3969 |
| lncRNA10266 | "x" | chr02:37193264-37198255 | 15.2141 | 24.4877 |
| lncRNA34732 | "x" | chr09:49974776-49979914 | 1.73022 | 0.96921 |
| lncRNA00334 | "x" | chr00:15479918-15483920 | 1.39727 | 0.695581 |
| lncRNA08897 | "x" | chr02:47797962-47802883 | 0.65964 | 4.21782 |
| lncRNA42193 | "x" | chr11:43070048-43072175 | 19.2265 | 29.4289 |
| lncRNA18677 | "x" | chr04:29682329-29684886 | 0.784461 | 0.316877 |
| lncRNA12165 | "u" | chr03:23390988-23393770 | 3.02128 | 5.04585 |
| lncRNA43795 | "x" | chr11:30306488-30316727 | 1.65655 | 0.278234 |
| lncRNA08852 | "x" | chr02:47263794-47264479 | 3.92891 | 8.82387 |
| lncRNA47733 | "x" | chr12:46405839-46409405 | 0.602837 | 4.04249 |
| lncRNA08638 | "x" | chr02:45062927-45065725 | 7.51887 | 3.40445 |
| lncRNA33375 | "x" | chr08:57365338-57366456 | 3.96223 | 0.48839 |
| lncRNA40295 | "x" | chr10:58725352-58729236 | 5.90253 | 20.6488 |
| lncRNA22188 | "x" | chr05:12393279-12397051 | 0.828153 | 2.77017 |
| lncRNA10215 | "x" | chr02:36490929-36491721 | 3.67336 | 0 |
| lncRNA20774 | "u" | chr05:34159065-34161109 | 1.90171 | 0.948286 |
| lncRNA01136 | "x" | chr01:581490-584457 | 27.8237 | 79.1535 |
| lncRNA35372 | "u" | chr09:65644691-65646202 | 0.264699 | 0.861303 |
| lncRNA05140 | "u" | chr01:69556262-69557540 | 0.261142 | 0.953408 |
| lncRNA13342 | "u" | chr03:61055715-61055940 | 532.604 | 242.573 |
| lncRNA18015 | "x" | chr04:1035525-1040396 | 3.68294 | 11.3583 |
| lncRNA01219 | "u" | chr01:1847821-1849243 | 3.06686 | 5.54352 |
| lncRNA20680 | "u" | chr05:28028228-28029454 | 0.646099 | 1.68182 |
| lncRNA48339 | "x" | chr12:65047726-65048317 | 0.56953 | 2.7455 |
| lncRNA22512 | "u" | chr05:41213618-41214509 | 0.536382 | 1.73621 |
| lncRNA40067 | "u" | chr10:50699890-50700604 | 0.797974 | 2.58783 |
| lncRNA43861 | "u" | chr11:35157085-35158374 | 1.08652 | 0.351837 |
| lncRNA18653 | "u" | chr04:25325014-25327994 | 0.596399 | 0.178949 |
| lncRNA29160 | "u" | chr07:8776731-8777626 | 1.29198 | 0.238643 |
| lncRNA45133 | "u" | chr12:4851554-4851756 | 406.144 | 75.0557 |
| lncRNA15600 | "x" | chr03:59874229-59877786 | 0.320127 | 4.16481 |
| lncRNA45270 | "u" | chr12:8796932-8797914 | 0.967686 | 0.179218 |
| lncRNA43874 | "u" | chr11:35604661-35605693 | 0.886028 | 2.22693 |
| lncRNA46646 | "x" | chr12:1197743-1198316 | 3.32089 | 7.91813 |
| lncRNA34190 | "u" | chr09:4149915-4151584 | 0.655408 | 1.50134 |
| lncRNA29380 | "u" | chr07:35881043-35881496 | 9.36819 | 3.41498 |
| lncRNA18309 | "x" | chr04:4802047-4802851 | 0 | 30.8021 |
| lncRNA30851 | "u" | chr08:10049855-10051476 | 2.62687 | 5.06925 |
| lncRNA06104 | "x" | chr01:82372616-82378174 | 2.227 | 0.723418 |
| lncRNA40765 | "x" | chr10:63070080-63070556 | 6.06215 | 1.37873 |
| lncRNA23882 | "x" | chr06:30774363-30780559 | 1.72562 | 0.247398 |
| lncRNA16918 | "x" | chr04:44843587-44849727 | 0.338447 | 1.55506 |
| lncRNA01963 | "u" | chr01:55999327-56000578 | 1.83384 | 0.744407 |
| lncRNA46344 | "u" | chr12:64145039-64145316 | 246.71 | 129.202 |
| lncRNA09226 | "x" | chr02:11048765-11050013 | 1.03856 | 2.3681 |
| lncRNA36476 | "u" | chr09:50294502-50297747 | 0.454079 | 2.57907 |
| lncRNA10701 | "x" | chr02:41979042-42002325 | 1.0053 | 0.390286 |
| lncRNA00448 | "u" | chr00:18469011-18469830 | 0.820982 | 2.42221 |
| lncRNA42184 | "x" | chr11:42531841-42533493 | 0.0799327 | 1.04586 |
| lncRNA14808 | "x" | chr03:46396641-46403381 | 0.621003 | 2.13995 |
| lncRNA00502 | "u" | chr00:21014186-21014624 | 14.9899 | 6.41818 |
| lncRNA37976 | "x" | chr10:36273150-36274835 | 17.2193 | 6.72203 |
| lncRNA44733 | "x" | chr12:756222-761422 | 0 | 2.08366 |
| lncRNA03757 | "x" | chr01:87837114-87862936 | 2.45259 | 4.2787 |
| lncRNA05543 | "u" | chr01:75683989-75686506 | 1.04181 | 0.471171 |
| lncRNA00957 | "u" | chr00:17182880-17183391 | 0.812757 | 3.8607 |
| lncRNA26804 | "x" | chr06:43485409-43486259 | 11.8999 | 45.6914 |
| lncRNA43497 | "u" | chr11:9129401-9131547 | 0.849224 | 1.72604 |
| lncRNA41037 | "x" | chr11:572116-573019 | 0 | 6.09195 |
| lncRNA42357 | "u" | chr11:48136023-48143512 | 0 | 2.1196 |
| lncRNA19416 | "x" | chr04:58430493-58435380 | 0.0679693 | 3.25937 |
| lncRNA36021 | "u" | chr09:6790158-6791124 | 0.504434 | 1.75469 |
| lncRNA35571 | "x" | chr09:21503-22460 | 0 | 4.55475 |
| lncRNA08991 | "u" | chr02:48739667-48740001 | 2.42974 | 25.426 |
| lncRNA34353 | "u" | chr09:8902079-8902959 | 0.184672 | 1.93448 |
| lncRNA37696 | "u" | chr10:8880372-8881048 | 0.310393 | 3.22788 |
| lncRNA31787 | "u" | chr08:59332018-59332874 | 0.176331 | 1.95779 |
| lncRNA32803 | "u" | chr08:42000185-42000646 | 0.666496 | 7.79867 |
| lncRNA04352 | "u" | chr01:6324078-6324725 | 0.305885 | 2.99028 |
| lncRNA36378 | "u" | chr09:43526228-43527034 | 0.646618 | 1.95802 |
| lncRNA43838 | "u" | chr11:34453242-34453755 | 1.58309 | 4.80676 |
| lncRNA16450 | "u" | chr04:4959990-4960843 | 1.02117 | 2.66556 |
| lncRNA39244 | "x" | chr10:1717851-1718736 | 2.67368 | 0 |
| lncRNA08344 | "u" | chr02:41829797-41904084 | 0.313051 | 4.07903 |
| lncRNA15608 | "x" | chr03:59941067-59942433 | 3.52837 | 0 |
| lncRNA48297 | "u" | chr12:64702201-64702488 | 62.5082 | 23.4263 |
| lncRNA42112 | "u" | chr11:36386485-36389536 | 2.50071 | 1.45081 |
| lncRNA04551 | "u" | chr01:22234725-22236384 | 0.49868 | 1.23726 |
| lncRNA10222 | "x" | chr02:36695970-36697471 | 7.83831 | 31.969 |
| lncRNA30062 | "x" | chr07:60919658-60927117 | 8.62364 | 16.8226 |
| lncRNA17969 | "x" | chr04:444511-446828 | 5.21819 | 15.2834 |
| lncRNA09828 | "x" | chr02:32274269-32278858 | 0.602347 | 1.84224 |
| lncRNA39299 | "u" | chr10:2338858-2339607 | 4.67176 | 2.02838 |
| lncRNA20527 | "u" | chr05:11825815-11830112 | 0.667476 | 0.325511 |
| lncRNA12037 | "u" | chr03:14312830-14313130 | 36.6933 | 11.0382 |
| lncRNA21968 | "x" | chr05:6125778-6127530 | 1.8703 | 4.89741 |
| lncRNA48279 | "x" | chr12:64612489-64619396 | 0.615506 | 3.5498 |
| lncRNA23836 | "x" | chr06:29702610-29704227 | 0.686937 | 2.16441 |
| lncRNA40240 | "x" | chr10:57697258-57698119 | 12.5679 | 43.171 |
| lncRNA07241 | "u" | chr02:25673793-25674698 | 1.46564 | 0.448122 |
| lncRNA09412 | "x" | chr02:19610721-19612268 | 1.22732 | 0.229748 |
| lncRNA44573 | "x" | chr11:52972751-52973800 | 0 | 1.06714 |
| lncRNA47566 | "x" | chr12:42592137-42594528 | 1.58791 | 3.46661 |
| lncRNA12789 | "x" | chr03:53316438-53319652 | 5.6811 | 12.8143 |
| lncRNA41915 | "x" | chr11:21627535-21638836 | 1.78014 | 3.55506 |
| lncRNA44938 | "x" | chr12:2768265-2794422 | 0 | 1.35343 |
| lncRNA03806 | "x" | chr01:88361511-88364324 | 0.294437 | 3.56419 |
| lncRNA41941 | "u" | chr11:24820303-24826896 | 0.590466 | 2.61357 |
| lncRNA02005 | "u" | chr01:58873954-58874397 | 0.724844 | 5.28437 |
| lncRNA26370 | "u" | chr06:38674046-38674374 | 230.571 | 135.985 |
| lncRNA32516 | "u" | chr08:7485990-7486732 | 0.272506 | 1.94564 |
| lncRNA47954 | "u" | chr12:57432601-57432912 | 5.3482 | 23.2947 |
| lncRNA42957 | "x" | chr11:1335464-1337916 | 0.629058 | 1.6664 |
| lncRNA23574 | "u" | chr06:16269576-16270602 | 4.96233 | 2.61908 |
| lncRNA24841 | "u" | chr06:42903684-42905376 | 1.51219 | 2.85356 |
| lncRNA07558 | "x" | chr02:32947998-32965109 | 0.942367 | 7.14942 |
| lncRNA04480 | "u" | chr01:14793048-14793987 | 0.252889 | 1.09491 |
| lncRNA23837 | "u" | chr06:29730753-29731765 | 0.226201 | 0.977287 |
| lncRNA27130 | "u" | chr07:920496-920788 | 7.11535 | 30.7828 |
| lncRNA34455 | "u" | chr09:16980860-16981991 | 0.193331 | 0.830931 |
| lncRNA39819 | "u" | chr10:37134194-37136102 | 3.08605 | 5.14319 |
| lncRNA36171 | "u" | chr09:18654280-18654966 | 5.99898 | 11.0044 |
| lncRNA32838 | "x" | chr08:45098463-45100390 | 0.944649 | 2.3143 |
| lncRNA43796 | "x" | chr11:30306488-30316727 | 0.54261 | 1.80275 |
| lncRNA10677 | "x" | chr02:41715235-41716114 | 0.897719 | 4.27664 |
| lncRNA33566 | "x" | chr08:59728468-59729171 | 21.2712 | 49.0683 |
| lncRNA47633 | "u" | chr12:43986948-43995236 | 3.70715 | 1.16494 |
| lncRNA14962 | "x" | chr03:49508931-49512210 | 0.739549 | 2.64766 |
| lncRNA36185 | "u" | chr09:20109084-20110149 | 1.56668 | 3.3151 |
| lncRNA01743 | "u" | chr01:32673860-32675784 | 3.9808 | 6.49169 |
| lncRNA21432 | "x" | chr05:64016156-64016880 | 0.381778 | 1.66014 |
| lncRNA35879 | "x" | chr09:3999003-4000757 | 6.51748 | 0 |
| lncRNA39859 | "u" | chr10:41243919-41245448 | 1.42638 | 0.598084 |
| lncRNA34370 | "x" | chr09:10704858-10705739 | 0.451619 | 1.42114 |
| lncRNA24336 | "x" | chr06:37639580-37641049 | 7.65101 | 2.32547 |
| lncRNA23441 | "u" | chr06:4231659-4232231 | 0.419453 | 2.71264 |
| lncRNA40932 | "u" | chr10:64496380-64497037 | 0.302542 | 1.9557 |
| lncRNA03399 | "u" | chr01:84328597-84329138 | 10.7995 | 19.7415 |
| lncRNA25667 | "u" | chr06:23134876-23137661 | 0.87257 | 0.410669 |
| lncRNA36953 | "x" | chr09:63560069-63562823 | 0.323341 | 0.725927 |
| lncRNA11973 | "u" | chr03:11109438-11109937 | 2.24581 | 6.66533 |
| lncRNA20093 | "u" | chr05:2357689-2358723 | 8.77477 | 31.0052 |
| lncRNA21008 | "u" | chr05:56284702-56288492 | 0.423939 | 0.837717 |
| lncRNA41534 | "u" | chr11:6062758-6063062 | 29.6814 | 8.1801 |
| lncRNA07593 | "u" | chr02:33392147-33394606 | 2.78301 | 1.63602 |
| lncRNA20480 | "u" | chr05:9434712-9443226 | 2.12471 | 1.37985 |
| lncRNA09913 | "x" | chr02:33195651-33199149 | 0.192973 | 1.37884 |
| lncRNA40138 | "u" | chr10:52262055-52262329 | 135.6 | 60.504 |
| lncRNA17155 | "u" | chr04:54098473-54099524 | 0.954581 | 0.266074 |
| lncRNA33179 | "x" | chr08:54692199-54698140 | 1.48682 | 0.306157 |
| lncRNA35745 | "u" | chr09:2042501-2043424 | 0.974458 | 0.211721 |
| lncRNA20183 | "x" | chr05:3477307-3482473 | 1.73486 | 3.26704 |
| lncRNA18403 | "u" | chr04:6172588-6173509 | 4.57238 | 2.19598 |
| lncRNA01786 | "x" | chr01:38354906-38357065 | 0.264722 | 0.722586 |
| lncRNA14474 | "x" | chr03:25619127-25621392 | 3.20165 | 1.44042 |
| lncRNA22016 | "u" | chr05:6773602-6773952 | 11.6106 | 28.3919 |
| lncRNA13352 | "x" | chr03:61188205-61198586 | 6.41272 | 14.8922 |
| lncRNA11197 | "x" | chr02:46994697-46995319 | 1.84254 | 4.9897 |
| lncRNA23292 | "u" | chr06:1669863-1671165 | 0.822475 | 0.265875 |
| lncRNA41676 | "x" | chr11:9597158-9598442 | 0 | 1.3961 |
| lncRNA06088 | "u" | chr01:82239126-82239972 | 1.59983 | 0.517812 |
| lncRNA41968 | "x" | chr11:28095241-28097066 | 7.68505 | 20.0046 |
| lncRNA35979 | "x" | chr09:5596206-5599498 | 4.14074 | 1.84256 |
| lncRNA10567 | "x" | chr02:40551326-40557705 | 11.3421 | 19.1846 |
| lncRNA27872 | "x" | chr07:53217866-53220281 | 0.242306 | 4.31043 |
| lncRNA20555 | "u" | chr05:14562072-14563058 | 0.551772 | 1.54625 |
| lncRNA43573 | "x" | chr11:12930247-12934220 | 2.32657 | 3.60535 |
| lncRNA10985 | "x" | chr02:44897965-44905576 | 0.421642 | 3.17007 |
| lncRNA32402 | "u" | chr08:3122627-3124192 | 2.16378 | 3.82955 |
| lncRNA39883 | "u" | chr10:41894446-41894912 | 1.69528 | 5.40293 |
| lncRNA27593 | "x" | chr07:23736736-23739475 | 2.33828 | 0.242389 |
| lncRNA30111 | "x" | chr07:61460391-61463238 | 2.84002 | 0 |
| lncRNA11329 | "x" | chr02:48339963-48342215 | 5.40298 | 16.7245 |
| lncRNA37133 | "x" | chr09:65837796-65842237 | 3.45925 | 5.55105 |
| lncRNA10359 | "x" | chr02:38213824-38215181 | 1.13701 | 7.64012 |
| lncRNA27896 | "x" | chr07:53663086-53682449 | 1.19583 | 0.255375 |
| lncRNA38370 | "x" | chr10:57382489-57385238 | 1.47558 | 0 |
| lncRNA11165 | "u" | chr02:46662311-46663299 | 12.8133 | 7.87829 |
| lncRNA31345 | "u" | chr08:52421438-52421865 | 9.60189 | 3.24345 |
| lncRNA43944 | "u" | chr11:37535103-37535513 | 10.0973 | 3.41441 |
| lncRNA43071 | "u" | chr11:2399564-2400846 | 0.274392 | 0.85958 |
| lncRNA27592 | "x" | chr07:23736736-23739475 | 5.0233 | 0.482672 |
| lncRNA07816 | "u" | chr02:36203556-36204253 | 2.82759 | 0.970368 |
| lncRNA09103 | "u" | chr02:586278-587168 | 0.184242 | 1.0775 |
| lncRNA44099 | "x" | chr11:46722580-46724036 | 0.293907 | 2.31511 |
| lncRNA47264 | "x" | chr12:18330731-18333243 | 3.77693 | 5.99592 |
| lncRNA39158 | "x" | chr10:879558-881917 | 1.32385 | 0 |
| lncRNA27344 | "u" | chr07:4252473-4252804 | 6.80079 | 20.9961 |
| lncRNA04867 | "x" | chr01:57853718-57859489 | 4.42304 | 9.4771 |
| lncRNA01281 | "x" | chr01:2351731-2354922 | 0.235258 | 1.12389 |
| lncRNA38632 | "x" | chr10:60699095-60701253 | 2.08661 | 3.52534 |
| lncRNA03151 | "x" | chr01:81501270-81502283 | 0 | 16.6693 |
| lncRNA09750 | "u" | chr02:31192201-31192691 | 5.4919 | 1.91877 |
| lncRNA33116 | "u" | chr08:53738034-53738621 | 2.68613 | 6.02328 |
| lncRNA47634 | "u" | chr12:43986948-43995236 | 2.12753 | 0 |
| lncRNA40942 | "x" | chr10:64546760-64548729 | 0.61403 | 0 |
| lncRNA36911 | "u" | chr09:63005359-63005692 | 14.9257 | 3.4904 |
| lncRNA32168 | "x" | chr08:246372-250280 | 0 | 1.70631 |
| lncRNA06163 | "x" | chr01:83039082-83049084 | 0.963471 | 2.49187 |
| lncRNA17406 | "x" | chr04:58024809-58025206 | 8.13257 | 120.944 |
| lncRNA25963 | "x" | chr06:33451917-33459215 | 1.16372 | 0.614133 |
| lncRNA01460 | "u" | chr01:6450515-6451335 | 1.17693 | 0.279321 |
| lncRNA21701 | "u" | chr05:2265656-2266279 | 2.06197 | 0.489192 |
| lncRNA41999 | "x" | chr11:30677308-30678765 | 53.5826 | 86.4863 |
| lncRNA15422 | "x" | chr03:58090160-58093921 | 2.76903 | 15.0584 |
| lncRNA13784 | "x" | chr03:924277-925035 | 0 | 9.91758 |
| lncRNA09688 | "x" | chr02:30136823-30137671 | 2.9239 | 0 |
| lncRNA46307 | "x" | chr12:63841272-63846257 | 33.1422 | 14.8716 |
| lncRNA09783 | "x" | chr02:31761821-31764561 | 0 | 7.46336 |
| lncRNA13882 | "x" | chr03:2447871-2450289 | 0 | 1.01315 |
| lncRNA35132 | "x" | chr09:62492454-62493246 | 7.42697 | 20.959 |
| lncRNA15885 | "x" | chr03:62953174-62958396 | 0 | 2.12908 |
| lncRNA28109 | "x" | chr07:57815207-57818076 | 0 | 1.47364 |
| lncRNA10501 | "x" | chr02:39818419-39819293 | 0 | 0.654112 |
| lncRNA45747 | "x" | chr12:43805595-43811395 | 2.96321 | 0.982659 |
| lncRNA20974 | "x" | chr05:54715410-54716835 | 1.27428 | 2.46013 |
| lncRNA30857 | "u" | chr08:10174510-10176474 | 0.536062 | 1.13885 |
| lncRNA02524 | "u" | chr01:74049079-74052597 | 0.80135 | 0.409527 |
| lncRNA26803 | "x" | chr06:43485409-43486259 | 7.27625 | 41.058 |
| lncRNA00913 | "o" | chr00:16398061-16401013 | 1.27837 | 2.4813 |
| lncRNA08227 | "x" | chr02:40810611-40812065 | 7.64105 | 11.8024 |
| lncRNA12464 | "x" | chr03:46007490-46008009 | 4.61117 | 48.4272 |
| lncRNA18540 | "u" | chr04:17037950-17038690 | 0.996938 | 2.61493 |
| lncRNA11158 | "x" | chr02:46616046-46620783 | 0.884851 | 1.51164 |
| lncRNA03945 | "x" | chr01:89940349-89944973 | 1.14409 | 5.15292 |
| lncRNA06137 | "x" | chr01:82700182-82706600 | 1.19784 | 4.36925 |
| lncRNA26651 | "u" | chr06:41791301-41794050 | 0.874185 | 0.416053 |
| lncRNA15476 | "u" | chr03:58655625-58656330 | 0.268733 | 1.39642 |
| lncRNA30237 | "x" | chr07:62950635-62951305 | 0 | 2.21292 |
| lncRNA04228 | "x" | chr01:3003152-3007792 | 0 | 1.40011 |
| lncRNA18603 | "x" | chr04:22253859-22258420 | 0.654871 | 1.12728 |
| lncRNA26579 | "x" | chr06:41085509-41086879 | 4.98022 | 27.3111 |
| lncRNA41405 | "u" | chr11:4558023-4558583 | 3.89652 | 8.23769 |
| lncRNA02021 | "x" | chr01:59598120-59600281 | 10.1069 | 14.9291 |
| lncRNA14260 | "x" | chr03:11829270-11833871 | 3.07666 | 0.611696 |
| lncRNA14272 | "u" | chr03:12362335-12364978 | 2.00203 | 1.15234 |
| lncRNA14889 | "x" | chr03:47847207-47852705 | 0 | 0.809418 |
| lncRNA21958 | "x" | chr05:5972392-5973726 | 3.30203 | 11.4213 |
| lncRNA43139 | "x" | chr11:3151022-3153505 | 0.907201 | 0.2623 |
| lncRNA10163 | "u" | chr02:35972148-35973538 | 0.842704 | 0.302344 |
| lncRNA30269 | "x" | chr07:63268541-63271127 | 0.403456 | 2.02213 |
| lncRNA48277 | "x" | chr12:64603645-64605645 | 0 | 0.878306 |
| lncRNA18793 | "u" | chr04:38930115-38933297 | 1.84038 | 0.553765 |
| lncRNA41875 | "u" | chr11:18020242-18021072 | 1.28868 | 0.387488 |
| lncRNA01102 | "x" | chr01:314641-317493 | 0 | 0.713799 |
| lncRNA25621 | "x" | chr06:21742746-21745614 | 1.89626 | 0.713335 |
| lncRNA17446 | "u" | chr04:58540625-58542091 | 0.684259 | 1.47833 |
| lncRNA46472 | "x" | chr12:65165431-65167889 | 88.9476 | 0 |
| lncRNA09105 | "u" | chr02:592167-593127 | 0.313492 | 1.01057 |
| lncRNA16603 | "u" | chr04:8943242-8943951 | 0.505048 | 1.64071 |
| lncRNA37501 | "u" | chr10:2723766-2724019 | 25.7498 | 83.6535 |
| lncRNA27631 | "u" | chr07:28910733-28912217 | 0.89295 | 0.347154 |
| lncRNA16379 | "x" | chr04:4102743-4104141 | 29.7717 | 18.7882 |
| lncRNA40638 | "x" | chr10:61954419-61960495 | 3.66621 | 0.100086 |
| lncRNA14017 | "u" | chr03:7749678-7750988 | 2.73604 | 1.44882 |
| lncRNA45524 | "x" | chr12:32459033-32460513 | 2.17961 | 0.0661638 |
| lncRNA15025 | "x" | chr03:51500938-51502966 | 2.35309 | 0.0708227 |
| lncRNA01945 | "x" | chr01:53762259-53768904 | 1.63824 | 0.534331 |
| lncRNA13669 | "x" | chr03:64506157-64507554 | 0 | 0.704836 |
| lncRNA12764 | "x" | chr03:52943950-52951087 | 0.939587 | 2.31367 |
| lncRNA38824 | "x" | chr10:62423532-62424630 | 1.78207 | 3.40914 |
| lncRNA32401 | "u" | chr08:3121701-3121986 | 16.0213 | 45.4289 |
| lncRNA40888 | "x" | chr10:64131897-64136617 | 1.05399 | 2.76654 |
| lncRNA03465 | "x" | chr01:84884014-84885380 | 2.35543 | 0.305604 |
| lncRNA30799 | "u" | chr08:6557885-6558856 | 5.75746 | 2.09827 |
| lncRNA39367 | "x" | chr10:3201654-3211010 | 1.43954 | 4.53493 |
| lncRNA19488 | "x" | chr04:59423287-59430627 | 0.60124 | 1.36727 |
| lncRNA00139 | "o" | chr00:11067019-11074070 | 0.0378298 | 0.793048 |
| lncRNA06838 | "x" | chr02:521767-522744 | 0.977429 | 2.20625 |
| lncRNA26955 | "x" | chr06:44977537-44982219 | 3.34469 | 5.80415 |
| lncRNA14915 | "x" | chr03:48476778-48477850 | 6.30358 | 17.3126 |
| lncRNA44094 | "x" | chr11:46703642-46704333 | 14.0198 | 22.4878 |
| lncRNA40637 | "u" | chr10:61953534-61954274 | 18.0656 | 0.163931 |
| lncRNA43860 | "u" | chr11:35155373-35156976 | 1.17362 | 0.050583 |
| lncRNA45269 | "u" | chr12:8794143-8795671 | 1.31673 | 0.053798 |
| lncRNA02002 | "x" | chr01:58770101-58774780 | 4.63312 | 11.8091 |
| lncRNA48015 | "x" | chr12:62213767-62215934 | 0.0642769 | 1.91879 |
| lncRNA09629 | "u" | chr02:29070507-29071909 | 0.27701 | 0.776651 |
| lncRNA16519 | "u" | chr04:6139675-6140249 | 4.75305 | 0.268757 |
| lncRNA20672 | "u" | chr05:27104259-27105642 | 1.15548 | 0.0622057 |
| lncRNA00407 | "u" | chr00:16863242-16863848 | 3.72126 | 0.253764 |
| lncRNA27630 | "u" | chr07:28901620-28910364 | 1.43171 | 0.932446 |
| lncRNA04706 | "u" | chr01:38333509-38334113 | 1.65729 | 3.97138 |
| lncRNA07598 | "u" | chr02:33585416-33586119 | 2.59675 | 0.188025 |
| lncRNA20600 | "u" | chr05:19938233-19939281 | 1.2132 | 0.0873582 |
| lncRNA37539 | "x" | chr10:3115286-3120260 | 1.56784 | 2.42603 |
| lncRNA12731 | "x" | chr03:52534418-52537953 | 0.625524 | 1.50619 |
| lncRNA37223 | "x" | chr09:67011460-67019762 | 1.83211 | 4.62668 |
| lncRNA19070 | "x" | chr04:53005643-53010973 | 5.86136 | 4.03378 |
| lncRNA30655 | "x" | chr08:2338721-2339698 | 0.45063 | 24.1226 |
| lncRNA02918 | "x" | chr01:79051799-79061053 | 13.6562 | 42.2859 |
| lncRNA22691 | "u" | chr05:57619541-57622045 | 2.30169 | 0.978095 |
| lncRNA45043 | "x" | chr12:3710824-3713679 | 3.75076 | 7.99728 |
| lncRNA11925 | "x" | chr03:10049495-10050240 | 28.6043 | 47.3068 |
| lncRNA04693 | "u" | chr01:37068447-37069419 | 1.30681 | 0.105544 |
| lncRNA13261 | "x" | chr03:60295718-60304075 | 1.29887 | 0.633922 |
| lncRNA15418 | "x" | chr03:58055975-58059292 | 8.28525 | 15.3296 |
| lncRNA21044 | "u" | chr05:57484259-57485207 | 1.20746 | 0.111766 |
| lncRNA43656 | "u" | chr11:17303021-17304012 | 1.17146 | 0.108167 |
| lncRNA15024 | "x" | chr03:51490006-51491498 | 2.67181 | 0.0819496 |
| lncRNA25735 | "x" | chr06:28150997-28154553 | 1.06054 | 0.0370595 |
| lncRNA03444 | "u" | chr01:84659488-84660667 | 6.22513 | 2.87696 |
| lncRNA12286 | "u" | chr03:33074663-33074998 | 17.2137 | 1.7081 |
| lncRNA40010 | "u" | chr10:48254186-48254425 | 107.768 | 10.6999 |
| lncRNA29381 | "u" | chr07:35881779-35882283 | 3.72875 | 0.373791 |
| lncRNA44477 | "x" | chr11:52033914-52041685 | 1.83698 | 1.04787 |
| lncRNA35711 | "x" | chr09:1486828-1491959 | 0.722204 | 3.01062 |
| lncRNA23175 | "x" | chr05:64992744-65017054 | 1.56417 | 0.794991 |
| lncRNA43428 | "x" | chr11:7149074-7152323 | 1.7775 | 2.84416 |
| lncRNA22985 | "u" | chr05:63025867-63026391 | 2.29516 | 5.57698 |
| lncRNA02526 | "u" | chr01:74059546-74063464 | 0.819176 | 0.447145 |
| lncRNA04797 | "x" | chr01:52332878-52333746 | 0.118312 | 2.14798 |
| lncRNA45317 | "u" | chr12:11125740-11125956 | 1069.16 | 1814.77 |
| lncRNA47464 | "x" | chr12:36029921-36030558 | 3.71329 | 0.852223 |
| lncRNA20538 | "u" | chr05:12106622-12107462 | 2.57581 | 4.83927 |
| lncRNA04946 | "u" | chr01:62530405-62530992 | 2.21353 | 0.241528 |
| lncRNA06289 | "u" | chr01:84498933-84499334 | 23.9136 | 2.57496 |
| lncRNA22373 | "u" | chr05:26933249-26934248 | 0.906681 | 0.0977051 |
| lncRNA05892 | "x" | chr01:80108156-80110207 | 0.036356 | 3.02179 |
| lncRNA06898 | "x" | chr02:6858324-6860295 | 0.0317455 | 1.28001 |
| lncRNA02163 | "u" | chr01:67810867-67811148 | 32.95 | 3.66615 |
| lncRNA23941 | "x" | chr06:32148709-32156014 | 0.971745 | 2.2405 |
| lncRNA25962 | "x" | chr06:33448176-33448873 | 0.398931 | 1.7377 |
| lncRNA34188 | "u" | chr09:4145379-4145708 | 53.1004 | 27.122 |
| lncRNA39762 | "x" | chr10:32356988-32360552 | 0.705491 | 1.27115 |
| lncRNA22826 | "x" | chr05:60666716-60668481 | 21.7056 | 58.8673 |
| lncRNA10585 | "x" | chr02:40822196-40830729 | 1.1304 | 0.659398 |
| lncRNA45875 | "x" | chr12:47038541-47039699 | 0.47313 | 5.53981 |
| lncRNA08815 | "x" | chr02:46873795-46874616 | 0.21409 | 2.80169 |
| lncRNA25101 | "x" | chr06:45635412-45639918 | 0.496857 | 2.74103 |
| lncRNA18326 | "x" | chr04:5013005-5013501 | 0.28542 | 3.69337 |
| lncRNA16461 | "x" | chr04:5055497-5059899 | 34.5993 | 20.9508 |
| lncRNA21373 | "u" | chr05:63266743-63267116 | 9.0553 | 2.91045 |
| lncRNA05341 | "u" | chr01:72991148-72992039 | 1.11039 | 0.358034 |
| lncRNA34952 | "u" | chr09:59452819-59453271 | 8.25438 | 3.59089 |
| lncRNA08976 | "u" | chr02:48558520-48559013 | 3.50664 | 1.13919 |
| lncRNA24035 | "u" | chr06:33614105-33614497 | 4.43151 | 11.4427 |
| lncRNA28300 | "x" | chr07:60307299-60307878 | 0.393839 | 2.06706 |
| lncRNA33800 | "u" | chr08:62621783-62622693 | 1.1007 | 0.355987 |
| lncRNA36114 | "u" | chr09:13824930-13825442 | 3.13027 | 1.0283 |
| lncRNA41427 | "u" | chr11:4779851-4780735 | 1.28781 | 0.418016 |
| lncRNA45595 | "x" | chr12:36398372-36399129 | 5.25866 | 0.382356 |
| lncRNA12815 | "x" | chr03:53797570-53802108 | 0.181754 | 1.33407 |
| lncRNA10602 | "x" | chr02:40937857-40942241 | 1.58622 | 0.813408 |
| lncRNA35930 | "u" | chr09:4775809-4776120 | 3.60621 | 16.3125 |
| lncRNA45771 | "u" | chr12:44558248-44558913 | 0.276207 | 1.25923 |
| lncRNA17697 | "x" | chr04:61565222-61568797 | 14.4017 | 29.2159 |
| lncRNA07869 | "u" | chr02:36753976-36754911 | 0.914013 | 0.107594 |
| lncRNA18745 | "u" | chr04:33199383-33199873 | 3.2697 | 0.387874 |
| lncRNA33693 | "u" | chr08:61345920-61346720 | 1.25291 | 0.148035 |
| lncRNA36007 | "u" | chr09:6171338-6171850 | 3.03353 | 0.361665 |
| lncRNA42154 | "u" | chr11:37956212-37956723 | 3.10276 | 0.366829 |
| lncRNA02115 | "u" | chr01:65799850-65801549 | 0.036311 | 1.65825 |
| lncRNA28029 | "x" | chr07:56198166-56199708 | 1.45374 | 4.8846 |
| lncRNA31397 | "u" | chr08:53527785-53543779 | 1.05837 | 0.267904 |
| lncRNA44994 | "u" | chr12:3308963-3310147 | 0.0589148 | 1.9102 |
| lncRNA23491 | "u" | chr06:6994994-6996772 | 0.0374032 | 1.06606 |
| lncRNA23366 | "x" | chr06:2751016-2756245 | 2.79862 | 4.82322 |
| lncRNA07471 | "x" | chr02:31920281-31922757 | 7.32272 | 11.7069 |
| lncRNA08809 | "u" | chr02:46828206-46828907 | 0.142169 | 2.99278 |
| lncRNA31420 | "x" | chr08:53791950-53795119 | 1.23523 | 0.698473 |
| lncRNA15529 | "u" | chr03:59147276-59147805 | 0.22794 | 4.48324 |
| lncRNA29655 | "x" | chr07:55287345-55287839 | 21.9888 | 35.3471 |
| lncRNA04728 | "u" | chr01:41406688-41407944 | 0.0561733 | 0.8838 |
| lncRNA13837 | "x" | chr03:1782260-1784088 | 0.484287 | 1.55961 |
| lncRNA05898 | "x" | chr01:80165237-80167324 | 0.153578 | 2.57894 |
| lncRNA12332 | "u" | chr03:41128867-41129851 | 1.25888 | 0.477613 |
| lncRNA44572 | "x" | chr11:52967547-52972273 | 1.91039 | 5.4889 |
| lncRNA42120 | "u" | chr11:36555579-36560732 | 0.529034 | 0.893995 |
| lncRNA42389 | "u" | chr11:48612923-48614580 | 1.12888 | 0.465593 |
| lncRNA38292 | "x" | chr10:52128394-52129614 | 0.350986 | 0.997608 |
| lncRNA03483 | "u" | chr01:85041571-85041953 | 0.677956 | 8.82386 |
| lncRNA30576 | "u" | chr08:1267121-1267347 | 13.0423 | 169.618 |
| lncRNA33604 | "x" | chr08:60284205-60287329 | 0.2182 | 1.9719 |
| lncRNA40838 | "x" | chr10:63683477-63684479 | 8.81424 | 22.0054 |
| lncRNA42110 | "u" | chr11:36381723-36382349 | 0.168067 | 2.17857 |
| lncRNA18938 | "x" | chr04:48879772-48894993 | 0.0556374 | 1.29508 |
| lncRNA00819 | "x" | chr00:14089900-14092830 | 1.99455 | 0.703588 |
| lncRNA41407 | "u" | chr11:4563921-4564640 | 1.8806 | 0.654114 |
| lncRNA05869 | "x" | chr01:79885939-79899140 | 14.6149 | 7.80662 |
| lncRNA18378 | "u" | chr04:5715739-5716044 | 4.09991 | 18.4025 |
| lncRNA47306 | "x" | chr12:24407409-24424790 | 3.24726 | 7.41403 |
| lncRNA46874 | "u" | chr12:3606606-3607534 | 2.47026 | 1.14297 |
| lncRNA37784 | "u" | chr10:19363625-19364485 | 0.989252 | 0.128934 |
| lncRNA00513 | "u" | chr00:21692119-21692341 | 146.806 | 19.2253 |
| lncRNA15938 | "x" | chr03:63482882-63487287 | 1.03835 | 3.79591 |
| lncRNA23379 | "u" | chr06:2940632-2941194 | 2.07687 | 0.271593 |
| lncRNA30654 | "x" | chr08:2324975-2327685 | 0.228525 | 6.84598 |
| lncRNA47953 | "x" | chr12:57421692-57431892 | 0.365917 | 0.872517 |
| lncRNA42884 | "u" | chr11:682004-682530 | 0.228942 | 2.68727 |
| lncRNA10609 | "u" | chr02:40993301-40993931 | 4.73071 | 2.20014 |
| lncRNA25006 | "u" | chr06:44613691-44623029 | 21.9924 | 13.4191 |
| lncRNA39630 | "x" | chr10:18289417-18295394 | 2.01714 | 5.83954 |
| lncRNA21959 | "x" | chr05:5972392-5973726 | 3.50787 | 9.21067 |
| lncRNA32659 | "u" | chr08:25432124-25433698 | 0.63994 | 1.3288 |
| lncRNA46686 | "x" | chr12:1648824-1652511 | 1.62184 | 0.629929 |
| lncRNA13295 | "u" | chr03:60623298-60641696 | 0.193489 | 0.633576 |
| lncRNA00054 | "u" | chr00:6573799-6574418 | 0.176174 | 1.83292 |
| lncRNA31870 | "x" | chr08:60152857-60158284 | 2.2629 | 5.66173 |
| lncRNA09099 | "x" | chr02:339588-350689 | 0.13175 | 1.52801 |
| lncRNA45244 | "x" | chr12:6967964-6969107 | 0.625293 | 4.9037 |
| lncRNA41168 | "x" | chr11:1763622-1765452 | 0.838281 | 0.090427 |
| lncRNA12019 | "x" | chr03:13169314-13171872 | 0.338094 | 1.21353 |
| lncRNA19605 | "x" | chr04:60958459-60963013 | 4.48247 | 6.79952 |
| lncRNA12299 | "u" | chr03:34296315-34298691 | 0.406938 | 0.822658 |
| lncRNA17658 | "u" | chr04:61174421-61175848 | 3.20656 | 1.8573 |
| lncRNA24136 | "x" | chr06:35101220-35102882 | 30.4615 | 77.5789 |
| lncRNA29071 | "x" | chr07:4955367-4957132 | 4.00349 | 6.13121 |
| lncRNA38621 | "u" | chr10:60592692-60593695 | 110.874 | 156.516 |
| lncRNA06189 | "x" | chr01:83291990-83297153 | 0.315402 | 2.83343 |
| lncRNA12909 | "x" | chr03:56307472-56309625 | 1.22209 | 0.308982 |
| lncRNA41082 | "x" | chr11:1024920-1026890 | 1.08965 | 0.0833144 |
| lncRNA28803 | "j" | chr07:494018-499725 | 5.89457 | 9.12062 |
| lncRNA38052 | "x" | chr10:41786952-41789243 | 0.865313 | 4.48875 |
| lncRNA29908 | "x" | chr07:59297757-59298457 | 1.69464 | 14.0652 |
| lncRNA26325 | "x" | chr06:38128023-38128852 | 1.06872 | 2.4026 |
| lncRNA32467 | "u" | chr08:5604685-5605551 | 0.456244 | 1.31367 |
| lncRNA04281 | "u" | chr01:4365381-4365755 | 14.7109 | 6.0525 |
| lncRNA13321 | "x" | chr03:60849665-60852290 | 11.1924 | 18.8111 |
| lncRNA33967 | "x" | chr09:1281402-1285331 | 5.14448 | 1.96833 |
| lncRNA02936 | "x" | chr01:79285951-79288255 | 4.67584 | 9.80682 |
| lncRNA13273 | "x" | chr03:60441444-60442427 | 0.247838 | 1.29335 |
| lncRNA22296 | "u" | chr05:20518366-20519703 | 2.28895 | 3.86966 |
| lncRNA40655 | "x" | chr10:62116399-62118573 | 2.58929 | 4.36646 |
| lncRNA29656 | "x" | chr07:55292546-55293092 | 36.4264 | 54.6058 |
| lncRNA19020 | "x" | chr04:51490444-51491092 | 2.40384 | 0.669256 |
| lncRNA16246 | "u" | chr04:2463097-2463925 | 0.955946 | 0.245943 |
| lncRNA17318 | "x" | chr04:56722271-56725055 | 0.256199 | 1.78011 |
| lncRNA44581 | "u" | chr11:53048544-53049101 | 2.06155 | 0.535972 |
| lncRNA11305 | "u" | chr02:48101341-48102157 | 1.06643 | 0.280099 |
| lncRNA11604 | "x" | chr03:1520012-1522985 | 0.348953 | 2.56552 |
| lncRNA42121 | "u" | chr11:36561196-36562065 | 1.03148 | 0.270759 |
| lncRNA43862 | "u" | chr11:35164110-35164878 | 1.1573 | 0.302161 |
| lncRNA02026 | "x" | chr01:59706521-59712895 | 0.823864 | 1.41772 |
| lncRNA26847 | "x" | chr06:43949613-43950310 | 4.13571 | 12.6708 |
| lncRNA05810 | "u" | chr01:79109601-79110690 | 0.324243 | 0.921879 |
| lncRNA40740 | "u" | chr10:62871643-62872161 | 1.37226 | 3.91104 |
| lncRNA15530 | "x" | chr03:59148330-59155113 | 0.162261 | 0.880851 |
| lncRNA15004 | "x" | chr03:50988190-50990791 | 1.2262 | 0.689676 |
| lncRNA09004 | "x" | chr02:48853855-48862875 | 0.895768 | 0.192459 |
| lncRNA40654 | "x" | chr10:62109425-62110361 | 3.59856 | 0.391952 |
| lncRNA27246 | "u" | chr07:2307688-2308570 | 0.287105 | 0.990997 |
| lncRNA02113 | "x" | chr01:65729913-65730404 | 3.35995 | 0.790195 |
| lncRNA22984 | "u" | chr05:63023628-63025776 | 3.12158 | 4.83247 |
| lncRNA38193 | "x" | chr10:48821346-48826073 | 0.239383 | 0.662011 |
| lncRNA41703 | "x" | chr11:10137087-10139788 | 3.14819 | 1.24329 |
| lncRNA06660 | "x" | chr01:88496280-88502147 | 0.0565566 | 1.20925 |
| lncRNA24062 | "x" | chr06:33816613-33818557 | 82.6538 | 178.114 |
| lncRNA37603 | "u" | chr10:4404576-4405809 | 4.85892 | 2.90977 |
| lncRNA01277 | "x" | chr01:2301643-2306017 | 3.36422 | 1.72462 |
| lncRNA00416 | "u" | chr00:17385676-17387398 | 0.79663 | 0.357206 |
| lncRNA08337 | "x" | chr02:41829797-41904084 | 6.60006 | 2.40568 |
| lncRNA27926 | "x" | chr07:54233029-54234010 | 4.42445 | 11.5839 |
| lncRNA44067 | "u" | chr11:46054081-46054289 | 129.191 | 365.115 |
| lncRNA30922 | "x" | chr08:21868473-21871861 | 0.270194 | 0.666734 |
| lncRNA20377 | "u" | chr05:6342520-6343246 | 4.43981 | 2.21912 |
| lncRNA17978 | "u" | chr04:536602-537615 | 2.57285 | 4.47516 |
| lncRNA29352 | "u" | chr07:32898876-32903987 | 2.63866 | 1.77223 |
| lncRNA13018 | "x" | chr03:57498269-57504777 | 5.70524 | 0.941124 |
| lncRNA40162 | "x" | chr10:52871308-52873227 | 4.64405 | 12.4153 |
| lncRNA25058 | "x" | chr06:45066448-45067576 | 1.99596 | 0.470459 |
| lncRNA45161 | "u" | chr12:5119773-5120224 | 3.00899 | 0.489891 |
| lncRNA29558 | "u" | chr07:52592056-52593862 | 0.921735 | 1.68412 |
| lncRNA19008 | "u" | chr04:50913135-50914380 | 0.359964 | 0.93546 |
| lncRNA23878 | "u" | chr06:30708751-30709375 | 1.02421 | 2.63522 |
| lncRNA26756 | "x" | chr06:43056750-43057451 | 15.7848 | 25.6171 |
| lncRNA23468 | "u" | chr06:5410628-5411728 | 2.53073 | 1.27602 |
| lncRNA40146 | "u" | chr10:52526225-52528932 | 0.787309 | 0.395492 |
| lncRNA06855 | "u" | chr02:2067981-2070086 | 2.74287 | 4.28166 |
| lncRNA37432 | "u" | chr10:1985406-1986265 | 1.3652 | 0.504076 |
| lncRNA28789 | "x" | chr07:323978-326854 | 1.2572 | 2.03244 |
| lncRNA01517 | "u" | chr01:11632211-11633005 | 1.51404 | 0.563619 |
| lncRNA45393 | "u" | chr12:20142941-20144244 | 1.22226 | 0.533718 |
| lncRNA46709 | "x" | chr12:1947115-1949585 | 2.43643 | 0.577125 |
| lncRNA35362 | "x" | chr09:65548463-65551101 | 1.15145 | 3.09879 |
| lncRNA14223 | "u" | chr03:10686804-10688287 | 0.588829 | 1.22794 |
| lncRNA27021 | "u" | chr06:45601637-45604754 | 0.572131 | 1.00307 |
| lncRNA40519 | "x" | chr10:60905384-60908374 | 0.393388 | 4.17241 |
| lncRNA34314 | "x" | chr09:7253590-7254258 | 32.4952 | 7.73653 |
| lncRNA05218 | "u" | chr01:70953493-70953755 | 59.2412 | 119.759 |
| lncRNA25985 | "x" | chr06:33695616-33696097 | 9.56995 | 1.05226 |
| lncRNA28226 | "u" | chr07:59272725-59274827 | 0.357443 | 0.744277 |
| lncRNA30451 | "x" | chr07:65176967-65177619 | 0.158023 | 1.64807 |
| lncRNA45100 | "x" | chr12:4461490-4464637 | 11.4473 | 23.2171 |
| lncRNA31102 | "u" | chr08:45567421-45570516 | 4.72368 | 6.79188 |
| lncRNA00434 | "u" | chr00:18038551-18041305 | 1.64224 | 0.97935 |
| lncRNA38131 | "u" | chr10:46660102-46668510 | 2.78802 | 1.9533 |
| lncRNA24148 | "x" | chr06:35266462-35269909 | 5.76608 | 0.990197 |
| lncRNA32099 | "u" | chr08:62638329-62640585 | 2.79542 | 4.28188 |
| lncRNA15016 | "x" | chr03:51317290-51320454 | 0.182519 | 2.25021 |
| lncRNA24870 | "u" | chr06:43225040-43225507 | 3.44798 | 7.72005 |
| lncRNA07034 | "u" | chr02:15487836-15490293 | 1.10823 | 0.318605 |
| lncRNA32881 | "x" | chr08:47084631-47087075 | 3.74081 | 0.442021 |
| lncRNA34846 | "u" | chr09:57009825-57012342 | 1.42319 | 2.27955 |
| lncRNA44052 | "x" | chr11:45477524-45479462 | 11.5357 | 2.72359 |
| lncRNA20491 | "x" | chr05:10281026-10283840 | 4.32949 | 6.3497 |
| lncRNA39201 | "x" | chr10:1199168-1200625 | 0.199182 | 2.45544 |
| lncRNA19099 | "u" | chr04:53611459-53613680 | 0.987883 | 0.512063 |
| lncRNA42270 | "u" | chr11:46579483-46580614 | 1.30285 | 2.48628 |
| lncRNA33254 | "x" | chr08:55639697-55641614 | 0.689633 | 0.277542 |
| lncRNA11120 | "u" | chr02:46245267-46246920 | 2.88078 | 1.69736 |
| lncRNA43725 | "x" | chr11:22608012-22610876 | 0.188716 | 1.46587 |
| lncRNA44126 | "x" | chr11:47407869-47411823 | 1.19961 | 2.59466 |
| lncRNA16616 | "x" | chr04:9607578-9609487 | 3.59726 | 5.4329 |
| lncRNA10233 | "x" | chr02:36777873-36781627 | 0.991812 | 0.461021 |
| lncRNA16975 | "u" | chr04:49317533-49318875 | 0.683326 | 1.3716 |
| lncRNA42185 | "u" | chr11:42545793-42547349 | 0.566957 | 1.14244 |
| lncRNA47659 | "u" | chr12:44633049-44633458 | 6.28056 | 12.587 |
| lncRNA34697 | "u" | chr09:47148684-47149394 | 9.20824 | 14.5945 |
| lncRNA06200 | "u" | chr01:83431513-83431800 | 92.5575 | 48.6219 |
| lncRNA18854 | "u" | chr04:44038352-44039122 | 1.15459 | 2.55567 |
| lncRNA44429 | "x" | chr11:51610236-51611309 | 0.13705 | 0.894939 |
| lncRNA36909 | "u" | chr09:62969995-62971932 | 5.08231 | 3.34568 |
| lncRNA00519 | "u" | chr00:551973-552576 | 2.49943 | 0.937416 |
| lncRNA29907 | "x" | chr07:59281491-59282896 | 1.52465 | 4.26648 |
| lncRNA12330 | "u" | chr03:40852102-40854570 | 1.29778 | 2.12362 |
| lncRNA05245 | "x" | chr01:71451797-71458212 | 0.385993 | 1.74465 |
| lncRNA11751 | "x" | chr03:7202520-7206635 | 0.13693 | 1.34079 |
| lncRNA10200 | "x" | chr02:36332849-36340459 | 3.306 | 0.39177 |
| lncRNA29888 | "x" | chr07:59022786-59023988 | 0.285923 | 3.52962 |
| lncRNA32320 | "u" | chr08:2165267-2166197 | 2.90825 | 1.51949 |
| lncRNA01008 | "x" | chr00:19305527-19306323 | 1.51831 | 4.60069 |
| lncRNA24526 | "u" | chr06:39759531-39760329 | 5.52352 | 3.10893 |
| lncRNA36475 | "u" | chr09:50294502-50297747 | 1.16902 | 2.52747 |
| lncRNA06262 | "x" | chr01:84211289-84215014 | 2.68763 | 4.97348 |
| lncRNA23825 | "x" | chr06:29430734-29434224 | 0.475839 | 5.86246 |
| lncRNA26543 | "x" | chr06:40712568-40715258 | 0.562913 | 1.27385 |
| lncRNA27533 | "u" | chr07:16246297-16248329 | 1.51439 | 0.860941 |
| lncRNA27968 | "x" | chr07:55156872-55162253 | 4.69341 | 0.404614 |
| lncRNA23993 | "x" | chr06:32954971-32960911 | 4.231 | 9.21922 |
| lncRNA36641 | "u" | chr09:58363947-58364604 | 0.48822 | 1.47099 |
| lncRNA21997 | "x" | chr05:6419436-6420695 | 0.691024 | 1.78864 |
| lncRNA34163 | "x" | chr09:3916735-3919570 | 4.90077 | 0.528028 |
| lncRNA09581 | "u" | chr02:28172060-28174009 | 0.611305 | 1.14928 |
| lncRNA18805 | "u" | chr04:39849587-39850876 | 0.516909 | 1.13024 |
| lncRNA17723 | "x" | chr04:61853023-61857677 | 0.587102 | 2.53425 |
| lncRNA07271 | "x" | chr02:28073079-28077641 | 1.31378 | 0.406725 |
| lncRNA39739 | "u" | chr10:28923669-28925928 | 2.5102 | 4.96795 |
| lncRNA14266 | "x" | chr03:12003208-12007502 | 6.02067 | 2.40226 |
| lncRNA34730 | "u" | chr09:49923926-49926312 | 1.05325 | 1.73696 |
| lncRNA38338 | "x" | chr10:54109843-54110654 | 2.7652 | 5.0288 |
| lncRNA19000 | "u" | chr04:50455609-50459013 | 0.752005 | 0.277423 |
| lncRNA13184 | "x" | chr03:59417708-59421304 | 3.14819 | 7.48725 |
| lncRNA10874 | "u" | chr02:43688354-43689094 | 1.43741 | 3.06194 |
| lncRNA38880 | "x" | chr10:63129152-63133192 | 1.75615 | 3.37472 |
| lncRNA35312 | "x" | chr09:64947002-64951962 | 1.31025 | 3.32122 |
| lncRNA22471 | "u" | chr05:36043797-36044967 | 0.754887 | 1.55107 |
| lncRNA15492 | "u" | chr03:58819260-58819771 | 2.06676 | 4.73505 |
| lncRNA32958 | "x" | chr08:49879905-49881913 | 3.53357 | 0.412012 |
| lncRNA19624 | "u" | chr04:61254084-61254473 | 10.6698 | 20.7611 |
| lncRNA17427 | "x" | chr04:58193640-58194365 | 2.35973 | 14.3845 |
| lncRNA06643 | "u" | chr01:88324008-88324567 | 1.61948 | 0.302435 |
| lncRNA23470 | "u" | chr06:5435172-5435417 | 56.829 | 10.3571 |
| lncRNA40376 | "x" | chr10:59592482-59593368 | 4.20103 | 8.24159 |
| lncRNA40690 | "x" | chr10:62400931-62406063 | 1.34426 | 2.6351 |
| lncRNA37332 | "u" | chr10:860396-862326 | 0.532771 | 1.05741 |
| lncRNA38985 | "x" | chr10:63958683-63959686 | 2.08179 | 1.06297 |
| lncRNA20427 | "u" | chr05:7907158-7908345 | 0.992517 | 0.399838 |
| lncRNA09776 | "u" | chr02:31506315-31507298 | 1.24003 | 0.50448 |
| lncRNA30636 | "x" | chr08:2125106-2129164 | 1.10581 | 0.337253 |
| lncRNA07121 | "u" | chr02:18819454-18820714 | 0.929487 | 0.378737 |
| lncRNA17564 | "u" | chr04:60073678-60075112 | 0.192707 | 1.30927 |
| lncRNA20697 | "x" | chr05:28060780-28074769 | 12.9861 | 5.57579 |
| lncRNA36560 | "x" | chr09:56216129-56220107 | 7.89701 | 27.1007 |
| lncRNA47691 | "x" | chr12:45417432-45419715 | 7.80436 | 4.92989 |
| lncRNA24171 | "x" | chr06:35582686-35585154 | 3.75136 | 7.47351 |
| lncRNA04597 | "u" | chr01:24911158-24916365 | 0.952284 | 0.613494 |
| lncRNA14781 | "x" | chr03:46115282-46121575 | 4.90542 | 15.763 |
| lncRNA02145 | "u" | chr01:67344669-67345108 | 3.25453 | 7.34736 |
| lncRNA36356 | "x" | chr09:41860222-41860811 | 6.43636 | 13.4689 |
| lncRNA02060 | "u" | chr01:62505941-62507032 | 0.819616 | 0.324763 |
| lncRNA25421 | "u" | chr06:3640991-3641269 | 15.5584 | 40.9075 |
| lncRNA27109 | "x" | chr07:625769-628788 | 8.48564 | 3.66612 |
| lncRNA12217 | "u" | chr03:27454818-27461192 | 13.7846 | 10.0558 |
| lncRNA10397 | "u" | chr02:38635911-38637045 | 1.10581 | 2.11399 |
| lncRNA11851 | "x" | chr03:8815494-8821682 | 1.17069 | 0.179012 |
| lncRNA07682 | "x" | chr02:34762583-34764796 | 4.63255 | 1.65963 |
| lncRNA34623 | "u" | chr09:37871822-37872853 | 1.48343 | 0.700759 |
| lncRNA14462 | "u" | chr03:25160614-25161486 | 1.62173 | 0.706916 |
| lncRNA46942 | "u" | chr12:4411441-4411874 | 7.63493 | 3.33372 |
| lncRNA19359 | "x" | chr04:57664049-57667533 | 7.57404 | 3.67118 |
| lncRNA45936 | "u" | chr12:47845867-47846399 | 15.6526 | 24.3559 |
| lncRNA25402 | "x" | chr06:3339557-3340984 | 30.8399 | 9.97889 |
| lncRNA01066 | "u" | chr00:21687896-21688909 | 1.4083 | 0.642487 |
| lncRNA29546 | "u" | chr07:51927202-51927989 | 2.18474 | 0.994718 |
| lncRNA19155 | "x" | chr04:54558742-54560834 | 2.85534 | 4.28692 |
| lncRNA08347 | "u" | chr02:41829797-41904084 | 0.380592 | 2.11099 |
| lncRNA07728 | "x" | chr02:35151244-35152689 | 4.34441 | 2.03125 |
| lncRNA15478 | "u" | chr03:58657017-58657516 | 0.557106 | 2.16103 |
| lncRNA41246 | "u" | chr11:2622306-2622800 | 0.575857 | 2.26209 |
| lncRNA16553 | "u" | chr04:7081361-7084058 | 0.524797 | 0.936785 |
| lncRNA45387 | "x" | chr12:19451799-19455248 | 2.044 | 4.08705 |
| lncRNA16111 | "x" | chr04:546765-552341 | 3.44058 | 7.05917 |
| lncRNA20749 | "u" | chr05:31123490-31124578 | 0.342427 | 0.885191 |
| lncRNA33565 | "x" | chr08:59707375-59710158 | 2.09401 | 4.97497 |
| lncRNA03451 | "u" | chr01:84729412-84730601 | 1.61022 | 0.783121 |
| lncRNA18063 | "x" | chr04:1647717-1648329 | 6.70669 | 11.1181 |
| lncRNA29498 | "x" | chr07:49459202-49463605 | 0.615032 | 0.0997212 |
| lncRNA42725 | "x" | chr11:52409695-52412521 | 0.397422 | 3.08512 |
| lncRNA42647 | "x" | chr11:51693103-51699612 | 2.12394 | 3.23466 |
| lncRNA13689 | "x" | chr03:64729996-64734589 | 1.28626 | 0.415391 |
| lncRNA39541 | "o" | chr10:10619521-10622591 | 2.89195 | 4.56149 |
| lncRNA15095 | "x" | chr03:53034150-53040369 | 0.699002 | 3.45549 |
| lncRNA43574 | "u" | chr11:12951668-12953495 | 5.15678 | 7.56298 |
| lncRNA19977 | "x" | chr05:734224-736907 | 6.5627 | 11.6437 |
| lncRNA31294 | "x" | chr08:51340059-51354381 | 1.57949 | 0.561749 |
| lncRNA43233 | "x" | chr11:4283300-4285910 | 6.56263 | 0.763866 |
| lncRNA43118 | "x" | chr11:2971602-2977020 | 1.77194 | 0.68509 |
| lncRNA25836 | "x" | chr06:30986025-30988348 | 0.881247 | 0.0884018 |
| lncRNA02341 | "x" | chr01:71223650-71229871 | 0.315414 | 1.69323 |
| lncRNA45522 | "u" | chr12:32412365-32413771 | 5.84075 | 8.57217 |
| lncRNA14100 | "u" | chr03:8776672-8777627 | 0.75891 | 1.6611 |
| lncRNA28071 | "u" | chr07:56985392-56986266 | 3.24422 | 5.43776 |
| lncRNA21482 | "x" | chr05:64483048-64485380 | 0.481376 | 2.08471 |
| lncRNA20589 | "u" | chr05:19330322-19334457 | 5.33122 | 7.36297 |
| lncRNA45785 | "x" | chr12:44736140-44739232 | 2.73312 | 1.22952 |
| lncRNA39740 | "u" | chr10:28923669-28925928 | 3.33117 | 6.73821 |
| lncRNA04450 | "x" | chr01:11829545-11830143 | 1.04526 | 2.52382 |
| lncRNA20961 | "u" | chr05:53790065-53790510 | 2.96154 | 0.970948 |
| lncRNA22074 | "u" | chr05:8064322-8064674 | 7.63542 | 2.48935 |
| lncRNA37791 | "u" | chr10:20512715-20513050 | 10.26 | 3.31422 |
| lncRNA14105 | "u" | chr03:8833810-8834243 | 4.50491 | 1.58585 |
| lncRNA46848 | "x" | chr12:3406946-3409965 | 3.2115 | 6.51821 |
| lncRNA24195 | "u" | chr06:35900231-35902204 | 1.34457 | 2.17434 |
| lncRNA01980 | "u" | chr01:56993584-56997276 | 4.11562 | 2.5857 |
| lncRNA05761 | "u" | chr01:78554341-78554742 | 3.35718 | 8.06237 |
| lncRNA22391 | "u" | chr05:28094435-28095454 | 0.448752 | 1.07875 |
| lncRNA05639 | "x" | chr01:76952591-76957268 | 0.543783 | 3.52874 |
| lncRNA28225 | "u" | chr07:59271353-59272570 | 7.47539 | 11.002 |
| lncRNA31893 | "u" | chr08:60448279-60448787 | 2.8607 | 1.01781 |
| lncRNA46548 | "x" | chr12:269846-271334 | 1.12733 | 14.6021 |
| lncRNA08827 | "x" | chr02:47031053-47041018 | 0.349193 | 0.837403 |
| lncRNA24388 | "x" | chr06:38231524-38238976 | 4.52252 | 14.3571 |
| lncRNA30671 | "u" | chr08:2565504-2566499 | 2.71621 | 1.47733 |
| lncRNA48200 | "x" | chr12:63859696-63864278 | 2.26874 | 6.96085 |
| lncRNA30900 | "u" | chr08:17261434-17264413 | 0.862257 | 0.49476 |
| lncRNA18972 | "u" | chr04:50080622-50081736 | 0.612924 | 1.32171 |
| lncRNA40009 | "x" | chr10:48253241-48253629 | 21.0105 | 9.66716 |
| lncRNA01947 | "x" | chr01:53823542-53828955 | 0.842896 | 1.31198 |
| lncRNA48271 | "u" | chr12:64533251-64535057 | 0.864636 | 0.42974 |
| lncRNA14023 | "x" | chr03:7800795-7802414 | 12.8942 | 1.67417 |
| lncRNA44751 | "x" | chr12:879276-887765 | 1.02315 | 3.22921 |
| lncRNA17062 | "x" | chr04:51649198-51650772 | 3.14046 | 4.84514 |
| lncRNA08035 | "x" | chr02:38747837-38748390 | 1.10242 | 3.17381 |
| lncRNA29372 | "u" | chr07:35815126-35835301 | 5.35424 | 2.57655 |
| lncRNA01076 | "x" | chr01:104190-107414 | 3.29465 | 1.36295 |
| lncRNA06023 | "x" | chr01:81486230-81494215 | 0.27636 | 0.642923 |
| lncRNA40703 | "x" | chr10:62525994-62530672 | 6.65343 | 14.1469 |
| lncRNA29363 | "u" | chr07:33874774-33876789 | 2.86753 | 4.27789 |
| lncRNA37825 | "u" | chr10:21786927-21787822 | 13.8144 | 9.11345 |
| lncRNA35710 | "x" | chr09:1486828-1491959 | 4.26689 | 12.1614 |
| lncRNA30858 | "u" | chr08:10176682-10177858 | 0.39971 | 0.950306 |
| lncRNA35709 | "u" | chr09:1486247-1486718 | 2.07155 | 4.93765 |
| lncRNA42794 | "u" | chr11:53244330-53244752 | 2.73448 | 6.5072 |
| lncRNA48062 | "x" | chr12:62634157-62636860 | 28.5031 | 46.5932 |
| lncRNA37260 | "u" | chr09:67516631-67518097 | 5.32668 | 7.8018 |
| lncRNA10610 | "x" | chr02:40997738-41004775 | 4.36745 | 3.16644 |
| lncRNA43402 | "u" | chr11:6335144-6335998 | 1.01652 | 2.10881 |
| lncRNA27365 | "x" | chr07:4571507-4572788 | 1.76465 | 0.4526 |
| lncRNA29569 | "u" | chr07:52807054-52810219 | 13.3858 | 18.4727 |
| lncRNA38194 | "x" | chr10:48821346-48826073 | 2.06447 | 1.34918 |
| lncRNA05961 | "x" | chr01:80834333-80837989 | 1.1184 | 2.097 |
| lncRNA04609 | "u" | chr01:25857795-25858825 | 2.34042 | 3.91475 |
| lncRNA44312 | "x" | chr11:50274715-50277867 | 1.99345 | 9.52203 |
| lncRNA43128 | "x" | chr11:3081867-3084392 | 3.83209 | 2.53525 |
| lncRNA22019 | "x" | chr05:6883576-6886664 | 8.67635 | 15.4994 |
| lncRNA42706 | "x" | chr11:52236772-52240135 | 2.71961 | 1.66766 |
| lncRNA33393 | "x" | chr08:57606500-57612289 | 0.0798731 | 0.617931 |
| lncRNA07336 | "x" | chr02:29744655-29747099 | 2.62919 | 1.36566 |
| lncRNA19418 | "x" | chr04:58440453-58446202 | 0.54101 | 1.04951 |
| lncRNA28262 | "x" | chr07:59897069-59898814 | 2.9415 | 6.06807 |
| lncRNA34791 | "x" | chr09:54256323-54260377 | 0.654867 | 1.86658 |
| lncRNA44937 | "x" | chr12:2768265-2794422 | 1.20718 | 3.97324 |
| lncRNA48392 | "u" | chr12:65456343-65458510 | 1.18774 | 0.673677 |
| lncRNA47926 | "x" | chr12:55015113-55018149 | 1.23913 | 1.91694 |
| lncRNA06639 | "x" | chr01:88288062-88291766 | 0.372291 | 1.04011 |
| lncRNA23306 | "x" | chr06:1759349-1761388 | 1.14725 | 0.14849 |
| lncRNA40876 | "x" | chr10:64036325-64052725 | 0.786418 | 2.84035 |
| lncRNA08335 | "o" | chr02:41829797-41904084 | 14.8512 | 26.0304 |
| lncRNA22729 | "u" | chr05:58547564-58548208 | 2.41334 | 1.0425 |
| lncRNA39347 | "u" | chr10:2916983-2917930 | 1.41944 | 0.61503 |
| lncRNA32574 | "u" | chr08:15755125-15756635 | 1.14824 | 0.574922 |
| lncRNA42388 | "u" | chr11:48610510-48611160 | 4.1865 | 2.09464 |
| lncRNA38412 | "x" | chr10:58060808-58063925 | 6.7031 | 12.8413 |
| lncRNA09013 | "u" | chr02:48957923-48958873 | 1.26886 | 0.551233 |
| lncRNA35172 | "x" | chr09:63166304-63171727 | 0.298643 | 0.727815 |
| lncRNA43881 | "x" | chr11:35784021-35790680 | 7.87947 | 11.6723 |
| lncRNA14670 | "x" | chr03:42106418-42108344 | 1.20922 | 2.90522 |
| lncRNA36120 | "u" | chr09:14238117-14239901 | 1.01726 | 1.72177 |
| lncRNA21009 | "x" | chr05:56289278-56290574 | 1.08004 | 0.520985 |
| lncRNA32555 | "u" | chr08:11357213-11358131 | 1.30489 | 0.568526 |
| lncRNA19680 | "u" | chr04:61763599-61764170 | 3.53887 | 1.62168 |
| lncRNA30095 | "u" | chr07:61283101-61283355 | 91.8765 | 41.8805 |
| lncRNA01023 | "u" | chr00:19990465-19991601 | 2.71807 | 1.55116 |
| lncRNA30895 | "o" | chr08:16981139-17000668 | 5.2288 | 8.15822 |
| lncRNA08873 | "u" | chr02:47547073-47548400 | 2.54755 | 1.52426 |
| lncRNA44063 | "x" | chr11:45917537-45921822 | 6.85585 | 9.25523 |
| lncRNA04316 | "x" | chr01:5338903-5342294 | 0.363254 | 2.13097 |
| lncRNA18362 | "u" | chr04:5471825-5472706 | 1.62227 | 0.748232 |
| lncRNA14888 | "x" | chr03:47847207-47852705 | 0.20481 | 0.796069 |
| lncRNA23522 | "u" | chr06:10701722-10702477 | 10.8571 | 7.09509 |
| lncRNA02411 | "u" | chr01:72573754-72574550 | 1.37526 | 0.552065 |
| lncRNA18781 | "u" | chr04:38227863-38228849 | 0.979902 | 0.392792 |
| lncRNA00191 | "u" | chr00:12121314-12121594 | 68.3831 | 32.5527 |
| lncRNA15958 | "x" | chr03:63694646-63696266 | 0.346078 | 0.818855 |
| lncRNA38227 | "x" | chr10:50710892-50717005 | 0.624063 | 1.55287 |
| lncRNA20552 | "x" | chr05:14383565-14385313 | 0.479843 | 1.24408 |
| lncRNA41989 | "u" | chr11:30393190-30393584 | 0.586087 | 3.10724 |
| lncRNA07532 | "x" | chr02:32661806-32665430 | 4.13879 | 7.24129 |
| lncRNA22579 | "u" | chr05:50833664-50834613 | 1.43086 | 0.626661 |
| lncRNA10594 | "x" | chr02:40875351-40878689 | 0.633351 | 1.48208 |
| lncRNA44360 | "x" | chr11:50742548-50745460 | 7.73598 | 15.2956 |
| lncRNA46859 | "x" | chr12:3467927-3470579 | 1.02825 | 5.66627 |
| lncRNA37625 | "x" | chr10:5095099-5108835 | 0.397032 | 1.24711 |
| lncRNA02731 | "x" | chr01:76818585-76826601 | 1.61374 | 0.737439 |
| lncRNA19848 | "u" | chr04:63526587-63529937 | 2.04851 | 1.37342 |
| lncRNA34662 | "u" | chr09:43025673-43025967 | 44.28 | 21.2296 |
| lncRNA42358 | "u" | chr11:48136023-48143512 | 119.416 | 161.144 |
| lncRNA03387 | "u" | chr01:84144815-84145948 | 1.68623 | 0.873387 |
| lncRNA19626 | "x" | chr04:61264316-61267652 | 1.1133 | 3.44098 |
| lncRNA22746 | "x" | chr05:59233532-59235230 | 2.92654 | 6.92285 |
| lncRNA31476 | "x" | chr08:54841495-54845522 | 42.6693 | 25.2863 |
| lncRNA13240 | "x" | chr03:59978698-59982625 | 3.55904 | 1.40188 |
| lncRNA14486 | "x" | chr03:27042532-27044026 | 19.7158 | 10.6776 |
| lncRNA39689 | "u" | chr10:22853119-22854107 | 3.33281 | 1.94527 |
| lncRNA04271 | "x" | chr01:3979008-3982118 | 1.72126 | 0.585702 |
| lncRNA47934 | "u" | chr12:55264417-55265954 | 1.25766 | 0.692011 |
| lncRNA03569 | "u" | chr01:85878926-85891837 | 10.6222 | 3.9351 |
| lncRNA10542 | "x" | chr02:40216886-40218892 | 26.3762 | 11.272 |
| lncRNA16437 | "u" | chr04:4788634-4789193 | 0.757914 | 2.00477 |
| lncRNA48002 | "x" | chr12:62086486-62092749 | 0.897856 | 2.81814 |
| lncRNA03748 | "u" | chr01:87722458-87722922 | 1.36153 | 3.55155 |
| lncRNA17015 | "u" | chr04:50226981-50227692 | 0.468283 | 1.22611 |
| lncRNA40952 | "x" | chr10:64646686-64649407 | 2.43242 | 0.35001 |
| lncRNA04843 | "x" | chr01:56576300-56580082 | 2.04333 | 12.5016 |
| lncRNA32338 | "u" | chr08:2432611-2435422 | 0.652859 | 0.355935 |
| lncRNA43909 | "u" | chr11:36433104-36434209 | 2.51654 | 1.42636 |
| lncRNA02465 | "u" | chr01:73160483-73161210 | 3.30957 | 1.72532 |
| lncRNA03077 | "x" | chr01:80784775-80786776 | 2.44988 | 3.66108 |
| lncRNA18486 | "x" | chr04:9603163-9604130 | 50.4842 | 122.879 |
| lncRNA15164 | "x" | chr03:53969221-53976547 | 1.89739 | 6.6138 |
| lncRNA38813 | "x" | chr10:62319637-62320429 | 5.04908 | 2.91346 |
| lncRNA43307 | "x" | chr11:5057081-5059346 | 0.500841 | 2.76379 |
| lncRNA27282 | "x" | chr07:3015218-3024992 | 1.95184 | 1.25201 |
| lncRNA35022 | "o" | chr09:60861307-60879115 | 13.3627 | 26.8611 |
| lncRNA31146 | "x" | chr08:46850285-46854202 | 1.18904 | 0.562416 |
| lncRNA25435 | "x" | chr06:4828048-4830379 | 1.73544 | 1.09524 |
| lncRNA05067 | "u" | chr01:67642596-67644451 | 3.47526 | 5.05092 |
| lncRNA29369 | "x" | chr07:35365430-35368572 | 0.39859 | 1.03032 |
| lncRNA02640 | "x" | chr01:75383650-75386800 | 0.179072 | 1.16305 |
| lncRNA09129 | "u" | chr02:3321390-3321760 | 31.427 | 49.6848 |
| lncRNA05540 | "x" | chr01:75618086-75620119 | 0.549479 | 1.23277 |
| lncRNA25779 | "x" | chr06:29483310-29483960 | 24.0839 | 113.517 |
| lncRNA14698 | "u" | chr03:43892262-43897490 | 1.31815 | 0.891176 |
| lncRNA06497 | "u" | chr01:86805164-86805790 | 7.35053 | 12.5864 |
| lncRNA16010 | "x" | chr03:64224833-64226287 | 16.1705 | 25.096 |
| lncRNA26262 | "x" | chr06:37382749-37391688 | 0.169673 | 0.73321 |
| lncRNA05150 | "u" | chr01:69695593-69696196 | 8.59731 | 5.05846 |
| lncRNA48098 | "x" | chr12:63072437-63075882 | 5.68248 | 14.7446 |
| lncRNA25961 | "x" | chr06:33431057-33440035 | 0.975102 | 2.34299 |
| lncRNA12990 | "x" | chr03:57260421-57261580 | 1.25592 | 0.298659 |
| lncRNA17597 | "x" | chr04:60507839-60511703 | 0.786961 | 2.61818 |
| lncRNA18376 | "x" | chr04:5667084-5670819 | 0.893175 | 0.258792 |
| lncRNA30747 | "x" | chr08:3776676-3777306 | 1.84765 | 0.6585 |
| lncRNA19717 | "u" | chr04:62120841-62121115 | 26.5678 | 56.3812 |
| lncRNA22302 | "u" | chr05:20710063-20710996 | 0.749806 | 1.5917 |
| lncRNA05342 | "u" | chr01:72992176-72993107 | 1.71792 | 3.10423 |
| lncRNA38568 | "x" | chr10:60064010-60068542 | 9.82567 | 20.9482 |
| lncRNA12755 | "u" | chr03:52870522-52871087 | 0.401481 | 1.32048 |
| lncRNA37748 | "u" | chr10:15670244-15670553 | 3.75192 | 12.2888 |
| lncRNA47750 | "x" | chr12:46716105-46778644 | 0.621461 | 2.35818 |
| lncRNA10872 | "x" | chr02:43675271-43676632 | 4.09293 | 0.973447 |
| lncRNA05038 | "x" | chr01:66882697-66885513 | 1.56854 | 2.32203 |
| lncRNA38536 | "x" | chr10:59742410-59744990 | 29.9268 | 5.48599 |
| lncRNA18137 | "u" | chr04:2676024-2676343 | 2.93616 | 9.44443 |
| lncRNA26013 | "x" | chr06:34160074-34160632 | 44.9588 | 63.7326 |
| lncRNA02025 | "x" | chr01:59706521-59712895 | 0.619818 | 0.229078 |
| lncRNA48357 | "x" | chr12:65169218-65173922 | 4.73773 | 2.57119 |
| lncRNA06889 | "u" | chr02:6334652-6346940 | 1.58324 | 3.54291 |
| lncRNA05580 | "x" | chr01:76330160-76349305 | 6.41031 | 1.81964 |
| lncRNA44414 | "x" | chr11:51383608-51390090 | 19.8451 | 12.4064 |
| lncRNA43921 | "x" | chr11:36544956-36552387 | 0.712331 | 2.31808 |
| lncRNA10910 | "x" | chr02:44154223-44155035 | 2.95568 | 5.97742 |
| lncRNA34986 | "x" | chr09:60034856-60044112 | 0.994865 | 1.51437 |
| lncRNA26285 | "x" | chr06:37743175-37743937 | 7.64968 | 12.6385 |
| lncRNA10606 | "x" | chr02:40967988-40969129 | 13.0435 | 3.17692 |
| lncRNA24444 | "x" | chr06:38837658-38843313 | 5.42673 | 2.8933 |
| lncRNA41379 | "u" | chr11:4220171-4221833 | 0.923842 | 0.467294 |
| lncRNA18125 | "x" | chr04:2554628-2556300 | 0.266019 | 1.74251 |
| lncRNA33936 | "u" | chr09:956973-957624 | 1.59832 | 0.62287 |
| lncRNA39873 | "u" | chr10:41643549-41643816 | 46.362 | 18.0746 |
| lncRNA26668 | "o" | chr06:41997666-42009879 | 59.2954 | 5.33695 |
| lncRNA14858 | "x" | chr03:47216664-47217701 | 8.54796 | 24.613 |
| lncRNA26446 | "x" | chr06:39485968-39495244 | 3.01955 | 2.22904 |
| lncRNA12730 | "x" | chr03:52534418-52537953 | 2.02022 | 1.2914 |
| lncRNA44111 | "x" | chr11:47171998-47179611 | 0.992089 | 1.78915 |
| lncRNA42686 | "x" | chr11:52052669-52053790 | 1.94519 | 4.21286 |
| lncRNA12143 | "x" | chr03:22085639-22092694 | 2.57525 | 6.06763 |
| lncRNA02063 | "x" | chr01:62615921-62617433 | 0.39756 | 1.15631 |
| lncRNA22080 | "x" | chr05:8101599-8102295 | 1.68773 | 0.731248 |
| lncRNA22692 | "u" | chr05:57619541-57622045 | 0.741777 | 0.364601 |
| lncRNA28895 | "x" | chr07:1792962-1795743 | 0.235101 | 1.22521 |
| lncRNA43759 | "x" | chr11:25529510-25532073 | 3.75294 | 7.04122 |
| lncRNA31572 | "x" | chr08:56464525-56468564 | 0.260063 | 0.783625 |
| lncRNA38586 | "x" | chr10:60231058-60236675 | 8.31734 | 6.16023 |
| lncRNA14519 | "u" | chr03:29486126-29488092 | 3.7638 | 5.37269 |
| lncRNA43350 | "x" | chr11:5485301-5487622 | 8.65407 | 4.15529 |
| lncRNA11810 | "x" | chr03:8081846-8084677 | 0.668088 | 2.01543 |
| lncRNA37158 | "x" | chr09:66290351-66293260 | 29.5697 | 54.8223 |
| lncRNA02159 | "u" | chr01:67707147-67708404 | 3.01036 | 1.88175 |
| lncRNA05670 | "x" | chr01:77433500-77439308 | 3.42019 | 12.2144 |
| lncRNA25888 | "x" | chr06:32163037-32167768 | 1.13599 | 0.357583 |
| lncRNA15038 | "u" | chr03:51844334-51846305 | 0.769237 | 0.419149 |
| lncRNA34382 | "u" | chr09:11277663-11278121 | 6.46945 | 11.5388 |
| lncRNA10803 | "x" | chr02:42931627-42936171 | 0.414355 | 1.1196 |
| lncRNA24786 | "x" | chr06:42310746-42311445 | 4.23024 | 2.35682 |
| lncRNA36384 | "x" | chr09:44378802-44381132 | 0.376924 | 1.84158 |
| lncRNA37795 | "u" | chr10:20821461-20824915 | 19.1331 | 25.4051 |
| lncRNA18414 | "u" | chr04:6711372-6713393 | 0.398756 | 0.729965 |
| lncRNA36118 | "x" | chr09:14009122-14011000 | 1.45859 | 11.4197 |
| lncRNA48361 | "x" | chr12:65186152-65189023 | 3.95024 | 2.08713 |
| lncRNA10626 | "u" | chr02:41223564-41223877 | 26.5942 | 12.8086 |
| lncRNA10711 | "x" | chr02:42021136-42030203 | 1.59577 | 3.12579 |
| lncRNA32010 | "x" | chr08:61808086-61811078 | 3.81977 | 5.81033 |
| lncRNA17116 | "u" | chr04:53057124-53058641 | 2.08118 | 1.25717 |
| lncRNA24302 | "x" | chr06:37127111-37132449 | 3.56178 | 1.46306 |
| lncRNA31054 | "x" | chr08:39566684-39572160 | 5.04421 | 0.786331 |
| lncRNA35890 | "x" | chr09:4169207-4172534 | 1.33259 | 3.86992 |
| lncRNA44327 | "x" | chr11:50407357-50413384 | 7.19098 | 3.389 |
| lncRNA36742 | "u" | chr09:60493085-60493429 | 17.3289 | 8.39987 |
| lncRNA06420 | "x" | chr01:85988825-85990630 | 3.37241 | 12.7171 |
| lncRNA36198 | "u" | chr09:21022259-21057350 | 0.468444 | 1.27666 |
| lncRNA34399 | "j" | chr09:12693828-12695864 | 13.8251 | 19.7697 |
| lncRNA25763 | "u" | chr06:29020709-29021394 | 3.7193 | 6.14038 |
| lncRNA34931 | "u" | chr09:59187435-59188243 | 3.50459 | 6.68915 |
| lncRNA29981 | "x" | chr07:60092915-60096795 | 0.762282 | 0.164682 |
| lncRNA38654 | "x" | chr10:60870627-60872562 | 8.63395 | 11.9315 |
| lncRNA40220 | "x" | chr10:57343209-57344959 | 0.452308 | 0.876315 |
| lncRNA42832 | "x" | chr11:163008-164425 | 4.51275 | 1.27064 |
| lncRNA11251 | "x" | chr02:47453551-47465026 | 0.590378 | 1.16242 |
| lncRNA45042 | "x" | chr12:3710824-3713679 | 16.5672 | 34.0961 |
| lncRNA17030 | "x" | chr04:50452723-50453664 | 1.05627 | 0.458895 |
| lncRNA37305 | "x" | chr10:541988-544410 | 13.8119 | 18.7804 |
| lncRNA36339 | "u" | chr09:40291405-40292306 | 0.430817 | 1.01122 |
| lncRNA40855 | "u" | chr10:63840164-63840842 | 0.722274 | 1.69268 |
| lncRNA22756 | "x" | chr05:59415664-59420894 | 1.30421 | 5.09214 |
| lncRNA20857 | "u" | chr05:42567979-42570882 | 0.63751 | 2.09857 |
| lncRNA10011 | "u" | chr02:34352037-34353913 | 1.7997 | 2.74991 |
| lncRNA09928 | "u" | chr02:33372102-33372895 | 44.786 | 22.9176 |
| lncRNA09471 | "o" | chr02:21672612-21674488 | 6.23854 | 4.34796 |
| lncRNA08351 | "x" | chr02:41925428-41948678 | 1.12344 | 0.184844 |
| lncRNA36208 | "u" | chr09:22289116-22293416 | 2.27751 | 3.14635 |
| lncRNA32994 | "u" | chr08:51166482-51167494 | 1.03565 | 0.480274 |
| lncRNA15747 | "u" | chr03:61430350-61431530 | 0.924802 | 0.430843 |
| lncRNA39315 | "u" | chr10:2576155-2577125 | 1.07496 | 0.499285 |
| lncRNA47082 | "u" | chr12:6306124-6307229 | 0.988405 | 0.459639 |
| lncRNA37688 | "x" | chr10:8654987-8661854 | 1.36652 | 0.708596 |
| lncRNA29399 | "x" | chr07:38873081-38875661 | 0.647326 | 1.0731 |
| lncRNA17543 | "x" | chr04:59848287-59850129 | 3.84505 | 10.3346 |
| lncRNA34663 | "x" | chr09:43026162-43028277 | 0.323654 | 0.614035 |
| lncRNA15333 | "x" | chr03:57000246-57002379 | 7.09629 | 1.13804 |
| lncRNA18736 | "u" | chr04:32413811-32416296 | 0.568237 | 0.969878 |
| lncRNA19444 | "x" | chr04:58837498-58839706 | 1.22797 | 4.77237 |
| lncRNA44898 | "x" | chr12:2340212-2343067 | 3.22836 | 9.02015 |
| lncRNA39773 | "x" | chr10:34709501-34712585 | 0.291418 | 0.756425 |
| lncRNA04682 | "u" | chr01:36167169-36169126 | 1.57279 | 0.964474 |
| lncRNA05527 | "x" | chr01:75489756-75492068 | 1.82854 | 2.81587 |
| lncRNA23509 | "x" | chr06:9456303-9470235 | 0.830789 | 0.220387 |
| lncRNA40173 | "u" | chr10:53496847-53497797 | 1.46516 | 2.58255 |
| lncRNA00124 | "x" | chr00:10682530-10686239 | 2.82374 | 6.51578 |
| lncRNA08448 | "u" | chr02:43083015-43085280 | 1.16303 | 0.698116 |
| lncRNA03353 | "x" | chr01:83760510-83763127 | 2.93966 | 0.575873 |
| lncRNA37142 | "u" | chr09:66010219-66010831 | 3.0935 | 5.42409 |
| lncRNA35282 | "x" | chr09:64763329-64764151 | 1.36753 | 2.7634 |
| lncRNA41383 | "x" | chr11:4263105-4264776 | 4.63479 | 1.49721 |
| lncRNA28198 | "x" | chr07:58939286-58949834 | 2.46063 | 6.06211 |
| lncRNA44502 | "u" | chr11:52284730-52286350 | 3.93266 | 5.63225 |
| lncRNA43202 | "x" | chr11:3800770-3802226 | 1.30105 | 4.10077 |
| lncRNA18953 | "u" | chr04:49552205-49553075 | 1.34313 | 0.627838 |
| lncRNA46976 | "x" | chr12:4840025-4842602 | 1.1912 | 0.465157 |
| lncRNA13895 | "x" | chr03:3326960-3327987 | 1.54662 | 0.827727 |
| lncRNA20684 | "u" | chr05:28037762-28039172 | 0.936917 | 0.481014 |
| lncRNA07939 | "x" | chr02:37621511-37624964 | 12.6086 | 1.62245 |
| lncRNA29606 | "x" | chr07:53840265-53846757 | 8.34512 | 14.7003 |
| lncRNA11373 | "x" | chr02:48807405-48814692 | 3.04447 | 1.70264 |
| lncRNA17531 | "x" | chr04:59682861-59683449 | 2.85428 | 6.27636 |
| lncRNA17618 | "x" | chr04:60754263-60756252 | 47.8107 | 82.1918 |
| lncRNA27470 | "u" | chr07:8871391-8874246 | 0.624344 | 1.00329 |
| lncRNA05958 | "x" | chr01:80787160-80789564 | 2.23706 | 3.76015 |
| lncRNA45901 | "x" | chr12:47420415-47424092 | 5.69059 | 11.9813 |
| lncRNA12741 | "x" | chr03:52698752-52711178 | 2.31761 | 0.754792 |
| lncRNA33138 | "u" | chr08:54043887-54044678 | 0.646082 | 1.41237 |
| lncRNA06015 | "u" | chr01:81449778-81450293 | 3.13605 | 1.3522 |
| lncRNA01857 | "u" | chr01:46550098-46550776 | 1.7935 | 0.778571 |
| lncRNA18873 | "u" | chr04:45366297-45366785 | 3.74962 | 1.63004 |
| lncRNA13668 | "x" | chr03:64506157-64507554 | 1.2867 | 5.43085 |
| lncRNA03645 | "o" | chr01:86618687-86622348 | 0.319854 | 0.898118 |
| lncRNA10619 | "u" | chr02:41116597-41116974 | 5.14782 | 10.5873 |
| lncRNA04834 | "u" | chr01:56357593-56358129 | 2.80925 | 1.22538 |
| lncRNA27818 | "u" | chr07:51177758-51178002 | 88.01 | 38.4624 |
| lncRNA39040 | "u" | chr10:64355218-64356894 | 0.781026 | 0.405881 |
| lncRNA38585 | "x" | chr10:60224745-60226499 | 8.85566 | 3.11434 |
| lncRNA36055 | "u" | chr09:8629546-8631376 | 0.836759 | 0.428423 |
| lncRNA39298 | "x" | chr10:2335366-2338246 | 52.6972 | 19.5429 |
| lncRNA23633 | "x" | chr06:20981415-20983546 | 1.25507 | 2.12895 |
| lncRNA33082 | "x" | chr08:53067357-53074557 | 1.14438 | 0.622021 |
| lncRNA42871 | "u" | chr11:564975-565905 | 8.5222 | 4.59925 |
| lncRNA27590 | "u" | chr07:23584770-23585416 | 3.17409 | 1.65302 |
| lncRNA39882 | "u" | chr10:41892417-41894236 | 0.39658 | 0.755727 |
| lncRNA07243 | "u" | chr02:25858900-25859774 | 1.11301 | 2.11757 |
| lncRNA27648 | "u" | chr07:31759450-31759897 | 28.1539 | 18.7143 |
| lncRNA01007 | "u" | chr00:19257448-19258769 | 1.63264 | 2.62299 |
| lncRNA43929 | "x" | chr11:36707481-36710928 | 7.39002 | 16.1416 |
| lncRNA03935 | "x" | chr01:89782661-89800491 | 1.96388 | 3.26395 |
| lncRNA42608 | "x" | chr11:51272866-51276687 | 2.64101 | 1.41701 |
| lncRNA40349 | "x" | chr10:59327642-59328459 | 11.2293 | 33.3456 |
| lncRNA14162 | "x" | chr03:9662767-9663558 | 2.34855 | 0.672677 |
| lncRNA37377 | "x" | chr10:1205052-1206786 | 0.693981 | 1.23799 |
| lncRNA19310 | "u" | chr04:57127553-57130871 | 5.21341 | 2.98091 |
| lncRNA10406 | "x" | chr02:38763923-38765847 | 1.24807 | 3.02924 |
| lncRNA32797 | "u" | chr08:41640918-41641898 | 1.81594 | 0.984411 |
| lncRNA48342 | "x" | chr12:65082353-65085848 | 1.96785 | 2.80009 |
| lncRNA16566 | "u" | chr04:7387140-7387477 | 50.2859 | 30.7541 |
| lncRNA17710 | "x" | chr04:61713197-61714235 | 1.23006 | 2.73244 |
| lncRNA22023 | "x" | chr05:6934257-6935131 | 20.2867 | 33.0404 |
| lncRNA45843 | "x" | chr12:46210405-46217854 | 6.39631 | 11.492 |
| lncRNA23626 | "u" | chr06:20717539-20718584 | 0.430374 | 0.926445 |
| lncRNA04828 | "u" | chr01:54722309-54723211 | 0.974251 | 1.84769 |
| lncRNA16261 | "x" | chr04:2582021-2584296 | 0.880246 | 4.68091 |
| lncRNA35950 | "x" | chr09:5033861-5037954 | 1.33306 | 3.98533 |
| lncRNA04046 | "x" | chr01:868612-870137 | 0.862317 | 0.149642 |
| lncRNA27190 | "x" | chr07:1761096-1764642 | 2.24626 | 4.55621 |
| lncRNA24485 | "o" | chr06:39240731-39241942 | 0.823022 | 1.45399 |
| lncRNA43102 | "x" | chr11:2778592-2786040 | 2.21758 | 4.78306 |
| lncRNA08050 | "x" | chr02:38951833-38954839 | 0.399417 | 1.13383 |
| lncRNA47468 | "x" | chr12:36049169-36054118 | 3.86185 | 7.13965 |
| lncRNA08868 | "x" | chr02:47486151-47487760 | 2.00731 | 4.63905 |
| lncRNA13322 | "x" | chr03:60849665-60852290 | 1.4669 | 2.8364 |
| lncRNA30056 | "x" | chr07:60870557-60878823 | 2.10689 | 1.20355 |
| lncRNA04359 | "u" | chr01:6545992-6548425 | 2.15831 | 3.08908 |
| lncRNA02821 | "u" | chr01:78012540-78013320 | 1.51172 | 0.752609 |
| lncRNA12498 | "u" | chr03:46430670-46431024 | 12.5798 | 6.23328 |
| lncRNA46182 | "u" | chr12:62660311-62661161 | 1.34654 | 0.671173 |
| lncRNA47086 | "x" | chr12:6401183-6407372 | 9.93669 | 6.08282 |
| lncRNA19955 | "x" | chr05:488526-489409 | 7.93416 | 4.00691 |
| lncRNA18485 | "u" | chr04:9586783-9587549 | 2.23515 | 3.83593 |
| lncRNA45356 | "x" | chr12:15406045-15407437 | 5.13365 | 2.98785 |
| lncRNA21562 | "x" | chr05:407904-410949 | 2.94359 | 1.50578 |
| lncRNA46846 | "x" | chr12:3377662-3380696 | 0.475113 | 1.03156 |
| lncRNA40107 | "x" | chr10:51540878-51541671 | 4.40848 | 10.4166 |
| lncRNA03435 | "u" | chr01:84595532-84629171 | 0.274602 | 0.713277 |
| lncRNA08769 | "u" | chr02:46351888-46352484 | 15.2558 | 10.203 |
| lncRNA11829 | "x" | chr03:8366727-8371789 | 5.54069 | 3.44729 |
| lncRNA12423 | "x" | chr03:45420237-45422288 | 0.428775 | 0.786292 |
| lncRNA47370 | "u" | chr12:30536582-30537533 | 0.944602 | 1.78222 |
| lncRNA43260 | "u" | chr11:4615315-4618280 | 2.88131 | 3.99726 |
| lncRNA23466 | "x" | chr06:5289008-5295897 | 4.78546 | 7.16261 |
| lncRNA18951 | "x" | chr04:49453193-49470655 | 3.21264 | 5.67743 |
| lncRNA42055 | "x" | chr11:35169112-35170391 | 6.02789 | 2.81566 |
| lncRNA34837 | "u" | chr09:56669090-56669580 | 19.983 | 13.4074 |
| lncRNA41068 | "x" | chr11:899284-912577 | 2.17941 | 1.06563 |
| lncRNA02124 | "u" | chr01:66756105-66758467 | 0.74656 | 0.393727 |
| lncRNA06955 | "u" | chr02:11961897-11962330 | 1.95124 | 0.510064 |
| lncRNA37560 | "x" | chr10:3413723-3418293 | 0.45134 | 2.33513 |
| lncRNA31147 | "x" | chr08:46854796-46866346 | 1.01088 | 0.653989 |
| lncRNA25151 | "u" | chr06:210685-211402 | 2.54482 | 4.35631 |
| lncRNA18227 | "x" | chr04:3626517-3628265 | 27.5258 | 13.7856 |
| lncRNA38419 | "x" | chr10:58156780-58157715 | 9.06823 | 18.9679 |
| lncRNA17672 | "x" | chr04:61268367-61273656 | 2.34047 | 1.03275 |
| lncRNA12570 | "x" | chr03:47796922-47801246 | 4.90412 | 2.85534 |
| lncRNA39657 | "u" | chr10:21303537-21305294 | 1.82471 | 1.15871 |
| lncRNA23390 | "x" | chr06:3094534-3095484 | 6.01643 | 9.92989 |
| lncRNA31947 | "x" | chr08:60985579-60987278 | 0.803422 | 4.96051 |
| lncRNA41748 | "u" | chr11:11996109-11997565 | 9.05231 | 6.48502 |
| lncRNA43534 | "u" | chr11:10487491-10488062 | 6.01545 | 9.5336 |
| lncRNA19929 | "x" | chr05:245353-246536 | 1.33091 | 3.8051 |
| lncRNA37687 | "x" | chr10:8636004-8651968 | 0.786379 | 0.432543 |
| lncRNA31944 | "x" | chr08:60943132-60947585 | 1.18663 | 2.92117 |
| lncRNA20279 | "x" | chr05:5074705-5075855 | 0.814702 | 1.71679 |
| lncRNA37488 | "u" | chr10:2602278-2602704 | 10.5098 | 17.3942 |
| lncRNA26096 | "x" | chr06:35526430-35531314 | 0.840538 | 2.35384 |
| lncRNA42322 | "u" | chr11:47559709-47562470 | 1.92383 | 2.73593 |
| lncRNA06312 | "x" | chr01:84842344-84845885 | 0.382836 | 0.894981 |
| lncRNA09088 | "x" | chr02:49679753-49699414 | 1.11863 | 0.825699 |
| lncRNA15780 | "u" | chr03:61785860-61786700 | 1.44188 | 2.54735 |
| lncRNA13513 | "x" | chr03:62897652-62903551 | 2.80584 | 1.43468 |
| lncRNA12118 | "u" | chr03:20217923-20220000 | 2.16395 | 1.45066 |
| lncRNA18556 | "x" | chr04:18238047-18251375 | 0.316561 | 0.982498 |
| lncRNA48264 | "x" | chr12:64475985-64479392 | 3.90764 | 2.20843 |
| lncRNA05959 | "u" | chr01:80790696-80791547 | 1.3484 | 2.37768 |
| lncRNA24217 | "x" | chr06:36126486-36128866 | 2.39045 | 5.36654 |
| lncRNA16568 | "x" | chr04:7438208-7447119 | 0.353076 | 0.798539 |
| lncRNA34174 | "x" | chr09:3969238-3971560 | 36.2199 | 73.6581 |
| lncRNA35972 | "x" | chr09:5483366-5484336 | 7.46124 | 4.1322 |
| lncRNA01850 | "x" | chr01:45655438-45657074 | 1.06345 | 0.48677 |
| lncRNA32941 | "x" | chr08:49555123-49557702 | 5.41137 | 2.16353 |
| lncRNA02789 | "x" | chr01:77641583-77645425 | 0.916367 | 2.31994 |
| lncRNA17968 | "x" | chr04:444511-446828 | 2.59377 | 22.7842 |
| lncRNA45344 | "x" | chr12:14397154-14399601 | 40.5274 | 31.3178 |
| lncRNA16415 | "x" | chr04:4494772-4495647 | 1.49156 | 4.23534 |
| lncRNA46068 | "u" | chr12:56881962-56883252 | 1.3307 | 0.758731 |
| lncRNA13149 | "u" | chr03:59071156-59071851 | 11.4035 | 16.2318 |
| lncRNA17113 | "x" | chr04:53024744-53031415 | 0.989168 | 1.95577 |
| lncRNA18088 | "x" | chr04:2007891-2011098 | 2.37013 | 4.80408 |
| lncRNA30430 | "u" | chr07:64996404-64998240 | 1.16149 | 0.679786 |
| lncRNA36502 | "x" | chr09:52621457-52622315 | 87.7794 | 14.3402 |
| lncRNA20148 | "u" | chr05:3047843-3048163 | 4.78698 | 12.3525 |
| lncRNA24442 | "x" | chr06:38807608-38809185 | 5.51979 | 14.2555 |
| lncRNA30665 | "x" | chr08:2502580-2508014 | 0.805675 | 0.174529 |
| lncRNA10327 | "x" | chr02:37776214-37780077 | 0.636205 | 0.117949 |
| lncRNA30688 | "x" | chr08:2794831-2797936 | 5.39614 | 12.0699 |
| lncRNA04217 | "x" | chr01:2877779-2883901 | 2.16509 | 5.71088 |
| lncRNA15446 | "x" | chr03:58392327-58397441 | 2.73168 | 0.413745 |
| lncRNA13495 | "x" | chr03:62704263-62718637 | 0.985288 | 0.402816 |
| lncRNA42645 | "x" | chr11:51686580-51692216 | 4.14441 | 6.43111 |
| lncRNA47105 | "x" | chr12:6737183-6744801 | 0.704381 | 1.9819 |
| lncRNA39319 | "x" | chr10:2625490-2630675 | 2.73481 | 1.89609 |
| lncRNA01874 | "u" | chr01:47296798-47297815 | 1.19397 | 0.421473 |
| lncRNA46915 | "x" | chr12:4033784-4039439 | 0.775889 | 0.344554 |
| lncRNA10964 | "x" | chr02:44655714-44658215 | 1.47383 | 3.73612 |
| lncRNA15470 | "u" | chr03:58609175-58610147 | 2.4962 | 1.46508 |
| lncRNA47600 | "x" | chr12:43327212-43332616 | 0.645269 | 0.22898 |
| lncRNA19646 | "x" | chr04:61391518-61395202 | 3.09042 | 12.8407 |
| lncRNA24249 | "x" | chr06:36509240-36511531 | 4.06945 | 1.65864 |
| lncRNA46486 | "x" | chr12:65281061-65282996 | 8.12186 | 3.56738 |
| lncRNA25892 | "x" | chr06:32207317-32210109 | 3.85343 | 5.43755 |
| lncRNA14532 | "x" | chr03:29845426-29861489 | 0.679849 | 0.312431 |
| lncRNA08414 | "u" | chr02:42697771-42699022 | 9.63818 | 6.94269 |
| lncRNA22104 | "x" | chr05:8814438-8825828 | 1.13025 | 2.93129 |
| lncRNA33278 | "u" | chr08:56063217-56066373 | 1.32702 | 0.884239 |
| lncRNA45182 | "x" | chr12:5559536-5566991 | 1.25532 | 0.760578 |
| lncRNA17419 | "x" | chr04:58137443-58138789 | 25.8082 | 39.4307 |
| lncRNA33185 | "u" | chr08:54788398-54793656 | 3.41298 | 4.48618 |
| lncRNA28942 | "u" | chr07:2299496-2302561 | 0.921519 | 0.584636 |
| lncRNA36461 | "u" | chr09:49810785-49811034 | 120.54 | 60.5513 |
| lncRNA01944 | "u" | chr01:53410401-53419907 | 1.52025 | 0.248576 |
| lncRNA04782 | "x" | chr01:50298954-50299666 | 27.08 | 10.1781 |
| lncRNA20990 | "u" | chr05:55424513-55451601 | 1.67661 | 0.457618 |
| lncRNA06544 | "x" | chr01:87253004-87257799 | 1.30708 | 2.1091 |
| lncRNA15421 | "x" | chr03:58090160-58093921 | 1.45039 | 4.01907 |
| lncRNA08462 | "x" | chr02:43202137-43203800 | 0.435962 | 1.0116 |
| lncRNA13799 | "x" | chr03:1082966-1089867 | 0.611638 | 1.66014 |
| lncRNA12556 | "x" | chr03:47425940-47428695 | 3.20229 | 4.41408 |
| lncRNA03098 | "x" | chr01:80976339-80981277 | 13.1477 | 33.3471 |
| lncRNA26422 | "x" | chr06:39171989-39172835 | 33.6943 | 55.1353 |
| lncRNA47821 | "x" | chr12:47762511-47771707 | 0.535832 | 2.33459 |
| lncRNA27629 | "x" | chr07:28778830-28789074 | 0.94006 | 0.656456 |
| lncRNA40430 | "x" | chr10:60025402-60031277 | 15.5196 | 9.84592 |
| lncRNA43575 | "x" | chr11:12954761-12955405 | 0.151505 | 0.597965 |
| lncRNA08431 | "x" | chr02:42923083-42924081 | 7.72045 | 2.49182 |
| lncRNA40159 | "x" | chr10:52762722-52763931 | 6.50247 | 4.31172 |
| lncRNA42345 | "u" | chr11:47914936-47916438 | 1.76821 | 1.08537 |
| lncRNA17454 | "x" | chr04:58657872-58662339 | 0.223008 | 1.03789 |
| lncRNA40658 | "x" | chr10:62125510-62134770 | 1.49344 | 3.05291 |
| lncRNA47113 | "u" | chr12:7084179-7085366 | 1.64684 | 0.961185 |
| lncRNA10605 | "x" | chr02:40967988-40969129 | 4.81727 | 1.73339 |
| lncRNA14253 | "u" | chr03:11706457-11707624 | 4.22862 | 2.81375 |
| lncRNA15754 | "x" | chr03:61502513-61505162 | 0.317593 | 1.30061 |
| lncRNA20274 | "x" | chr05:4973134-4979848 | 11.2641 | 4.80711 |
| lncRNA28643 | "x" | chr07:64131969-64135830 | 8.58733 | 18.1491 |
| lncRNA44587 | "x" | chr11:53127228-53127912 | 18.5331 | 12.6687 |
| lncRNA48330 | "x" | chr12:64988457-64992828 | 0.826379 | 0.32143 |
| lncRNA46965 | "x" | chr12:4655975-4664687 | 36.4413 | 55.2087 |
| lncRNA37392 | "x" | chr10:1383532-1384559 | 1.93101 | 0.419246 |
| lncRNA10990 | "x" | chr02:44949823-44954729 | 0.359061 | 0.742589 |
| lncRNA19417 | "x" | chr04:58440453-58446202 | 0.567938 | 1.28334 |
| lncRNA45801 | "x" | chr12:45095363-45103315 | 2.07167 | 0.883448 |
| lncRNA19490 | "x" | chr04:59468118-59475483 | 1.45449 | 2.54149 |
| lncRNA30670 | "x" | chr08:2550423-2552065 | 4.981 | 14.7969 |
| lncRNA44821 | "x" | chr12:1633953-1635822 | 1.19226 | 0.570385 |
| lncRNA46975 | "x" | chr12:4840025-4842602 | 2.80311 | 0.522429 |
| lncRNA29683 | "x" | chr07:55830468-55832227 | 1.6653 | 3.28032 |
| lncRNA20348 | "x" | chr05:5997586-5998721 | 3.4909 | 9.68485 |
| lncRNA25014 | "u" | chr06:44666688-44666987 | 76.7181 | 46.7442 |
| lncRNA24418 | "u" | chr06:38565722-38578393 | 0.208873 | 1.84537 |
| lncRNA40907 | "x" | chr10:64295021-64302759 | 3.27438 | 2.18268 |
| lncRNA43411 | "x" | chr11:6470192-6473135 | 3.16217 | 1.74631 |
| lncRNA29972 | "x" | chr07:59985788-59986473 | 5.44278 | 2.04812 |
| lncRNA44745 | "x" | chr12:856650-859108 | 2.7387 | 1.04089 |
| lncRNA34643 | "x" | chr09:41770545-41775897 | 2.07467 | 6.69098 |
| lncRNA32167 | "u" | chr08:234846-235091 | 82.5842 | 38.6279 |
| lncRNA27485 | "u" | chr07:9825619-9828130 | 3.16333 | 4.33881 |
| lncRNA40562 | "u" | chr10:61269858-61270136 | 13.2664 | 30.351 |
| lncRNA29958 | "x" | chr07:59853345-59856910 | 9.69795 | 2.61391 |
| lncRNA06181 | "x" | chr01:83186276-83189882 | 2.86579 | 1.81936 |
| lncRNA02627 | "u" | chr01:75265031-75265348 | 9.81342 | 3.6948 |
| lncRNA01518 | "x" | chr01:11642016-11642875 | 7.06512 | 4.59273 |
| lncRNA03464 | "x" | chr01:84877024-84879874 | 4.96659 | 2.79347 |
| lncRNA10725 | "x" | chr02:42208892-42212859 | 0.349364 | 1.65408 |
| lncRNA25005 | "u" | chr06:44613691-44623029 | 1.27054 | 4.94158 |
| lncRNA38257 | "x" | chr10:51281615-51284677 | 1.33435 | 0.817843 |
| lncRNA19235 | "u" | chr04:55809284-55810071 | 2.55215 | 1.44606 |
| lncRNA41705 | "u" | chr11:10262716-10264133 | 3.20029 | 4.56964 |
| lncRNA08801 | "x" | chr02:46741975-46745553 | 7.69679 | 2.40062 |
| lncRNA45877 | "x" | chr12:47085276-47090460 | 7.70201 | 18.8698 |
| lncRNA43740 | "u" | chr11:23883136-23883782 | 1.51633 | 2.78943 |
| lncRNA03803 | "u" | chr01:88339126-88339440 | 18.21 | 8.59538 |
| lncRNA15827 | "u" | chr03:62288241-62288790 | 2.49147 | 1.17675 |
| lncRNA20700 | "x" | chr05:28060780-28074769 | 24.4592 | 4.61302 |
| lncRNA34629 | "u" | chr09:39123752-39124113 | 8.77257 | 4.13436 |
| lncRNA19932 | "x" | chr05:250070-251114 | 23.2465 | 41.9124 |
| lncRNA15477 | "u" | chr03:58656604-58656829 | 13.5531 | 52.6038 |
| lncRNA27550 | "u" | chr07:17546388-17546657 | 4.00096 | 15.6412 |
| lncRNA13319 | "x" | chr03:60832704-60836089 | 1.38362 | 0.329219 |
| lncRNA08975 | "x" | chr02:48550848-48557510 | 2.2796 | 3.53846 |
| lncRNA16032 | "u" | chr03:64497510-64497721 | 82.1031 | 187.133 |
| lncRNA04264 | "x" | chr01:3821469-3822737 | 9.2182 | 13.9047 |
| lncRNA47708 | "x" | chr12:45731776-45733212 | 1.42757 | 2.71207 |
| lncRNA20206 | "x" | chr05:3904324-3922357 | 5.45559 | 10.3514 |
| lncRNA45508 | "x" | chr12:31282408-31284648 | 1.206 | 0.614244 |
| lncRNA38220 | "x" | chr10:50069604-50078550 | 2.07408 | 3.32397 |
| lncRNA29394 | "u" | chr07:37924903-37930914 | 2.66079 | 3.48382 |
| lncRNA26561 | "x" | chr06:40902228-40906959 | 0.390341 | 1.84699 |
| lncRNA22000 | "u" | chr05:6449650-6449898 | 30.6419 | 9.87021 |
| lncRNA22897 | "x" | chr05:61781894-61783583 | 0.669345 | 2.16761 |
| lncRNA37686 | "x" | chr10:8636004-8651968 | 0.681926 | 0.293327 |
| lncRNA43939 | "x" | chr11:37114907-37116194 | 348.423 | 677.141 |
| lncRNA30825 | "o" | chr08:7632679-7635015 | 7.49591 | 9.83112 |
| lncRNA47296 | "x" | chr12:23997245-24003471 | 1.90349 | 0.675855 |
| lncRNA01928 | "x" | chr01:51951616-51963641 | 0.403233 | 0.775999 |
| lncRNA04596 | "u" | chr01:24908891-24910284 | 0.727631 | 0.398041 |
| lncRNA35866 | "u" | chr09:3828028-3829338 | 0.962956 | 0.526827 |
| lncRNA03950 | "x" | chr01:89998307-90002839 | 1.25445 | 2.06512 |
| lncRNA24351 | "x" | chr06:37754410-37757648 | 0.115135 | 0.787105 |
| lncRNA00006 | "u" | chr00:1150457-1151268 | 0.888537 | 1.73947 |
| lncRNA07161 | "u" | chr02:20434352-20434823 | 1.74987 | 3.65596 |
| lncRNA00011 | "u" | chr00:1243677-1244529 | 0.498496 | 1.03598 |
| lncRNA38945 | "x" | chr10:63675324-63676722 | 0.761647 | 1.78976 |
| lncRNA21392 | "u" | chr05:63562087-63562875 | 2.42203 | 1.34803 |
| lncRNA31936 | "x" | chr08:60850337-60853084 | 1.24088 | 0.267651 |
| lncRNA18709 | "x" | chr04:30813138-30815853 | 4.90616 | 3.64094 |
| lncRNA02056 | "x" | chr01:62402291-62404637 | 1.60793 | 0.833175 |
| lncRNA07770 | "x" | chr02:35706525-35712769 | 0.812157 | 2.24218 |
| lncRNA33459 | "x" | chr08:58386449-58388993 | 2.22991 | 1.06195 |
| lncRNA38294 | "u" | chr10:52139092-52140127 | 0.664465 | 1.25407 |
| lncRNA05391 | "u" | chr01:73604528-73607568 | 0.967252 | 0.622913 |
| lncRNA09359 | "x" | chr02:17755465-17761083 | 1.49143 | 2.20895 |
| lncRNA30804 | "x" | chr08:6916597-6919914 | 0.307654 | 0.737682 |
| lncRNA28957 | "u" | chr07:2408959-2410294 | 3.49028 | 2.39182 |
| lncRNA18987 | "x" | chr04:50314334-50320348 | 2.8272 | 1.60282 |
| lncRNA27835 | "x" | chr07:52128502-52130786 | 1.75761 | 0.521971 |
| lncRNA23357 | "u" | chr06:2627612-2628324 | 1.44275 | 2.64111 |
| lncRNA22472 | "u" | chr05:36045688-36046383 | 2.62484 | 4.30978 |
| lncRNA33605 | "x" | chr08:60284205-60287329 | 0.411554 | 0.775837 |
| lncRNA29114 | "x" | chr07:6483973-6485524 | 4.6163 | 1.87599 |
| lncRNA21950 | "x" | chr05:5878698-5881106 | 1.00908 | 0.218891 |
| lncRNA02994 | "u" | chr01:79931734-79933231 | 2.16151 | 1.40237 |
| lncRNA02839 | "u" | chr01:78181058-78182524 | 0.873402 | 1.43219 |
| lncRNA13148 | "x" | chr03:59054787-59060217 | 1.81847 | 0.338915 |
| lncRNA17398 | "x" | chr04:57867744-57869335 | 2.12066 | 4.83817 |
| lncRNA30695 | "x" | chr08:2859171-2859538 | 388.45 | 221.249 |
| lncRNA34117 | "x" | chr09:3372430-3379841 | 11.5632 | 7.54231 |
| lncRNA04605 | "u" | chr01:25792490-25794851 | 0.953473 | 0.603217 |
| lncRNA34703 | "u" | chr09:47696602-47699294 | 2.76798 | 1.98474 |
| lncRNA33760 | "x" | chr08:62154055-62154746 | 22.9532 | 71.0149 |
| lncRNA41919 | "x" | chr11:22387259-22391128 | 7.64381 | 9.84513 |
| lncRNA39888 | "u" | chr10:42359077-42360946 | 1.04501 | 0.627969 |
| lncRNA45137 | "x" | chr12:4869531-4874487 | 10.4343 | 14.7712 |
| lncRNA25206 | "x" | chr06:882632-883410 | 6.09508 | 9.83989 |
| lncRNA37102 | "x" | chr09:65547716-65548317 | 2.91803 | 4.95004 |
| lncRNA01130 | "x" | chr01:505426-506960 | 32.7052 | 7.01967 |
| lncRNA17541 | "x" | chr04:59836298-59839484 | 1.076 | 8.16463 |
| lncRNA39720 | "u" | chr10:27380672-27381796 | 2.80205 | 4.15635 |
| lncRNA10740 | "x" | chr02:42417192-42419603 | 2.71435 | 1.35963 |
| lncRNA47135 | "u" | chr12:9008099-9009953 | 1.44667 | 2.14853 |
| lncRNA08198 | "x" | chr02:40446911-40449212 | 26.86 | 10.1738 |
| lncRNA41264 | "u" | chr11:2794869-2795696 | 1.02808 | 1.86495 |
| lncRNA40762 | "u" | chr10:63042070-63043286 | 0.896851 | 0.463088 |
| lncRNA11052 | "u" | chr02:45443204-45444681 | 0.839123 | 1.37266 |
| lncRNA09297 | "x" | chr02:15085318-15087946 | 1.98755 | 2.84442 |
| lncRNA13038 | "u" | chr03:57777796-57778080 | 47.2452 | 24.4877 |
| lncRNA37624 | "x" | chr10:5095099-5108835 | 0.692522 | 1.47072 |
| lncRNA47563 | "x" | chr12:42571957-42580423 | 1.13434 | 0.846004 |
| lncRNA25907 | "u" | chr06:32477106-32478294 | 2.20375 | 1.38706 |
| lncRNA26498 | "x" | chr06:40201524-40202219 | 5.15879 | 9.77222 |
| lncRNA45889 | "x" | chr12:47277143-47281131 | 1.12607 | 1.99459 |
| lncRNA43715 | "u" | chr11:21613873-21614688 | 1.67657 | 0.869623 |
| lncRNA28099 | "x" | chr07:57398396-57402361 | 1.27702 | 2.57152 |
| lncRNA21712 | "x" | chr05:2448149-2453637 | 1.17439 | 0.28518 |
| lncRNA43384 | "x" | chr11:5961439-5965936 | 25.4677 | 8.85886 |
| lncRNA29889 | "x" | chr07:59046951-59058126 | 6.21883 | 4.88563 |
| lncRNA03764 | "x" | chr01:87837114-87862936 | 0.508293 | 1.7134 |
| lncRNA41620 | "x" | chr11:8158146-8162027 | 1.94429 | 2.85919 |
| lncRNA17225 | "x" | chr04:55000261-55004007 | 6.66964 | 9.32148 |
| lncRNA25320 | "x" | chr06:2158309-2160810 | 0.197018 | 0.851998 |
| lncRNA43426 | "x" | chr11:7140519-7146547 | 2.39517 | 5.75102 |
| lncRNA23803 | "x" | chr06:28865345-28867783 | 0.489264 | 0.953148 |
| lncRNA17435 | "x" | chr04:58374113-58378144 | 1.16044 | 2.6287 |
| lncRNA04727 | "x" | chr01:41404291-41406583 | 12.6836 | 16.7583 |
| lncRNA28858 | "x" | chr07:1069323-1072745 | 4.52821 | 25.1633 |
| lncRNA25664 | "x" | chr06:23122757-23125806 | 2.13347 | 1.48143 |
| lncRNA03174 | "x" | chr01:81708778-81716769 | 6.07731 | 9.1435 |
| lncRNA07043 | "x" | chr02:15866021-15868254 | 1.91962 | 3.22317 |
| lncRNA02566 | "x" | chr01:74440935-74443546 | 4.2274 | 0.680717 |
| lncRNA26316 | "x" | chr06:38065875-38067512 | 7.41339 | 15.7773 |
| lncRNA08279 | "x" | chr02:41291405-41294442 | 1.11647 | 0.361102 |
| lncRNA20783 | "u" | chr05:36206811-36207286 | 3.64912 | 6.53594 |
| lncRNA25639 | "x" | chr06:22246751-22247890 | 2.63125 | 5.99022 |
| lncRNA27090 | "x" | chr07:330821-334735 | 1.31039 | 0.766233 |
| lncRNA11997 | "u" | chr03:12066668-12069634 | 0.984719 | 0.649631 |
| lncRNA45839 | "x" | chr12:46073128-46073982 | 46.2489 | 120.654 |
| lncRNA12215 | "x" | chr03:27437411-27441368 | 0.819579 | 1.35582 |
| lncRNA38016 | "x" | chr10:39135092-39135965 | 5.3618 | 9.6408 |
| lncRNA31185 | "x" | chr08:48171242-48173768 | 2.80617 | 1.95338 |
| lncRNA30635 | "x" | chr08:2125106-2129164 | 0.680813 | 1.74214 |
| lncRNA44423 | "x" | chr11:51534464-51544612 | 0.798573 | 0.20718 |
| lncRNA44744 | "u" | chr12:851996-852549 | 45.8165 | 34.4179 |
| lncRNA31847 | "x" | chr08:59989065-59989747 | 9.46822 | 15.4218 |
| lncRNA46259 | "x" | chr12:63494822-63498391 | 2.33064 | 3.43412 |
| lncRNA40784 | "x" | chr10:63212065-63219976 | 1.45142 | 0.461529 |
| lncRNA27617 | "u" | chr07:26958104-26959185 | 4.65654 | 6.55789 |
| lncRNA13494 | "x" | chr03:62704263-62718637 | 5.16929 | 1.86992 |
| lncRNA25561 | "x" | chr06:19972668-19975694 | 2.69651 | 3.63328 |
| lncRNA00275 | "u" | chr00:13529508-13530883 | 5.98034 | 8.12505 |
| lncRNA46173 | "x" | chr12:62565936-62571104 | 0.328631 | 0.744976 |
| lncRNA22157 | "x" | chr05:10464346-10467535 | 0.645564 | 0.955756 |
| lncRNA04595 | "x" | chr01:24903492-24905004 | 1.28318 | 1.9507 |
| lncRNA10441 | "x" | chr02:39084531-39087315 | 0.739478 | 0.192251 |
| lncRNA43344 | "x" | chr11:5423278-5424069 | 27.2975 | 49.9833 |
| lncRNA26796 | "u" | chr06:43413725-43414843 | 1.78854 | 1.07864 |
| lncRNA14678 | "o" | chr03:42527782-42530606 | 4.07128 | 5.33459 |
| lncRNA32792 | "x" | chr08:40721913-40723941 | 1.20453 | 1.84496 |
| lncRNA21492 | "x" | chr05:64568905-64574489 | 4.11555 | 7.71888 |
| lncRNA06956 | "x" | chr02:11962645-11964764 | 1.81115 | 3.05315 |
| lncRNA30094 | "u" | chr07:61268158-61269292 | 0.757064 | 1.34554 |
| lncRNA00433 | "x" | chr00:18004719-18005626 | 1.84469 | 3.28102 |
| lncRNA40135 | "x" | chr10:52150377-52151333 | 1.32495 | 2.1738 |
| lncRNA46619 | "x" | chr12:871640-874064 | 3.73114 | 1.48164 |
| lncRNA13809 | "x" | chr03:1219447-1221149 | 5.54891 | 3.36054 |
| lncRNA16320 | "x" | chr04:3090704-3100196 | 3.47867 | 1.54765 |
| lncRNA27719 | "x" | chr07:42662204-42667542 | 1.35014 | 3.70971 |
| lncRNA07446 | "x" | chr02:31538848-31539321 | 5.12435 | 8.4683 |
| lncRNA11722 | "x" | chr03:6818990-6825581 | 1.44418 | 3.16794 |
| lncRNA08626 | "x" | chr02:44944624-44947657 | 1.59178 | 1.03051 |
| lncRNA12448 | "x" | chr03:45742856-45743649 | 1.95794 | 3.29442 |
| lncRNA15952 | "x" | chr03:63621076-63623684 | 5.85145 | 3.80045 |
| lncRNA38864 | "x" | chr10:62989359-62990917 | 44.9722 | 96.9863 |
| lncRNA25012 | "x" | chr06:44653365-44654794 | 14.2809 | 29.2926 |
| lncRNA34180 | "x" | chr09:4049457-4051699 | 3.26874 | 5.01962 |
| lncRNA41431 | "x" | chr11:4801540-4806446 | 1.71047 | 2.91883 |
| lncRNA27626 | "x" | chr07:28717268-28723257 | 2.05534 | 1.55978 |
| lncRNA09298 | "x" | chr02:15092783-15093784 | 0.896277 | 1.74313 |
| lncRNA43577 | "x" | chr11:12992122-12997361 | 15.4245 | 11.6378 |
| lncRNA48141 | "x" | chr12:63449073-63450758 | 3.79872 | 8.25664 |
| lncRNA43050 | "x" | chr11:2083218-2083880 | 28.8206 | 19.9243 |
| lncRNA45063 | "x" | chr12:3989259-3990836 | 1.93433 | 1.25874 |
| lncRNA16584 | "u" | chr04:8175014-8178056 | 0.711787 | 0.461278 |
| lncRNA00624 | "u" | chr00:8779116-8780600 | 1.03278 | 1.62181 |
| lncRNA19337 | "x" | chr04:57367549-57370526 | 0.58523 | 1.51102 |
| lncRNA46591 | "x" | chr12:608256-613819 | 3.89314 | 8.36571 |
| lncRNA05900 | "x" | chr01:80205746-80209405 | 0.965701 | 0.626666 |
| lncRNA34499 | "u" | chr09:19805199-19806442 | 1.54665 | 2.37848 |
| lncRNA39078 | "x" | chr10:64739919-64743646 | 0.989539 | 1.79363 |
| lncRNA32080 | "x" | chr08:62494710-62498149 | 3.77674 | 10.0119 |
| lncRNA48326 | "x" | chr12:64963130-64965570 | 8.45144 | 11.7937 |
| lncRNA29567 | "u" | chr07:52798450-52799071 | 29.2691 | 38.5872 |
| lncRNA13196 | "x" | chr03:59551958-59554223 | 1.98977 | 3.41977 |
| lncRNA06271 | "x" | chr01:84314300-84318754 | 1.9445 | 3.1838 |
| lncRNA19625 | "u" | chr04:61260434-61260925 | 25.07 | 17.7363 |
| lncRNA39331 | "u" | chr10:2749635-2750127 | 25.314 | 34.9943 |
| lncRNA27123 | "u" | chr07:785466-786309 | 5.1746 | 7.35347 |
| lncRNA08516 | "x" | chr02:43863584-43867883 | 1.06209 | 0.58036 |
| lncRNA45975 | "x" | chr12:48459071-48460603 | 5.02714 | 7.71809 |
| lncRNA09796 | "x" | chr02:31907834-31911447 | 0.918413 | 0.297421 |
| lncRNA33498 | "x" | chr08:59006971-59008449 | 3.65163 | 1.00467 |
| lncRNA00095 | "u" | chr00:8840925-8842839 | 1.29879 | 0.841191 |
| lncRNA45150 | "u" | chr12:5020116-5020786 | 11.3927 | 15.6895 |
| lncRNA13260 | "x" | chr03:60295718-60304075 | 1.96194 | 0.927142 |
| lncRNA43041 | "x" | chr11:1970356-1975062 | 1.28679 | 0.843148 |
| lncRNA28020 | "x" | chr07:55980822-55981717 | 6.80868 | 17.1952 |
| lncRNA23181 | "x" | chr06:23848-26003 | 5.1647 | 7.3794 |
| lncRNA01215 | "x" | chr01:1839868-1842652 | 0.933801 | 1.54898 |
| lncRNA18160 | "x" | chr04:2827527-2834470 | 4.61623 | 2.84517 |
| lncRNA36888 | "x" | chr09:62771856-62772853 | 10.3477 | 6.83212 |
| lncRNA15914 | "x" | chr03:63268131-63273261 | 0.303071 | 2.49555 |
| lncRNA10447 | "x" | chr02:39178656-39181039 | 1.3155 | 0.683415 |
| lncRNA37106 | "u" | chr09:65576957-65581797 | 10.067 | 7.14486 |
| lncRNA10467 | "u" | chr02:39412428-39414539 | 0.71901 | 1.12061 |
| lncRNA44718 | "x" | chr12:681861-683498 | 20.3416 | 28.292 |
| lncRNA38422 | "x" | chr10:58197644-58199438 | 1.83633 | 1.16666 |
| lncRNA31053 | "x" | chr08:39566684-39572160 | 6.48608 | 3.64383 |
| lncRNA42705 | "x" | chr11:52234619-52235549 | 1.67162 | 0.830175 |
| lncRNA26290 | "u" | chr06:37775313-37775558 | 209.637 | 128.621 |
| lncRNA43034 | "x" | chr11:1882648-1885109 | 28.9827 | 55.46 |
| lncRNA30416 | "x" | chr07:64821768-64828228 | 2.62797 | 1.51737 |
| lncRNA31991 | "x" | chr08:61596203-61596843 | 2.91591 | 15.2125 |
| lncRNA28811 | "x" | chr07:561664-569230 | 6.64573 | 10.6184 |
| lncRNA19033 | "x" | chr04:51757495-51766115 | 1.70984 | 1.01534 |
| lncRNA08949 | "x" | chr02:48291399-48293973 | 2.99203 | 7.73472 |
| lncRNA43254 | "x" | chr11:4568210-4570780 | 3.85527 | 5.13012 |
| lncRNA34025 | "x" | chr09:2019875-2027165 | 1.91266 | 0.455822 |
| lncRNA14748 | "u" | chr03:45657120-45668274 | 0.37062 | 1.43867 |
| lncRNA22777 | "x" | chr05:59857179-59859222 | 7.98656 | 17.7135 |
| lncRNA01461 | "x" | chr01:6454333-6465847 | 0.708437 | 0.286178 |
| lncRNA40012 | "x" | chr10:48291460-48293593 | 9.57088 | 17.2127 |
| lncRNA35989 | "x" | chr09:5772956-5774563 | 39.3089 | 102.436 |
| lncRNA09722 | "x" | chr02:30708571-30708945 | 4.10409 | 1.06063 |
| lncRNA16193 | "u" | chr04:1754039-1754970 | 5.20545 | 3.63666 |
| lncRNA43973 | "x" | chr11:40366302-40371128 | 2.91954 | 1.91494 |
| lncRNA33062 | "x" | chr08:52731667-52737656 | 1.73311 | 1.11735 |
| lncRNA36044 | "x" | chr09:7854111-7856100 | 9.05905 | 18.0324 |
| lncRNA27855 | "x" | chr07:52755628-52756451 | 1.74363 | 0.569042 |
| lncRNA40239 | "x" | chr10:57679787-57683287 | 6.05169 | 2.24631 |
| lncRNA04633 | "u" | chr01:29362160-29364210 | 1.67206 | 1.15327 |
| lncRNA09747 | "x" | chr02:31167570-31168339 | 2.3235 | 4.24861 |
| lncRNA16143 | "x" | chr04:1078961-1081158 | 1.45055 | 3.28742 |
| lncRNA16260 | "x" | chr04:2582021-2584296 | 3.8834 | 2.35634 |
| lncRNA16974 | "x" | chr04:49314356-49317471 | 2.00114 | 2.69529 |
| lncRNA23286 | "x" | chr06:1599867-1603303 | 1.05396 | 2.73692 |
| lncRNA26448 | "u" | chr06:39536893-39538145 | 121.951 | 96.8901 |
| lncRNA28277 | "x" | chr07:60113502-60114206 | 2.52518 | 4.04886 |
| lncRNA29651 | "u" | chr07:55231106-55232622 | 11.1253 | 6.45753 |
| lncRNA38092 | "u" | chr10:43283109-43284675 | 0.801692 | 0.464704 |
| lncRNA17154 | "u" | chr04:54096011-54097974 | 0.891951 | 0.55601 |
| lncRNA48335 | "x" | chr12:65017074-65019950 | 5.46125 | 7.02473 |
| lncRNA21285 | "x" | chr05:62122564-62126690 | 1.0355 | 1.9729 |
| lncRNA37457 | "u" | chr10:2215169-2218476 | 0.836056 | 1.19558 |
| lncRNA03520 | "x" | chr01:85464805-85470238 | 1.01661 | 1.86366 |
| lncRNA30664 | "x" | chr08:2492701-2500799 | 0.721542 | 0.344339 |
| lncRNA37571 | "x" | chr10:3624897-3635076 | 2.77271 | 1.3364 |
| lncRNA38298 | "u" | chr10:52270729-52273807 | 0.660982 | 0.975835 |
| lncRNA40944 | "x" | chr10:64555370-64560375 | 16.0522 | 24.3045 |
| lncRNA20463 | "x" | chr05:8400259-8401305 | 1.23823 | 4.37204 |
| lncRNA45012 | "x" | chr12:3461989-3464511 | 0.791959 | 1.40825 |
| lncRNA14645 | "x" | chr03:41249465-41255903 | 12.1304 | 15.2305 |
| lncRNA37682 | "x" | chr10:7367205-7373994 | 1.71966 | 3.11379 |
| lncRNA22487 | "x" | chr05:37895078-37898659 | 1.98073 | 2.727 |
| lncRNA45905 | "x" | chr12:47449456-47453634 | 2.67762 | 0.866318 |
| lncRNA27376 | "x" | chr07:4982954-4983585 | 5.86311 | 1.55099 |
| lncRNA02692 | "x" | chr01:76141601-76142995 | 0.315384 | 1.12766 |
| lncRNA11198 | "u" | chr02:47021632-47022101 | 4.48174 | 2.26037 |
| lncRNA26551 | "u" | chr06:40808073-40808511 | 5.15623 | 2.58727 |
| lncRNA37220 | "u" | chr09:66993651-66994398 | 1.77745 | 0.891045 |
| lncRNA31612 | "x" | chr08:57011770-57015543 | 3.72887 | 2.56346 |
| lncRNA15831 | "u" | chr03:62325861-62330270 | 1.35617 | 0.445253 |
| lncRNA38608 | "x" | chr10:60427111-60429006 | 3.16377 | 1.51847 |
| lncRNA44439 | "x" | chr11:51683589-51685539 | 1.08794 | 2.63378 |
| lncRNA22453 | "u" | chr05:33081895-33082730 | 2.34883 | 3.63085 |
| lncRNA28060 | "u" | chr07:56830676-56831046 | 56.8763 | 78.3121 |
| lncRNA04882 | "u" | chr01:58552341-58552659 | 2.93002 | 7.67949 |
| lncRNA16150 | "u" | chr04:1169824-1170200 | 1.47919 | 3.87994 |
| lncRNA18568 | "u" | chr04:19253549-19255096 | 2.45654 | 3.43357 |
| lncRNA22805 | "x" | chr05:60312325-60319582 | 0.264613 | 0.799289 |
| lncRNA28008 | "x" | chr07:55765794-55769600 | 2.36783 | 3.55342 |
| lncRNA01110 | "x" | chr01:400721-403120 | 30.7654 | 99.54 |
| lncRNA13512 | "x" | chr03:62897652-62903551 | 6.91064 | 4.33233 |
| lncRNA33180 | "u" | chr08:54698350-54698757 | 0.984977 | 2.56063 |
| lncRNA37507 | "x" | chr10:2775630-2779504 | 0.951213 | 0.396556 |
| lncRNA35931 | "x" | chr09:4793544-4796953 | 1.43746 | 2.35213 |
| lncRNA13935 | "x" | chr03:6644685-6645675 | 3.49625 | 2.34925 |
| lncRNA35809 | "x" | chr09:3123570-3124309 | 9.05493 | 2.54631 |
| lncRNA01424 | "x" | chr01:5195719-5200917 | 3.38715 | 1.05157 |
| lncRNA15194 | "x" | chr03:54407059-54408908 | 1.11348 | 3.22751 |
| lncRNA16427 | "x" | chr04:4624986-4631132 | 1.86927 | 0.97104 |
| lncRNA23365 | "x" | chr06:2751016-2756245 | 3.25339 | 8.15495 |
| lncRNA13836 | "x" | chr03:1778820-1782208 | 3.26911 | 5.40097 |
| lncRNA05088 | "x" | chr01:68009110-68023501 | 1.37673 | 0.883889 |
| lncRNA46523 | "x" | chr12:91809-95281 | 2.12324 | 1.32627 |
| lncRNA17152 | "x" | chr04:54089583-54093373 | 1.01672 | 2.47937 |
| lncRNA47829 | "x" | chr12:47861645-47865470 | 4.17554 | 6.10633 |
| lncRNA38607 | "x" | chr10:60427111-60429006 | 12.3741 | 5.18608 |
| lncRNA23669 | "u" | chr06:21793327-21795050 | 0.700778 | 1.12168 |
| lncRNA40490 | "x" | chr10:60635456-60637850 | 95.2777 | 134.17 |
| lncRNA20325 | "o" | chr05:5799500-5800416 | 27.7846 | 57.9875 |
| lncRNA31613 | "x" | chr08:57030705-57032365 | 12.0453 | 15.3714 |
| lncRNA22486 | "x" | chr05:37895078-37898659 | 1.03344 | 1.72552 |
| lncRNA43380 | "x" | chr11:5934666-5948813 | 1.28256 | 0.814797 |
| lncRNA12176 | "x" | chr03:24372662-24390055 | 0.622723 | 1.46197 |
| lncRNA23008 | "x" | chr05:63243124-63247100 | 7.37424 | 3.59253 |
| lncRNA28144 | "u" | chr07:58323858-58324397 | 1.65093 | 3.10186 |
| lncRNA21984 | "x" | chr05:6275062-6278128 | 4.28057 | 2.20859 |
| lncRNA34354 | "x" | chr09:8937656-8943475 | 6.2694 | 3.77665 |
| lncRNA02104 | "u" | chr01:65208488-65212422 | 2.13575 | 1.58832 |
| lncRNA31454 | "x" | chr08:54471768-54475227 | 1.45046 | 0.314498 |
| lncRNA28524 | "x" | chr07:62995344-63002900 | 7.75644 | 15.8477 |
| lncRNA06649 | "x" | chr01:88381000-88383049 | 1.69834 | 3.05647 |
| lncRNA26784 | "x" | chr06:43293637-43294422 | 4.19247 | 2.73162 |
| lncRNA34189 | "x" | chr09:4148229-4149616 | 15.1451 | 34.327 |
| lncRNA04496 | "u" | chr01:16079296-16085363 | 1.67213 | 1.27288 |
| lncRNA22599 | "x" | chr05:52917390-52924690 | 1.61089 | 0.390104 |
| lncRNA32175 | "x" | chr08:331750-333165 | 43.1245 | 65.832 |
| lncRNA23231 | "x" | chr06:785188-789735 | 0.827766 | 4.20017 |
| lncRNA37812 | "u" | chr10:21044741-21078139 | 2.74442 | 1.80522 |
| lncRNA21654 | "u" | chr05:1637377-1637819 | 7.15117 | 4.02822 |
| lncRNA41042 | "u" | chr11:656912-659920 | 0.747119 | 0.49807 |
| lncRNA09519 | "u" | chr02:24943050-24944442 | 0.851288 | 0.489977 |
| lncRNA07604 | "x" | chr02:33691122-33692429 | 3.36517 | 1.54559 |
| lncRNA41303 | "x" | chr11:3254830-3262718 | 1.07941 | 2.49866 |
| lncRNA18593 | "u" | chr04:21810380-21812300 | 0.501021 | 0.812022 |
| lncRNA20478 | "u" | chr05:9427740-9428428 | 2.33133 | 3.78319 |
| lncRNA38685 | "x" | chr10:61096786-61099234 | 26.0055 | 8.4054 |
| lncRNA02300 | "u" | chr01:70583350-70585608 | 1.9633 | 1.39317 |
| lncRNA40491 | "x" | chr10:60639506-60641635 | 2.60363 | 1.83864 |
| lncRNA05133 | "x" | chr01:69442213-69447222 | 1.54845 | 2.65588 |
| lncRNA09251 | "u" | chr02:12882495-12883601 | 1.10797 | 0.640379 |
| lncRNA38725 | "x" | chr10:61515216-61520273 | 3.94309 | 8.13848 |
| lncRNA35130 | "u" | chr09:62487355-62488613 | 1.37481 | 0.851508 |
| lncRNA21405 | "u" | chr05:63699989-63700567 | 9.67313 | 6.6405 |
| lncRNA44141 | "u" | chr11:47703101-47703363 | 150.121 | 94.5438 |
| lncRNA09995 | "x" | chr02:34159275-34162937 | 0.769303 | 0.272162 |
| lncRNA19650 | "x" | chr04:61492327-61492752 | 9.21004 | 15.8359 |
| lncRNA09037 | "u" | chr02:49159239-49159677 | 6.5623 | 3.72774 |
| lncRNA15469 | "u" | chr03:58602638-58603466 | 3.56178 | 5.18422 |
| lncRNA23038 | "x" | chr05:63632747-63633211 | 5.97976 | 3.17427 |
| lncRNA44297 | "x" | chr11:50079732-50085326 | 3.32524 | 2.20199 |
| lncRNA36605 | "x" | chr09:57355328-57358269 | 8.97093 | 14.7101 |
| lncRNA41509 | "x" | chr11:5682713-5696581 | 2.67811 | 1.79324 |
| lncRNA05970 | "x" | chr01:80943684-80950471 | 4.02624 | 7.21899 |
| lncRNA34119 | "x" | chr09:3396761-3398827 | 14.2001 | 9.48596 |
| lncRNA48135 | "x" | chr12:63414201-63415500 | 1.41117 | 3.17679 |
| lncRNA48155 | "u" | chr12:63524419-63525593 | 1.6354 | 1.01799 |
| lncRNA06858 | "x" | chr02:2082680-2086381 | 1.42118 | 1.04317 |
| lncRNA18175 | "x" | chr04:3007659-3012779 | 12.2003 | 7.63621 |
| lncRNA29457 | "u" | chr07:45103368-45105371 | 1.09815 | 1.5998 |
| lncRNA01343 | "x" | chr01:3075588-3080069 | 0.928288 | 0.462859 |
| lncRNA28291 | "x" | chr07:60247591-60250658 | 2.38472 | 0.984059 |
| lncRNA00639 | "u" | chr00:9795292-9833981 | 146.612 | 542.546 |
| lncRNA36950 | "x" | chr09:63534871-63547345 | 0.598601 | 0.129008 |
| lncRNA45964 | "x" | chr12:48320032-48324702 | 2.63875 | 2.00292 |
| lncRNA30676 | "x" | chr08:2645180-2649458 | 1.00658 | 0.42346 |
| lncRNA46928 | "x" | chr12:4241294-4242428 | 13.1941 | 16.8887 |
| lncRNA39363 | "x" | chr10:3168004-3173396 | 0.198624 | 0.780357 |
| lncRNA20003 | "x" | chr05:1078397-1081504 | 2.435 | 1.50435 |
| lncRNA46702 | "x" | chr12:1883667-1886869 | 1.12577 | 1.7526 |
| lncRNA47448 | "u" | chr12:35726210-35729786 | 2.40926 | 3.16098 |
| lncRNA42934 | "x" | chr11:1156917-1159917 | 13.5588 | 5.01931 |
| lncRNA44174 | "x" | chr11:48467534-48471289 | 1.89212 | 3.89656 |
| lncRNA11009 | "x" | chr02:45113498-45122929 | 0.941153 | 1.2548 |
| lncRNA40401 | "x" | chr10:59788054-59791549 | 8.04968 | 2.45165 |
| lncRNA14904 | "x" | chr03:48303255-48310800 | 0.840709 | 1.59572 |
| lncRNA38210 | "u" | chr10:49742299-49744086 | 0.769389 | 1.19643 |
| lncRNA19829 | "x" | chr04:63347130-63347776 | 14.6077 | 25.2514 |
| lncRNA04012 | "x" | chr01:574915-576400 | 3.89128 | 2.30143 |
| lncRNA21412 | "x" | chr05:63770697-63772898 | 7.18714 | 12.9415 |
| lncRNA39996 | "x" | chr10:47277390-47291255 | 0.676074 | 0.94527 |
| lncRNA17490 | "x" | chr04:59092280-59095192 | 1.11276 | 2.56384 |
| lncRNA01566 | "x" | chr01:15577504-15583605 | 11.1925 | 5.38474 |
| lncRNA48387 | "x" | chr12:65395457-65407186 | 0.474343 | 1.22594 |
| lncRNA31086 | "x" | chr08:43354427-43361015 | 1.4568 | 2.86174 |
| lncRNA10165 | "x" | chr02:36004960-36006990 | 2.76648 | 4.70328 |
| lncRNA48091 | "x" | chr12:62957018-62958198 | 5.77569 | 9.22654 |
| lncRNA09889 | "x" | chr02:32919196-32922504 | 0.705155 | 2.38403 |
| lncRNA39174 | "x" | chr10:988429-990781 | 3.20775 | 2.02122 |
| lncRNA14976 | "u" | chr03:49903137-49904473 | 1.02002 | 1.57887 |
| lncRNA38410 | "x" | chr10:58018489-58022891 | 2.01509 | 1.48628 |
| lncRNA20637 | "x" | chr05:22898837-22900552 | 2.64129 | 4.8541 |
| lncRNA00053 | "u" | chr00:6549191-6550008 | 0.849837 | 1.5193 |
| lncRNA17314 | "u" | chr04:56576086-56576294 | 169.077 | 302.342 |
| lncRNA30768 | "u" | chr08:4447975-4452766 | 0.945843 | 0.68619 |
| lncRNA41780 | "x" | chr11:13394658-13395069 | 33.1917 | 71.1482 |
| lncRNA09199 | "x" | chr02:8085545-8097243 | 0.926971 | 0.184684 |
| lncRNA08004 | "u" | chr02:38418724-38419208 | 1.82093 | 0.790447 |
| lncRNA13677 | "x" | chr03:64565198-64568771 | 3.60794 | 8.07987 |
| lncRNA01579 | "u" | chr01:16808338-16810747 | 4.58532 | 5.92856 |
| lncRNA43247 | "x" | chr11:4515207-4519732 | 5.01596 | 2.57481 |
| lncRNA14728 | "u" | chr03:45329447-45330348 | 0.745062 | 1.32948 |
| lncRNA28457 | "u" | chr07:62261928-62262198 | 32.5709 | 57.9459 |
| lncRNA42626 | "x" | chr11:51545012-51552055 | 7.94516 | 3.429 |
| lncRNA02800 | "x" | chr01:77697059-77698757 | 0.656053 | 1.53907 |
| lncRNA01546 | "x" | chr01:13959369-13962350 | 6.16733 | 2.76106 |
| lncRNA42757 | "x" | chr11:52697579-52701602 | 2.71781 | 1.30151 |
| lncRNA41242 | "x" | chr11:2579285-2581469 | 28.226 | 66.0662 |
| lncRNA30298 | "x" | chr07:63632749-63636089 | 0.984783 | 0.670001 |
| lncRNA33807 | "x" | chr08:62705442-62711140 | 4.99982 | 11.7786 |
| lncRNA41585 | "x" | chr11:7263654-7267098 | 0.844306 | 0.568737 |
| lncRNA44774 | "x" | chr12:1172165-1176511 | 0.837578 | 0.42697 |
| lncRNA11904 | "u" | chr03:9613303-9616795 | 0.805815 | 1.14089 |
| lncRNA24659 | "x" | chr06:41074562-41077043 | 0.404945 | 0.831138 |
| lncRNA10300 | "x" | chr02:37546658-37553218 | 7.30386 | 4.04021 |
| lncRNA23501 | "x" | chr06:8117226-8119729 | 1.50374 | 2.23967 |
| lncRNA44304 | "x" | chr11:50191389-50195261 | 9.58171 | 3.12387 |
| lncRNA47362 | "u" | chr12:29743588-29745762 | 0.60029 | 0.926528 |
| lncRNA14276 | "x" | chr03:12455770-12457761 | 6.84228 | 14.3839 |
| lncRNA23129 | "u" | chr05:64519416-64520311 | 4.04449 | 2.74909 |
| lncRNA03488 | "x" | chr01:85089605-85092408 | 5.54541 | 1.42951 |
| lncRNA08846 | "u" | chr02:47225238-47225754 | 14.8228 | 10.4158 |
| lncRNA43650 | "x" | chr11:17049554-17054156 | 18.3709 | 14.6007 |
| lncRNA19928 | "x" | chr05:208669-209765 | 20.6634 | 56.921 |
| lncRNA18567 | "u" | chr04:19251031-19252713 | 3.23634 | 4.36163 |
| lncRNA38461 | "x" | chr10:59007593-59011112 | 1.28988 | 1.87878 |
| lncRNA18286 | "x" | chr04:4521571-4523174 | 1.77528 | 3.22624 |
| lncRNA12344 | "x" | chr03:41323604-41325241 | 1.42195 | 2.04505 |
| lncRNA14817 | "u" | chr03:46515726-46517952 | 0.700611 | 1.04411 |
| lncRNA42859 | "x" | chr11:419066-426076 | 2.39984 | 7.07686 |
| lncRNA19515 | "x" | chr04:59958590-59961594 | 0.627861 | 1.20045 |
| lncRNA21074 | "x" | chr05:58486160-58490924 | 1.44127 | 0.674596 |
| lncRNA11658 | "x" | chr03:2383873-2386244 | 3.91856 | 1.38459 |
| lncRNA27177 | "u" | chr07:1605690-1606080 | 2.02692 | 4.37339 |
| lncRNA07332 | "x" | chr02:29625468-29629342 | 5.51325 | 3.71503 |
| lncRNA12602 | "u" | chr03:48601232-48601876 | 2.20939 | 1.23298 |
| lncRNA37853 | "x" | chr10:23752985-23760474 | 1.18197 | 0.763878 |
| lncRNA23269 | "x" | chr06:1311363-1316191 | 6.3265 | 2.60194 |
| lncRNA40770 | "x" | chr10:63078127-63084428 | 1.36299 | 0.364465 |
| lncRNA10437 | "x" | chr02:39035490-39037002 | 1.26807 | 0.587267 |
| lncRNA06991 | "u" | chr02:13664764-13667712 | 0.900645 | 0.616704 |
| lncRNA12636 | "u" | chr03:49683501-49683805 | 24.4614 | 13.0932 |
| lncRNA02161 | "x" | chr01:67756586-67758593 | 0.868461 | 2.06919 |
| lncRNA44466 | "x" | chr11:51928766-51930063 | 4.06895 | 7.4097 |
| lncRNA03511 | "x" | chr01:85396087-85400651 | 1.01811 | 1.36448 |
| lncRNA00443 | "x" | chr00:18403199-18404342 | 18.9867 | 11.1429 |
| lncRNA41073 | "x" | chr11:924276-930481 | 20.8441 | 7.99426 |
| lncRNA20081 | "x" | chr05:2246192-2252833 | 10.3626 | 15.0535 |
| lncRNA25363 | "x" | chr06:2763580-2770677 | 0.700897 | 0.388641 |
| lncRNA05520 | "x" | chr01:75413934-75416835 | 0.956505 | 0.329486 |
| lncRNA24855 | "x" | chr06:43044323-43044970 | 24.1004 | 44.7089 |
| lncRNA18632 | "x" | chr04:25235417-25242550 | 4.51493 | 2.88259 |
| lncRNA04630 | "x" | chr01:29350550-29356065 | 1.81745 | 1.19305 |
| lncRNA28709 | "x" | chr07:64762081-64762739 | 9.18037 | 26.4444 |
| lncRNA30792 | "x" | chr08:6074039-6078983 | 3.02872 | 5.85996 |
| lncRNA22889 | "x" | chr05:61668008-61674505 | 0.846982 | 1.47997 |
| lncRNA39410 | "x" | chr10:4066957-4068832 | 11.0615 | 6.28434 |
| lncRNA37109 | "x" | chr09:65586857-65589581 | 3.22513 | 0.83433 |
| lncRNA21768 | "x" | chr05:3182172-3185140 | 3.95863 | 13.4834 |
| lncRNA22909 | "x" | chr05:61964748-61965984 | 422.916 | 117.918 |
| lncRNA31664 | "x" | chr08:57709565-57714096 | 2.77975 | 1.47994 |
| lncRNA35581 | "x" | chr09:168623-169638 | 1.50486 | 2.9495 |
| lncRNA15556 | "x" | chr03:59353773-59360741 | 1.92818 | 3.71539 |
| lncRNA33404 | "x" | chr08:57705825-57709166 | 0.658176 | 0.183125 |
| lncRNA39578 | "u" | chr10:13620877-13625459 | 4.02178 | 5.27055 |
| lncRNA12420 | "x" | chr03:45336234-45340788 | 8.13817 | 3.31604 |
| lncRNA39200 | "x" | chr10:1190014-1193506 | 1.99231 | 2.93782 |
| lncRNA13340 | "x" | chr03:61040552-61043249 | 0.724871 | 2.76061 |
| lncRNA36342 | "u" | chr09:40324513-40326437 | 9.17571 | 11.5718 |
| lncRNA03694 | "x" | chr01:87157182-87161875 | 0.656968 | 1.51821 |
| lncRNA43024 | "x" | chr11:1786664-1791043 | 8.84535 | 3.82592 |
| lncRNA07797 | "x" | chr02:35976351-35983439 | 0.420053 | 0.723283 |
| lncRNA30726 | "u" | chr08:3340769-3341743 | 1.23667 | 0.710953 |
| lncRNA32417 | "u" | chr08:3523251-3523963 | 2.08266 | 1.19287 |
| lncRNA18310 | "x" | chr04:4813268-4815928 | 1.52795 | 0.803229 |
| lncRNA26680 | "x" | chr06:42140222-42151673 | 1.03218 | 0.571313 |
| lncRNA17168 | "x" | chr04:54296086-54298871 | 2.03471 | 2.6849 |
| lncRNA36270 | "x" | chr09:30120238-30123287 | 1.60798 | 0.617811 |
| lncRNA21257 | "x" | chr05:61816492-61828800 | 0.614982 | 0.176298 |
| lncRNA38450 | "x" | chr10:58838917-58852333 | 6.74636 | 4.17967 |
| lncRNA10680 | "u" | chr02:41763135-41764236 | 2.40369 | 3.46868 |
| lncRNA47293 | "x" | chr12:23790103-23798010 | 5.78455 | 1.24586 |
| lncRNA10922 | "x" | chr02:44305170-44306332 | 0.631243 | 2.05629 |
| lncRNA08990 | "x" | chr02:48735361-48737955 | 1.31722 | 2.7242 |
| lncRNA18303 | "u" | chr04:4689738-4690473 | 10.3048 | 7.51274 |
| lncRNA30390 | "x" | chr07:64560187-64564962 | 0.868924 | 0.424225 |
| lncRNA24248 | "x" | chr06:36509240-36511531 | 0.608532 | 1.15097 |
| lncRNA41874 | "u" | chr11:18017048-18019385 | 2.12486 | 1.55236 |
| lncRNA29526 | "x" | chr07:51399398-51402969 | 1.60505 | 0.952298 |
| lncRNA30140 | "u" | chr07:62016354-62017173 | 1.2564 | 0.678811 |
| lncRNA12481 | "u" | chr03:46211698-46212583 | 0.320354 | 0.835655 |
| lncRNA25221 | "x" | chr06:1082903-1084179 | 0.936659 | 0.452547 |
| lncRNA07240 | "u" | chr02:25671676-25672696 | 0.886017 | 0.481778 |
| lncRNA18965 | "u" | chr04:49810197-49810940 | 1.43579 | 0.779852 |
| lncRNA27619 | "u" | chr07:27033219-27033688 | 4.18632 | 2.27472 |
| lncRNA30024 | "u" | chr07:60544982-60545499 | 3.30097 | 1.79568 |
| lncRNA11926 | "x" | chr03:10050484-10054603 | 14.9842 | 26.6881 |
| lncRNA12191 | "x" | chr03:25219058-25230016 | 3.43516 | 1.77792 |
| lncRNA41003 | "x" | chr11:269662-272662 | 0.855642 | 3.12399 |
| lncRNA46370 | "x" | chr12:64302381-64314399 | 3.36341 | 1.87556 |
| lncRNA30583 | "x" | chr08:1365017-1372630 | 1.98984 | 1.37976 |
| lncRNA45034 | "x" | chr12:3635211-3635714 | 11.7995 | 46.3901 |
| lncRNA40862 | "x" | chr10:63887617-63889043 | 31.7589 | 16.9819 |
| lncRNA25104 | "x" | chr06:45660098-45662380 | 19.6603 | 10.6218 |
| lncRNA14132 | "x" | chr03:9242800-9245006 | 0.943196 | 0.243634 |
| lncRNA39815 | "u" | chr10:37042667-37043474 | 0.971873 | 1.68205 |
| lncRNA28829 | "u" | chr07:723768-725635 | 43.7211 | 54.9352 |
| lncRNA08489 | "x" | chr02:43553933-43554808 | 53.6487 | 102.607 |
| lncRNA40915 | "x" | chr10:64360213-64367667 | 2.02809 | 4.28132 |
| lncRNA39573 | "x" | chr10:13006048-13008448 | 11.0688 | 13.8032 |
| lncRNA06196 | "x" | chr01:83415884-83422712 | 2.31733 | 1.16616 |
| lncRNA41441 | "x" | chr11:5009015-5013900 | 26.0378 | 54.6496 |
| lncRNA38468 | "x" | chr10:59069860-59075366 | 1.07516 | 2.92657 |
| lncRNA18158 | "x" | chr04:2807611-2815821 | 1.04951 | 0.609224 |
| lncRNA47224 | "x" | chr12:14653911-14655550 | 7.62126 | 10.3657 |
| lncRNA31292 | "x" | chr08:51333632-51335838 | 1.16806 | 0.339452 |
| lncRNA19025 | "x" | chr04:51618047-51623875 | 0.716647 | 0.383803 |
| lncRNA08085 | "x" | chr02:39331908-39332631 | 1.64033 | 0.825511 |
| lncRNA21356 | "x" | chr05:63063216-63065233 | 2.39449 | 6.19799 |
| lncRNA25866 | "x" | chr06:31930213-31946322 | 3.01496 | 1.91033 |
| lncRNA16922 | "u" | chr04:45428797-45433491 | 3.29152 | 4.14549 |
| lncRNA32856 | "x" | chr08:45975800-45982039 | 0.574811 | 1.00781 |
| lncRNA09246 | "u" | chr02:12522784-12523775 | 0.747222 | 1.26413 |
| lncRNA46710 | "x" | chr12:1949736-1951671 | 4.72832 | 13.5682 |
| lncRNA35854 | "u" | chr09:3689915-3693695 | 0.91967 | 1.24544 |
| lncRNA47872 | "x" | chr12:48576307-48577271 | 5.56195 | 8.75813 |
| lncRNA15918 | "u" | chr03:63283544-63295748 | 7.0412 | 10.14 |
| lncRNA35536 | "x" | chr09:67440994-67442749 | 3.83068 | 7.29545 |
| lncRNA41086 | "x" | chr11:1074140-1075765 | 37.1299 | 29.9881 |
| lncRNA35667 | "u" | chr09:1006614-1020453 | 1.21381 | 0.243386 |
| lncRNA43853 | "x" | chr11:35060522-35061378 | 23.3889 | 13.1314 |
| lncRNA44566 | "x" | chr11:52928611-52929242 | 6.375 | 9.24038 |
| lncRNA03132 | "u" | chr01:81360051-81360573 | 2.78963 | 4.66793 |
| lncRNA18193 | "u" | chr04:3185169-3185772 | 44.0349 | 55.8474 |
| lncRNA12736 | "x" | chr03:52639192-52646159 | 5.6297 | 9.90286 |
| lncRNA14538 | "u" | chr03:29883485-29884740 | 3.75724 | 5.0906 |
| lncRNA19931 | "x" | chr05:250070-251114 | 9.76685 | 5.782 |
| lncRNA01088 | "x" | chr01:204616-207451 | 1.26751 | 0.506519 |
| lncRNA30769 | "u" | chr08:4453971-4454658 | 3.72095 | 5.57935 |
| lncRNA28547 | "x" | chr07:63244232-63247487 | 1.03512 | 0.415431 |
| lncRNA11345 | "x" | chr02:48531617-48533667 | 6.50691 | 13.3843 |
| lncRNA27549 | "u" | chr07:17037460-17037966 | 15.2923 | 20.9461 |
| lncRNA03626 | "x" | chr01:86414202-86421830 | 1.07638 | 0.679756 |
| lncRNA11113 | "x" | chr02:46129230-46131184 | 5.27413 | 3.38819 |
| lncRNA07278 | "x" | chr02:28212793-28219060 | 2.14238 | 1.67807 |
| lncRNA20683 | "x" | chr05:28033580-28034766 | 1.21603 | 1.86242 |
| lncRNA07708 | "x" | chr02:34956174-34960025 | 3.59679 | 2.40001 |
| lncRNA33601 | "x" | chr08:60261958-60264602 | 0.729918 | 1.33756 |
| lncRNA12961 | "x" | chr03:56899828-56907767 | 2.66327 | 1.40421 |
| lncRNA37916 | "u" | chr10:30355506-30365319 | 2.21502 | 1.76284 |
| lncRNA25533 | "u" | chr06:15683810-15686061 | 2.4903 | 1.84407 |
| lncRNA08426 | "x" | chr02:42822794-42826264 | 5.39107 | 4.29315 |
| lncRNA16546 | "x" | chr04:6971877-6975822 | 0.28926 | 1.13613 |
| lncRNA17566 | "u" | chr04:60086669-60087484 | 2.36103 | 1.47768 |
| lncRNA15338 | "x" | chr03:57066300-57068725 | 0.755267 | 1.97165 |
| lncRNA23917 | "x" | chr06:31531948-31534505 | 1.20377 | 2.21997 |
| lncRNA30755 | "x" | chr08:3975000-3978331 | 1.35503 | 0.788996 |
| lncRNA24803 | "x" | chr06:42477946-42480623 | 11.6882 | 16.118 |
| lncRNA45036 | "x" | chr12:3644954-3647850 | 4.66701 | 6.73432 |
| lncRNA34590 | "u" | chr09:30460444-30461282 | 16.4827 | 21.2408 |
| lncRNA31231 | "x" | chr08:49588278-49591151 | 7.90426 | 11.4292 |
| lncRNA47630 | "x" | chr12:43815508-43822367 | 75.3186 | 51.7978 |
| lncRNA36744 | "x" | chr09:60505400-60507843 | 1.52354 | 2.3405 |
| lncRNA48317 | "x" | chr12:64885995-64888286 | 3.62512 | 2.42779 |
| lncRNA30470 | "x" | chr08:91609-96770 | 5.27592 | 3.342 |
| lncRNA48195 | "x" | chr12:63821174-63829483 | 0.977192 | 3.1665 |
| lncRNA06536 | "x" | chr01:87178847-87179887 | 4.77012 | 3.34008 |
| lncRNA29973 | "x" | chr07:60012266-60019147 | 1.77683 | 1.27858 |
| lncRNA37521 | "x" | chr10:2924760-2931466 | 0.646975 | 0.394521 |
| lncRNA39932 | "u" | chr10:45401793-45402433 | 2.13742 | 3.44883 |
| lncRNA19177 | "x" | chr04:54843788-54847535 | 27.4805 | 61.6529 |
| lncRNA06074 | "u" | chr01:82084155-82084659 | 3.45391 | 5.54092 |
| lncRNA13647 | "u" | chr03:64325499-64325994 | 3.79992 | 6.09344 |
| lncRNA45298 | "u" | chr12:10198804-10199432 | 2.23698 | 3.59346 |
| lncRNA27603 | "u" | chr07:24670582-24671337 | 2.71085 | 4.03093 |
| lncRNA07200 | "u" | chr02:21868248-21869166 | 1.5293 | 2.37292 |
| lncRNA25499 | "x" | chr06:12748791-12756956 | 1.05257 | 0.570922 |
| lncRNA38073 | "u" | chr10:42473780-42476898 | 0.929933 | 0.404319 |
| lncRNA40948 | "x" | chr10:64607862-64615349 | 1.80105 | 1.07702 |
| lncRNA39300 | "x" | chr10:2340382-2349504 | 5.9335 | 3.97168 |
| lncRNA48294 | "x" | chr12:64677239-64679751 | 26.1197 | 46.8428 |
| lncRNA25069 | "x" | chr06:45217267-45218794 | 3.62756 | 6.93272 |
| lncRNA03016 | "u" | chr01:80136016-80136639 | 7.30616 | 10.2215 |
| lncRNA09597 | "u" | chr02:28420355-28422784 | 1.28837 | 1.77131 |
| lncRNA44220 | "x" | chr11:49167158-49176185 | 1.73864 | 4.50796 |
| lncRNA42514 | "x" | chr11:50268778-50270432 | 6.76673 | 1.94784 |
| lncRNA30819 | "o" | chr08:7417505-7427352 | 17.2026 | 22.2739 |
| lncRNA31644 | "x" | chr08:57411106-57413970 | 1.52372 | 1.01963 |
| lncRNA04367 | "x" | chr01:6866920-6873274 | 0.763478 | 0.56525 |
| lncRNA47314 | "u" | chr12:25893773-25894020 | 119.943 | 185.246 |
| lncRNA08282 | "x" | chr02:41341005-41345754 | 11.5896 | 15.873 |
| lncRNA22088 | "x" | chr05:8322380-8350401 | 0.880857 | 0.704186 |
| lncRNA03152 | "x" | chr01:81507606-81515514 | 1.49835 | 1.02058 |
| lncRNA44795 | "x" | chr12:1338432-1341924 | 6.40019 | 10.7398 |
| lncRNA26341 | "u" | chr06:38319697-38320424 | 5.41851 | 7.51727 |
| lncRNA09429 | "u" | chr02:20068859-20069462 | 6.48074 | 9.26566 |
| lncRNA43371 | "u" | chr11:5807751-5808373 | 20.1527 | 15.2417 |
| lncRNA15171 | "x" | chr03:54043608-54048042 | 0.447245 | 1.10261 |
| lncRNA20797 | "u" | chr05:38722366-38724286 | 0.501673 | 0.775825 |
| lncRNA09631 | "u" | chr02:29074637-29075992 | 0.568406 | 0.925549 |
| lncRNA05680 | "x" | chr01:77543954-77553574 | 3.05317 | 2.25467 |
| lncRNA04187 | "x" | chr01:2485164-2492775 | 1.11762 | 0.642007 |
| lncRNA19498 | "x" | chr04:59664714-59670763 | 0.945188 | 1.89948 |
| lncRNA03632 | "x" | chr01:86482489-86484428 | 0.427421 | 1.38952 |
| lncRNA17567 | "u" | chr04:60108946-60109829 | 3.10326 | 2.07757 |
| lncRNA46152 | "x" | chr12:62408771-62410949 | 15.7095 | 10.1514 |
| lncRNA21091 | "x" | chr05:58951212-58958621 | 19.2357 | 5.94083 |
| lncRNA46726 | "x" | chr12:2116903-2121194 | 10.3914 | 6.16753 |
| lncRNA16888 | "x" | chr04:41915504-41916261 | 18.3673 | 3.99367 |
| lncRNA05999 | "x" | chr01:81224469-81230198 | 12.9918 | 4.99904 |
| lncRNA01979 | "x" | chr01:56949669-56955982 | 23.0327 | 12.8892 |
| lncRNA44530 | "x" | chr11:52531248-52533711 | 1.21636 | 2.74698 |
| lncRNA24531 | "x" | chr06:39792730-39798747 | 3.30783 | 5.48689 |
| lncRNA24994 | "x" | chr06:44505350-44507339 | 0.648314 | 1.50522 |
| lncRNA40581 | "x" | chr10:61463527-61465527 | 1.0704 | 2.3759 |
| lncRNA27499 | "u" | chr07:11187041-11189069 | 1.06245 | 0.723162 |
| lncRNA28945 | "u" | chr07:2314998-2315409 | 14.2424 | 9.23098 |
| lncRNA41395 | "x" | chr11:4399140-4401854 | 2.20154 | 3.65238 |
| lncRNA08197 | "u" | chr02:40446012-40446596 | 3.72456 | 5.60332 |
| lncRNA46918 | "x" | chr12:4075663-4077636 | 3.38992 | 4.44085 |
| lncRNA15792 | "x" | chr03:61924601-61925654 | 10.6479 | 13.9928 |
| lncRNA04941 | "x" | chr01:62492586-62494651 | 0.241206 | 0.748285 |
| lncRNA03571 | "u" | chr01:85878926-85891837 | 1.1257 | 0.813893 |
| lncRNA34069 | "u" | chr09:2649417-2650856 | 1.3188 | 1.91372 |
| lncRNA05558 | "x" | chr01:75891414-75896855 | 0.700878 | 2.26321 |
| lncRNA08904 | "x" | chr02:47876949-47877930 | 10.0602 | 17.1944 |
| lncRNA06769 | "x" | chr01:89630012-89631472 | 0.147511 | 0.662811 |
| lncRNA13142 | "x" | chr03:59021833-59027357 | 1.03445 | 1.87081 |
| lncRNA18806 | "u" | chr04:39851052-39853360 | 0.691525 | 1.00926 |
| lncRNA22516 | "x" | chr05:41470828-41477470 | 0.328353 | 0.746753 |
| lncRNA10913 | "x" | chr02:44214760-44221532 | 2.06262 | 3.21935 |
| lncRNA18117 | "x" | chr04:2495075-2498631 | 4.06965 | 1.86793 |
| lncRNA29772 | "u" | chr07:57200561-57202348 | 2.04563 | 1.48395 |
| lncRNA11850 | "x" | chr03:8815494-8821682 | 1.38149 | 0.598332 |
| lncRNA23460 | "u" | chr06:4887198-4887863 | 2.46278 | 3.79909 |
| lncRNA07154 | "x" | chr02:20383477-20385172 | 0.831509 | 1.88851 |
| lncRNA02444 | "x" | chr01:73015694-73019108 | 1.32123 | 0.597669 |
| lncRNA14029 | "x" | chr03:7887302-7889851 | 0.317365 | 1.16333 |
| lncRNA26734 | "u" | chr06:42742275-42743823 | 11.5948 | 16.2536 |
| lncRNA26002 | "x" | chr06:33912725-33913294 | 2.73611 | 4.65254 |
| lncRNA07508 | "x" | chr02:32407023-32408160 | 0.978222 | 2.12944 |
| lncRNA41876 | "u" | chr11:18023024-18024083 | 2.77809 | 1.8993 |
| lncRNA10143 | "x" | chr02:35727778-35734877 | 3.0054 | 1.76889 |
| lncRNA04953 | "x" | chr01:62785029-62787547 | 3.22576 | 2.472 |
| lncRNA06198 | "u" | chr01:83422770-83425338 | 1.56094 | 1.13223 |
| lncRNA03962 | "x" | chr01:44354-54276 | 2.74256 | 1.60366 |
| lncRNA06339 | "x" | chr01:85121586-85125517 | 14.0099 | 18.6402 |
| lncRNA30814 | "x" | chr08:7099228-7104076 | 3.33189 | 7.74521 |
| lncRNA43440 | "x" | chr11:7712544-7726167 | 7.22698 | 12.4272 |
| lncRNA33778 | "x" | chr08:62321255-62324062 | 0.859781 | 1.26608 |
| lncRNA32269 | "u" | chr08:1479383-1480915 | 0.565467 | 0.899969 |
| lncRNA19930 | "x" | chr05:245353-246536 | 2.39007 | 4.46481 |
| lncRNA47393 | "u" | chr12:31697795-31698829 | 1.01842 | 1.59963 |
| lncRNA26550 | "x" | chr06:40805682-40806903 | 1.54375 | 1.00471 |
| lncRNA43141 | "x" | chr11:3153556-3159145 | 14.8432 | 11.1063 |
| lncRNA44239 | "x" | chr11:49460539-49465480 | 8.11876 | 4.86868 |
| lncRNA30082 | "x" | chr07:61111918-61120035 | 0.943427 | 0.423521 |
| lncRNA31576 | "x" | chr08:56515260-56516078 | 6.20265 | 11.7077 |
| lncRNA02886 | "x" | chr01:78616586-78623890 | 0.296897 | 0.960175 |
| lncRNA19007 | "u" | chr04:50911145-50912625 | 0.898345 | 0.553329 |
| lncRNA35064 | "u" | chr09:61653040-61654965 | 1.1712 | 1.64316 |
| lncRNA36687 | "x" | chr09:59394395-59400038 | 1.22324 | 2.19805 |
| lncRNA24199 | "x" | chr06:35929194-35941667 | 0.993374 | 2.11919 |
| lncRNA28708 | "x" | chr07:64759546-64760149 | 24.5136 | 6.3214 |
| lncRNA47871 | "x" | chr12:48552652-48555436 | 1.80568 | 2.98259 |
| lncRNA30061 | "x" | chr07:60919658-60927117 | 5.33229 | 8.43252 |
| lncRNA07419 | "x" | chr02:31140835-31146667 | 0.234431 | 1.20491 |
| lncRNA12271 | "x" | chr03:31910982-31913027 | 4.26549 | 1.48427 |
| lncRNA14456 | "x" | chr03:24946355-24949142 | 10.1657 | 7.38277 |
| lncRNA19352 | "x" | chr04:57582444-57585687 | 1.96774 | 4.30901 |
| lncRNA26984 | "x" | chr06:45225264-45230214 | 0.830998 | 1.80297 |
| lncRNA41970 | "x" | chr11:28095241-28097066 | 3.96932 | 7.88758 |
| lncRNA14487 | "u" | chr03:27053247-27055060 | 1.40494 | 0.979379 |
| lncRNA13615 | "x" | chr03:64059260-64063945 | 3.51476 | 2.64262 |
| lncRNA01934 | "u" | chr01:52713487-52713720 | 115.305 | 58.9977 |
| lncRNA02010 | "u" | chr01:59187297-59188028 | 1.26204 | 0.655681 |
| lncRNA02029 | "u" | chr01:60363090-60363867 | 1.11738 | 0.581558 |
| lncRNA17413 | "x" | chr04:58099537-58101422 | 1.23831 | 3.22118 |
| lncRNA34927 | "u" | chr09:59115707-59116432 | 1.32583 | 0.689763 |
| lncRNA22541 | "u" | chr05:44853751-44854308 | 2.21692 | 1.1568 |
| lncRNA47177 | "u" | chr12:10648193-10648862 | 1.44456 | 0.755369 |
| lncRNA28616 | "x" | chr07:63922766-63927583 | 0.730544 | 0.410997 |
| lncRNA47906 | "x" | chr12:52764632-52767025 | 11.5214 | 14.2 |
| lncRNA03279 | "u" | chr01:82897790-82898754 | 1.45874 | 0.891963 |
| lncRNA45969 | "x" | chr12:48390082-48391594 | 0.681251 | 0.179179 |
| lncRNA34834 | "u" | chr09:56525364-56525961 | 3.14383 | 1.93214 |
| lncRNA08816 | "x" | chr02:46876371-46878474 | 1.70216 | 3.19738 |
| lncRNA27776 | "u" | chr07:48526937-48528130 | 1.05527 | 1.60257 |
| lncRNA19919 | "x" | chr05:111579-116788 | 1.15038 | 0.771215 |
| lncRNA45292 | "x" | chr12:9927248-9929519 | 1.26858 | 2.03328 |
| lncRNA00163 | "x" | chr00:11782542-11786069 | 1.40318 | 2.40955 |
| lncRNA28940 | "u" | chr07:2292794-2294577 | 2.50417 | 1.82417 |
| lncRNA01895 | "x" | chr01:49182436-49192507 | 1.10949 | 2.176 |
| lncRNA14692 | "u" | chr03:43732578-43739980 | 1.11915 | 1.71408 |
| lncRNA41665 | "x" | chr11:8955135-8956887 | 0.769777 | 2.16835 |
| lncRNA22331 | "u" | chr05:23501318-23512832 | 1.24064 | 0.983386 |
| lncRNA45429 | "u" | chr12:24071954-24073267 | 1.4514 | 2.1092 |
| lncRNA45186 | "x" | chr12:5639784-5641459 | 8.64531 | 33.648 |
| lncRNA17903 | "u" | chr04:63973519-63974256 | 3.05428 | 1.97667 |
| lncRNA20305 | "x" | chr05:5576089-5578091 | 2.10645 | 0.842943 |
| lncRNA01155 | "x" | chr01:839098-851722 | 3.27999 | 5.48145 |
| lncRNA45392 | "u" | chr12:20139267-20142210 | 1.37772 | 1.84292 |
| lncRNA03784 | "u" | chr01:88026757-88028262 | 1.03392 | 0.671278 |
| lncRNA04987 | "x" | chr01:64822898-64826400 | 2.01232 | 3.12902 |
| lncRNA38065 | "x" | chr10:42302931-42307238 | 0.734069 | 1.53337 |
| lncRNA41069 | "u" | chr11:899284-912577 | 1.97734 | 1.17869 |
| lncRNA42026 | "x" | chr11:34358179-34358722 | 2.98264 | 1.7808 |
| lncRNA45614 | "x" | chr12:37670839-37688024 | 0.918755 | 1.84519 |
| lncRNA16745 | "x" | chr04:24621932-24631775 | 11.3121 | 25.1872 |
| lncRNA30154 | "u" | chr07:62129586-62129978 | 13.4568 | 19.8991 |
| lncRNA37395 | "x" | chr10:1426758-1429479 | 9.37804 | 7.43379 |
| lncRNA27758 | "u" | chr07:46965842-46966831 | 2.2008 | 3.15671 |
| lncRNA32801 | "u" | chr08:41961416-41966014 | 0.72068 | 0.52539 |
| lncRNA43143 | "x" | chr11:3164930-3166346 | 95.8967 | 220.165 |
| lncRNA30421 | "x" | chr07:64884957-64888007 | 3.82587 | 2.403 |
| lncRNA33699 | "x" | chr08:61380518-61384815 | 1.13764 | 1.64344 |
| lncRNA38651 | "x" | chr10:60857408-60862695 | 8.54225 | 13.5783 |
| lncRNA41382 | "x" | chr11:4255153-4259816 | 2.75856 | 1.92204 |
| lncRNA16804 | "x" | chr04:31110635-31111834 | 0.570641 | 2.24196 |
| lncRNA13132 | "x" | chr03:58944224-58948198 | 0.854061 | 1.39467 |
| lncRNA31765 | "x" | chr08:59087957-59090059 | 2.9175 | 1.13755 |
| lncRNA06800 | "x" | chr01:89954209-89962384 | 0.630294 | 1.63239 |
| lncRNA18788 | "x" | chr04:38662590-38666499 | 3.20308 | 1.80741 |
| lncRNA34259 | "u" | chr09:5208030-5208372 | 81.8131 | 61.092 |
| lncRNA17449 | "x" | chr04:58597639-58600314 | 4.82046 | 2.72233 |
| lncRNA25878 | "x" | chr06:32070494-32079972 | 3.36671 | 2.43783 |
| lncRNA32376 | "x" | chr08:2839998-2841001 | 2.19938 | 0.567631 |
| lncRNA14389 | "o" | chr03:19408760-19427739 | 0.266369 | 0.957875 |
| lncRNA15388 | "x" | chr03:57675976-57687123 | 2.93413 | 1.71793 |
| lncRNA20960 | "x" | chr05:53786663-53790030 | 1.05812 | 0.768677 |
| lncRNA35841 | "x" | chr09:3516950-3518990 | 0.798763 | 1.58185 |
| lncRNA05704 | "u" | chr01:77824879-77826005 | 2.16966 | 1.45623 |
| lncRNA14548 | "x" | chr03:30408627-30413568 | 2.58528 | 4.05784 |
| lncRNA11076 | "x" | chr02:45756371-45761955 | 1.336 | 2.08219 |
| lncRNA08354 | "x" | chr02:41967457-41971815 | 53.8787 | 84.1713 |
| lncRNA42525 | "x" | chr11:50350297-50352818 | 0.433071 | 1.03626 |
| lncRNA46306 | "x" | chr12:63841272-63846257 | 7.4301 | 3.34268 |
| lncRNA39130 | "u" | chr10:572883-573831 | 1.05988 | 0.634693 |
| lncRNA23274 | "x" | chr06:1353671-1356856 | 0.981228 | 0.479591 |
| lncRNA18077 | "x" | chr04:1894159-1896762 | 9.51225 | 4.86795 |
| lncRNA31696 | "x" | chr08:58160800-58166163 | 14.4108 | 11.0724 |
| lncRNA24303 | "x" | chr06:37149259-37150216 | 7.78907 | 3.90944 |
| lncRNA15417 | "x" | chr03:58039946-58042768 | 0.710258 | 2.31137 |
| lncRNA25246 | "x" | chr06:1406686-1407431 | 2.3687 | 0.767725 |
| lncRNA14290 | "x" | chr03:13182850-13192978 | 1.01953 | 0.452168 |
| lncRNA36191 | "u" | chr09:21022259-21057350 | 1.32573 | 0.554869 |
| lncRNA03139 | "x" | chr01:81421625-81427337 | 1.59453 | 2.15496 |
| lncRNA23314 | "x" | chr06:1846026-1849951 | 1.08671 | 0.617904 |
| lncRNA32063 | "u" | chr08:62370912-62371569 | 10.4587 | 7.70569 |
| lncRNA41622 | "x" | chr11:8172865-8192556 | 1.51115 | 1.09892 |
| lncRNA47162 | "u" | chr12:10294938-10295144 | 27.6036 | 71.8209 |
| lncRNA13363 | "x" | chr03:61325253-61326031 | 36.2918 | 48.8853 |
| lncRNA23925 | "x" | chr06:31780474-31781323 | 7.55899 | 5.64493 |
| lncRNA05091 | "x" | chr01:68053131-68064366 | 4.84439 | 7.55083 |
| lncRNA44910 | "x" | chr12:2477481-2480583 | 1.78736 | 3.37763 |
| lncRNA24891 | "x" | chr06:43510767-43511331 | 9.23884 | 6.36172 |
| lncRNA46297 | "x" | chr12:63770747-63771773 | 1.17698 | 0.719108 |
| lncRNA17231 | "u" | chr04:55086365-55086795 | 13.3713 | 19.0774 |
| lncRNA44788 | "u" | chr12:1281408-1281787 | 60.2939 | 45.591 |
| lncRNA34928 | "u" | chr09:59116515-59118274 | 0.744687 | 0.481784 |
| lncRNA47212 | "u" | chr12:12854692-12859394 | 3.85752 | 2.83939 |
| lncRNA11320 | "x" | chr02:48247967-48254209 | 1.27393 | 0.368034 |
| lncRNA40150 | "x" | chr10:52566135-52569890 | 4.36783 | 6.44112 |
| lncRNA07800 | "x" | chr02:36008352-36013800 | 13.2176 | 25.5164 |
| lncRNA22934 | "x" | chr05:62256442-62263477 | 0.442003 | 0.925944 |
| lncRNA28046 | "x" | chr07:56499980-56504055 | 0.655576 | 0.265622 |
| lncRNA42472 | "x" | chr11:49822521-49823919 | 1.86228 | 1.20658 |
| lncRNA11362 | "x" | chr02:48708502-48728575 | 6.65332 | 8.85358 |
| lncRNA23800 | "x" | chr06:28826493-28832938 | 1.1861 | 1.69048 |
| lncRNA48046 | "x" | chr12:62498109-62498749 | 10.8324 | 14.6295 |
| lncRNA27759 | "u" | chr07:46966934-46968259 | 4.21051 | 3.15042 |
| lncRNA15343 | "x" | chr03:57120138-57122993 | 4.69746 | 7.58818 |
| lncRNA41368 | "x" | chr11:4089249-4094356 | 0.734287 | 1.25229 |
| lncRNA02295 | "u" | chr01:70529797-70530704 | 6.44431 | 8.46134 |
| lncRNA20847 | "u" | chr05:41680816-41681211 | 8.92964 | 13.9463 |
| lncRNA20447 | "x" | chr05:8250660-8252088 | 1.00498 | 0.46444 |
| lncRNA43140 | "x" | chr11:3153556-3159145 | 1.02848 | 0.675046 |
| lncRNA28686 | "x" | chr07:64574661-64575300 | 1.25914 | 2.28961 |
| lncRNA44173 | "x" | chr11:48467534-48471289 | 2.63986 | 3.66222 |
| lncRNA46468 | "x" | chr12:65139387-65148741 | 0.552594 | 0.938033 |
| lncRNA25430 | "x" | chr06:3868148-3874945 | 0.780793 | 1.58362 |
| lncRNA29289 | "x" | chr07:24481851-24484486 | 4.01937 | 5.03946 |
| lncRNA24889 | "x" | chr06:43504424-43505083 | 26.7054 | 19.8816 |
| lncRNA30953 | "x" | chr08:25698587-25700540 | 0.36089 | 0.777232 |
| lncRNA36662 | "u" | chr09:59023307-59023685 | 22.7527 | 15.5001 |
| lncRNA38984 | "u" | chr10:63949720-63950141 | 6.73388 | 4.08541 |
| lncRNA44331 | "u" | chr11:50463114-50464393 | 0.83314 | 0.503348 |
| lncRNA11341 | "x" | chr02:48433905-48444181 | 2.87251 | 4.18638 |
| lncRNA38799 | "x" | chr10:62143218-62146156 | 8.07293 | 22.5104 |
| lncRNA43911 | "u" | chr11:36435707-36436484 | 1.72996 | 1.05192 |
| lncRNA35793 | "x" | chr09:2897148-2899315 | 3.9551 | 2.25384 |
| lncRNA38539 | "x" | chr10:59762290-59766956 | 1.15622 | 1.98577 |
| lncRNA38490 | "x" | chr10:59311807-59315187 | 0.440336 | 0.88829 |
| lncRNA46237 | "x" | chr12:63259974-63269260 | 0.998937 | 2.27398 |
| lncRNA33518 | "x" | chr08:59142152-59144723 | 11.1348 | 29.149 |
| lncRNA00491 | "x" | chr00:20647169-20649279 | 59.2329 | 72.9953 |
| lncRNA43652 | "x" | chr11:17153328-17153886 | 24.1962 | 43.6906 |
| lncRNA45768 | "x" | chr12:44433414-44437619 | 11.3686 | 6.54991 |
| lncRNA21664 | "x" | chr05:1860322-1862631 | 27.3595 | 14.9437 |
| lncRNA05868 | "x" | chr01:79879393-79881916 | 0.313051 | 0.757396 |
| lncRNA09252 | "u" | chr02:12884282-12884964 | 2.08613 | 1.27292 |
| lncRNA24218 | "x" | chr06:36126486-36128866 | 1.84648 | 2.99868 |
| lncRNA08942 | "x" | chr02:48226397-48235324 | 6.6542 | 5.09379 |
| lncRNA39320 | "u" | chr10:2630874-2633164 | 1.69184 | 1.25607 |
| lncRNA20306 | "x" | chr05:5576089-5578091 | 2.24722 | 1.35137 |
| lncRNA06307 | "x" | chr01:84798175-84801154 | 3.01373 | 1.97018 |
| lncRNA11920 | "u" | chr03:9998363-9999624 | 1.18823 | 0.772062 |
| lncRNA14221 | "x" | chr03:10670226-10672021 | 98.4507 | 68.1038 |
| lncRNA22343 | "x" | chr05:24234353-24235988 | 2.00713 | 2.71256 |
| lncRNA42130 | "x" | chr11:36897737-36899171 | 11.2406 | 16.8582 |
| lncRNA13351 | "o" | chr03:61177669-61181739 | 1.85436 | 4.75413 |
| lncRNA33104 | "x" | chr08:53594241-53595881 | 1.77623 | 0.576324 |
| lncRNA32916 | "x" | chr08:48364803-48366524 | 0.522566 | 1.22929 |
| lncRNA39698 | "x" | chr10:23503306-23504200 | 0.538399 | 0.976884 |
| lncRNA07169 | "x" | chr02:21192405-21201016 | 0.411275 | 0.700396 |
| lncRNA13429 | "x" | chr03:61943065-61945914 | 0.599022 | 3.05224 |
| lncRNA00411 | "x" | chr00:16968148-16969339 | 39.3492 | 29.8019 |
| lncRNA43252 | "u" | chr11:4566229-4566628 | 2.4562 | 4.57823 |
| lncRNA13281 | "x" | chr03:60531633-60532509 | 6.89027 | 2.43306 |
| lncRNA00923 | "x" | chr00:16636610-16639803 | 0.963011 | 1.42407 |
| lncRNA42911 | "x" | chr11:957463-959086 | 3.02504 | 2.27328 |
| lncRNA15669 | "x" | chr03:60710386-60715329 | 4.3552 | 2.06193 |
| lncRNA10321 | "x" | chr02:37736167-37742554 | 2.1069 | 1.63974 |
| lncRNA31562 | "x" | chr08:56302600-56304917 | 3.01425 | 1.07134 |
| lncRNA47816 | "x" | chr12:47682781-47686174 | 3.62592 | 5.10159 |
| lncRNA26023 | "x" | chr06:34354168-34356824 | 0.603211 | 1.30586 |
| lncRNA36554 | "x" | chr09:55925014-55926948 | 1.18304 | 0.512235 |
| lncRNA26398 | "j" | chr06:38927891-38929148 | 1.55756 | 2.74405 |
| lncRNA47410 | "u" | chr12:33581535-33582509 | 10.6765 | 13.5424 |
| lncRNA23567 | "x" | chr06:15436255-15438408 | 2.06354 | 4.6154 |
| lncRNA28689 | "x" | chr07:64591910-64596903 | 12.3898 | 15.8352 |
| lncRNA11012 | "x" | chr02:45161432-45169302 | 1.16361 | 1.70348 |
| lncRNA18214 | "o" | chr04:3420331-3430862 | 1.13247 | 2.28419 |
| lncRNA30029 | "x" | chr07:60598617-60601321 | 8.23868 | 5.43291 |
| lncRNA23972 | "x" | chr06:32606320-32608000 | 5.71183 | 3.39009 |
| lncRNA29602 | "x" | chr07:53752299-53758204 | 4.36082 | 3.26436 |
| lncRNA38244 | "x" | chr10:51072596-51077244 | 0.392698 | 1.14431 |
| lncRNA30259 | "x" | chr07:63199660-63206577 | 2.40425 | 1.4955 |
| lncRNA36389 | "u" | chr09:44742422-44744936 | 7.65256 | 9.41036 |
| lncRNA39101 | "x" | chr10:213418-215035 | 3.07519 | 5.96572 |
| lncRNA19135 | "u" | chr04:54274312-54275279 | 7.06224 | 9.16404 |
| lncRNA22544 | "x" | chr05:45539871-45546337 | 5.28873 | 7.64286 |
| lncRNA29688 | "x" | chr07:55929218-55930841 | 11.4865 | 5.18631 |
| lncRNA24579 | "x" | chr06:40309434-40311025 | 3.78877 | 2.62418 |
| lncRNA43981 | "x" | chr11:41129654-41130523 | 9.22838 | 6.79448 |
| lncRNA20630 | "x" | chr05:22403198-22406376 | 1.19813 | 0.518207 |
| lncRNA38368 | "x" | chr10:57374014-57382100 | 38.5004 | 61.472 |
| lncRNA09360 | "x" | chr02:17778514-17790343 | 1.0509 | 0.480926 |
| lncRNA26704 | "x" | chr06:42379438-42382533 | 1.41745 | 0.838561 |
| lncRNA32031 | "u" | chr08:62061338-62062001 | 7.6405 | 10.1873 |
| lncRNA38375 | "x" | chr10:57474447-57476031 | 4.49939 | 14.6774 |
| lncRNA03567 | "u" | chr01:85878926-85891837 | 8.54344 | 16.3201 |
| lncRNA31831 | "u" | chr08:59784452-59785195 | 10.0116 | 7.56077 |
| lncRNA23907 | "u" | chr06:31305195-31305934 | 6.63296 | 8.8174 |
| lncRNA25143 | "x" | chr06:56284-58268 | 6.57815 | 4.84407 |
| lncRNA28696 | "x" | chr07:64642056-64644993 | 1.84739 | 3.33056 |
| lncRNA19679 | "x" | chr04:61758848-61763568 | 0.894072 | 2.03071 |
| lncRNA37557 | "x" | chr10:3390384-3395700 | 0.802685 | 2.43019 |
| lncRNA34108 | "x" | chr09:3264031-3287147 | 2.55425 | 0.894344 |
| lncRNA47702 | "x" | chr12:45637958-45643012 | 2.99063 | 1.6198 |
| lncRNA39633 | "x" | chr10:19021326-19025617 | 1.05482 | 0.548426 |
| lncRNA45218 | "u" | chr12:6379617-6381465 | 1.0173 | 1.43424 |
| lncRNA24040 | "u" | chr06:33637824-33638258 | 14.6549 | 10.3655 |
| lncRNA08433 | "x" | chr02:42936943-42938397 | 2.93814 | 1.28721 |
| lncRNA09653 | "u" | chr02:29644843-29645462 | 7.6861 | 5.58919 |
| lncRNA36202 | "x" | chr09:21105414-21107629 | 0.548448 | 0.86474 |
| lncRNA07101 | "u" | chr02:18018318-18020259 | 1.53355 | 2.04529 |
| lncRNA04452 | "x" | chr01:11926765-11929412 | 3.81071 | 4.81906 |
| lncRNA33261 | "x" | chr08:55748310-55749035 | 58.3251 | 94.6891 |
| lncRNA25311 | "u" | chr06:2076952-2077825 | 2.50068 | 1.68731 |
| lncRNA07443 | "x" | chr02:31501106-31501779 | 2.70773 | 1.377 |
| lncRNA14821 | "x" | chr03:46543662-46546981 | 2.27801 | 1.10858 |
| lncRNA37339 | "x" | chr10:909609-910153 | 3.97975 | 2.19558 |
| lncRNA39437 | "x" | chr10:4720082-4724156 | 3.09804 | 2.03093 |
| lncRNA12910 | "u" | chr03:56313596-56314157 | 1.11194 | 2.03124 |
| lncRNA44676 | "x" | chr12:306513-309886 | 5.34841 | 10.7583 |
| lncRNA21010 | "x" | chr05:56314285-56316542 | 4.40116 | 3.50309 |
| lncRNA01010 | "u" | chr00:19335699-19336170 | 1.69093 | 3.05883 |
| lncRNA25918 | "u" | chr06:32743276-32743493 | 99.8687 | 179.776 |
| lncRNA27116 | "u" | chr07:684926-685162 | 26.1967 | 11.4027 |
| lncRNA36142 | "u" | chr09:16515058-16517587 | 2.6863 | 2.0862 |
| lncRNA06780 | "x" | chr01:89737897-89740311 | 4.94849 | 7.36719 |
| lncRNA31851 | "u" | chr08:60016566-60017917 | 7.04042 | 5.51896 |
| lncRNA36480 | "x" | chr09:50463632-50466385 | 0.735167 | 0.402063 |
| lncRNA01454 | "u" | chr01:6295270-6298376 | 4.57418 | 5.6477 |
| lncRNA34187 | "u" | chr09:4124724-4125416 | 11.0437 | 14.2813 |
| lncRNA44569 | "x" | chr11:52945836-52949973 | 1.06476 | 3.13976 |
| lncRNA05699 | "x" | chr01:77752794-77757781 | 2.26538 | 3.22776 |
| lncRNA26108 | "x" | chr06:35698536-35703436 | 4.12114 | 2.47252 |
| lncRNA14818 | "x" | chr03:46519059-46521744 | 1.03322 | 1.58672 |
| lncRNA13570 | "x" | chr03:63559108-63560219 | 3.40919 | 6.21531 |
| lncRNA45965 | "x" | chr12:48333345-48336580 | 1.38309 | 2.40152 |
| lncRNA30945 | "u" | chr08:24809186-24809595 | 12.5545 | 8.41955 |
| lncRNA45339 | "x" | chr12:14121081-14123112 | 0.640031 | 0.995113 |
| lncRNA39626 | "x" | chr10:18003740-18006532 | 2.13413 | 3.53966 |
| lncRNA14011 | "x" | chr03:7714579-7716038 | 3.18299 | 6.16942 |
| lncRNA22689 | "u" | chr05:57615824-57617404 | 1.35173 | 2.33683 |
| lncRNA32098 | "u" | chr08:62636347-62637020 | 0.916314 | 1.59369 |
| lncRNA17447 | "x" | chr04:58565791-58569731 | 39.0457 | 57.2397 |
| lncRNA24775 | "x" | chr06:42166734-42168437 | 0.605088 | 1.30938 |
| lncRNA43713 | "x" | chr11:21504135-21524244 | 10.2637 | 3.312 |
| lncRNA42811 | "x" | chr11:53359850-53364725 | 1.62187 | 2.44983 |
| lncRNA08710 | "u" | chr02:45738045-45738772 | 0.814422 | 1.40428 |
| lncRNA25204 | "x" | chr06:871262-874146 | 2.53839 | 1.10207 |
| lncRNA46282 | "x" | chr12:63660374-63663035 | 11.5499 | 7.80347 |
| lncRNA33445 | "x" | chr08:58262558-58264641 | 5.48738 | 6.85462 |
| lncRNA44204 | "x" | chr11:48890354-48891267 | 38.3544 | 79.1179 |
| lncRNA38938 | "x" | chr10:63600247-63603604 | 9.75346 | 20.0148 |
| lncRNA45639 | "x" | chr12:39447346-39448565 | 13.469 | 4.73032 |
| lncRNA29501 | "x" | chr07:49509216-49521517 | 0.570073 | 0.966351 |
| lncRNA20227 | "x" | chr05:4214705-4215788 | 4.54131 | 2.1365 |
| lncRNA12354 | "x" | chr03:42183461-42186209 | 0.742936 | 1.7436 |
| lncRNA46345 | "u" | chr12:64145418-64145700 | 90.248 | 62.4183 |
| lncRNA34608 | "u" | chr09:33957800-33958211 | 5.75548 | 3.38109 |
| lncRNA45681 | "x" | chr12:41307346-41315062 | 2.39019 | 1.5074 |
| lncRNA18140 | "x" | chr04:2692464-2695221 | 0.305817 | 0.792025 |
| lncRNA24452 | "x" | chr06:38910664-38912035 | 3.8359 | 1.93995 |
| lncRNA34144 | "x" | chr09:3673361-3681783 | 0.68826 | 0.42187 |
| lncRNA05377 | "u" | chr01:73419349-73419919 | 4.40188 | 2.8525 |
| lncRNA43400 | "u" | chr11:6326373-6327925 | 0.789355 | 0.511691 |
| lncRNA20334 | "x" | chr05:5840871-5851353 | 0.939297 | 0.22324 |
| lncRNA46876 | "x" | chr12:3627523-3630579 | 10.3939 | 19.7495 |
| lncRNA11597 | "x" | chr03:1367432-1377406 | 0.461519 | 1.15056 |
| lncRNA44095 | "x" | chr11:46704760-46706429 | 1.31557 | 1.94514 |
| lncRNA11746 | "x" | chr03:7152854-7154205 | 20.3141 | 39.408 |
| lncRNA40463 | "x" | chr10:60285915-60290148 | 7.74792 | 5.88223 |
| lncRNA15153 | "x" | chr03:53844508-53847335 | 1.78208 | 4.44015 |
| lncRNA17852 | "x" | chr04:63396425-63397904 | 3.94074 | 5.19559 |
| lncRNA34945 | "x" | chr09:59345025-59357196 | 2.01673 | 2.97711 |
| lncRNA25157 | "x" | chr06:266780-271800 | 1.09159 | 2.03151 |
| lncRNA14696 | "x" | chr03:43759845-43774381 | 0.244733 | 0.633009 |
| lncRNA02435 | "x" | chr01:72957706-72961480 | 2.37389 | 1.67612 |
| lncRNA47732 | "x" | chr12:46357604-46362460 | 1.30424 | 0.96769 |
| lncRNA11327 | "x" | chr02:48294712-48298166 | 0.256028 | 0.664924 |
| lncRNA41593 | "u" | chr11:7496059-7496329 | 47.037 | 27.774 |
| lncRNA07541 | "x" | chr02:32773570-32774326 | 0.460252 | 1.19967 |
| lncRNA22797 | "x" | chr05:60144524-60145753 | 2.18513 | 4.4394 |
| lncRNA34658 | "x" | chr09:42741329-42742301 | 2.33406 | 1.44647 |
| lncRNA05089 | "x" | chr01:68009110-68023501 | 2.36529 | 1.76177 |
| lncRNA46801 | "u" | chr12:2915276-2915524 | 69.8613 | 41.356 |
| lncRNA14043 | "x" | chr03:8091697-8092546 | 2.12713 | 5.52726 |
| lncRNA42377 | "x" | chr11:48427716-48442402 | 3.10936 | 5.32256 |
| lncRNA39366 | "x" | chr10:3201654-3211010 | 4.02504 | 2.36725 |
| lncRNA02804 | "x" | chr01:77730525-77733865 | 9.62557 | 7.09072 |
| lncRNA19234 | "x" | chr04:55805627-55808167 | 1.22433 | 0.499732 |
| lncRNA35028 | "x" | chr09:61038510-61039828 | 7.39926 | 5.78307 |
| lncRNA08407 | "u" | chr02:42619848-42620650 | 0.880399 | 1.44639 |
| lncRNA01449 | "u" | chr01:5729278-5730380 | 0.833811 | 0.500323 |
| lncRNA18221 | "u" | chr04:3552380-3553428 | 0.848533 | 0.509849 |
| lncRNA48364 | "x" | chr12:65216364-65221534 | 0.733612 | 1.45697 |
| lncRNA38418 | "x" | chr10:58156780-58157715 | 133.669 | 226.829 |
| lncRNA26583 | "x" | chr06:41103360-41106812 | 0.793192 | 0.466802 |
| lncRNA32418 | "x" | chr08:3531349-3533010 | 6.81342 | 3.82236 |
| lncRNA13704 | "x" | chr03:75629-77619 | 3.06733 | 4.65317 |
| lncRNA26996 | "x" | chr06:45345358-45359353 | 0.314645 | 0.815492 |
| lncRNA09504 | "u" | chr02:24201064-24202066 | 0.627191 | 1.02999 |
| lncRNA40462 | "x" | chr10:60282481-60283364 | 77.2471 | 137.236 |
| lncRNA42623 | "x" | chr11:51520906-51527993 | 1.12398 | 1.73897 |
| lncRNA23339 | "x" | chr06:2318595-2322030 | 1.60733 | 2.27139 |
| lncRNA27763 | "u" | chr07:46978271-46979352 | 1.24897 | 0.806831 |
| lncRNA43243 | "x" | chr11:4479439-4483278 | 1.09976 | 0.771583 |
| lncRNA24238 | "x" | chr06:36344390-36356737 | 0.671586 | 0.28204 |
| lncRNA19780 | "x" | chr04:62784622-62788098 | 1.15826 | 0.543929 |
| lncRNA43312 | "x" | chr11:5108251-5112340 | 8.81508 | 13.8685 |
| lncRNA22303 | "u" | chr05:20711188-20713311 | 3.9663 | 4.96259 |
| lncRNA11065 | "u" | chr02:45548414-45550675 | 43.1741 | 51.8632 |
| lncRNA01767 | "u" | chr01:36270828-36272800 | 5.0698 | 4.02293 |
| lncRNA20204 | "x" | chr05:3876344-3878140 | 12.2467 | 26.109 |
| lncRNA12822 | "x" | chr03:53901215-53903729 | 1.20916 | 0.683441 |
| lncRNA20613 | "x" | chr05:20592932-20598301 | 1.66715 | 3.17059 |
| lncRNA16025 | "x" | chr03:64452644-64456165 | 1.61958 | 0.768679 |
| lncRNA41293 | "x" | chr11:3181785-3190461 | 3.61952 | 2.07064 |
| lncRNA23524 | "u" | chr06:10875203-10876083 | 0.745443 | 1.21969 |
| lncRNA05799 | "u" | chr01:78946374-78947025 | 1.24209 | 2.02184 |
| lncRNA38699 | "x" | chr10:61217212-61220722 | 0.718627 | 1.62503 |
| lncRNA35255 | "x" | chr09:64404040-64405561 | 18.8034 | 28.5621 |
| lncRNA41381 | "x" | chr11:4235209-4237261 | 10.5091 | 14.2113 |
| lncRNA26492 | "x" | chr06:40055226-40056449 | 6.05778 | 4.50952 |
| lncRNA34376 | "x" | chr09:10987957-10989890 | 5.86711 | 14.8687 |
| lncRNA33452 | "x" | chr08:58349120-58352212 | 0.974154 | 0.531715 |
| lncRNA38156 | "x" | chr10:47041421-47052335 | 1.18053 | 0.47147 |
| lncRNA40950 | "x" | chr10:64630981-64633513 | 3.78353 | 2.93566 |
| lncRNA35917 | "x" | chr09:4603002-4605126 | 1.27997 | 2.27167 |
| lncRNA36474 | "u" | chr09:50294502-50297747 | 1.37991 | 2.05413 |
| lncRNA26758 | "x" | chr06:43067776-43068845 | 10.1736 | 16.4064 |
| lncRNA35436 | "x" | chr09:66402328-66414666 | 1.61923 | 0.769506 |
| lncRNA28833 | "x" | chr07:764088-775870 | 2.63548 | 1.50398 |
| lncRNA43283 | "x" | chr11:4788417-4801208 | 10.0884 | 6.62055 |
| lncRNA22092 | "x" | chr05:8393206-8399475 | 4.88934 | 1.99292 |
| lncRNA27982 | "u" | chr07:55351964-55352334 | 60.2182 | 46.1231 |
| lncRNA43454 | "x" | chr11:8004127-8013565 | 8.95695 | 11.8329 |
| lncRNA09662 | "u" | chr02:29732676-29733661 | 4.568 | 5.9794 |
| lncRNA43947 | "x" | chr11:37739017-37741967 | 28.4644 | 36.8658 |
| lncRNA06075 | "x" | chr01:82089175-82095527 | 1.66101 | 1.19641 |
| lncRNA01400 | "x" | chr01:4659288-4670992 | 7.22447 | 4.22456 |
| lncRNA32933 | "x" | chr08:49466469-49467657 | 0.996105 | 0.649758 |
| lncRNA04169 | "x" | chr01:2274517-2283875 | 2.74039 | 6.54758 |
| lncRNA07559 | "x" | chr02:32968402-32972526 | 4.97173 | 6.20258 |
| lncRNA32299 | "x" | chr08:1853182-1855877 | 3.12861 | 6.52716 |
| lncRNA22861 | "u" | chr05:61332832-61333396 | 30.3801 | 24.0666 |
| lncRNA00431 | "x" | chr00:17945600-17948327 | 1.06607 | 1.6316 |
| lncRNA09060 | "u" | chr02:49400679-49402531 | 0.734887 | 0.473502 |
| lncRNA33780 | "u" | chr08:62337301-62338659 | 3.13086 | 2.36361 |
| lncRNA37469 | "x" | chr10:2315210-2319689 | 2.49522 | 1.21622 |
| lncRNA37138 | "x" | chr09:65895465-65898023 | 16.8082 | 11.1132 |
| lncRNA30378 | "x" | chr07:64402690-64403327 | 4.97126 | 6.91708 |
| lncRNA23223 | "u" | chr06:695956-708656 | 1.07706 | 0.630463 |
| lncRNA22540 | "u" | chr05:44851487-44852749 | 1.32995 | 1.86501 |
| lncRNA13135 | "x" | chr03:58960420-58965564 | 13.6052 | 7.93501 |
| lncRNA19574 | "x" | chr04:60598302-60599212 | 2.48594 | 0.923649 |
| lncRNA05166 | "x" | chr01:70131981-70142726 | 0.698341 | 0.918093 |
| lncRNA19584 | "x" | chr04:60660615-60662744 | 1.82207 | 0.911532 |
| lncRNA29611 | "x" | chr07:53956839-53957377 | 3.21567 | 4.80029 |
| lncRNA44251 | "x" | chr11:49630535-49631024 | 28.763 | 22.367 |
| lncRNA16310 | "x" | chr04:2998012-3006166 | 0.798415 | 0.432958 |
| lncRNA28597 | "x" | chr07:63742297-63744672 | 27.7214 | 20.7818 |
| lncRNA05914 | "x" | chr01:80335949-80341370 | 2.03182 | 2.87261 |
| lncRNA33839 | "x" | chr09:15630-16603 | 12.584 | 19.4171 |
| lncRNA13646 | "x" | chr03:64320303-64321487 | 93.8176 | 63.5749 |
| lncRNA03251 | "x" | chr01:82619599-82622439 | 0.763713 | 1.7225 |
| lncRNA36524 | "u" | chr09:54117501-54117847 | 8.30654 | 13.3016 |
| lncRNA00877 | "x" | chr00:15611984-15614634 | 72.4529 | 129.682 |
| lncRNA38209 | "x" | chr10:49735089-49737049 | 0.675332 | 0.95528 |
| lncRNA05178 | "u" | chr01:70334519-70334954 | 1.71233 | 3.36111 |
| lncRNA07015 | "u" | chr02:14973872-14974083 | 74.5023 | 143.356 |
| lncRNA19210 | "u" | chr04:55395941-55397028 | 1.24699 | 1.79791 |
| lncRNA38822 | "x" | chr10:62415316-62418815 | 34.0063 | 27.0926 |
| lncRNA24379 | "x" | chr06:38112269-38118914 | 15.5164 | 23.5446 |
| lncRNA45891 | "x" | chr12:47295645-47301387 | 3.58086 | 2.21846 |
| lncRNA37247 | "u" | chr09:67345371-67347794 | 1.35803 | 1.01738 |
| lncRNA22548 | "x" | chr05:46933087-46934236 | 2.04033 | 2.94971 |
| lncRNA11083 | "x" | chr02:45823533-45828102 | 0.55179 | 0.793361 |
| lncRNA42988 | "x" | chr11:1541090-1544796 | 9.04308 | 3.3658 |
| lncRNA30832 | "x" | chr08:7901087-7902621 | 1.35263 | 3.5146 |
| lncRNA43863 | "x" | chr11:35165265-35168495 | 38.2024 | 47.2212 |
| lncRNA15770 | "x" | chr03:61719902-61722713 | 0.755841 | 1.11824 |
| lncRNA07923 | "x" | chr02:37401402-37404168 | 4.25414 | 3.1126 |
| lncRNA44315 | "x" | chr11:50296678-50298681 | 28.6773 | 36.6042 |
| lncRNA26314 | "x" | chr06:38002897-38009075 | 4.25512 | 2.58993 |
| lncRNA15087 | "x" | chr03:52940388-52943622 | 1.12326 | 1.77288 |
| lncRNA47715 | "x" | chr12:45864704-45866058 | 7.3748 | 12.4887 |
| lncRNA43786 | "x" | chr11:28592235-28599231 | 7.72777 | 11.6441 |
| lncRNA45892 | "x" | chr12:47295645-47301387 | 0.286541 | 0.741527 |
| lncRNA28943 | "x" | chr07:2303072-2305559 | 48.3387 | 36.0732 |
| lncRNA34371 | "u" | chr09:10711335-10712541 | 0.950584 | 1.39945 |
| lncRNA39036 | "x" | chr10:64341199-64347037 | 1.35889 | 1.88054 |
| lncRNA01875 | "x" | chr01:47303068-47304549 | 1.23346 | 1.89253 |
| lncRNA41698 | "x" | chr11:10030114-10034259 | 0.883953 | 0.311467 |
| lncRNA39256 | "x" | chr10:1803608-1808066 | 15.4193 | 9.11495 |
| lncRNA00318 | "u" | chr00:15074048-15074719 | 2.43538 | 3.52955 |
| lncRNA20681 | "u" | chr05:28029690-28031220 | 0.729385 | 1.05677 |
| lncRNA27293 | "u" | chr07:3360886-3361291 | 45.5743 | 35.3323 |
| lncRNA45187 | "x" | chr12:5639784-5641459 | 1.41661 | 3.67666 |
| lncRNA29898 | "x" | chr07:59175602-59176920 | 4.51244 | 11.9571 |
| lncRNA20271 | "x" | chr05:4911581-4912933 | 2.24749 | 1.21372 |
| lncRNA11250 | "x" | chr02:47453551-47465026 | 0.719049 | 0.546829 |
| lncRNA33035 | "u" | chr08:52084899-52086301 | 0.570444 | 0.856141 |
| lncRNA15174 | "x" | chr03:54121750-54127481 | 1.01911 | 0.658227 |
| lncRNA42197 | "x" | chr11:43097176-43099149 | 1.78438 | 2.50499 |
| lncRNA42473 | "x" | chr11:49825181-49832412 | 0.57507 | 1.24153 |
| lncRNA32288 | "x" | chr08:1737351-1739951 | 11.9144 | 8.22426 |
| lncRNA20690 | "x" | chr05:28060780-28074769 | 64.5208 | 43.3477 |
| lncRNA26259 | "x" | chr06:37376493-37381443 | 0.949136 | 1.92683 |
| lncRNA27927 | "x" | chr07:54233029-54234010 | 15.8765 | 33.9974 |
| lncRNA34985 | "x" | chr09:60034856-60044112 | 7.38935 | 16.2213 |
| lncRNA37321 | "x" | chr10:722065-725047 | 2.27971 | 3.84521 |
| lncRNA22986 | "u" | chr05:63026809-63027855 | 31.3606 | 37.6592 |
| lncRNA12315 | "u" | chr03:38258863-38260170 | 1.49354 | 2.09083 |
| lncRNA30525 | "x" | chr08:725503-726188 | 38.9421 | 24.114 |
| lncRNA34657 | "u" | chr09:42736565-42740471 | 12.8476 | 10.7319 |
| lncRNA43079 | "x" | chr11:2487181-2489996 | 2.1801 | 5.61726 |
| lncRNA37456 | "x" | chr10:2209165-2214155 | 4.18509 | 3.14486 |
| lncRNA11845 | "u" | chr03:8639596-8640072 | 7.46884 | 5.10457 |
| lncRNA27860 | "x" | chr07:52852234-52852891 | 8.32042 | 21.5219 |
| lncRNA10162 | "u" | chr02:35970208-35970812 | 3.90388 | 5.54659 |
| lncRNA03450 | "x" | chr01:84726972-84729190 | 10.4405 | 8.44944 |
| lncRNA15198 | "u" | chr03:54422244-54423974 | 5.33281 | 4.24668 |
| lncRNA24294 | "x" | chr06:37036821-37038193 | 12.0921 | 7.54453 |
| lncRNA45480 | "u" | chr12:29792919-29795217 | 1.87757 | 2.42568 |
| lncRNA09921 | "u" | chr02:33272893-33274233 | 1.49182 | 1.03858 |
| lncRNA30218 | "x" | chr07:62742398-62770824 | 16.6964 | 3.96747 |
| lncRNA06292 | "x" | chr01:84506542-84509427 | 4.6704 | 10.1482 |
| lncRNA21239 | "x" | chr05:61637099-61640215 | 0.803214 | 1.24795 |
| lncRNA24190 | "x" | chr06:35828933-35829866 | 9.11324 | 14.8059 |
| lncRNA04374 | "u" | chr01:7579928-7581240 | 0.514142 | 0.789667 |
| lncRNA45682 | "u" | chr12:41316271-41317086 | 1.16764 | 1.79412 |
| lncRNA19730 | "x" | chr04:62219562-62221469 | 19.1958 | 30.5203 |
| lncRNA42513 | "x" | chr11:50264071-50267585 | 3.09855 | 2.19919 |
| lncRNA44075 | "x" | chr11:46281465-46286680 | 0.68521 | 0.498304 |
| lncRNA18575 | "u" | chr04:20107421-20109735 | 2.2623 | 2.88405 |
| lncRNA38035 | "x" | chr10:40716930-40723657 | 4.51467 | 7.3045 |
| lncRNA46703 | "x" | chr12:1883667-1886869 | 4.20214 | 2.20111 |
| lncRNA31670 | "x" | chr08:57776441-57781889 | 5.96534 | 4.52744 |
| lncRNA05702 | "x" | chr01:77786296-77789212 | 1.10856 | 0.681176 |
| lncRNA14795 | "u" | chr03:46211698-46212583 | 0.773993 | 1.27052 |
| lncRNA05902 | "u" | chr01:80218157-80218859 | 21.0432 | 16.7847 |
| lncRNA42343 | "x" | chr11:47894562-47898879 | 2.22318 | 3.64777 |
| lncRNA10411 | "x" | chr02:38820568-38822023 | 1.00831 | 2.62318 |
| lncRNA26882 | "x" | chr06:44342665-44357079 | 6.47516 | 4.87225 |
| lncRNA45902 | "x" | chr12:47426292-47430878 | 96.2767 | 133.883 |
| lncRNA35589 | "x" | chr09:220543-223678 | 4.40615 | 8.80119 |
| lncRNA06996 | "u" | chr02:13876376-13877185 | 8.02338 | 6.15553 |
| lncRNA14382 | "u" | chr03:18735420-18736175 | 1.71777 | 2.53816 |
| lncRNA40615 | "x" | chr10:61775434-61781369 | 6.01075 | 2.6762 |
| lncRNA20446 | "x" | chr05:8246529-8250265 | 0.432045 | 1.26158 |
| lncRNA15612 | "x" | chr03:59971464-59974718 | 1.40293 | 0.993734 |
| lncRNA25149 | "x" | chr06:192107-195349 | 1.59969 | 7.44446 |
| lncRNA44637 | "x" | chr12:41550-45922 | 2.02748 | 2.99665 |
| lncRNA33917 | "u" | chr09:757092-757427 | 68.8934 | 52.1214 |
| lncRNA36121 | "u" | chr09:14240838-14246969 | 1.50917 | 1.21535 |
| lncRNA46400 | "x" | chr12:64627946-64630059 | 2.13493 | 1.07188 |
| lncRNA48079 | "x" | chr12:62852278-62855348 | 1.22828 | 2.22348 |
| lncRNA13977 | "u" | chr03:7172898-7173218 | 47.1977 | 33.4065 |
| lncRNA21902 | "x" | chr05:5212784-5216258 | 3.09279 | 0.998908 |
| lncRNA12622 | "x" | chr03:49196468-49200370 | 2.38038 | 3.13623 |
| lncRNA44489 | "x" | chr11:52204799-52209357 | 1.60824 | 2.30693 |
| lncRNA43834 | "x" | chr11:34413850-34416945 | 1.92272 | 0.928432 |
| lncRNA34793 | "x" | chr09:54667102-54667837 | 2.36652 | 1.54792 |
| lncRNA06860 | "u" | chr02:2108614-2109245 | 3.56397 | 2.4471 |
| lncRNA42847 | "x" | chr11:304293-306427 | 12.3826 | 20.8333 |
| lncRNA38467 | "x" | chr10:59069860-59075366 | 2.12823 | 0.962118 |
| lncRNA31244 | "x" | chr08:50036213-50037316 | 4.24237 | 7.86343 |
| lncRNA37261 | "x" | chr09:67518616-67521779 | 1.48592 | 1.09033 |
| lncRNA47097 | "x" | chr12:6620090-6623212 | 0.661273 | 1.39347 |
| lncRNA15799 | "u" | chr03:61980530-61983314 | 1.78842 | 1.37793 |
| lncRNA26717 | "x" | chr06:42537441-42538066 | 2.37238 | 1.57457 |
| lncRNA17175 | "u" | chr04:54317378-54319294 | 1.08634 | 1.4512 |
| lncRNA21919 | "x" | chr05:5500850-5506199 | 1.34039 | 0.648218 |
| lncRNA36827 | "u" | chr09:62024843-62049150 | 1.39643 | 0.72654 |
| lncRNA08056 | "u" | chr02:39028342-39029886 | 2.95979 | 3.79014 |
| lncRNA19716 | "u" | chr04:62111905-62112970 | 1.47908 | 1.00507 |
| lncRNA38404 | "x" | chr10:57961335-57966227 | 15.2497 | 27.9198 |
| lncRNA02810 | "u" | chr01:77836348-77842781 | 9.86314 | 2.82914 |
| lncRNA06993 | "x" | chr02:13682622-13683769 | 6.30996 | 3.29348 |
| lncRNA00483 | "u" | chr00:20042693-20043087 | 5.20358 | 2.99163 |
| lncRNA30300 | "u" | chr07:63636632-63637114 | 4.57937 | 2.92406 |
| lncRNA31481 | "u" | chr08:54892322-54892736 | 4.27392 | 2.48002 |
| lncRNA11159 | "u" | chr02:46621049-46622280 | 1.08869 | 1.55237 |
| lncRNA23222 | "u" | chr06:695956-708656 | 1.57873 | 2.08244 |
| lncRNA42369 | "x" | chr11:48296987-48301779 | 6.56747 | 4.73297 |
| lncRNA22993 | "j" | chr05:63111560-63124254 | 0.635648 | 1.08142 |
| lncRNA47531 | "x" | chr12:39489011-39492283 | 1.05539 | 2.03194 |
| lncRNA33849 | "x" | chr09:156252-158894 | 0.62617 | 1.03796 |
| lncRNA46677 | "x" | chr12:1555499-1559940 | 0.997551 | 1.71547 |
| lncRNA25360 | "x" | chr06:2680329-2681566 | 46.1674 | 38.1462 |
| lncRNA36682 | "x" | chr09:59328415-59334228 | 2.12317 | 0.686141 |
| lncRNA44182 | "x" | chr11:48570084-48574651 | 6.06786 | 4.306 |
| lncRNA15471 | "x" | chr03:58611729-58624729 | 2.29309 | 3.74098 |
| lncRNA42967 | "x" | chr11:1397412-1400486 | 7.32136 | 4.54822 |
| lncRNA11808 | "x" | chr03:8056251-8061491 | 11.6114 | 16.8074 |
| lncRNA35843 | "x" | chr09:3521392-3529854 | 0.836881 | 0.563153 |
| lncRNA19103 | "u" | chr04:53655805-53656099 | 36.2986 | 53.6193 |
| lncRNA14316 | "x" | chr03:14875452-14876553 | 8.79396 | 13.218 |
| lncRNA36122 | "u" | chr09:14247545-14254111 | 1.50673 | 1.22518 |
| lncRNA24802 | "x" | chr06:42477946-42480623 | 3.23877 | 4.64896 |
| lncRNA36627 | "x" | chr09:57914679-57927124 | 1.33888 | 0.708231 |
| lncRNA43871 | "x" | chr11:35459459-35463195 | 0.885693 | 1.1468 |
| lncRNA46539 | "x" | chr12:172116-176753 | 7.6358 | 15.0033 |
| lncRNA01463 | "u" | chr01:6475474-6477458 | 0.963265 | 0.685444 |
| lncRNA26467 | "x" | chr06:39775281-39790838 | 1.46377 | 0.794547 |
| lncRNA36823 | "x" | chr09:62019632-62020496 | 3.39995 | 4.53756 |
| lncRNA42662 | "x" | chr11:51851247-51854715 | 3.19348 | 1.74091 |
| lncRNA30647 | "x" | chr08:2191585-2194527 | 1.26679 | 0.818876 |
| lncRNA42595 | "x" | chr11:51085807-51086839 | 2.45102 | 1.73023 |
| lncRNA04244 | "x" | chr01:3253258-3262099 | 15.9277 | 8.01165 |
| lncRNA46467 | "x" | chr12:65139387-65148741 | 2.70621 | 3.78841 |
| lncRNA01941 | "x" | chr01:53410401-53419907 | 2.86245 | 4.02412 |
| lncRNA06495 | "x" | chr01:86773451-86774482 | 2.23122 | 3.95527 |
| lncRNA28972 | "x" | chr07:2738672-2742284 | 1.37485 | 0.880599 |
| lncRNA25186 | "x" | chr06:671167-671961 | 1.4066 | 0.926021 |
| lncRNA35016 | "x" | chr09:60789849-60793229 | 2.78464 | 1.87588 |
| lncRNA29325 | "x" | chr07:29734564-29737794 | 3.24201 | 3.95879 |
| lncRNA26068 | "x" | chr06:35166186-35177784 | 3.15791 | 2.36472 |
| lncRNA22874 | "x" | chr05:61543528-61544549 | 18.2763 | 31.2322 |
| lncRNA20261 | "x" | chr05:4748493-4753142 | 2.11035 | 7.29027 |
| lncRNA28326 | "x" | chr07:60697003-60702293 | 1.165 | 0.677596 |
| lncRNA38931 | "x" | chr10:63549666-63554014 | 6.85212 | 3.33901 |
| lncRNA01380 | "u" | chr01:3990900-3991349 | 4.22255 | 2.53936 |
| lncRNA02536 | "u" | chr01:74148029-74152050 | 6.37036 | 5.32936 |
| lncRNA16966 | "x" | chr04:48789655-48790602 | 0.611415 | 1.03232 |
| lncRNA38325 | "u" | chr10:52781255-52783700 | 0.79574 | 1.07104 |
| lncRNA41462 | "x" | chr11:5141286-5145280 | 1.24102 | 0.790858 |
| lncRNA15341 | "x" | chr03:57094202-57100243 | 2.39598 | 1.44971 |
| lncRNA27061 | "x" | chr07:101156-103579 | 1.82797 | 0.979472 |
| lncRNA31950 | "x" | chr08:61001511-61006067 | 2.17893 | 0.865301 |
| lncRNA47175 | "u" | chr12:10642021-10643491 | 1.11227 | 0.775826 |
| lncRNA43790 | "x" | chr11:29161878-29170962 | 4.81886 | 3.18344 |
| lncRNA31645 | "x" | chr08:57411106-57413970 | 27.6361 | 11.8899 |
| lncRNA41617 | "x" | chr11:8039102-8042521 | 2.94293 | 6.22442 |
| lncRNA26849 | "x" | chr06:43965639-43970354 | 1.02054 | 2.64202 |
| lncRNA20117 | "x" | chr05:2698892-2703279 | 3.97513 | 1.48133 |
| lncRNA02942 | "u" | chr01:79352361-79352687 | 53.5058 | 39.421 |
| lncRNA38392 | "u" | chr10:57748719-57753550 | 3.72084 | 4.46104 |
| lncRNA16563 | "x" | chr04:7364545-7369208 | 3.78044 | 2.89461 |
| lncRNA20105 | "x" | chr05:2521247-2521858 | 11.3308 | 5.64002 |
| lncRNA42646 | "x" | chr11:51693103-51699612 | 5.17871 | 8.09848 |
| lncRNA24705 | "u" | chr06:41441967-41442502 | 3.39897 | 2.19828 |
| lncRNA44467 | "x" | chr11:51930685-51936424 | 0.739955 | 0.434294 |
| lncRNA40104 | "x" | chr10:51490279-51501790 | 4.96333 | 9.53005 |
| lncRNA43256 | "x" | chr11:4586350-4609181 | 6.32282 | 5.10693 |
| lncRNA06275 | "u" | chr01:84369668-84369916 | 48.3827 | 26.7621 |
| lncRNA31310 | "x" | chr08:51675914-51687612 | 0.870551 | 1.50051 |
| lncRNA21165 | "x" | chr05:60421917-60429626 | 5.31194 | 10.0063 |
| lncRNA40476 | "x" | chr10:60442709-60445947 | 8.33574 | 13.9742 |
| lncRNA21265 | "x" | chr05:61892538-61895062 | 11.636 | 25.4421 |
| lncRNA23744 | "x" | chr06:25335562-25347915 | 8.27283 | 11.3536 |
| lncRNA36357 | "u" | chr09:42006479-42009789 | 1.17654 | 1.50451 |
| lncRNA02618 | "x" | chr01:75132657-75135971 | 3.96443 | 6.35918 |
| lncRNA42516 | "x" | chr11:50278079-50280307 | 3.30274 | 4.12513 |
| lncRNA23221 | "x" | chr06:695956-708656 | 1.38536 | 0.554199 |
| lncRNA26060 | "x" | chr06:35076832-35087425 | 0.887343 | 1.62741 |
| lncRNA17478 | "x" | chr04:58926721-58932414 | 1.70524 | 0.733579 |
| lncRNA18036 | "x" | chr04:1283913-1287795 | 1.36759 | 2.50132 |
| lncRNA09369 | "u" | chr02:17950503-17953031 | 3.80417 | 3.08046 |
| lncRNA15979 | "x" | chr03:63958574-63962297 | 1.22304 | 0.895441 |
| lncRNA43372 | "x" | chr11:5850496-5854386 | 2.27891 | 1.11399 |
| lncRNA04861 | "u" | chr01:57540605-57540831 | 180.779 | 117.04 |
| lncRNA47731 | "x" | chr12:46337178-46339981 | 4.24252 | 8.04949 |
| lncRNA15818 | "x" | chr03:62183746-62189147 | 1.77148 | 0.9081 |
| lncRNA18559 | "u" | chr04:18279635-18281693 | 11.9388 | 10.015 |
| lncRNA40872 | "x" | chr10:63963192-63966253 | 8.86051 | 6.36266 |
| lncRNA40599 | "x" | chr10:61621968-61626173 | 3.46186 | 2.15808 |
| lncRNA47565 | "x" | chr12:42592137-42594528 | 0.951633 | 1.58396 |
| lncRNA15151 | "x" | chr03:53815620-53822753 | 0.825093 | 1.46875 |
| lncRNA40993 | "x" | chr11:167627-178529 | 0.819235 | 0.505686 |
| lncRNA46520 | "u" | chr12:35174-36330 | 0.845497 | 0.549909 |
| lncRNA27618 | "u" | chr07:27031851-27032162 | 85.201 | 110.501 |
| lncRNA45390 | "x" | chr12:20128895-20139142 | 7.66495 | 12.0782 |
| lncRNA38240 | "x" | chr10:50988642-50990442 | 1.37526 | 2.06041 |
| lncRNA28633 | "u" | chr07:64056380-64057391 | 1.14985 | 0.749936 |
| lncRNA31949 | "u" | chr08:61000217-61000564 | 14.2172 | 9.27388 |
| lncRNA18374 | "u" | chr04:5632498-5632889 | 31.9149 | 41.211 |
| lncRNA22135 | "u" | chr05:9820015-9822414 | 2.95484 | 3.65344 |
| lncRNA22606 | "x" | chr05:53328282-53331147 | 2.67428 | 9.42851 |
| lncRNA41216 | "x" | chr11:2288655-2290634 | 2.07342 | 1.38074 |
| lncRNA14170 | "u" | chr03:9701804-9702242 | 4.93189 | 3.27853 |
| lncRNA40856 | "x" | chr10:63843259-63848333 | 9.43363 | 6.33102 |
| lncRNA05226 | "u" | chr01:71068025-71069417 | 4.41402 | 3.47446 |
| lncRNA23449 | "u" | chr06:4553716-4558176 | 0.866568 | 1.60293 |
| lncRNA06369 | "x" | chr01:85388618-85395969 | 2.76916 | 1.49478 |
| lncRNA03680 | "x" | chr01:86945728-86948859 | 0.458417 | 0.871529 |
| lncRNA15862 | "x" | chr03:62690113-62690700 | 2.62344 | 4.19551 |
| lncRNA14820 | "x" | chr03:46522037-46533607 | 1.15253 | 1.41707 |
| lncRNA28666 | "x" | chr07:64392364-64397327 | 1.14151 | 0.649729 |
| lncRNA19881 | "u" | chr04:63805465-63805793 | 24.9369 | 16.9889 |
| lncRNA25919 | "u" | chr06:32743936-32744632 | 2.64774 | 1.80443 |
| lncRNA09657 | "u" | chr02:29705981-29707155 | 1.52446 | 1.06961 |
| lncRNA31598 | "x" | chr08:56838046-56842836 | 13.6475 | 10.1801 |
| lncRNA31488 | "x" | chr08:55111271-55112078 | 120.438 | 48.2985 |
| lncRNA42314 | "x" | chr11:47430898-47434506 | 4.36853 | 6.67277 |
| lncRNA03985 | "x" | chr01:280018-283591 | 2.58665 | 1.50212 |
| lncRNA10216 | "x" | chr02:36499353-36503211 | 1.46363 | 2.20111 |
| lncRNA24639 | "u" | chr06:40844676-40846113 | 2.00232 | 2.60771 |
| lncRNA16832 | "u" | chr04:33415303-33416660 | 2.45199 | 1.86277 |
| lncRNA24319 | "x" | chr06:37352390-37356280 | 0.609566 | 0.31919 |
| lncRNA08758 | "x" | chr02:46214573-46218934 | 7.20607 | 4.14331 |
| lncRNA31256 | "x" | chr08:50427387-50431295 | 11.6048 | 9.22666 |
| lncRNA18869 | "u" | chr04:44936880-44937443 | 8.67 | 6.56923 |
| lncRNA44853 | "x" | chr12:1952583-1953085 | 288.265 | 235.672 |
| lncRNA27295 | "x" | chr07:3375303-3379162 | 5.96094 | 8.84652 |
| lncRNA01725 | "u" | chr01:29998396-29999072 | 2.71669 | 1.85478 |
| lncRNA17981 | "x" | chr04:582016-585430 | 0.852712 | 0.467431 |
| lncRNA15103 | "x" | chr03:53176704-53177646 | 4.78103 | 3.66272 |
| lncRNA38766 | "x" | chr10:61859170-61862831 | 6.36011 | 8.89878 |
| lncRNA18845 | "x" | chr04:42472205-42473738 | 4.96822 | 10.7719 |
| lncRNA26174 | "x" | chr06:36393611-36399129 | 2.82763 | 1.64149 |
| lncRNA43784 | "u" | chr11:28480554-28481002 | 62.0441 | 50.2835 |
| lncRNA10199 | "u" | chr02:36331998-36332423 | 3.06012 | 1.70435 |
| lncRNA18410 | "u" | chr04:6638114-6638535 | 3.20175 | 1.78493 |
| lncRNA36719 | "x" | chr09:60142354-60147548 | 0.947275 | 1.71247 |
| lncRNA01397 | "u" | chr01:4572408-4572757 | 7.50991 | 4.23361 |
| lncRNA44741 | "x" | chr12:818592-819454 | 15.3864 | 39.6348 |
| lncRNA08812 | "x" | chr02:46833949-46842776 | 1.03099 | 1.30273 |
| lncRNA13231 | "x" | chr03:59932808-59936469 | 6.20195 | 3.66551 |
| lncRNA24445 | "u" | chr06:38849327-38849886 | 13.1434 | 10.1709 |
| lncRNA19958 | "x" | chr05:509428-510476 | 9.79391 | 7.08523 |
| lncRNA34449 | "x" | chr09:16673366-16678758 | 0.758448 | 1.59642 |
| lncRNA37972 | "x" | chr10:36109779-36114394 | 5.05141 | 10.0178 |
| lncRNA15855 | "x" | chr03:62621403-62626450 | 1.33979 | 1.92528 |
| lncRNA46849 | "x" | chr12:3406946-3409965 | 2.66687 | 1.75295 |
| lncRNA38369 | "x" | chr10:57382489-57385238 | 0.815692 | 1.27154 |
| lncRNA40412 | "x" | chr10:59847570-59851730 | 3.11187 | 4.71091 |
| lncRNA13313 | "x" | chr03:60775353-60776507 | 10.5014 | 6.48799 |
| lncRNA05442 | "x" | chr01:74383078-74384666 | 4.14403 | 9.79877 |
| lncRNA30188 | "x" | chr07:62431981-62433201 | 9.20427 | 6.36926 |
| lncRNA30348 | "u" | chr07:64188642-64188907 | 88.829 | 122.349 |
| lncRNA45353 | "x" | chr12:15120632-15133486 | 1.54541 | 1.0393 |
| lncRNA44452 | "x" | chr11:51758656-51767705 | 0.523481 | 1.13446 |
| lncRNA27628 | "x" | chr07:28770989-28778367 | 0.778322 | 0.384566 |
| lncRNA05215 | "x" | chr01:70898329-70904143 | 0.867941 | 0.528006 |
| lncRNA16187 | "x" | chr04:1682463-1697247 | 0.845683 | 0.494031 |
| lncRNA30715 | "x" | chr08:3170641-3174481 | 2.23232 | 3.70072 |
| lncRNA02388 | "u" | chr01:72197786-72199295 | 1.23502 | 1.6525 |
| lncRNA12536 | "x" | chr03:47036391-47037894 | 8.29682 | 4.86122 |
| lncRNA14305 | "x" | chr03:14424753-14429593 | 1.26856 | 2.28716 |
| lncRNA07493 | "x" | chr02:32266397-32268537 | 7.32762 | 8.83073 |
| lncRNA35194 | "u" | chr09:63551357-63552865 | 1.42286 | 1.03527 |
| lncRNA20691 | "x" | chr05:28060780-28074769 | 82.3939 | 52.7187 |
| lncRNA35827 | "x" | chr09:3409649-3412273 | 6.00453 | 8.48652 |
| lncRNA02628 | "x" | chr01:75275582-75278282 | 6.34674 | 2.54464 |
| lncRNA43439 | "x" | chr11:7660094-7671679 | 17.8032 | 23.9402 |
| lncRNA46798 | "x" | chr12:2884182-2891031 | 6.18067 | 3.94655 |
| lncRNA37064 | "x" | chr09:65118729-65121622 | 2.71985 | 1.40918 |
| lncRNA30901 | "x" | chr08:17265935-17275353 | 0.800739 | 0.549259 |
| lncRNA09526 | "x" | chr02:25746531-25749237 | 7.15383 | 5.97522 |
| lncRNA30217 | "x" | chr07:62742398-62770824 | 8.18603 | 5.38458 |
| lncRNA29350 | "x" | chr07:32765263-32781498 | 66.6035 | 91.0998 |
| lncRNA38687 | "x" | chr10:61117052-61119915 | 2.34855 | 4.19293 |
| lncRNA38169 | "x" | chr10:47366113-47373268 | 10.2307 | 14.1526 |
| lncRNA41214 | "x" | chr11:2273074-2275000 | 1.57925 | 2.51801 |
| lncRNA06197 | "x" | chr01:83415884-83422712 | 2.44504 | 0.792489 |
| lncRNA41080 | "x" | chr11:1009488-1012021 | 2.14626 | 4.5158 |
| lncRNA41804 | "x" | chr11:14688574-14691058 | 4.5203 | 3.13966 |
| lncRNA47647 | "x" | chr12:44345438-44347389 | 13.5568 | 16.4525 |
| lncRNA24816 | "x" | chr06:42609069-42616109 | 3.06467 | 3.99721 |
| lncRNA18337 | "x" | chr04:5152383-5153872 | 21.9111 | 16.8246 |
| lncRNA23855 | "x" | chr06:30082684-30084651 | 3.56583 | 6.4623 |
| lncRNA10041 | "u" | chr02:34599597-34599956 | 11.2839 | 7.2145 |
| lncRNA18134 | "x" | chr04:2646822-2651089 | 3.1671 | 2.41755 |
| lncRNA44366 | "x" | chr11:50820289-50833473 | 0.905084 | 1.09048 |
| lncRNA42854 | "x" | chr11:374638-378429 | 0.852222 | 1.72815 |
| lncRNA09223 | "x" | chr02:10951105-10953091 | 1.04564 | 0.753422 |
| lncRNA29950 | "x" | chr07:59810020-59812746 | 4.33384 | 2.46351 |
| lncRNA29975 | "x" | chr07:60020590-60026247 | 3.40149 | 2.83426 |
| lncRNA44782 | "x" | chr12:1220889-1223936 | 3.67561 | 10.5462 |
| lncRNA02741 | "x" | chr01:76945254-76949653 | 0.819833 | 1.35617 |
| lncRNA15029 | "x" | chr03:51603133-51604309 | 8.89339 | 6.18502 |
| lncRNA17228 | "x" | chr04:55013507-55018537 | 3.03308 | 2.01574 |
| lncRNA08002 | "x" | chr02:38397079-38401238 | 5.55977 | 3.09673 |
| lncRNA42980 | "x" | chr11:1485190-1486454 | 1.16129 | 1.81367 |
| lncRNA43304 | "x" | chr11:5031093-5036032 | 1.28396 | 0.706315 |
| lncRNA06194 | "x" | chr01:83364522-83371065 | 2.11004 | 0.648754 |
| lncRNA28365 | "x" | chr07:61203404-61207625 | 0.781392 | 0.397082 |
| lncRNA46667 | "x" | chr12:1441032-1446614 | 2.28638 | 1.71868 |
| lncRNA36226 | "x" | chr09:24965632-24969681 | 0.55684 | 0.721387 |
| lncRNA05059 | "x" | chr01:67387972-67396607 | 1.79571 | 0.773175 |
| lncRNA12845 | "x" | chr03:54202387-54206465 | 0.496784 | 0.947758 |
| lncRNA39814 | "x" | chr10:37017563-37020617 | 8.26791 | 6.44788 |
| lncRNA06640 | "x" | chr01:88288062-88291766 | 4.80985 | 1.77762 |
| lncRNA01832 | "u" | chr01:42730889-42733061 | 0.969836 | 0.720027 |
| lncRNA19992 | "u" | chr05:881725-882203 | 8.32719 | 11.215 |
| lncRNA27495 | "x" | chr07:10515449-10530682 | 1.47614 | 1.23062 |
| lncRNA11838 | "x" | chr03:8594585-8595975 | 4.95965 | 7.85263 |
| lncRNA00270 | "u" | chr00:13375065-13375684 | 6.33983 | 8.29605 |
| lncRNA33554 | "x" | chr08:59615830-59617757 | 18.1672 | 24.1028 |
| lncRNA22268 | "u" | chr05:19312525-19316891 | 3.30012 | 2.7622 |
| lncRNA22952 | "x" | chr05:62542765-62558988 | 103.341 | 68.7307 |
| lncRNA30191 | "x" | chr07:62443566-62447482 | 0.622124 | 1.14234 |
| lncRNA12258 | "x" | chr03:30892042-30896175 | 1.2169 | 1.63627 |
| lncRNA09378 | "x" | chr02:18711442-18715788 | 16.0536 | 10.8893 |
| lncRNA47443 | "u" | chr12:35551160-35556152 | 1.61979 | 1.31246 |
| lncRNA46349 | "x" | chr12:64169263-64170041 | 2.73933 | 4.44294 |
| lncRNA42723 | "x" | chr11:52385491-52392740 | 2.8487 | 4.60604 |
| lncRNA15888 | "x" | chr03:62997550-63005605 | 1.49666 | 2.038 |
| lncRNA00041 | "u" | chr00:6386510-6386766 | 312.292 | 398.474 |
| lncRNA29681 | "x" | chr07:55815202-55820658 | 3.36795 | 2.60086 |
| lncRNA43331 | "x" | chr11:5312805-5318598 | 4.60831 | 2.62433 |
| lncRNA19801 | "x" | chr04:63032062-63035236 | 3.25105 | 7.07921 |
| lncRNA15496 | "u" | chr03:58883467-58883901 | 5.18429 | 3.34815 |
| lncRNA15590 | "x" | chr03:59701532-59704016 | 4.36617 | 1.7823 |
| lncRNA38933 | "x" | chr10:63559777-63564969 | 4.89636 | 7.68995 |
| lncRNA26872 | "x" | chr06:44215179-44219042 | 0.891174 | 0.383708 |
| lncRNA44499 | "x" | chr11:52259862-52265018 | 2.84111 | 1.72748 |
| lncRNA22009 | "x" | chr05:6701172-6717388 | 3.16444 | 1.67959 |
| lncRNA25718 | "u" | chr06:27654465-27654942 | 20.8075 | 25.9169 |
| lncRNA46188 | "x" | chr12:62708821-62712424 | 19.8943 | 31.7667 |
| lncRNA15443 | "x" | chr03:58333901-58336148 | 6.48691 | 4.2511 |
| lncRNA43797 | "x" | chr11:30324878-30326006 | 0.86957 | 1.38222 |
| lncRNA35432 | "x" | chr09:66354921-66361692 | 4.78975 | 3.30214 |
| lncRNA45647 | "u" | chr12:39949469-39951438 | 1.15208 | 0.859549 |
| lncRNA30189 | "x" | chr07:62434831-62439287 | 4.01185 | 1.87337 |
| lncRNA38929 | "x" | chr10:63540039-63542488 | 2.43338 | 3.01917 |
| lncRNA17591 | "x" | chr04:60394501-60411312 | 30.6708 | 16.902 |
| lncRNA17896 | "x" | chr04:63915914-63921381 | 4.44432 | 5.42861 |
| lncRNA40484 | "u" | chr10:60562515-60563464 | 1.05368 | 0.683935 |
| lncRNA10749 | "x" | chr02:42494622-42496760 | 1.59943 | 1.02015 |
| lncRNA31709 | "x" | chr08:58360689-58361722 | 2.89839 | 3.98487 |
| lncRNA48235 | "x" | chr12:64210088-64212694 | 5.60482 | 4.48799 |
| lncRNA38814 | "u" | chr10:62335909-62336480 | 4.35222 | 5.96472 |
| lncRNA05576 | "x" | chr01:76114873-76119835 | 1.60631 | 2.3544 |
| lncRNA01275 | "u" | chr01:2263625-2264065 | 14.3 | 10.6308 |
| lncRNA34966 | "x" | chr09:59719772-59727045 | 3.44572 | 2.25334 |
| lncRNA14086 | "u" | chr03:8605245-8606013 | 1.42963 | 0.930502 |
| lncRNA08711 | "x" | chr02:45748521-45750494 | 5.71959 | 8.11288 |
| lncRNA42009 | "u" | chr11:32273832-32275038 | 1.7987 | 1.31604 |
| lncRNA33219 | "u" | chr08:55156337-55157116 | 1.29152 | 0.859115 |
| lncRNA23007 | "x" | chr05:63232819-63233438 | 6.34862 | 10.5765 |
| lncRNA11257 | "u" | chr02:47551454-47551850 | 12.3854 | 8.83693 |
| lncRNA23431 | "x" | chr06:3832407-3833208 | 13.68 | 7.64147 |
| lncRNA33834 | "x" | chr08:62996570-63000055 | 5.58078 | 9.17636 |
| lncRNA05407 | "x" | chr01:73938192-73944527 | 1.43649 | 2.26167 |
| lncRNA35669 | "u" | chr09:1006614-1020453 | 1.59276 | 0.685483 |
| lncRNA17189 | "u" | chr04:54463874-54464758 | 1.99799 | 2.72396 |
| lncRNA47424 | "x" | chr12:34144339-34145207 | 3.55851 | 4.65129 |
| lncRNA10841 | "j" | chr02:43425412-43431403 | 1.40566 | 0.760911 |
| lncRNA10720 | "u" | chr02:42116843-42118123 | 1.88956 | 1.41634 |
| lncRNA21073 | "u" | chr05:58485039-58485850 | 4.95086 | 6.31577 |
| lncRNA16986 | "x" | chr04:49629976-49632768 | 1.52105 | 1.19616 |
| lncRNA28464 | "x" | chr07:62388556-62392637 | 3.84674 | 7.05816 |
| lncRNA43358 | "u" | chr11:5593816-5594839 | 1.76863 | 2.39237 |
| lncRNA46210 | "x" | chr12:63006623-63010272 | 0.514557 | 1.11361 |
| lncRNA42021 | "u" | chr11:34115039-34119710 | 0.919572 | 1.14908 |
| lncRNA29865 | "x" | chr07:58637129-58639511 | 17.2992 | 12.2714 |
| lncRNA30262 | "x" | chr07:63208354-63212096 | 2.43068 | 4.42068 |
| lncRNA02525 | "x" | chr01:74052768-74059391 | 1.34795 | 1.10202 |
| lncRNA47357 | "x" | chr12:29623118-29632342 | 1.01652 | 0.614668 |
| lncRNA19611 | "x" | chr04:61113343-61120190 | 1.26319 | 1.92022 |
| lncRNA04607 | "u" | chr01:25841698-25843792 | 0.7644 | 0.459544 |
| lncRNA23371 | "x" | chr06:2801289-2806168 | 0.390001 | 0.758802 |
| lncRNA23630 | "x" | chr06:20724112-20724819 | 1.2068 | 0.697566 |
| lncRNA34921 | "x" | chr09:59029954-59030878 | 4.88474 | 3.47425 |
| lncRNA44913 | "x" | chr12:2510165-2516230 | 2.3548 | 4.57653 |
| lncRNA48167 | "x" | chr12:63618191-63620840 | 5.27663 | 9.38291 |
| lncRNA15592 | "u" | chr03:59740602-59741920 | 1.80704 | 2.34442 |
| lncRNA15359 | "x" | chr03:57346639-57347674 | 25.4646 | 38.475 |
| lncRNA15716 | "x" | chr03:61152621-61154175 | 1.2569 | 0.892257 |
| lncRNA33819 | "x" | chr08:62824726-62826291 | 2.22123 | 1.54865 |
| lncRNA33940 | "x" | chr09:978550-981575 | 5.04909 | 10.1904 |
| lncRNA37607 | "x" | chr10:4506385-4514163 | 0.602739 | 0.275672 |
| lncRNA06885 | "u" | chr02:6334652-6346940 | 1.33824 | 0.708805 |
| lncRNA03117 | "x" | chr01:81210417-81217602 | 0.544119 | 1.02669 |
| lncRNA38500 | "x" | chr10:59372040-59377866 | 0.601719 | 0.298852 |
| lncRNA38772 | "x" | chr10:61903925-61910472 | 1.03979 | 2.20138 |
| lncRNA31493 | "x" | chr08:55135932-55140666 | 0.642719 | 0.427289 |
| lncRNA32487 | "x" | chr08:6646726-6657472 | 14.1269 | 11.4561 |
| lncRNA42640 | "x" | chr11:51669431-51673400 | 1.08518 | 0.375672 |
| lncRNA29892 | "x" | chr07:59110487-59116171 | 4.36049 | 3.32281 |
| lncRNA45949 | "x" | chr12:48016541-48021020 | 17.0355 | 9.43511 |
| lncRNA39750 | "x" | chr10:30297710-30305111 | 6.48637 | 8.72481 |
| lncRNA26728 | "u" | chr06:42686359-42688403 | 1.20464 | 0.923382 |
| lncRNA19885 | "x" | chr04:63828696-63835334 | 4.28213 | 2.90708 |
| lncRNA23118 | "x" | chr05:64437169-64441321 | 4.03286 | 2.33786 |
| lncRNA35712 | "x" | chr09:1499864-1504313 | 1.00397 | 1.54493 |
| lncRNA38934 | "x" | chr10:63559777-63564969 | 5.17444 | 6.39264 |
| lncRNA20170 | "x" | chr05:3251898-3264160 | 2.65811 | 1.74871 |
| lncRNA12919 | "x" | chr03:56476472-56478792 | 173.324 | 114.678 |
| lncRNA33480 | "u" | chr08:58762429-58763108 | 2.35293 | 1.62329 |
| lncRNA16435 | "x" | chr04:4729012-4732903 | 3.05495 | 0.993857 |
| lncRNA28876 | "u" | chr07:1475187-1476519 | 5.85971 | 4.7592 |
| lncRNA46768 | "x" | chr12:2537940-2545182 | 1.49482 | 0.839467 |
| lncRNA47952 | "x" | chr12:57421692-57431892 | 0.683043 | 0.3863 |
| lncRNA11528 | "u" | chr03:561769-562165 | 40.6484 | 50.6006 |
| lncRNA40262 | "x" | chr10:57977792-57986678 | 41.0567 | 23.795 |
| lncRNA19077 | "x" | chr04:53261056-53262118 | 9.87261 | 12.4708 |
| lncRNA25417 | "x" | chr06:3608114-3612636 | 1.79482 | 1.30808 |
| lncRNA08944 | "x" | chr02:48245177-48246752 | 2.22782 | 1.70153 |
| lncRNA41074 | "x" | chr11:924276-930481 | 2.92551 | 1.43538 |
| lncRNA01218 | "x" | chr01:1846092-1847506 | 1.54163 | 2.53005 |
| lncRNA28660 | "x" | chr07:64353391-64359178 | 12.781 | 7.85754 |
| lncRNA26009 | "x" | chr06:34103560-34110110 | 4.61516 | 3.08094 |
| lncRNA17245 | "u" | chr04:55463112-55464348 | 8.93081 | 10.7346 |
| lncRNA01431 | "j" | chr01:5360137-5364988 | 1.64564 | 3.91435 |
| lncRNA02532 | "x" | chr01:74097195-74105740 | 2.3912 | 1.88062 |
| lncRNA48367 | "x" | chr12:65238625-65241667 | 1.71445 | 2.61452 |
| lncRNA13848 | "x" | chr03:1938145-1944988 | 0.373204 | 0.963988 |
| lncRNA41627 | "x" | chr11:8198128-8200893 | 7.95559 | 9.46279 |
| lncRNA32512 | "x" | chr08:7411935-7414293 | 2.85228 | 3.4713 |
| lncRNA22084 | "x" | chr05:8209488-8211818 | 0.857851 | 1.12186 |
| lncRNA32091 | "u" | chr08:62561432-62566129 | 3.16181 | 2.35482 |
| lncRNA37156 | "x" | chr09:66227326-66243063 | 2.81573 | 1.49484 |
| lncRNA26634 | "x" | chr06:41513599-41520853 | 0.675357 | 1.35482 |
| lncRNA42948 | "x" | chr11:1242255-1246945 | 0.58346 | 1.26681 |
| lncRNA39352 | "x" | chr10:3017000-3024548 | 2.43398 | 1.64054 |
| lncRNA19564 | "x" | chr04:60529682-60534831 | 5.77083 | 7.29178 |
| lncRNA09654 | "x" | chr02:29649124-29653417 | 363.064 | 219.29 |
| lncRNA22519 | "x" | chr05:41617825-41620380 | 6.41818 | 7.56588 |
| lncRNA03868 | "u" | chr01:89067713-89068305 | 1.86955 | 1.21124 |
| lncRNA04094 | "u" | chr01:1493365-1494385 | 1.55338 | 2.11085 |
| lncRNA08176 | "u" | chr02:40270355-40270924 | 1.98919 | 1.28888 |
| lncRNA15525 | "x" | chr03:59132508-59135382 | 4.59244 | 5.75918 |
| lncRNA29559 | "u" | chr07:52594212-52595148 | 1.2867 | 1.80877 |
| lncRNA33083 | "u" | chr08:53077566-53078126 | 3.24477 | 4.54268 |
| lncRNA40589 | "u" | chr10:61565400-61566116 | 1.35504 | 0.880911 |
| lncRNA09061 | "u" | chr02:49405977-49406847 | 0.929364 | 0.607555 |
| lncRNA17115 | "u" | chr04:53055479-53056239 | 1.1612 | 0.761221 |
| lncRNA13474 | "x" | chr03:62519740-62521518 | 18.3807 | 31.6173 |
| lncRNA22919 | "x" | chr05:62051691-62054909 | 0.827714 | 0.451887 |
| lncRNA04773 | "x" | chr01:47922647-47930463 | 1.78612 | 1.43529 |
| lncRNA17166 | "x" | chr04:54248695-54262878 | 3.64596 | 2.5794 |
| lncRNA03453 | "x" | chr01:84743806-84751406 | 3.1855 | 6.49778 |
| lncRNA44747 | "u" | chr12:870118-871505 | 6.02488 | 10.4379 |
| lncRNA24135 | "x" | chr06:35097285-35098428 | 19.4688 | 32.996 |
| lncRNA35389 | "x" | chr09:65842512-65846890 | 1.06553 | 0.581747 |
| lncRNA15419 | "x" | chr03:58066644-58067981 | 0.577465 | 1.03513 |
| lncRNA06140 | "x" | chr01:82748478-82754338 | 33.4253 | 43.365 |
| lncRNA19560 | "x" | chr04:60505456-60506566 | 0.806613 | 1.23459 |
| lncRNA31691 | "u" | chr08:58075324-58090632 | 6.0496 | 23.8106 |
| lncRNA15386 | "x" | chr03:57655306-57656461 | 11.5647 | 17.5324 |
| lncRNA10081 | "x" | chr02:35057066-35059876 | 37.6279 | 49.4025 |
| lncRNA46753 | "x" | chr12:2414293-2418504 | 7.71177 | 11.365 |
| lncRNA37535 | "x" | chr10:3060365-3069973 | 0.929295 | 1.17008 |
| lncRNA02344 | "x" | chr01:71266820-71271597 | 0.790218 | 0.487924 |
| lncRNA43323 | "x" | chr11:5226768-5227463 | 11.1575 | 13.7586 |
| lncRNA38942 | "x" | chr10:63653406-63655433 | 2.58077 | 3.58701 |
| lncRNA33410 | "x" | chr08:57799738-57802421 | 1.58678 | 1.05767 |
| lncRNA13386 | "x" | chr03:61578570-61582874 | 0.326466 | 0.600993 |
| lncRNA27895 | "x" | chr07:53663086-53682449 | 1.28731 | 1.75889 |
| lncRNA35726 | "x" | chr09:1671522-1672019 | 7.97921 | 11.3154 |
| lncRNA10276 | "u" | chr02:37332573-37334917 | 5.89571 | 6.96932 |
| lncRNA12014 | "x" | chr03:12859151-12876072 | 6.6638 | 9.49642 |
| lncRNA39831 | "u" | chr10:38304401-38308812 | 5.29161 | 6.21477 |
| lncRNA30243 | "x" | chr07:62992754-62995291 | 1.15096 | 0.781606 |
| lncRNA18822 | "x" | chr04:41008447-41012773 | 0.387227 | 0.668693 |
| lncRNA10940 | "x" | chr02:44476076-44478513 | 13.5181 | 20.4988 |
| lncRNA32268 | "u" | chr08:1473898-1478310 | 0.975999 | 1.20071 |
| lncRNA48042 | "x" | chr12:62423489-62429537 | 4.44757 | 2.5691 |
| lncRNA19307 | "x" | chr04:57116120-57118444 | 2.42624 | 3.23411 |
| lncRNA20733 | "u" | chr05:30205520-30208454 | 8.92785 | 10.4544 |
| lncRNA29917 | "x" | chr07:59441365-59444178 | 1.59416 | 1.16698 |
| lncRNA41223 | "u" | chr11:2414589-2415305 | 28.0203 | 33.2355 |
| lncRNA32160 | "u" | chr08:161520-161891 | 34.574 | 43.6776 |
| lncRNA35119 | "x" | chr09:62390489-62393444 | 1.66378 | 0.979106 |
| lncRNA13070 | "x" | chr03:58189454-58195300 | 1.26636 | 0.780032 |
| lncRNA32348 | "x" | chr08:2534140-2538308 | 1.49049 | 2.02683 |
| lncRNA19154 | "u" | chr04:54556257-54556700 | 5.74778 | 3.95982 |
| lncRNA04082 | "u" | chr01:1365088-1365940 | 1.63113 | 1.1283 |
| lncRNA26547 | "x" | chr06:40781864-40784665 | 11.0347 | 12.8877 |
| lncRNA45104 | "x" | chr12:4532509-4535243 | 5.61183 | 3.87883 |
| lncRNA39963 | "u" | chr10:46355822-46357229 | 2.01056 | 2.57647 |
| lncRNA43930 | "u" | chr11:36707481-36710928 | 7.42698 | 26.8204 |
| lncRNA44060 | "u" | chr11:45867489-45868358 | 9.7996 | 11.9491 |
| lncRNA35066 | "x" | chr09:61655002-61662278 | 6.50113 | 4.95327 |
| lncRNA36034 | "x" | chr09:7176396-7178286 | 2.68457 | 1.38717 |
| lncRNA00623 | "x" | chr00:8777404-8778182 | 33.4086 | 46.0555 |
| lncRNA28812 | "x" | chr07:573549-576970 | 4.18567 | 2.88095 |
| lncRNA45200 | "x" | chr12:5801484-5804416 | 5.35819 | 10.2302 |
| lncRNA02440 | "x" | chr01:72979535-72985606 | 5.41759 | 4.37626 |
| lncRNA46244 | "x" | chr12:63326747-63335562 | 4.47589 | 3.18907 |
| lncRNA21514 | "x" | chr05:64876143-64880531 | 1.31099 | 1.83747 |
| lncRNA08195 | "x" | chr02:40427821-40431629 | 2.24437 | 2.96999 |
| lncRNA29768 | "x" | chr07:57087822-57093362 | 1.57063 | 1.23113 |
| lncRNA26558 | "u" | chr06:40862565-40865640 | 2.06666 | 1.68949 |
| lncRNA30354 | "x" | chr07:64230816-64231279 | 5.79928 | 3.75825 |
| lncRNA10712 | "u" | chr02:42035745-42037694 | 1.14395 | 1.47801 |
| lncRNA45769 | "x" | chr12:44488886-44494577 | 4.99829 | 3.38796 |
| lncRNA35682 | "x" | chr09:1218276-1226403 | 8.80893 | 12.5856 |
| lncRNA11431 | "x" | chr02:49383544-49389074 | 2.55057 | 1.78499 |
| lncRNA21699 | "x" | chr05:2257139-2262966 | 5.84768 | 4.75457 |
| lncRNA03239 | "x" | chr01:82494901-82497982 | 0.835069 | 1.31498 |
| lncRNA15498 | "x" | chr03:58890916-58896673 | 5.99371 | 11.9407 |
| lncRNA42926 | "x" | chr11:1086709-1090759 | 5.1696 | 3.83951 |
| lncRNA06216 | "x" | chr01:83625083-83629542 | 0.625157 | 1.42273 |
| lncRNA12379 | "x" | chr03:44222985-44226714 | 1.98767 | 3.87156 |
| lncRNA01187 | "x" | chr01:1422779-1427208 | 3.09946 | 1.80667 |
| lncRNA19510 | "x" | chr04:59890125-59892898 | 1.86753 | 2.51357 |
| lncRNA46668 | "u" | chr12:1456038-1456937 | 7.94229 | 6.38371 |
| lncRNA19346 | "x" | chr04:57425446-57431154 | 0.44262 | 0.738813 |
| lncRNA38715 | "x" | chr10:61407630-61408277 | 1.12208 | 1.7671 |
| lncRNA44903 | "x" | chr12:2419068-2430753 | 2.24422 | 3.81146 |
| lncRNA10016 | "u" | chr02:34372046-34374118 | 9.62056 | 12.1555 |
| lncRNA23040 | "x" | chr05:63656581-63661919 | 49.0788 | 29.7073 |
| lncRNA24493 | "x" | chr06:39347729-39352677 | 1.93095 | 3.47023 |
| lncRNA26868 | "x" | chr06:44165522-44168095 | 0.483343 | 0.886951 |
| lncRNA28875 | "x" | chr07:1425403-1425989 | 3.16937 | 1.92419 |
| lncRNA38935 | "x" | chr10:63570162-63572223 | 3.38801 | 7.29676 |
| lncRNA15678 | "x" | chr03:60787313-60791258 | 3.26315 | 2.11943 |
| lncRNA33661 | "x" | chr08:61010943-61011980 | 34.2067 | 40.6919 |
| lncRNA12270 | "u" | chr03:31907850-31910082 | 3.06023 | 2.32713 |
| lncRNA00439 | "x" | chr00:18259369-18259577 | 666.094 | 899.796 |
| lncRNA04804 | "x" | chr01:53150488-53153577 | 0.793134 | 1.09924 |
| lncRNA39419 | "x" | chr10:4272368-4276978 | 1.26677 | 1.75068 |
| lncRNA09522 | "u" | chr02:25310920-25311130 | 143.172 | 216.393 |
| lncRNA14178 | "u" | chr03:9731839-9732045 | 148.281 | 224.176 |
| lncRNA19830 | "x" | chr04:63370582-63373715 | 1.03223 | 1.77393 |
| lncRNA34098 | "x" | chr09:3058496-3059804 | 4.97248 | 6.18661 |
| lncRNA35131 | "x" | chr09:62491050-62491784 | 21.4236 | 32.9259 |
| lncRNA22547 | "u" | chr05:46915359-46918261 | 0.982633 | 0.76975 |
| lncRNA45724 | "x" | chr12:43332870-43333843 | 1.74334 | 2.45022 |
| lncRNA09082 | "x" | chr02:49614027-49632724 | 0.909114 | 1.62839 |
| lncRNA47354 | "x" | chr12:29606520-29617960 | 3.11081 | 1.23546 |
| lncRNA15126 | "x" | chr03:53499138-53506306 | 17.8621 | 28.2584 |
| lncRNA40228 | "x" | chr10:57440594-57450199 | 5.01464 | 2.66354 |
| lncRNA44132 | "x" | chr11:47453917-47458321 | 4.32363 | 3.00613 |
| lncRNA18412 | "x" | chr04:6706875-6709744 | 10.7917 | 12.5344 |
| lncRNA10943 | "x" | chr02:44493133-44496742 | 0.197351 | 0.679665 |
| lncRNA07199 | "u" | chr02:21866449-21868178 | 1.87386 | 2.37009 |
| lncRNA24137 | "x" | chr06:35107924-35108465 | 17.2109 | 37.3325 |
| lncRNA31563 | "x" | chr08:56313650-56324720 | 1.56969 | 0.957332 |
| lncRNA17274 | "u" | chr04:55982677-55983412 | 8.19208 | 10.1695 |
| lncRNA23879 | "x" | chr06:30740492-30742107 | 11.1131 | 7.08515 |
| lncRNA40841 | "x" | chr10:63714220-63717812 | 3.98496 | 5.0683 |
| lncRNA44267 | "x" | chr11:49811615-49812791 | 20.2166 | 12.1005 |
| lncRNA48050 | "x" | chr12:62541116-62544192 | 1.30923 | 0.818902 |
| lncRNA18554 | "u" | chr04:18236956-18237702 | 1.9585 | 2.71822 |
| lncRNA38405 | "x" | chr10:57961335-57966227 | 5.66819 | 8.32062 |
| lncRNA41626 | "x" | chr11:8192676-8194719 | 2.72176 | 2.1788 |
| lncRNA29667 | "x" | chr07:55614262-55621499 | 8.17879 | 6.5584 |
| lncRNA40086 | "x" | chr10:51116722-51121737 | 21.2809 | 26.3875 |
| lncRNA37510 | "x" | chr10:2802326-2803208 | 2.34394 | 6.16552 |
| lncRNA14333 | "x" | chr03:15560574-15565116 | 4.84626 | 6.13632 |
| lncRNA37030 | "x" | chr09:64797615-64800559 | 0.898015 | 0.556758 |
| lncRNA39502 | "x" | chr10:7205944-7211854 | 2.30264 | 3.59314 |
| lncRNA45808 | "x" | chr12:45419801-45430613 | 2.76924 | 3.97384 |
| lncRNA34665 | "u" | chr09:43142731-43143474 | 8.36459 | 10.3345 |
| lncRNA30771 | "x" | chr08:4545314-4548052 | 1.56733 | 0.947171 |
| lncRNA27351 | "x" | chr07:4380915-4383225 | 37.7183 | 26.9491 |
| lncRNA28359 | "o" | chr07:61120607-61122920 | 4.18279 | 5.6224 |
| lncRNA46784 | "x" | chr12:2765412-2768037 | 1.9113 | 3.23535 |
| lncRNA02613 | "x" | chr01:75092332-75095495 | 2.56886 | 3.81631 |
| lncRNA14725 | "x" | chr03:45224993-45228588 | 2.05589 | 2.45904 |
| lncRNA17312 | "x" | chr04:56541319-56546303 | 2.73575 | 1.79263 |
| lncRNA30020 | "x" | chr07:60490455-60500283 | 6.08703 | 5.19912 |
| lncRNA26857 | "x" | chr06:44003101-44006569 | 10.0858 | 15.6106 |
| lncRNA20830 | "u" | chr05:41056434-41056983 | 19.2121 | 23.505 |
| lncRNA36624 | "u" | chr09:57833120-57834255 | 0.953448 | 1.50549 |
| lncRNA15416 | "x" | chr03:57991470-57997056 | 1.76754 | 1.19926 |
| lncRNA20513 | "x" | chr05:10832060-10835195 | 0.78318 | 1.34248 |
| lncRNA47900 | "x" | chr12:51909854-51910243 | 32.3868 | 45.0895 |
| lncRNA40432 | "u" | chr10:60039975-60040438 | 214.878 | 185.118 |
| lncRNA27765 | "u" | chr07:46979539-46981371 | 2.46571 | 1.63815 |
| lncRNA10843 | "x" | chr02:43441148-43444337 | 1.14367 | 1.80722 |
| lncRNA43354 | "u" | chr11:5549083-5549618 | 41.2523 | 34.6459 |
| lncRNA13985 | "x" | chr03:7264937-7268774 | 0.526929 | 0.727873 |
| lncRNA47024 | "x" | chr12:5610111-5616726 | 0.442183 | 0.915559 |
| lncRNA08476 | "x" | chr02:43425412-43431403 | 1.35913 | 1.91717 |
| lncRNA07447 | "x" | chr02:31548352-31549876 | 1.3559 | 1.0095 |
| lncRNA10679 | "x" | chr02:41756600-41762425 | 1.4062 | 2.29096 |
| lncRNA19749 | "x" | chr04:62421079-62429518 | 0.678621 | 0.202393 |
| lncRNA31882 | "x" | chr08:60318784-60323467 | 4.10045 | 5.52972 |
| lncRNA22939 | "x" | chr05:62313005-62314056 | 16.0794 | 35.6489 |
| lncRNA42892 | "x" | chr11:762196-766211 | 44.9741 | 27.3974 |
| lncRNA10239 | "x" | chr02:36859618-36863340 | 1.11928 | 1.47778 |
| lncRNA34131 | "u" | chr09:3484423-3487896 | 3.68353 | 3.08159 |
| lncRNA22218 | "x" | chr05:15420451-15425382 | 3.85257 | 3.27124 |
| lncRNA40975 | "x" | chr10:64825438-64829000 | 2.04034 | 1.17894 |
| lncRNA26061 | "x" | chr06:35076832-35087425 | 5.53079 | 9.52312 |
| lncRNA15641 | "x" | chr03:60314445-60322820 | 1.14097 | 0.798307 |
| lncRNA35733 | "x" | chr09:1804837-1810052 | 1.44008 | 1.00415 |
| lncRNA29570 | "x" | chr07:52813085-52820003 | 1.236 | 1.00931 |
| lncRNA41006 | "x" | chr11:296148-300071 | 2.51016 | 3.32473 |
| lncRNA23835 | "x" | chr06:29665019-29666198 | 1.03653 | 1.80539 |
| lncRNA00044 | "u" | chr00:6390127-6405452 | 0.697919 | 1.63425 |
| lncRNA12765 | "x" | chr03:52965472-52970000 | 3.71392 | 3.08864 |
| lncRNA40089 | "x" | chr10:51181750-51187823 | 27.0743 | 40.8604 |
| lncRNA26887 | "u" | chr06:44409698-44410503 | 1.03042 | 1.47859 |
| lncRNA43910 | "u" | chr11:36434502-36435215 | 1.33554 | 1.91757 |
| lncRNA35948 | "u" | chr09:5008280-5009198 | 0.840083 | 1.20073 |
| lncRNA38587 | "x" | chr10:60238856-60242603 | 6.05009 | 7.53518 |
| lncRNA32157 | "x" | chr08:124883-130710 | 11.7603 | 19.9963 |
| lncRNA44140 | "x" | chr11:47642991-47650100 | 2.37263 | 4.2112 |
| lncRNA18083 | "x" | chr04:1974143-1980633 | 50.7263 | 30.4541 |
| lncRNA35794 | "x" | chr09:2903669-2905662 | 3.75659 | 4.6936 |
| lncRNA18814 | "u" | chr04:40645613-40646696 | 1.60995 | 1.18209 |
| lncRNA38922 | "x" | chr10:63478350-63484267 | 0.943005 | 1.65855 |
| lncRNA23838 | "x" | chr06:29732553-29733710 | 0.352848 | 0.616795 |
| lncRNA46665 | "u" | chr12:1438531-1439115 | 5.16018 | 6.74014 |
| lncRNA35848 | "x" | chr09:3624971-3637177 | 6.68741 | 8.78761 |
| lncRNA23082 | "x" | chr05:64164750-64169256 | 3.17455 | 4.10814 |
| lncRNA13857 | "x" | chr03:2083330-2089256 | 6.52795 | 10.2597 |
| lncRNA18917 | "x" | chr04:47393039-47400609 | 6.21299 | 5.00335 |
| lncRNA10308 | "x" | chr02:37627437-37629404 | 0.843912 | 1.36756 |
| lncRNA12428 | "x" | chr03:45442682-45457735 | 3.95213 | 2.63972 |
| lncRNA45485 | "x" | chr12:30145244-30148205 | 4.01906 | 3.1422 |
| lncRNA30580 | "x" | chr08:1336525-1338769 | 1.39209 | 2.37941 |
| lncRNA42133 | "x" | chr11:36979391-36988535 | 7.48936 | 9.19862 |
| lncRNA16226 | "u" | chr04:2249702-2250891 | 0.907763 | 1.2646 |
| lncRNA29726 | "x" | chr07:56497361-56499412 | 26.2822 | 17.0869 |
| lncRNA37292 | "x" | chr10:253915-256665 | 3.07098 | 4.24455 |
| lncRNA06659 | "x" | chr01:88484658-88485881 | 3.85978 | 2.05689 |
| lncRNA13091 | "u" | chr03:58444416-58445416 | 1.1123 | 1.54753 |
| lncRNA20049 | "x" | chr05:1930298-1935562 | 1.19138 | 0.73265 |
| lncRNA37066 | "x" | chr09:65122767-65128032 | 0.404061 | 0.786911 |
| lncRNA02011 | "x" | chr01:59188094-59189389 | 1.41666 | 1.84657 |
| lncRNA16257 | "x" | chr04:2570308-2578353 | 1.83916 | 1.35204 |
| lncRNA34132 | "x" | chr09:3499760-3502328 | 8.71829 | 6.20315 |
| lncRNA07940 | "x" | chr02:37621511-37624964 | 0.817281 | 0.432861 |
| lncRNA30614 | "u" | chr08:1862637-1863057 | 2.36908 | 1.23199 |
| lncRNA39597 | "u" | chr10:14880984-14881252 | 20.9956 | 10.9572 |
| lncRNA44131 | "u" | chr11:47453314-47453550 | 54.0466 | 27.6716 |
| lncRNA38553 | "x" | chr10:59885354-59889349 | 4.78841 | 7.66545 |
| lncRNA16067 | "u" | chr04:110938-113892 | 2.11418 | 2.55331 |
| lncRNA42111 | "u" | chr11:36382657-36383616 | 3.5961 | 2.82129 |
| lncRNA33016 | "x" | chr08:51705679-51710593 | 1.01904 | 2.08337 |
| lncRNA18858 | "x" | chr04:44289235-44291713 | 11.0949 | 12.8741 |
| lncRNA22944 | "x" | chr05:62380993-62397028 | 6.52623 | 4.33608 |
| lncRNA42741 | "x" | chr11:52559197-52571191 | 9.02782 | 11.748 |
| lncRNA41921 | "u" | chr11:22604817-22605512 | 1.1763 | 1.73648 |
| lncRNA18304 | "x" | chr04:4690977-4695742 | 9.08482 | 7.23529 |
| lncRNA44984 | "x" | chr12:3203220-3206950 | 14.7969 | 20.4415 |
| lncRNA23614 | "x" | chr06:20400257-20407369 | 1.78739 | 1.15531 |
| lncRNA28810 | "x" | chr07:561664-569230 | 2.29748 | 3.79394 |
| lncRNA46816 | "x" | chr12:3095648-3097004 | 141.074 | 184.606 |
| lncRNA05814 | "x" | chr01:79166715-79168365 | 0.635864 | 0.907309 |
| lncRNA47727 | "x" | chr12:46238588-46245484 | 1.56655 | 1.9651 |
| lncRNA09041 | "x" | chr02:49192401-49198143 | 1.20447 | 0.720892 |
| lncRNA17922 | "x" | chr04:85167-88296 | 3.58385 | 2.63198 |
| lncRNA17605 | "x" | chr04:60616805-60621171 | 1.27183 | 0.74306 |
| lncRNA00060 | "x" | chr00:6708737-6712713 | 1.22522 | 0.718675 |
| lncRNA23421 | "x" | chr06:3585051-3587390 | 6.32394 | 7.65097 |
| lncRNA01493 | "x" | chr01:9699751-9702925 | 0.873305 | 0.691965 |
| lncRNA38133 | "u" | chr10:46676074-46678093 | 5.69538 | 6.75362 |
| lncRNA47243 | "u" | chr12:16694575-16695181 | 0.931606 | 1.46934 |
| lncRNA39057 | "x" | chr10:64529611-64534734 | 22.8268 | 30.1679 |
| lncRNA05918 | "x" | chr01:80358781-80365636 | 1.23575 | 0.507395 |
| lncRNA36402 | "u" | chr09:45534223-45536731 | 1.25679 | 1.55677 |
| lncRNA43425 | "x" | chr11:7100970-7105587 | 8.57632 | 6.91404 |
| lncRNA00930 | "x" | chr00:16686996-16688259 | 2.48506 | 3.47139 |
| lncRNA12610 | "u" | chr03:48942379-48943717 | 4.11207 | 3.34041 |
| lncRNA19999 | "x" | chr05:1044055-1047873 | 2.82076 | 4.35738 |
| lncRNA09738 | "x" | chr02:31020919-31026815 | 3.08181 | 1.82416 |
| lncRNA19687 | "x" | chr04:61862712-61866268 | 2.30822 | 3.85965 |
| lncRNA25547 | "x" | chr06:17306389-17319502 | 0.938185 | 1.45605 |
| lncRNA25817 | "x" | chr06:30572701-30579292 | 3.53936 | 4.77884 |
| lncRNA26281 | "x" | chr06:37721325-37723464 | 1.94701 | 2.72506 |
| lncRNA30215 | "x" | chr07:62742398-62770824 | 3.34022 | 4.52471 |
| lncRNA43149 | "x" | chr11:3200298-3203957 | 213.58 | 313.675 |
| lncRNA01106 | "x" | chr01:353572-355936 | 6.65223 | 5.34023 |
| lncRNA28866 | "x" | chr07:1221695-1224155 | 69.425 | 35.4259 |
| lncRNA46830 | "x" | chr12:3235747-3247041 | 1.3777 | 2.03585 |
| lncRNA02934 | "x" | chr01:79269959-79274213 | 4.25832 | 2.6315 |
| lncRNA34925 | "x" | chr09:59112202-59113847 | 3.3823 | 5.18519 |
| lncRNA17037 | "u" | chr04:50584546-50585852 | 2.81663 | 2.23366 |
| lncRNA21898 | "x" | chr05:5179821-5182337 | 9.67471 | 6.58729 |
| lncRNA05553 | "x" | chr01:75841445-75843956 | 0.996993 | 0.645186 |
| lncRNA44259 | "x" | chr11:49736053-49741315 | 5.20438 | 3.528 |
| lncRNA33305 | "x" | chr08:56487333-56490213 | 1.73837 | 2.10738 |
| lncRNA06456 | "u" | chr01:86356163-86362118 | 2.54656 | 1.06185 |
| lncRNA27316 | "u" | chr07:3711124-3711723 | 43.6379 | 37.078 |
| lncRNA39002 | "x" | chr10:64053604-64057974 | 6.86286 | 5.17457 |
| lncRNA41664 | "x" | chr11:8955135-8956887 | 6.65717 | 10.9077 |
| lncRNA36010 | "u" | chr09:6208657-6209152 | 2.24688 | 1.45443 |
| lncRNA44004 | "u" | chr11:42374881-42375366 | 2.46238 | 1.60817 |
| lncRNA44567 | "u" | chr11:52935226-52935719 | 2.32183 | 1.52122 |
| lncRNA08588 | "x" | chr02:44585074-44590007 | 5.24833 | 2.27943 |
| lncRNA20386 | "x" | chr05:6468235-6474651 | 8.41221 | 4.95209 |
| lncRNA38525 | "x" | chr10:59645273-59647481 | 12.191 | 18.6566 |
| lncRNA44272 | "x" | chr11:49843260-49848948 | 5.74702 | 4.55956 |
| lncRNA04146 | "x" | chr01:1939494-1948962 | 2.17241 | 1.61827 |
| lncRNA15800 | "x" | chr03:61984209-61994404 | 1.0849 | 0.746207 |
| lncRNA02269 | "x" | chr01:70162433-70169225 | 0.667644 | 0.884654 |
| lncRNA09315 | "u" | chr02:15852502-15853053 | 3.00575 | 2.09916 |
| lncRNA46098 | "x" | chr12:61833152-61836527 | 33.3412 | 25.1962 |
| lncRNA32851 | "x" | chr08:45860733-45862945 | 1.05355 | 1.32937 |
| lncRNA31387 | "u" | chr08:53379128-53379722 | 2.43913 | 3.40858 |
| lncRNA26737 | "x" | chr06:42763786-42769026 | 2.15224 | 3.08665 |
| lncRNA18870 | "x" | chr04:44946774-44956676 | 1.78909 | 1.40278 |
| lncRNA28455 | "x" | chr07:62253400-62260108 | 6.49803 | 10.9671 |
| lncRNA48260 | "x" | chr12:64437937-64445800 | 1.91052 | 0.985572 |
| lncRNA43768 | "x" | chr11:27426690-27429313 | 1.74111 | 2.4334 |
| lncRNA10335 | "x" | chr02:37850456-37851848 | 10.6553 | 5.04706 |
| lncRNA15526 | "x" | chr03:59132508-59135382 | 4.94987 | 8.74088 |
| lncRNA25992 | "x" | chr06:33772754-33775920 | 5.67422 | 3.61885 |
| lncRNA42743 | "x" | chr11:52572322-52578579 | 22.3016 | 52.1474 |
| lncRNA22096 | "x" | chr05:8445998-8449582 | 0.760959 | 0.596214 |
| lncRNA37585 | "x" | chr10:3990597-3992416 | 1.14675 | 1.79433 |
| lncRNA01217 | "x" | chr01:1844882-1845987 | 6.14965 | 4.64771 |
| lncRNA02869 | "x" | chr01:78437976-78440511 | 3.88467 | 2.05405 |
| lncRNA10506 | "u" | chr02:39869840-39870451 | 1.77301 | 2.53276 |
| lncRNA24318 | "u" | chr06:37339629-37340351 | 1.30589 | 1.85226 |
| lncRNA28730 | "u" | chr07:64937007-64937339 | 3.81629 | 6.57405 |
| lncRNA47465 | "u" | chr12:36031416-36031665 | 23.0179 | 39.7802 |
| lncRNA14747 | "x" | chr03:45657120-45668274 | 0.937046 | 1.44574 |
| lncRNA21927 | "x" | chr05:5568468-5575981 | 2.79947 | 4.50232 |
| lncRNA22077 | "u" | chr05:8072275-8074241 | 2.15931 | 2.63883 |
| lncRNA07270 | "x" | chr02:28073079-28077641 | 1.62133 | 2.04479 |
| lncRNA45388 | "x" | chr12:19461738-19462640 | 7.97679 | 9.62112 |
| lncRNA15281 | "x" | chr03:56473307-56475421 | 0.631564 | 0.941211 |
| lncRNA25509 | "u" | chr06:14419038-14419583 | 1.14794 | 1.78479 |
| lncRNA38020 | "u" | chr10:39641344-39641545 | 162.974 | 253.099 |
| lncRNA43261 | "u" | chr11:4621420-4623257 | 5.41591 | 4.54976 |
| lncRNA46987 | "x" | chr12:4979761-4996830 | 2.66723 | 1.5762 |
| lncRNA02971 | "j" | chr01:79725751-79732204 | 2.77626 | 2.08056 |
| lncRNA33123 | "u" | chr08:53821464-53822909 | 0.872173 | 0.62585 |
| lncRNA44448 | "u" | chr11:51736058-51737000 | 1.49634 | 1.07458 |
| lncRNA01047 | "u" | chr00:21054914-21055458 | 1.17556 | 1.80779 |
| lncRNA26237 | "x" | chr06:37107167-37109010 | 1.37254 | 0.888306 |
| lncRNA28404 | "x" | chr07:61646262-61651507 | 2.07069 | 1.66482 |
| lncRNA27198 | "x" | chr07:1836679-1840269 | 3.37023 | 2.39996 |
| lncRNA18206 | "x" | chr04:3347067-3350915 | 1.91748 | 1.13332 |
| lncRNA31143 | "u" | chr08:46847571-46849005 | 0.885859 | 0.638701 |
| lncRNA41969 | "x" | chr11:28095241-28097066 | 8.17887 | 12.018 |
| lncRNA28227 | "u" | chr07:59275286-59276947 | 21.7489 | 25.0802 |
| lncRNA01905 | "x" | chr01:49645234-49655527 | 0.517186 | 0.783762 |
| lncRNA07025 | "u" | chr02:15179445-15181586 | 11.9616 | 13.8255 |
| lncRNA05960 | "x" | chr01:80817212-80818517 | 12.4963 | 17.6424 |
| lncRNA32011 | "x" | chr08:61808086-61811078 | 6.43702 | 14.7865 |
| lncRNA14505 | "u" | chr03:29149977-29155023 | 11.079 | 12.7513 |
| lncRNA36701 | "x" | chr09:59739140-59746647 | 3.27001 | 4.35833 |
| lncRNA06402 | "x" | chr01:85768996-85775706 | 0.739077 | 0.402057 |
| lncRNA29916 | "x" | chr07:59416585-59418182 | 174.13 | 99.9827 |
| lncRNA07633 | "x" | chr02:33985493-33990498 | 1.28562 | 0.715068 |
| lncRNA26789 | "u" | chr06:43363667-43364332 | 1.19263 | 1.76489 |
| lncRNA30927 | "x" | chr08:22666639-22676056 | 2.21849 | 1.41602 |
| lncRNA47178 | "x" | chr12:10649716-10651956 | 5.17546 | 7.30894 |
| lncRNA39947 | "u" | chr10:45828110-45828992 | 0.786218 | 1.15323 |
| lncRNA42570 | "x" | chr11:50808253-50810153 | 2.55098 | 3.3067 |
| lncRNA18344 | "x" | chr04:5190621-5192142 | 0.635354 | 0.899467 |
| lncRNA42459 | "x" | chr11:49647391-49660185 | 8.78479 | 14.116 |
| lncRNA39033 | "x" | chr10:64311046-64321881 | 12.2077 | 17.9549 |
| lncRNA18807 | "u" | chr04:40162134-40162567 | 3.21855 | 4.71443 |
| lncRNA02433 | "x" | chr01:72953845-72956902 | 0.696642 | 1.11277 |
| lncRNA46973 | "x" | chr12:4805083-4813039 | 11.0146 | 17.0187 |
| lncRNA32635 | "x" | chr08:22827080-22830300 | 3.05544 | 4.00576 |
| lncRNA14740 | "x" | chr03:45571023-45571439 | 1.001 | 1.95521 |
| lncRNA06101 | "u" | chr01:82368435-82371906 | 8.81394 | 7.6188 |
| lncRNA28138 | "x" | chr07:58253246-58259534 | 0.784073 | 0.45323 |
| lncRNA23926 | "u" | chr06:31782105-31783099 | 0.585452 | 0.855293 |
| lncRNA19740 | "x" | chr04:62343278-62344932 | 3.79993 | 5.44215 |
| lncRNA40437 | "x" | chr10:60076613-60079724 | 2.11801 | 1.35076 |
| lncRNA35168 | "x" | chr09:63034251-63038210 | 5.91073 | 7.85563 |
| lncRNA05349 | "x" | chr01:73022084-73023448 | 3.00152 | 4.82439 |
| lncRNA08485 | "u" | chr02:43533126-43534167 | 1.31768 | 0.952149 |
| lncRNA17114 | "u" | chr04:53052881-53053904 | 1.29903 | 0.938518 |
| lncRNA09111 | "x" | chr02:979607-989637 | 3.41225 | 2.9598 |
| lncRNA31303 | "o" | chr08:51504565-51505950 | 120.625 | 99.0546 |
| lncRNA37907 | "u" | chr10:29454406-29455335 | 1.51906 | 1.10009 |
| lncRNA15271 | "x" | chr03:56347667-56350481 | 5.5228 | 3.60191 |
| lncRNA18975 | "x" | chr04:50163411-50166584 | 0.561576 | 0.803893 |
| lncRNA33859 | "x" | chr09:212495-215736 | 15.4857 | 10.2168 |
| lncRNA18342 | "o" | chr04:5187974-5190114 | 1.228 | 1.53057 |
| lncRNA43022 | "x" | chr11:1786664-1791043 | 2.26365 | 1.61304 |
| lncRNA05367 | "x" | chr01:73275834-73278744 | 2.31413 | 1.24878 |
| lncRNA01589 | "u" | chr01:18521774-18524214 | 2.95825 | 3.53633 |
| lncRNA45662 | "x" | chr12:40668206-40671881 | 7.95287 | 4.73844 |
| lncRNA47312 | "x" | chr12:25089210-25091252 | 5.33721 | 3.02693 |
| lncRNA38618 | "x" | chr10:60540613-60547437 | 4.59446 | 2.97698 |
| lncRNA23209 | "u" | chr06:444055-450405 | 0.882452 | 0.734091 |
| lncRNA06425 | "x" | chr01:86019079-86030855 | 0.928189 | 0.430732 |
| lncRNA23242 | "x" | chr06:892129-896727 | 1.24676 | 0.649597 |
| lncRNA43099 | "x" | chr11:2728126-2736638 | 9.24433 | 5.66839 |
| lncRNA35541 | "x" | chr09:67455902-67464916 | 1.68248 | 2.40901 |
| lncRNA16264 | "x" | chr04:2611383-2617110 | 0.724708 | 1.04043 |
| lncRNA01288 | "x" | chr01:2405017-2406951 | 0.785511 | 0.509461 |
| lncRNA14973 | "x" | chr03:49854665-49872626 | 5.38311 | 3.93338 |
| lncRNA19420 | "x" | chr04:58478525-58479964 | 1.58768 | 2.13959 |
| lncRNA11343 | "x" | chr02:48498382-48499748 | 0.938208 | 1.53675 |
| lncRNA44880 | "x" | chr12:2198765-2202063 | 4.04082 | 7.13896 |
| lncRNA07785 | "x" | chr02:35891250-35896029 | 0.682595 | 1.30726 |
| lncRNA19621 | "x" | chr04:61235849-61237175 | 55.866 | 44.3506 |
| lncRNA24736 | "x" | chr06:41703224-41710440 | 1.15888 | 0.645358 |
| lncRNA01679 | "u" | chr01:26308980-26309285 | 33.0598 | 44.8334 |
| lncRNA26608 | "u" | chr06:41318703-41318969 | 67.8359 | 92.2845 |
| lncRNA11324 | "x" | chr02:48279730-48286030 | 2.36578 | 4.00005 |
| lncRNA12768 | "x" | chr03:52989789-52994332 | 1.57704 | 1.30358 |
| lncRNA28293 | "u" | chr07:60264256-60267194 | 1.87632 | 2.25422 |
| lncRNA42410 | "x" | chr11:49045600-49049862 | 2.89368 | 4.56174 |
| lncRNA13604 | "x" | chr03:63953874-63956934 | 7.16941 | 13.586 |
| lncRNA18904 | "x" | chr04:46729216-46740152 | 2.1287 | 2.72525 |
| lncRNA30479 | "u" | chr08:201676-201917 | 97.0346 | 68.4812 |
| lncRNA19345 | "x" | chr04:57425446-57431154 | 1.04503 | 0.613423 |
| lncRNA46759 | "x" | chr12:2460182-2464423 | 1.35306 | 0.857628 |
| lncRNA01826 | "x" | chr01:42168607-42178943 | 0.615501 | 0.44462 |
| lncRNA29076 | "x" | chr07:5091850-5097815 | 3.95704 | 2.91221 |
| lncRNA08470 | "x" | chr02:43273016-43276340 | 1.94791 | 2.76564 |
| lncRNA41094 | "x" | chr11:1149412-1152899 | 14.7414 | 24.4092 |
| lncRNA14207 | "u" | chr03:10197371-10198533 | 3.47035 | 2.80846 |
| lncRNA25108 | "x" | chr06:45674662-45677733 | 21.4702 | 25.1311 |
| lncRNA00412 | "x" | chr00:16968148-16969339 | 18.663 | 12.9143 |
| lncRNA14839 | "x" | chr03:46824851-46835629 | 5.42423 | 3.59764 |
| lncRNA18660 | "x" | chr04:26444326-26447007 | 7.87794 | 9.0733 |
| lncRNA38366 | "x" | chr10:57326790-57331826 | 2.49468 | 0.946839 |
| lncRNA46461 | "x" | chr12:65108204-65111658 | 4.37618 | 3.45365 |
| lncRNA34529 | "x" | chr09:23699416-23702446 | 0.748048 | 0.528827 |
| lncRNA26167 | "x" | chr06:36287043-36287626 | 4.44235 | 3.27789 |
| lncRNA16814 | "u" | chr04:31482059-31483961 | 1.34632 | 1.67772 |
| lncRNA36776 | "x" | chr09:61085462-61090631 | 2.61314 | 1.83374 |
| lncRNA22772 | "x" | chr05:59778184-59783636 | 1.10303 | 1.61924 |
| lncRNA40802 | "x" | chr10:63343021-63352252 | 1.63321 | 0.844647 |
| lncRNA08665 | "x" | chr02:45291149-45292547 | 26.1994 | 32.8213 |
| lncRNA17156 | "x" | chr04:54108407-54109912 | 2.17533 | 1.48081 |
| lncRNA11060 | "x" | chr02:45480964-45499020 | 1.44622 | 1.19067 |
| lncRNA28374 | "x" | chr07:61298004-61304066 | 1.44744 | 0.815345 |
| lncRNA43084 | "x" | chr11:2522010-2523249 | 14.7308 | 21.1439 |
| lncRNA18336 | "u" | chr04:5149623-5150559 | 1.82548 | 1.35564 |
| lncRNA07509 | "x" | chr02:32407023-32408160 | 0.479294 | 0.621208 |
| lncRNA42624 | "u" | chr11:51528818-51529724 | 1.96128 | 1.45996 |
| lncRNA45547 | "u" | chr12:33657396-33658311 | 1.92373 | 1.43052 |
| lncRNA33050 | "j" | chr08:52505032-52509064 | 3.86171 | 3.05379 |
| lncRNA43488 | "x" | chr11:8752888-8759597 | 89.8657 | 133.334 |
| lncRNA17843 | "x" | chr04:63303698-63308948 | 4.41754 | 3.27322 |
| lncRNA18139 | "x" | chr04:2687047-2691103 | 5.84083 | 7.32019 |
| lncRNA29902 | "x" | chr07:59211524-59214134 | 45.6667 | 28.659 |
| lncRNA05127 | "x" | chr01:69190397-69192447 | 1.49913 | 0.775282 |
| lncRNA12783 | "x" | chr03:53235915-53245445 | 2.51731 | 1.24461 |
| lncRNA13855 | "x" | chr03:2070363-2072761 | 1.57518 | 1.97659 |
| lncRNA05181 | "x" | chr01:70398108-70399264 | 6.18843 | 7.98607 |
| lncRNA05406 | "u" | chr01:73913153-73915538 | 1.6552 | 1.35101 |
| lncRNA15839 | "x" | chr03:62440994-62441745 | 2.97831 | 1.66151 |
| lncRNA19786 | "x" | chr04:62925051-62928981 | 1.60692 | 2.29472 |
| lncRNA25406 | "x" | chr06:3451315-3452881 | 2.02198 | 1.37063 |
| lncRNA03437 | "u" | chr01:84595532-84629171 | 1.40412 | 2.65432 |
| lncRNA11934 | "x" | chr03:10127627-10144738 | 0.685662 | 0.835009 |
| lncRNA40860 | "x" | chr10:63872643-63879195 | 1.7345 | 1.06148 |
| lncRNA46666 | "x" | chr12:1441032-1446614 | 4.37841 | 5.79515 |
| lncRNA34084 | "u" | chr09:2880894-2881761 | 3.64623 | 4.57147 |
| lncRNA42717 | "x" | chr11:52334893-52341200 | 0.688764 | 0.357629 |
| lncRNA19101 | "x" | chr04:53628299-53629624 | 4.00209 | 3.28173 |
| lncRNA39658 | "x" | chr10:21450344-21460183 | 0.553338 | 1.04089 |
| lncRNA39383 | "x" | chr10:3664497-3667738 | 2.31695 | 1.69631 |
| lncRNA28690 | "x" | chr07:64613546-64616777 | 3.44056 | 2.44842 |
| lncRNA40840 | "x" | chr10:63693702-63703010 | 7.07613 | 10.1709 |
| lncRNA12875 | "x" | chr03:55733844-55737259 | 13.5419 | 8.42522 |
| lncRNA15295 | "x" | chr03:56639757-56644031 | 9.79781 | 13.9975 |
| lncRNA19031 | "u" | chr04:51733710-51734266 | 2.13351 | 3.03265 |
| lncRNA03830 | "x" | chr01:88691960-88697480 | 15.7714 | 9.23546 |
| lncRNA15981 | "x" | chr03:63964952-63970550 | 6.93383 | 11.3719 |
| lncRNA01325 | "x" | chr01:2909911-2917728 | 7.80713 | 5.16859 |
| lncRNA12044 | "x" | chr03:14579117-14582404 | 0.924064 | 0.637005 |
| lncRNA24704 | "x" | chr06:41440397-41441789 | 7.76986 | 9.96653 |
| lncRNA44961 | "x" | chr12:3021765-3026459 | 4.76579 | 6.67089 |
| lncRNA16182 | "x" | chr04:1594826-1599582 | 0.828092 | 1.07984 |
| lncRNA25342 | "x" | chr06:2406339-2410266 | 1.50811 | 0.997283 |
| lncRNA38971 | "x" | chr10:63850346-63854136 | 0.75474 | 1.18796 |
| lncRNA19289 | "x" | chr04:56792509-56796408 | 3.15665 | 2.47582 |
| lncRNA37820 | "u" | chr10:21743710-21744177 | 16.7192 | 20.5401 |
| lncRNA08564 | "x" | chr02:44273724-44285278 | 2.5798 | 4.37521 |
| lncRNA23169 | "x" | chr05:64915277-64917382 | 0.908578 | 0.586149 |
| lncRNA11306 | "x" | chr02:48109838-48115386 | 3.11338 | 2.17402 |
| lncRNA46579 | "u" | chr12:511399-511930 | 24.803 | 29.6063 |
| lncRNA22406 | "x" | chr05:29363243-29365771 | 2.4391 | 4.14891 |
| lncRNA41183 | "x" | chr11:1911567-1914794 | 3.887 | 3.12702 |
| lncRNA00123 | "u" | chr00:10680478-10681284 | 0.820411 | 1.22671 |
| lncRNA39834 | "u" | chr10:38322664-38323307 | 1.22328 | 1.81573 |
| lncRNA07870 | "x" | chr02:36760381-36761958 | 16.544 | 11.5258 |
| lncRNA15718 | "u" | chr03:61174517-61175152 | 3.21129 | 2.40332 |
| lncRNA25555 | "u" | chr06:18030620-18030944 | 29.7953 | 22.3083 |
| lncRNA27609 | "x" | chr07:26157083-26168061 | 1.09793 | 0.695191 |
| lncRNA48152 | "x" | chr12:63516574-63520088 | 29.5081 | 25.8976 |
| lncRNA30336 | "x" | chr07:64060641-64061262 | 16.4438 | 8.27667 |
| lncRNA42770 | "x" | chr11:52911687-52921096 | 0.7869 | 0.484126 |
| lncRNA30897 | "x" | chr08:17243265-17256990 | 1.29267 | 1.09457 |
| lncRNA41071 | "x" | chr11:899284-912577 | 2.01805 | 1.50551 |
| lncRNA45450 | "x" | chr12:27263961-27267617 | 0.835111 | 1.02266 |
| lncRNA31651 | "x" | chr08:57594646-57603672 | 9.68805 | 14.9967 |
| lncRNA04765 | "u" | chr01:47296798-47297815 | 0.721627 | 1.03512 |
| lncRNA36550 | "u" | chr09:55885028-55889505 | 8.60455 | 6.61264 |
| lncRNA06365 | "u" | chr01:85366664-85373036 | 0.636897 | 1.07405 |
| lncRNA02948 | "x" | chr01:79421543-79440342 | 0.665089 | 1.20288 |
| lncRNA06239 | "x" | chr01:83958123-83964384 | 0.660858 | 1.11268 |
| lncRNA06611 | "u" | chr01:87996446-87996947 | 1.73575 | 1.12137 |
| lncRNA18808 | "u" | chr04:40162777-40163744 | 3.02397 | 2.38636 |
| lncRNA04364 | "x" | chr01:6839658-6846955 | 3.34685 | 6.43436 |
| lncRNA00045 | "u" | chr00:6390127-6405452 | 1.03373 | 0.447946 |
| lncRNA23946 | "u" | chr06:32215331-32215689 | 4.81588 | 3.13158 |
| lncRNA33683 | "u" | chr08:61243511-61244036 | 1.4481 | 0.938134 |
| lncRNA38960 | "x" | chr10:63761666-63765608 | 1.68134 | 1.18401 |
| lncRNA28901 | "x" | chr07:1832945-1833484 | 1.7187 | 3.14033 |
| lncRNA40346 | "x" | chr10:59311807-59315187 | 3.18528 | 6.15321 |
| lncRNA32217 | "x" | chr08:891600-893647 | 1.18242 | 1.75481 |
| lncRNA27764 | "u" | chr07:46979539-46981371 | 2.60212 | 1.74594 |
| lncRNA03862 | "x" | chr01:89040901-89067561 | 0.419074 | 0.699293 |
| lncRNA24309 | "x" | chr06:37200118-37205677 | 1.69245 | 2.38986 |
| lncRNA03314 | "x" | chr01:83313052-83324485 | 1.07317 | 0.638185 |
| lncRNA31922 | "x" | chr08:60678187-60683202 | 1.41001 | 0.550927 |
| lncRNA12998 | "x" | chr03:57356346-57363214 | 1.18146 | 0.670746 |
| lncRNA24127 | "x" | chr06:34955530-34956677 | 6.19276 | 9.88681 |
| lncRNA30956 | "u" | chr08:25827458-25828364 | 5.57162 | 6.75357 |
| lncRNA12532 | "x" | chr03:46975388-46978149 | 16.9489 | 21.6133 |
| lncRNA41060 | "x" | chr11:861638-862328 | 41.0547 | 29.7427 |
| lncRNA42862 | "x" | chr11:432321-436359 | 29.1214 | 23.9206 |
| lncRNA46510 | "x" | chr12:65427339-65429750 | 0.639204 | 1.12789 |
| lncRNA08066 | "u" | chr02:39120271-39120530 | 11.5133 | 22.3272 |
| lncRNA34381 | "x" | chr09:11274732-11277447 | 71.3625 | 100.16 |
| lncRNA13409 | "x" | chr03:61772489-61778815 | 0.713027 | 1.16237 |
| lncRNA37854 | "x" | chr10:23752985-23760474 | 2.14333 | 3.34116 |
| lncRNA06813 | "u" | chr01:90091178-90092733 | 1.0094 | 0.761839 |
| lncRNA09707 | "x" | chr02:30419777-30425448 | 6.69827 | 4.66177 |
| lncRNA27486 | "x" | chr07:9831864-9832976 | 17.9656 | 25.5354 |
| lncRNA39039 | "u" | chr10:64353634-64354537 | 58.2383 | 50.8785 |
| lncRNA11827 | "x" | chr03:8346705-8348968 | 0.637722 | 0.333131 |
| lncRNA40940 | "x" | chr10:64540172-64543143 | 14.5694 | 17.8013 |
| lncRNA32001 | "u" | chr08:61711641-61715964 | 4.98112 | 4.31486 |
| lncRNA25585 | "x" | chr06:20751057-20768833 | 13.5537 | 10.9966 |
| lncRNA10614 | "x" | chr02:41034298-41044992 | 0.609702 | 0.297461 |
| lncRNA22583 | "x" | chr05:50955934-50960275 | 0.574689 | 0.707199 |
| lncRNA42835 | "x" | chr11:188097-192782 | 1.47255 | 1.0257 |
| lncRNA36604 | "x" | chr09:57355328-57358269 | 20.1963 | 31.6406 |
| lncRNA39384 | "x" | chr10:3664497-3667738 | 3.54593 | 4.29588 |
| lncRNA41331 | "x" | chr11:3597344-3606823 | 1.99794 | 3.15652 |
| lncRNA18435 | "x" | chr04:6986536-6988507 | 37.8376 | 32.4508 |
| lncRNA16091 | "x" | chr04:385718-389274 | 0.748911 | 1.25946 |
| lncRNA15883 | "x" | chr03:62947040-62953026 | 1.74467 | 1.4418 |
| lncRNA45856 | "x" | chr12:46439156-46444896 | 1.72769 | 2.15847 |
| lncRNA39326 | "o" | chr10:2695468-2697177 | 2.20834 | 1.71239 |
| lncRNA35983 | "x" | chr09:5705196-5707608 | 5.11797 | 3.29594 |
| lncRNA06958 | "x" | chr02:12011269-12016553 | 2.92918 | 3.90506 |
| lncRNA14291 | "x" | chr03:13305344-13308989 | 2.10842 | 1.28549 |
| lncRNA26937 | "x" | chr06:44821207-44827496 | 1.47412 | 0.956811 |
| lncRNA21502 | "u" | chr05:64760462-64762694 | 1.13627 | 1.40551 |
| lncRNA35865 | "x" | chr09:3813995-3817092 | 6.98882 | 4.74069 |
| lncRNA05983 | "x" | chr01:81105284-81111003 | 5.07686 | 3.70404 |
| lncRNA25268 | "x" | chr06:1644025-1644767 | 18.8685 | 11.9098 |
| lncRNA41973 | "x" | chr11:28366612-28370692 | 5.82055 | 3.51946 |
| lncRNA43498 | "u" | chr11:9305839-9309663 | 0.897147 | 0.735259 |
| lncRNA02517 | "x" | chr01:73882627-73887074 | 1.61813 | 2.29652 |
| lncRNA26880 | "x" | chr06:44333170-44336457 | 1.4526 | 0.90168 |
| lncRNA07775 | "x" | chr02:35797453-35809682 | 1.79469 | 1.29372 |
| lncRNA02916 | "x" | chr01:79034637-79037851 | 2.63739 | 1.98754 |
| lncRNA24456 | "x" | chr06:38933871-38935635 | 2.99893 | 2.36033 |
| lncRNA01262 | "x" | chr01:2134713-2143061 | 1.75931 | 2.59838 |
| lncRNA17954 | "x" | chr04:326469-332708 | 3.07593 | 4.60413 |
| lncRNA48368 | "x" | chr12:65238625-65241667 | 14.9306 | 10.2285 |
| lncRNA37512 | "x" | chr10:2826476-2829741 | 2.56635 | 1.2675 |
| lncRNA42755 | "x" | chr11:52667621-52692241 | 0.861209 | 0.483616 |
| lncRNA15537 | "x" | chr03:59199582-59201528 | 4.0344 | 6.14822 |
| lncRNA24769 | "x" | chr06:42068610-42070616 | 9.99607 | 11.9134 |
| lncRNA02409 | "x" | chr01:72490431-72498057 | 1.93117 | 0.830402 |
| lncRNA08639 | "x" | chr02:45066560-45073107 | 1.35313 | 0.999291 |
| lncRNA11757 | "x" | chr03:7278414-7283352 | 2.96428 | 4.70015 |
| lncRNA25801 | "x" | chr06:29980371-29982511 | 2.05845 | 2.96736 |
| lncRNA19013 | "u" | chr04:51063231-51066795 | 4.68578 | 5.37809 |
| lncRNA11891 | "x" | chr03:9319631-9320960 | 2.46331 | 1.856 |
| lncRNA01310 | "x" | chr01:2746554-2751492 | 1.37088 | 2.52347 |
| lncRNA27112 | "x" | chr07:641088-647292 | 0.85339 | 0.552525 |
| lncRNA05432 | "u" | chr01:74228468-74228757 | 59.7412 | 45.4413 |
| lncRNA08303 | "x" | chr02:41507852-41509331 | 4.1843 | 2.65383 |
| lncRNA43169 | "u" | chr11:3432566-3432945 | 17.2032 | 13.0852 |
| lncRNA17708 | "x" | chr04:61696178-61698205 | 1.26668 | 0.904895 |
| lncRNA19822 | "x" | chr04:63291171-63293327 | 1.8622 | 2.93366 |
| lncRNA43281 | "o" | chr11:4788417-4801208 | 0.380555 | 0.892175 |
| lncRNA00187 | "x" | chr00:12012451-12016317 | 0.861276 | 0.637389 |
| lncRNA31689 | "u" | chr08:58075324-58090632 | 6.66679 | 23.567 |
| lncRNA24999 | "x" | chr06:44579621-44585131 | 3.03783 | 2.35821 |
| lncRNA36224 | "x" | chr09:24424857-24427603 | 3.71476 | 2.58797 |
| lncRNA07219 | "u" | chr02:23332880-23333588 | 2.13 | 1.55907 |
| lncRNA06543 | "x" | chr01:87218576-87223859 | 0.910107 | 1.25379 |
| lncRNA12678 | "x" | chr03:51230959-51234648 | 190.736 | 242.594 |
| lncRNA43299 | "x" | chr11:4991629-4996469 | 1.52739 | 2.33759 |
| lncRNA03155 | "u" | chr01:81582843-81587700 | 0.93364 | 0.777912 |
| lncRNA03865 | "x" | chr01:89040901-89067561 | 2.92896 | 4.03584 |
| lncRNA16174 | "x" | chr04:1515365-1517843 | 1.43215 | 2.3111 |
| lncRNA46174 | "x" | chr12:62576317-62584915 | 2.03592 | 2.69281 |
| lncRNA05712 | "x" | chr01:77882949-77894918 | 3.59966 | 5.07956 |
| lncRNA31392 | "x" | chr08:53473689-53480890 | 4.46489 | 2.74004 |
| lncRNA31718 | "x" | chr08:58437301-58439198 | 1.11206 | 0.77712 |
| lncRNA36105 | "u" | chr09:13481580-13483488 | 2.13934 | 2.57208 |
| lncRNA42493 | "x" | chr11:50060257-50066358 | 1.8315 | 2.55199 |
| lncRNA10080 | "x" | chr02:35057066-35059876 | 8.03706 | 6.39977 |
| lncRNA15745 | "u" | chr03:61417302-61418567 | 1.81131 | 2.27323 |
| lncRNA17190 | "x" | chr04:54468418-54470438 | 4.48282 | 6.75689 |
| lncRNA22580 | "x" | chr05:50848962-50851677 | 6.98087 | 8.58309 |
| lncRNA43535 | "x" | chr11:10488197-10491376 | 5.08592 | 5.84559 |
| lncRNA01339 | "x" | chr01:3021935-3024369 | 1.0971 | 1.83555 |
| lncRNA27074 | "x" | chr07:213547-218992 | 2.13163 | 1.58455 |
| lncRNA15816 | "x" | chr03:62183746-62189147 | 12.8334 | 7.79078 |
| lncRNA06215 | "x" | chr01:83615904-83617335 | 2.79306 | 4.01887 |
| lncRNA15502 | "x" | chr03:58933141-58937532 | 1.10064 | 2.2841 |
| lncRNA36872 | "x" | chr09:62533093-62533941 | 5.80993 | 3.02992 |
| lncRNA03942 | "x" | chr01:89898591-89901258 | 2.27 | 1.47915 |
| lncRNA09104 | "u" | chr02:589861-591873 | 0.511652 | 0.665137 |
| lncRNA23973 | "u" | chr06:32621758-32622320 | 27.2357 | 23.0801 |
| lncRNA41048 | "x" | chr11:713235-715034 | 1.63811 | 2.32167 |
| lncRNA17512 | "u" | chr04:59492745-59493527 | 2.09533 | 2.71341 |
| lncRNA20339 | "x" | chr05:5881435-5887946 | 0.975946 | 1.3251 |
| lncRNA47311 | "x" | chr12:24889980-24893149 | 2.94061 | 3.41959 |
| lncRNA39278 | "x" | chr10:2014233-2015443 | 5.64251 | 7.59174 |
| lncRNA40156 | "x" | chr10:52712204-52715021 | 4.07744 | 5.16179 |
| lncRNA46580 | "x" | chr12:514174-518078 | 8.36865 | 5.26097 |
| lncRNA27587 | "u" | chr07:23230486-23232236 | 0.826161 | 1.0672 |
| lncRNA39577 | "u" | chr10:13617943-13618190 | 164.877 | 125.881 |
| lncRNA41651 | "x" | chr11:8631105-8633554 | 246.141 | 415.406 |
| lncRNA15347 | "x" | chr03:57174318-57175600 | 3.21314 | 4.05101 |
| lncRNA44188 | "x" | chr11:48643229-48645282 | 18.8063 | 13.7629 |
| lncRNA41053 | "x" | chr11:753421-760342 | 1.98607 | 1.62648 |
| lncRNA02527 | "x" | chr01:74064301-74066944 | 3.63497 | 2.92907 |
| lncRNA40480 | "x" | chr10:60528397-60530625 | 5.37168 | 3.98701 |
| lncRNA26726 | "u" | chr06:42683445-42684115 | 2.79624 | 3.66403 |
| lncRNA01245 | "j" | chr01:1897088-1903877 | 6.08525 | 14.2265 |
| lncRNA46633 | "x" | chr12:1027443-1036126 | 1.9418 | 2.86298 |
| lncRNA17509 | "x" | chr04:59454342-59461803 | 3.81319 | 5.58095 |
| lncRNA12446 | "x" | chr03:45726644-45729457 | 7.88953 | 11.1913 |
| lncRNA28265 | "u" | chr07:59990718-59994044 | 1.02974 | 0.842342 |
| lncRNA35283 | "u" | chr09:64769615-64770398 | 4.24725 | 5.26503 |
| lncRNA30162 | "x" | chr07:62191787-62196856 | 1.21244 | 0.522575 |
| lncRNA41011 | "x" | chr11:323208-326089 | 11.9996 | 8.61761 |
| lncRNA21096 | "u" | chr05:59068974-59069548 | 5.33504 | 4.12508 |
| lncRNA21489 | "u" | chr05:64548756-64549645 | 2.57645 | 1.99335 |
| lncRNA26263 | "x" | chr06:37444278-37451300 | 2.51292 | 5.41072 |
| lncRNA20319 | "u" | chr05:5738728-5741442 | 1.06779 | 1.29644 |
| lncRNA05560 | "x" | chr01:75913573-75920741 | 0.618013 | 0.299769 |
| lncRNA43329 | "x" | chr11:5296399-5298810 | 3.88519 | 5.0346 |
| lncRNA35067 | "x" | chr09:61663673-61667766 | 1.30828 | 0.993817 |
| lncRNA47249 | "u" | chr12:16939234-16941875 | 2.71085 | 3.18727 |
| lncRNA20785 | "x" | chr05:36735651-36738139 | 0.732062 | 0.915656 |
| lncRNA21970 | "x" | chr05:6145881-6147456 | 19.351 | 29.4557 |
| lncRNA40247 | "x" | chr10:57843107-57850214 | 2.05903 | 1.59599 |
| lncRNA41281 | "x" | chr11:3059195-3063859 | 49.6057 | 31.432 |
| lncRNA30440 | "x" | chr07:65086441-65095484 | 10.8024 | 15.6303 |
| lncRNA31681 | "x" | chr08:57904884-57907008 | 0.856539 | 0.442503 |
| lncRNA48285 | "x" | chr12:64645084-64648815 | 1.93396 | 3.17742 |
| lncRNA40552 | "x" | chr10:61166124-61167633 | 160.528 | 126.778 |
| lncRNA40665 | "x" | chr10:62194246-62195357 | 1.85562 | 1.03133 |
| lncRNA12758 | "x" | chr03:52900673-52904929 | 3.90217 | 5.35519 |
| lncRNA43753 | "x" | chr11:25327267-25328266 | 2.00309 | 2.52653 |
| lncRNA19843 | "u" | chr04:63473291-63473852 | 3.86811 | 2.94042 |
| lncRNA09729 | "u" | chr02:30868614-30869397 | 1.31173 | 0.926047 |
| lncRNA14682 | "u" | chr03:43397363-43397769 | 5.9111 | 4.18461 |
| lncRNA09416 | "u" | chr02:19681247-19681641 | 6.36136 | 4.53119 |
| lncRNA16166 | "u" | chr04:1400936-1401485 | 2.39165 | 1.69448 |
| lncRNA40714 | "x" | chr10:62591082-62601807 | 44.4632 | 62.1493 |
| lncRNA12946 | "x" | chr03:56743334-56744186 | 89.0502 | 76.9565 |
| lncRNA22804 | "x" | chr05:60306633-60308701 | 6.56837 | 8.69558 |
| lncRNA28745 | "x" | chr07:65062856-65073697 | 5.83165 | 3.78633 |
| lncRNA37570 | "u" | chr10:3624474-3624886 | 17.6061 | 14.0207 |
| lncRNA45181 | "x" | chr12:5559536-5566991 | 6.95036 | 9.82751 |
| lncRNA35182 | "x" | chr09:63374805-63382281 | 0.749547 | 0.467308 |
| lncRNA34171 | "x" | chr09:3947450-3949898 | 1.67885 | 1.33401 |
| lncRNA13173 | "x" | chr03:59290075-59293960 | 0.889096 | 0.516207 |
| lncRNA16283 | "x" | chr04:2708579-2711828 | 0.881541 | 1.26031 |
| lncRNA40767 | "x" | chr10:63073716-63077422 | 1.5449 | 1.23313 |
| lncRNA14536 | "u" | chr03:29873460-29876089 | 1.09964 | 1.33504 |
| lncRNA40739 | "x" | chr10:62858794-62864339 | 6.57683 | 4.73157 |
| lncRNA44354 | "x" | chr11:50648078-50651754 | 5.48128 | 4.57307 |
| lncRNA45960 | "x" | chr12:48272767-48277117 | 31.232 | 42.5303 |
| lncRNA37125 | "x" | chr09:65730189-65735838 | 42.2734 | 69.8128 |
| lncRNA19924 | "x" | chr05:173411-177352 | 0.397412 | 0.639937 |
| lncRNA35158 | "x" | chr09:62866644-62867840 | 13.2328 | 24.9044 |
| lncRNA20763 | "u" | chr05:32221466-32229031 | 1.33576 | 1.15171 |
| lncRNA19362 | "x" | chr04:57711281-57714900 | 0.800477 | 0.51877 |
| lncRNA24866 | "x" | chr06:43181967-43189299 | 2.46595 | 3.61575 |
| lncRNA37783 | "u" | chr10:19360009-19363426 | 0.868182 | 1.05356 |
| lncRNA45697 | "x" | chr12:42645655-42654179 | 6.88399 | 8.9443 |
| lncRNA32220 | "x" | chr08:927730-928558 | 5.49741 | 8.2294 |
| lncRNA24459 | "x" | chr06:38986528-38993423 | 0.735621 | 1.01561 |
| lncRNA48121 | "x" | chr12:63270716-63276349 | 1.00323 | 2.17207 |
| lncRNA43754 | "u" | chr11:25330251-25330736 | 5.56254 | 7.25494 |
| lncRNA27137 | "u" | chr07:1026774-1027914 | 1.23254 | 1.60539 |
| lncRNA42340 | "x" | chr11:47845950-47851492 | 4.69902 | 3.41979 |
| lncRNA43572 | "x" | chr11:12908977-12917341 | 6.4711 | 4.80772 |
| lncRNA40351 | "x" | chr10:59347335-59350521 | 2.94665 | 2.09029 |
| lncRNA32183 | "x" | chr08:393370-396901 | 1.0171 | 1.22529 |
| lncRNA39324 | "x" | chr10:2686334-2687915 | 3.02734 | 4.20061 |
| lncRNA02558 | "x" | chr01:74375860-74378427 | 3.58913 | 2.69918 |
| lncRNA06376 | "x" | chr01:85428609-85440669 | 1.08861 | 0.601372 |
| lncRNA30863 | "x" | chr08:10360927-10372570 | 0.58575 | 0.885756 |
| lncRNA28528 | "x" | chr07:63054492-63056714 | 3.83187 | 2.3249 |
| lncRNA14395 | "x" | chr03:19835585-19850837 | 2.50695 | 2.99463 |
| lncRNA40382 | "x" | chr10:59637702-59641173 | 1.57853 | 1.12251 |
| lncRNA10816 | "x" | chr02:43075014-43078764 | 17.8547 | 8.23459 |
| lncRNA16151 | "x" | chr04:1170313-1170939 | 15.8433 | 28.6747 |
| lncRNA25415 | "x" | chr06:3584116-3584857 | 74.1117 | 96.703 |
| lncRNA10124 | "u" | chr02:35550108-35550700 | 3.28824 | 4.29207 |
| lncRNA43992 | "u" | chr11:42081073-42082774 | 0.629301 | 0.822543 |
| lncRNA00129 | "x" | chr00:10687937-10692676 | 0.475894 | 0.670621 |
| lncRNA40070 | "x" | chr10:50725975-50733040 | 41.9579 | 55.6456 |
| lncRNA10839 | "x" | chr02:43422136-43425124 | 1.70894 | 1.23435 |
| lncRNA16282 | "x" | chr04:2705102-2708194 | 1.44251 | 2.23953 |
| lncRNA45944 | "x" | chr12:47949016-47950974 | 2.1697 | 1.6335 |
| lncRNA04050 | "x" | chr01:915538-919333 | 5.38933 | 6.77238 |
| lncRNA30063 | "x" | chr07:60945364-60946499 | 3.52432 | 5.07893 |
| lncRNA37475 | "x" | chr10:2460340-2462146 | 9.43875 | 10.9955 |
| lncRNA40880 | "x" | chr10:64072672-64082262 | 32.5022 | 26.7265 |
| lncRNA19723 | "x" | chr04:62155072-62158474 | 9.97099 | 12.9304 |
| lncRNA42375 | "x" | chr11:48393784-48402079 | 1.67162 | 1.31812 |
| lncRNA04103 | "x" | chr01:1595933-1601058 | 1.62056 | 1.10599 |
| lncRNA23719 | "u" | chr06:23161433-23163165 | 0.652966 | 0.84968 |
| lncRNA45075 | "x" | chr12:4139190-4140793 | 40.758 | 66.2234 |
| lncRNA48300 | "x" | chr12:64752121-64756747 | 4.03514 | 5.37619 |
| lncRNA46595 | "x" | chr12:648796-656982 | 1.20998 | 0.832535 |
| lncRNA03093 | "x" | chr01:80889917-80896362 | 2.13189 | 1.63785 |
| lncRNA11226 | "x" | chr02:47253987-47256530 | 6.10969 | 4.60423 |
| lncRNA31213 | "x" | chr08:48833026-48840941 | 8.17796 | 5.35351 |
| lncRNA46916 | "x" | chr12:4033784-4039439 | 7.34609 | 5.48594 |
| lncRNA42163 | "u" | chr11:38136609-38138851 | 0.702541 | 0.880233 |
| lncRNA17565 | "u" | chr04:60085514-60086084 | 3.09273 | 2.29979 |
| lncRNA25142 | "u" | chr06:55248-55633 | 9.8686 | 7.36477 |
| lncRNA19236 | "x" | chr04:55811076-55818764 | 2.19166 | 1.76986 |
| lncRNA01057 | "u" | chr00:21404777-21405974 | 1.68675 | 1.33444 |
| lncRNA18563 | "x" | chr04:19180777-19194213 | 1.05387 | 0.678149 |
| lncRNA36284 | "x" | chr09:31327327-31331118 | 1.07485 | 0.892979 |
| lncRNA36479 | "x" | chr09:50406659-50409020 | 5.95306 | 6.81962 |
| lncRNA40858 | "x" | chr10:63858277-63860326 | 1.18456 | 0.876618 |
| lncRNA01419 | "x" | chr01:5134693-5138389 | 0.755984 | 0.511398 |
| lncRNA07351 | "x" | chr02:30046995-30047549 | 1.71154 | 1.11855 |
| lncRNA43627 | "x" | chr11:15633539-15636765 | 1.3187 | 0.775464 |
| lncRNA16309 | "x" | chr04:2995393-2997327 | 6.02273 | 5.20114 |
| lncRNA06963 | "x" | chr02:12119190-12132004 | 0.440632 | 0.758557 |
| lncRNA19974 | "x" | chr05:686671-691952 | 3.62374 | 2.83622 |
| lncRNA06756 | "x" | chr01:89429433-89436218 | 1.45136 | 2.09803 |
| lncRNA41621 | "u" | chr11:8172865-8192556 | 2.14005 | 2.63853 |
| lncRNA08660 | "x" | chr02:45248844-45251410 | 1.14644 | 1.98198 |
| lncRNA38958 | "x" | chr10:63758836-63761440 | 8.35398 | 10.1962 |
| lncRNA19308 | "x" | chr04:57120456-57125200 | 1.80411 | 1.38361 |
| lncRNA41025 | "x" | chr11:449969-456855 | 3.62311 | 2.85096 |
| lncRNA44864 | "x" | chr12:2069979-2071739 | 2.37377 | 1.9526 |
| lncRNA13143 | "u" | chr03:59021833-59027357 | 1.28901 | 1.01907 |
| lncRNA43008 | "x" | chr11:1694546-1705386 | 1.94083 | 2.79678 |
| lncRNA25093 | "x" | chr06:45551947-45553841 | 13.0017 | 9.78867 |
| lncRNA21461 | "x" | chr05:64284982-64286688 | 2.84867 | 3.60642 |
| lncRNA12117 | "u" | chr03:20216434-20216919 | 5.53874 | 7.18657 |
| lncRNA20258 | "x" | chr05:4706111-4711597 | 7.07978 | 4.75574 |
| lncRNA34907 | "x" | chr09:58581272-58591924 | 4.47942 | 3.55354 |
| lncRNA14974 | "x" | chr03:49854665-49872626 | 0.969662 | 0.599338 |
| lncRNA06019 | "x" | chr01:81463392-81480559 | 2.37113 | 5.15797 |
| lncRNA35898 | "x" | chr09:4259084-4262312 | 3.44403 | 2.40462 |
| lncRNA38130 | "x" | chr10:46653943-46656325 | 2.63567 | 3.09786 |
| lncRNA40544 | "x" | chr10:61060292-61064202 | 6.21329 | 9.60985 |
| lncRNA22983 | "u" | chr05:63022396-63022832 | 65.0494 | 55.744 |
| lncRNA23695 | "x" | chr06:22354580-22355878 | 0.892687 | 1.15492 |
| lncRNA17603 | "x" | chr04:60578294-60581135 | 17.8919 | 14.4984 |
| lncRNA18341 | "x" | chr04:5177401-5182959 | 3.42409 | 4.10338 |
| lncRNA48171 | "x" | chr12:63642737-63647810 | 7.98121 | 9.34677 |
| lncRNA36131 | "x" | chr09:15118956-15122657 | 2.34352 | 3.40471 |
| lncRNA39816 | "x" | chr10:37043744-37044274 | 1.46563 | 0.924105 |
| lncRNA44226 | "x" | chr11:49283925-49284449 | 150.483 | 212.692 |
| lncRNA12814 | "x" | chr03:53789899-53796491 | 2.18723 | 3.68015 |
| lncRNA14180 | "x" | chr03:9732697-9739046 | 0.729179 | 0.404268 |
| lncRNA25251 | "x" | chr06:1445897-1449428 | 4.40089 | 2.93668 |
| lncRNA45677 | "x" | chr12:41300662-41304105 | 7.21381 | 10.6871 |
| lncRNA10554 | "x" | chr02:40330025-40331355 | 38.5502 | 20.0612 |
| lncRNA08488 | "x" | chr02:43553933-43554808 | 23.9697 | 30.8596 |
| lncRNA13284 | "x" | chr03:60548157-60550304 | 2.75544 | 3.81946 |
| lncRNA44307 | "x" | chr11:50236168-50238658 | 2.04482 | 2.49209 |
| lncRNA07547 | "x" | chr02:32811138-32816106 | 2.13703 | 1.69052 |
| lncRNA42295 | "x" | chr11:47036895-47042310 | 3.37549 | 2.28806 |
| lncRNA33324 | "x" | chr08:56777330-56783818 | 3.92197 | 7.28546 |
| lncRNA45803 | "x" | chr12:45137613-45142570 | 1.27122 | 1.74269 |
| lncRNA36717 | "x" | chr09:60118118-60126053 | 1.35681 | 2.28762 |
| lncRNA06475 | "x" | chr01:86612728-86615007 | 2.08597 | 2.76671 |
| lncRNA24477 | "x" | chr06:39176879-39179596 | 4.40447 | 2.17578 |
| lncRNA37435 | "u" | chr10:2008127-2008452 | 37.9485 | 29.7813 |
| lncRNA37833 | "x" | chr10:22280179-22281092 | 0.693151 | 0.453125 |
| lncRNA41928 | "x" | chr11:23164879-23167068 | 0.62757 | 0.411234 |
| lncRNA02798 | "x" | chr01:77689810-77694407 | 7.08774 | 5.43945 |
| lncRNA18153 | "u" | chr04:2756238-2756835 | 2.56467 | 3.37494 |
| lncRNA34900 | "x" | chr09:58280978-58301208 | 1.26742 | 0.978913 |
| lncRNA45172 | "x" | chr12:5414131-5417709 | 5.35391 | 6.32953 |
| lncRNA45234 | "x" | chr12:6720257-6721618 | 49.0899 | 79.8899 |
| lncRNA02461 | "x" | chr01:73121739-73125023 | 2.63676 | 3.32585 |
| lncRNA08862 | "u" | chr02:47411700-47412919 | 3.59445 | 4.27296 |
| lncRNA32263 | "x" | chr08:1373763-1381568 | 2.11623 | 1.48817 |
| lncRNA25043 | "x" | chr06:44900179-44904605 | 1.36336 | 1.03034 |
| lncRNA26546 | "x" | chr06:40776121-40779022 | 1.58499 | 1.87664 |
| lncRNA40506 | "x" | chr10:60758845-60765016 | 0.862296 | 1.37086 |
| lncRNA43869 | "x" | chr11:35368976-35380150 | 14.1973 | 22.9214 |
| lncRNA13693 | "x" | chr03:64780564-64800816 | 7.17943 | 5.31022 |
| lncRNA31298 | "x" | chr08:51408880-51413600 | 0.754134 | 0.515171 |
| lncRNA08955 | "x" | chr02:48351717-48353125 | 2.10046 | 2.86138 |
| lncRNA21116 | "x" | chr05:59629186-59630954 | 15.4276 | 17.4524 |
| lncRNA20307 | "x" | chr05:5580164-5584303 | 1.60702 | 1.01306 |
| lncRNA33717 | "x" | chr08:61664026-61670068 | 0.814619 | 0.542581 |
| lncRNA45863 | "u" | chr12:46610746-46611295 | 4.63983 | 3.57724 |
| lncRNA14441 | "u" | chr03:24111917-24114637 | 0.955417 | 1.16075 |
| lncRNA24719 | "x" | chr06:41549399-41550391 | 11.0984 | 14.9787 |
| lncRNA25537 | "u" | chr06:16513079-16513403 | 5.5886 | 3.6474 |
| lncRNA32176 | "u" | chr08:338270-338611 | 4.52087 | 2.93861 |
| lncRNA11398 | "x" | chr02:49040401-49041158 | 29.527 | 19.2513 |
| lncRNA24088 | "x" | chr06:34213416-34254456 | 3.25038 | 4.25459 |
| lncRNA41245 | "x" | chr11:2610536-2611404 | 8.16158 | 5.27237 |
| lncRNA14129 | "x" | chr03:9207522-9212045 | 1.00142 | 0.557656 |
| lncRNA45507 | "x" | chr12:31282408-31284648 | 0.721151 | 0.475083 |
| lncRNA01593 | "x" | chr01:19076747-19080220 | 1.33872 | 0.929106 |
| lncRNA39312 | "x" | chr10:2538390-2539305 | 19.4592 | 29.4594 |
| lncRNA48147 | "x" | chr12:63486941-63490867 | 1.68552 | 0.999993 |
| lncRNA41241 | "x" | chr11:2579285-2581469 | 3.31274 | 2.2849 |
| lncRNA42307 | "x" | chr11:47345790-47352691 | 24.7317 | 38.4953 |
| lncRNA13612 | "x" | chr03:63991443-63996129 | 0.817238 | 0.642137 |
| lncRNA10633 | "x" | chr02:41269552-41282409 | 0.522644 | 1.10542 |
| lncRNA17475 | "x" | chr04:58906100-58909496 | 1.21034 | 0.879809 |
| lncRNA40439 | "x" | chr10:60089816-60093292 | 3.22252 | 2.3932 |
| lncRNA46881 | "x" | chr12:3670659-3675514 | 2.07404 | 1.41565 |
| lncRNA39332 | "x" | chr10:2752884-2754679 | 10.3889 | 11.9725 |
| lncRNA21062 | "u" | chr05:58248824-58249276 | 5.51199 | 7.17875 |
| lncRNA02467 | "x" | chr01:73180569-73185697 | 0.790287 | 0.972827 |
| lncRNA06207 | "x" | chr01:83534653-83541901 | 1.53191 | 1.02502 |
| lncRNA28322 | "x" | chr07:60656008-60658304 | 6.72341 | 5.08202 |
| lncRNA38216 | "x" | chr10:50012452-50016896 | 13.6278 | 21.1937 |
| lncRNA17097 | "x" | chr04:52685581-52690885 | 2.86364 | 3.40535 |
| lncRNA43942 | "x" | chr11:37366992-37376073 | 11.0823 | 16.4567 |
| lncRNA08313 | "x" | chr02:41627836-41631881 | 6.58348 | 4.71268 |
| lncRNA39572 | "u" | chr10:12215353-12218660 | 1.36547 | 1.1499 |
| lncRNA26559 | "u" | chr06:40865818-40868497 | 9.45324 | 10.6928 |
| lncRNA27281 | "x" | chr07:2976011-2979543 | 48.0917 | 37.1964 |
| lncRNA48323 | "x" | chr12:64925084-64929069 | 1.76237 | 2.21283 |
| lncRNA47292 | "x" | chr12:23790103-23798010 | 3.16717 | 2.05724 |
| lncRNA06990 | "u" | chr02:13663625-13664528 | 1.81195 | 1.40307 |
| lncRNA17322 | "x" | chr04:56746213-56747341 | 0.958275 | 1.62008 |
| lncRNA31583 | "x" | chr08:56554329-56563200 | 2.0193 | 2.53345 |
| lncRNA27431 | "x" | chr07:7463795-7481340 | 8.9657 | 11.1764 |
| lncRNA33467 | "u" | chr08:58469320-58469682 | 15.0601 | 11.4652 |
| lncRNA28275 | "x" | chr07:60072437-60079579 | 1.63636 | 2.3557 |
| lncRNA33056 | "x" | chr08:52611680-52619577 | 8.86379 | 5.93858 |
| lncRNA43360 | "x" | chr11:5628338-5637037 | 10.6738 | 13.0823 |
| lncRNA25649 | "x" | chr06:22555373-22556321 | 23.9522 | 43.0214 |
| lncRNA43127 | "u" | chr11:3079634-3080566 | 2.38794 | 2.97772 |
| lncRNA30527 | "x" | chr08:763882-768964 | 1.4962 | 1.03226 |
| lncRNA35671 | "u" | chr09:1006614-1020453 | 1.25601 | 0.791064 |
| lncRNA04235 | "x" | chr01:3091051-3093978 | 1.8423 | 1.27421 |
| lncRNA10707 | "x" | chr02:42008367-42020239 | 1.88452 | 1.28304 |
| lncRNA17901 | "u" | chr04:63965274-63965833 | 2.65842 | 3.47696 |
| lncRNA32717 | "u" | chr08:30717056-30718430 | 13.3487 | 15.144 |
| lncRNA37880 | "u" | chr10:27316029-27318747 | 1.48421 | 1.76324 |
| lncRNA07347 | "x" | chr02:29934676-29938091 | 6.02482 | 4.3418 |
| lncRNA36303 | "x" | chr09:32507431-32522286 | 3.40319 | 2.59901 |
| lncRNA47798 | "x" | chr12:47444898-47446807 | 6.52374 | 11.0186 |
| lncRNA19909 | "x" | chr04:64044593-64047428 | 4.71609 | 5.89694 |
| lncRNA12392 | "u" | chr03:44589498-44589909 | 14.5198 | 17.9973 |
| lncRNA33759 | "x" | chr08:62143391-62149197 | 1.1937 | 0.675379 |
| lncRNA31059 | "x" | chr08:39946965-39949816 | 8.63402 | 7.08558 |
| lncRNA00573 | "u" | chr00:6754899-6755833 | 1.41444 | 1.83385 |
| lncRNA19274 | "x" | chr04:56686361-56690115 | 1.1733 | 1.73388 |
| lncRNA36052 | "u" | chr09:8164925-8167091 | 0.998656 | 0.810196 |
| lncRNA35082 | "x" | chr09:61882717-61883551 | 21.0092 | 18.0571 |
| lncRNA36051 | "x" | chr09:8151740-8154870 | 4.56537 | 8.1415 |
| lncRNA04282 | "x" | chr01:4369327-4376294 | 1.03834 | 0.764223 |
| lncRNA29370 | "x" | chr07:35645041-35659176 | 10.522 | 8.89606 |
| lncRNA20070 | "x" | chr05:2160741-2164468 | 4.25129 | 5.62444 |
| lncRNA40566 | "x" | chr10:61293120-61302359 | 1.80247 | 2.37264 |
| lncRNA43690 | "x" | chr11:19639366-19648210 | 3.07397 | 2.55143 |
| lncRNA46734 | "x" | chr12:2183772-2190714 | 1.42916 | 2.99857 |
| lncRNA45604 | "x" | chr12:37136145-37138218 | 13.2128 | 10.1709 |
| lncRNA25700 | "x" | chr06:25998873-26005144 | 1.39429 | 1.64908 |
| lncRNA43250 | "x" | chr11:4539052-4542723 | 5.62904 | 7.10828 |
| lncRNA24899 | "x" | chr06:43535795-43536498 | 19.597 | 16.5285 |
| lncRNA14239 | "u" | chr03:11277850-11278108 | 52.078 | 37.4208 |
| lncRNA25552 | "u" | chr06:17639793-17640294 | 3.1332 | 2.23858 |
| lncRNA32341 | "u" | chr08:2471560-2471978 | 5.34163 | 3.82868 |
| lncRNA16057 | "x" | chr04:3072-9758 | 2.0492 | 2.81428 |
| lncRNA12742 | "x" | chr03:52722062-52729606 | 10.9657 | 8.99188 |
| lncRNA33054 | "x" | chr08:52577242-52578051 | 1.43445 | 2.15706 |
| lncRNA16096 | "x" | chr04:428693-430139 | 1.15882 | 0.753575 |
| lncRNA09239 | "u" | chr02:12498081-12500741 | 0.86196 | 0.701926 |
| lncRNA14406 | "x" | chr03:20451687-20456967 | 89.528 | 63.1312 |
| lncRNA08486 | "x" | chr02:43535368-43537160 | 10.2748 | 7.80246 |
| lncRNA09847 | "x" | chr02:32540130-32544141 | 0.846033 | 0.57648 |
| lncRNA26938 | "x" | chr06:44821207-44827496 | 3.93939 | 4.78447 |
| lncRNA40982 | "x" | chr11:53854-59721 | 1.16003 | 1.40881 |
| lncRNA18809 | "u" | chr04:40165732-40172260 | 1.91324 | 2.17778 |
| lncRNA08051 | "x" | chr02:38960022-38972293 | 0.473088 | 0.913711 |
| lncRNA19571 | "x" | chr04:60559326-60567244 | 17.7118 | 9.93763 |
| lncRNA10624 | "x" | chr02:41214860-41216286 | 2.25464 | 3.15492 |
| lncRNA17985 | "x" | chr04:618399-625090 | 0.569985 | 1.11315 |
| lncRNA10253 | "x" | chr02:36985327-36986044 | 318.259 | 483.73 |
| lncRNA20030 | "x" | chr05:1536689-1542139 | 1.01311 | 0.793396 |
| lncRNA11057 | "x" | chr02:45465910-45470585 | 4.38633 | 3.858 |
| lncRNA37061 | "u" | chr09:65088723-65089640 | 1.10601 | 1.44315 |
| lncRNA43556 | "x" | chr11:11747787-11753122 | 8.37755 | 14.8548 |
| lncRNA46723 | "x" | chr12:2087449-2089503 | 8.67424 | 7.1571 |
| lncRNA32295 | "x" | chr08:1824234-1828359 | 4.92095 | 7.17604 |
| lncRNA18770 | "x" | chr04:37712980-37716923 | 4.92563 | 7.98162 |
| lncRNA47092 | "x" | chr12:6467281-6473512 | 0.901189 | 0.769869 |
| lncRNA11154 | "x" | chr02:46568707-46573829 | 12.201 | 15.7415 |
| lncRNA15336 | "x" | chr03:57046296-57050443 | 1.09013 | 0.7052 |
| lncRNA01115 | "u" | chr01:440659-441599 | 1.32984 | 1.72839 |
| lncRNA23039 | "u" | chr05:63653342-63653656 | 56.2311 | 45.152 |
| lncRNA37788 | "x" | chr10:19661876-19666448 | 0.792239 | 0.51128 |
| lncRNA38735 | "u" | chr10:61594055-61594397 | 37.7993 | 30.7355 |
| lncRNA32400 | "x" | chr08:3113896-3119821 | 0.432216 | 0.650485 |
| lncRNA32102 | "x" | chr08:62669478-62675212 | 3.72274 | 4.95399 |
| lncRNA43240 | "x" | chr11:4456052-4460503 | 27.7174 | 21.1355 |
| lncRNA08517 | "u" | chr02:43868734-43869425 | 2.29429 | 1.75831 |
| lncRNA26084 | "x" | chr06:35317422-35324636 | 11.0074 | 6.45793 |
| lncRNA30869 | "x" | chr08:11992310-12010413 | 3.48065 | 2.99109 |
| lncRNA35192 | "x" | chr09:63509208-63514374 | 2.10115 | 2.82969 |
| lncRNA17356 | "x" | chr04:57169565-57171207 | 0.971815 | 0.746902 |
| lncRNA38950 | "x" | chr10:63720056-63721795 | 9.34653 | 11.8361 |
| lncRNA45302 | "x" | chr12:10403029-10411464 | 1.12094 | 0.937977 |
| lncRNA26245 | "x" | chr06:37237365-37238838 | 26.1909 | 20.7862 |
| lncRNA11050 | "x" | chr02:45434850-45440326 | 10.6417 | 8.90942 |
| lncRNA46471 | "u" | chr12:65161028-65162030 | 1.06148 | 0.79457 |
| lncRNA18182 | "x" | chr04:3052310-3055413 | 1.32704 | 1.08002 |
| lncRNA42872 | "x" | chr11:566241-569198 | 13.9359 | 10.5376 |
| lncRNA27906 | "x" | chr07:53814475-53820388 | 402.974 | 269.091 |
| lncRNA35475 | "x" | chr09:66898402-66901851 | 7.28058 | 5.99098 |
| lncRNA22301 | "u" | chr05:20706480-20708901 | 16.1259 | 18.0851 |
| lncRNA43194 | "x" | chr11:3721104-3724626 | 2.03507 | 2.6433 |
| lncRNA05698 | "x" | chr01:77752794-77757781 | 3.67197 | 5.81648 |
| lncRNA31302 | "x" | chr08:51504565-51505950 | 89.74 | 71.0004 |
| lncRNA16920 | "x" | chr04:44908946-44913382 | 3.61934 | 3.18396 |
| lncRNA30074 | "x" | chr07:61029814-61034603 | 3.38762 | 4.13188 |
| lncRNA06291 | "x" | chr01:84506542-84509427 | 0.870159 | 0.667313 |
| lncRNA08653 | "x" | chr02:45173928-45183799 | 11.8749 | 9.66612 |
| lncRNA35055 | "x" | chr09:61508699-61511771 | 1.5166 | 2.21177 |
| lncRNA47878 | "x" | chr12:49501288-49506975 | 22.7214 | 31.3155 |
| lncRNA20986 | "x" | chr05:55424513-55451601 | 1.97565 | 3.08444 |
| lncRNA07033 | "u" | chr02:15416495-15417997 | 0.749863 | 0.964485 |
| lncRNA16522 | "x" | chr04:6218670-6220398 | 74.3801 | 63.3021 |
| lncRNA32250 | "x" | chr08:1252317-1257044 | 0.556791 | 0.86448 |
| lncRNA47570 | "x" | chr12:42688644-42703753 | 9.16916 | 10.8721 |
| lncRNA21648 | "x" | chr05:1552684-1555321 | 1.37444 | 0.860696 |
| lncRNA10277 | "x" | chr02:37348725-37350630 | 4.31926 | 3.32078 |
| lncRNA14222 | "u" | chr03:10673144-10674882 | 0.784101 | 0.612368 |
| lncRNA33237 | "u" | chr08:55399694-55401088 | 0.941728 | 0.734869 |
| lncRNA08151 | "x" | chr02:40021707-40034066 | 0.436777 | 0.706721 |
| lncRNA33591 | "x" | chr08:60129738-60131640 | 6.22943 | 9.40626 |
| lncRNA05772 | "x" | chr01:78675421-78679108 | 2.98382 | 1.80187 |
| lncRNA33055 | "x" | chr08:52581190-52586810 | 0.716985 | 0.606381 |
| lncRNA40144 | "x" | chr10:52372077-52383992 | 0.823287 | 0.592564 |
| lncRNA34316 | "u" | chr09:7306406-7309201 | 2.98216 | 3.43531 |
| lncRNA12269 | "x" | chr03:31895327-31898262 | 0.730323 | 0.887589 |
| lncRNA38861 | "x" | chr10:62957400-62961163 | 2.0727 | 3.15831 |
| lncRNA40866 | "x" | chr10:63930626-63935091 | 1.21928 | 1.70127 |
| lncRNA23197 | "x" | chr06:331014-352825 | 1.24228 | 2.44218 |
| lncRNA26512 | "x" | chr06:40330070-40335137 | 0.743445 | 0.463803 |
| lncRNA32711 | "x" | chr08:30099707-30100428 | 1.45876 | 1.90759 |
| lncRNA12363 | "u" | chr03:43627240-43627701 | 104.611 | 92.2174 |
| lncRNA30894 | "x" | chr08:16981139-17000668 | 7.61442 | 6.49714 |
| lncRNA16543 | "u" | chr04:6936238-6941824 | 2.56409 | 3.09481 |
| lncRNA44629 | "x" | chr12:10433-11752 | 1.07317 | 0.696467 |
| lncRNA41510 | "x" | chr11:5682713-5696581 | 1.0761 | 1.46741 |
| lncRNA45401 | "x" | chr12:21200022-21227856 | 3.67789 | 5.1593 |
| lncRNA07583 | "o" | chr02:33312431-33337659 | 1.49965 | 1.11344 |
| lncRNA16075 | "x" | chr04:195807-199205 | 36.3858 | 25.8181 |
| lncRNA41096 | "x" | chr11:1161597-1163447 | 4.51435 | 3.42223 |
| lncRNA41443 | "x" | chr11:5022031-5025981 | 3.69175 | 2.0497 |
| lncRNA47778 | "j" | chr12:47223223-47227759 | 6.68837 | 7.78273 |
| lncRNA22964 | "x" | chr05:62725403-62728002 | 0.373516 | 0.778894 |
| lncRNA44221 | "x" | chr11:49204722-49206188 | 12.1175 | 7.13216 |
| lncRNA05709 | "x" | chr01:77864546-77873889 | 1.39687 | 2.0579 |
| lncRNA13575 | "u" | chr03:63621076-63623684 | 3.40716 | 5.24881 |
| lncRNA38155 | "x" | chr10:47041421-47052335 | 2.79926 | 4.16013 |
| lncRNA44722 | "x" | chr12:705204-710291 | 1.83135 | 1.49756 |
| lncRNA46613 | "x" | chr12:824007-829745 | 0.489608 | 0.686631 |
| lncRNA18312 | "x" | chr04:4825798-4832573 | 2.26124 | 1.73232 |
| lncRNA24064 | "x" | chr06:33820602-33828091 | 46.9929 | 30.7966 |
| lncRNA44879 | "x" | chr12:2194866-2198052 | 8.7198 | 7.12575 |
| lncRNA32466 | "u" | chr08:5602219-5603369 | 1.3108 | 1.64035 |
| lncRNA32388 | "x" | chr08:2962191-2966912 | 1.29766 | 0.8845 |
| lncRNA29529 | "x" | chr07:51459057-51465488 | 1.9628 | 2.41606 |
| lncRNA10999 | "x" | chr02:45015852-45025052 | 3.19808 | 4.05859 |
| lncRNA47440 | "x" | chr12:35197505-35216182 | 2.7299 | 3.3479 |
| lncRNA08255 | "u" | chr02:41155163-41158612 | 1.05359 | 0.886427 |
| lncRNA31706 | "x" | chr08:58342091-58346823 | 0.823781 | 0.507354 |
| lncRNA01313 | "x" | chr01:2765226-2774729 | 1.35595 | 0.934919 |
| lncRNA44379 | "u" | chr11:51004826-51005246 | 28.2363 | 33.2921 |
| lncRNA46919 | "x" | chr12:4132344-4139148 | 13.8285 | 18.1376 |
| lncRNA46868 | "x" | chr12:3547558-3550131 | 1.35857 | 1.86389 |
| lncRNA29947 | "x" | chr07:59779015-59780307 | 1.95624 | 1.02578 |
| lncRNA41689 | "x" | chr11:9788866-9802965 | 15.3219 | 19.8761 |
| lncRNA26599 | "x" | chr06:41252094-41253372 | 2.15118 | 3.26317 |
| lncRNA36553 | "x" | chr09:55904413-55906599 | 0.78156 | 1.5191 |
| lncRNA08972 | "u" | chr02:48541501-48541870 | 22.8902 | 18.6617 |
| lncRNA45613 | "x" | chr12:37670839-37688024 | 4.5402 | 5.28655 |
| lncRNA45151 | "x" | chr12:5027573-5032069 | 5.68172 | 8.61151 |
| lncRNA48243 | "x" | chr12:64280960-64285394 | 6.72864 | 5.31566 |
| lncRNA07774 | "x" | chr02:35775650-35781006 | 1.14683 | 0.94989 |
| lncRNA30979 | "u" | chr08:28684596-28685329 | 1.45181 | 1.89848 |
| lncRNA30902 | "u" | chr08:17276641-17279182 | 0.90922 | 0.751878 |
| lncRNA05192 | "x" | chr01:70468063-70470215 | 19.1568 | 21.8829 |
| lncRNA30145 | "x" | chr07:62054707-62058543 | 4.54929 | 3.20889 |
| lncRNA01547 | "x" | chr01:13976403-13992385 | 1.45578 | 1.98856 |
| lncRNA46286 | "x" | chr12:63696931-63704739 | 1.99008 | 2.75221 |
| lncRNA23571 | "x" | chr06:16057616-16067362 | 2.21647 | 1.85653 |
| lncRNA18218 | "x" | chr04:3502066-3507108 | 29.8436 | 26.5142 |
| lncRNA40375 | "x" | chr10:59571653-59580145 | 10.333 | 6.2439 |
| lncRNA47381 | "u" | chr12:31120029-31121566 | 1.42179 | 1.1421 |
| lncRNA17772 | "x" | chr04:62473042-62478543 | 1.19982 | 0.806203 |
| lncRNA18611 | "u" | chr04:23177059-23177296 | 255.42 | 208.84 |
| lncRNA29137 | "u" | chr07:7713809-7714308 | 6.16363 | 7.67134 |
| lncRNA19350 | "x" | chr04:57525978-57528627 | 3.38416 | 4.09194 |
| lncRNA28948 | "x" | chr07:2356041-2360542 | 2.12945 | 1.75886 |
| lncRNA40607 | "x" | chr10:61667782-61677647 | 11.4766 | 14.4979 |
| lncRNA45795 | "x" | chr12:44891829-44895545 | 1.0529 | 0.706156 |
| lncRNA34444 | "x" | chr09:16650955-16659447 | 1.12073 | 0.726743 |
| lncRNA05797 | "u" | chr01:78925176-78926324 | 2.65599 | 3.20047 |
| lncRNA02784 | "x" | chr01:77576592-77580139 | 9.52431 | 13.6051 |
| lncRNA08640 | "x" | chr02:45066560-45073107 | 3.45625 | 3.03623 |
| lncRNA13610 | "x" | chr03:63978805-63981110 | 31.5322 | 42.038 |
| lncRNA45152 | "x" | chr12:5033556-5040335 | 6.69434 | 8.60461 |
| lncRNA39365 | "x" | chr10:3196500-3199644 | 0.742306 | 0.522043 |
| lncRNA33189 | "x" | chr08:54869207-54872476 | 3.42563 | 4.3199 |
| lncRNA10078 | "x" | chr02:35032800-35046444 | 0.792375 | 1.25091 |
| lncRNA23931 | "u" | chr06:32019170-32021034 | 1.16232 | 0.942344 |
| lncRNA06652 | "u" | chr01:88413170-88414049 | 1.63713 | 1.26238 |
| lncRNA20028 | "x" | chr05:1492864-1493716 | 4.66387 | 3.67862 |
| lncRNA39047 | "x" | chr10:64402546-64404803 | 92.8284 | 131.218 |
| lncRNA44109 | "x" | chr11:47135687-47142683 | 4.77869 | 5.94755 |
| lncRNA42840 | "x" | chr11:227134-228057 | 2.59169 | 1.50602 |
| lncRNA03832 | "x" | chr01:88708718-88721589 | 5.43333 | 7.59686 |
| lncRNA28867 | "x" | chr07:1221695-1224155 | 19.9503 | 16.6836 |
| lncRNA41944 | "u" | chr11:25062696-25065069 | 0.849936 | 0.696495 |
| lncRNA10983 | "x" | chr02:44884613-44890250 | 0.579878 | 0.815797 |
| lncRNA28222 | "x" | chr07:59257197-59261292 | 0.683229 | 0.950777 |
| lncRNA25437 | "x" | chr06:4875172-4878173 | 3.85358 | 3.34583 |
| lncRNA42219 | "x" | chr11:45479726-45480789 | 18.0701 | 14.8733 |
| lncRNA23113 | "x" | chr05:64407059-64413767 | 1.62652 | 2.48461 |
| lncRNA03767 | "x" | chr01:87873330-87876049 | 2.13426 | 3.28546 |
| lncRNA18742 | "u" | chr04:32698007-32701834 | 1.68252 | 1.45084 |
| lncRNA44691 | "x" | chr12:414719-427014 | 3.89362 | 3.17946 |
| lncRNA46827 | "x" | chr12:3196637-3201576 | 2.78734 | 2.07428 |
| lncRNA34287 | "x" | chr09:6032410-6039150 | 11.0173 | 9.89804 |
| lncRNA04833 | "u" | chr01:56353664-56355725 | 1.13543 | 0.934292 |
| lncRNA42666 | "x" | chr11:51893417-51896905 | 2.80967 | 2.14497 |
| lncRNA43191 | "x" | chr11:3691034-3704230 | 4.92145 | 3.7215 |
| lncRNA06009 | "x" | chr01:81375621-81379067 | 1.70937 | 3.31337 |
| lncRNA25986 | "x" | chr06:33708200-33710989 | 4.93471 | 6.11966 |
| lncRNA37076 | "x" | chr09:65304825-65309530 | 5.51887 | 2.88077 |
| lncRNA03369 | "x" | chr01:83937776-83946456 | 3.55059 | 2.63612 |
| lncRNA23216 | "x" | chr06:546429-550360 | 0.888049 | 0.556804 |
| lncRNA25740 | "x" | chr06:28241779-28244261 | 8.95764 | 10.6304 |
| lncRNA35975 | "x" | chr09:5513118-5516464 | 2.37453 | 1.84202 |
| lncRNA39914 | "x" | chr10:43617220-43626899 | 0.742483 | 0.521128 |
| lncRNA48208 | "x" | chr12:63969252-63971720 | 0.723279 | 1.09886 |
| lncRNA17125 | "x" | chr04:53301447-53306177 | 8.42699 | 6.21846 |
| lncRNA22963 | "x" | chr05:62715753-62722080 | 3.14615 | 1.93146 |
| lncRNA10528 | "x" | chr02:40082179-40089741 | 4.31388 | 5.33678 |
| lncRNA29515 | "x" | chr07:50732372-50744523 | 2.43537 | 1.69516 |
| lncRNA36603 | "x" | chr09:57355328-57358269 | 60.5638 | 76.6861 |
| lncRNA02351 | "x" | chr01:71438379-71444159 | 1.37489 | 2.14765 |
| lncRNA44957 | "x" | chr12:3004821-3008886 | 67.181 | 89.4881 |
| lncRNA28615 | "x" | chr07:63922766-63927583 | 6.67793 | 5.23788 |
| lncRNA13093 | "x" | chr03:58479806-58480390 | 2.66962 | 1.86774 |
| lncRNA37848 | "x" | chr10:23228315-23235285 | 2.90825 | 3.78421 |
| lncRNA40332 | "x" | chr10:59213209-59214274 | 7.13829 | 3.66094 |
| lncRNA43501 | "x" | chr11:9347644-9356782 | 6.88298 | 4.88131 |
| lncRNA26024 | "x" | chr06:34359017-34368653 | 20.1255 | 17.6453 |
| lncRNA44706 | "x" | chr12:593192-597651 | 1.78306 | 1.08725 |
| lncRNA44701 | "x" | chr12:553932-559357 | 7.03351 | 10.4086 |
| lncRNA32943 | "x" | chr08:49555123-49557702 | 1.43819 | 0.750899 |
| lncRNA05787 | "u" | chr01:78799371-78799878 | 5.23652 | 4.11531 |
| lncRNA30021 | "x" | chr07:60502723-60503396 | 2.23553 | 2.90961 |
| lncRNA45849 | "x" | chr12:46262683-46267398 | 17.3211 | 14.0108 |
| lncRNA35506 | "x" | chr09:67174781-67177251 | 4.35958 | 5.26518 |
| lncRNA38478 | "x" | chr10:59216337-59218670 | 1.16302 | 1.46174 |
| lncRNA28868 | "x" | chr07:1229953-1231307 | 2.77241 | 1.82044 |
| lncRNA13738 | "x" | chr03:492525-500547 | 1.70211 | 1.31349 |
| lncRNA24120 | "x" | chr06:34870272-34875164 | 5.65501 | 4.57292 |
| lncRNA04185 | "x" | chr01:2463093-2470694 | 2.77445 | 1.90192 |
| lncRNA26890 | "x" | chr06:44426966-44432209 | 3.89234 | 2.92269 |
| lncRNA30211 | "x" | chr07:62696419-62698764 | 4.3625 | 5.69184 |
| lncRNA26871 | "x" | chr06:44208851-44212836 | 0.819997 | 0.570212 |
| lncRNA19542 | "x" | chr04:60294923-60296077 | 1.31589 | 0.853266 |
| lncRNA19603 | "x" | chr04:60925633-60926316 | 91.429 | 74.5427 |
| lncRNA23792 | "x" | chr06:28437985-28441902 | 1.48708 | 1.90677 |
| lncRNA17861 | "x" | chr04:63487951-63491962 | 6.50159 | 8.14239 |
| lncRNA38095 | "x" | chr10:43547063-43551461 | 52.8607 | 33.2431 |
| lncRNA44478 | "x" | chr11:52044786-52049694 | 3.71528 | 6.0831 |
| lncRNA39930 | "u" | chr10:45266453-45266983 | 2.13314 | 1.53881 |
| lncRNA12204 | "x" | chr03:26231105-26232987 | 2.88646 | 2.33506 |
| lncRNA39795 | "x" | chr10:35843119-35848544 | 14.013 | 18.9617 |
| lncRNA46943 | "x" | chr12:4417406-4424160 | 7.83332 | 10.8543 |
| lncRNA18273 | "x" | chr04:4367411-4376060 | 3.52417 | 5.40333 |
| lncRNA35945 | "u" | chr09:4954452-4955041 | 2.08751 | 2.72817 |
| lncRNA03789 | "x" | chr01:88111928-88114394 | 2.41842 | 3.48255 |
| lncRNA08928 | "x" | chr02:48062612-48065896 | 1.26944 | 2.46908 |
| lncRNA17958 | "u" | chr04:367919-368801 | 8.80809 | 10.1845 |
| lncRNA39651 | "x" | chr10:20931188-20937092 | 1.68461 | 1.34706 |
| lncRNA21004 | "u" | chr05:56213338-56213802 | 4.08184 | 5.30236 |
| lncRNA27886 | "u" | chr07:53584361-53585478 | 0.806288 | 1.04769 |
| lncRNA33465 | "u" | chr08:58431372-58434179 | 1.99404 | 1.70984 |
| lncRNA07389 | "x" | chr02:30629516-30635896 | 2.82208 | 4.46482 |
| lncRNA08316 | "u" | chr02:41682948-41683792 | 61.6448 | 54.9962 |
| lncRNA45664 | "x" | chr12:40775611-40782991 | 17.386 | 22.2855 |
| lncRNA22871 | "x" | chr05:61505373-61513495 | 1.48459 | 1.14669 |
| lncRNA31804 | "x" | chr08:59546612-59551611 | 3.90911 | 3.0981 |
| lncRNA17665 | "x" | chr04:61207893-61212920 | 4.25241 | 5.92931 |
| lncRNA38696 | "x" | chr10:61207994-61211985 | 5.83148 | 7.73079 |
| lncRNA43162 | "x" | chr11:3323399-3327178 | 0.538893 | 0.868695 |
| lncRNA34118 | "x" | chr09:3382754-3395367 | 0.623065 | 0.828301 |
| lncRNA44819 | "x" | chr12:1616277-1625594 | 7.55863 | 5.81142 |
| lncRNA37917 | "x" | chr10:30366966-30369544 | 40.7897 | 49.4281 |
| lncRNA13141 | "x" | chr03:59021833-59027357 | 1.1508 | 0.763873 |
| lncRNA34477 | "x" | chr09:18302109-18307942 | 3.04808 | 3.49767 |
| lncRNA42477 | "x" | chr11:49862706-49864048 | 64.3268 | 39.2794 |
| lncRNA15480 | "u" | chr03:58677637-58677933 | 83.4902 | 99.9962 |
| lncRNA19925 | "x" | chr05:179198-183983 | 1.51754 | 1.12352 |
| lncRNA02401 | "x" | chr01:72407171-72413247 | 2.57881 | 2.18342 |
| lncRNA40327 | "x" | chr10:59148139-59170739 | 2.94579 | 3.65692 |
| lncRNA34909 | "o" | chr09:58665174-58666303 | 42.3146 | 47.0149 |
| lncRNA34564 | "x" | chr09:27232730-27236005 | 38.9001 | 30.7503 |
| lncRNA37844 | "x" | chr10:22992016-22995517 | 1.49236 | 1.9023 |
| lncRNA46815 | "x" | chr12:3091397-3093554 | 2.39648 | 2.08514 |
| lncRNA19312 | "x" | chr04:57131446-57136751 | 0.615621 | 1.03986 |
| lncRNA16056 | "x" | chr04:3072-9758 | 0.506186 | 0.704264 |
| lncRNA05676 | "x" | chr01:77458345-77468495 | 1.32079 | 0.97301 |
| lncRNA25471 | "x" | chr06:8019244-8025806 | 8.6897 | 9.85567 |
| lncRNA05554 | "x" | chr01:75844141-75847640 | 1.77674 | 1.38279 |
| lncRNA37263 | "x" | chr09:67569665-67573987 | 1.52849 | 2.06499 |
| lncRNA32048 | "x" | chr08:62203728-62206170 | 1.68784 | 1.23856 |
| lncRNA32790 | "o" | chr08:40542912-40551032 | 3.24265 | 4.2474 |
| lncRNA42426 | "x" | chr11:49272781-49276760 | 10.4127 | 13.1458 |
| lncRNA20535 | "x" | chr05:12063103-12066641 | 3.90148 | 4.38603 |
| lncRNA39030 | "x" | chr10:64289815-64294227 | 33.7019 | 37.9014 |
| lncRNA46872 | "x" | chr12:3593794-3597228 | 1.61544 | 1.27484 |
| lncRNA48298 | "x" | chr12:64706409-64710756 | 12.1739 | 16.2658 |
| lncRNA38527 | "x" | chr10:59665048-59668393 | 5.52305 | 3.95192 |
| lncRNA47641 | "x" | chr12:44198949-44201583 | 10.9585 | 14.7428 |
| lncRNA00360 | "u" | chr00:16023137-16023834 | 1.26223 | 0.919931 |
| lncRNA46236 | "x" | chr12:63252594-63256413 | 0.865604 | 1.15117 |
| lncRNA47366 | "x" | chr12:29804399-29807973 | 8.75916 | 11.3238 |
| lncRNA29831 | "x" | chr07:58243155-58248190 | 5.06404 | 3.3239 |
| lncRNA06220 | "x" | chr01:83685196-83733759 | 2.45898 | 3.51041 |
| lncRNA12676 | "x" | chr03:51110185-51115541 | 2.48592 | 2.15243 |
| lncRNA42237 | "u" | chr11:45781769-45783203 | 0.992717 | 1.23827 |
| lncRNA04852 | "u" | chr01:56993584-56997276 | 1.87302 | 2.28479 |
| lncRNA44876 | "x" | chr12:2150251-2155983 | 1.19074 | 1.01063 |
| lncRNA42429 | "x" | chr11:49289030-49289890 | 16.2808 | 18.7789 |
| lncRNA03213 | "x" | chr01:82200889-82204243 | 5.97474 | 5.13197 |
| lncRNA42319 | "x" | chr11:47499431-47502280 | 3.04324 | 2.51404 |
| lncRNA08930 | "x" | chr02:48071386-48076554 | 1.33256 | 2.59113 |
| lncRNA21915 | "x" | chr05:5462849-5468978 | 13.3116 | 10.4952 |
| lncRNA24414 | "x" | chr06:38551840-38555343 | 0.509259 | 0.7976 |
| lncRNA29949 | "x" | chr07:59810020-59812746 | 116.84 | 70.3132 |
| lncRNA13994 | "x" | chr03:7376052-7384703 | 1.72462 | 1.1779 |
| lncRNA33152 | "x" | chr08:54226393-54232365 | 20.188 | 17.24 |
| lncRNA47930 | "u" | chr12:55111079-55111363 | 69.8451 | 86.6152 |
| lncRNA37067 | "x" | chr09:65131584-65137345 | 9.73275 | 14.9339 |
| lncRNA45666 | "x" | chr12:40775611-40782991 | 0.832072 | 0.59323 |
| lncRNA31348 | "x" | chr08:52490702-52497569 | 2.06769 | 1.39896 |
| lncRNA43403 | "x" | chr11:6343390-6344558 | 2.92713 | 3.47239 |
| lncRNA41370 | "x" | chr11:4138864-4144532 | 1.79181 | 1.05503 |
| lncRNA04954 | "u" | chr01:62791498-62792878 | 3.086 | 2.62002 |
| lncRNA20250 | "x" | chr05:4521362-4525290 | 6.99162 | 7.78657 |
| lncRNA36821 | "x" | chr09:62013934-62019436 | 8.24698 | 6.28279 |
| lncRNA47814 | "x" | chr12:47655420-47661612 | 7.07838 | 4.39033 |
| lncRNA34865 | "x" | chr09:57414145-57418748 | 7.70357 | 10.4915 |
| lncRNA33027 | "x" | chr08:51839179-51851486 | 5.36844 | 7.36541 |
| lncRNA44614 | "x" | chr11:53300558-53304883 | 4.27241 | 5.32897 |
| lncRNA06146 | "x" | chr01:82854585-82858807 | 1.53295 | 1.1536 |
| lncRNA23923 | "x" | chr06:31701202-31701846 | 48.3123 | 38.6267 |
| lncRNA37338 | "x" | chr10:899293-908860 | 2.92127 | 2.51048 |
| lncRNA43893 | "u" | chr11:35868773-35869831 | 1.11984 | 0.869998 |
| lncRNA47001 | "x" | chr12:5099924-5102374 | 6.78698 | 9.32289 |
| lncRNA30566 | "x" | chr08:1184262-1187021 | 8.02932 | 5.303 |
| lncRNA32090 | "u" | chr08:62561432-62566129 | 4.47394 | 3.83543 |
| lncRNA39890 | "x" | chr10:42439174-42449952 | 1.84041 | 1.37636 |
| lncRNA23235 | "x" | chr06:864328-868988 | 6.45512 | 4.61924 |
| lncRNA41459 | "x" | chr11:5131714-5133645 | 9.8005 | 12.3924 |
| lncRNA45358 | "x" | chr12:15670454-15671576 | 52.5984 | 58.2311 |
| lncRNA38328 | "x" | chr10:52882125-52883283 | 3.07752 | 2.33467 |
| lncRNA10062 | "x" | chr02:34928631-34932333 | 4.5332 | 3.80662 |
| lncRNA15420 | "x" | chr03:58080122-58085965 | 1.51272 | 2.30829 |
| lncRNA17893 | "x" | chr04:63900806-63904450 | 6.57985 | 5.59289 |
| lncRNA10892 | "x" | chr02:43930485-43935980 | 2.67175 | 1.69662 |
| lncRNA47474 | "x" | chr12:36155148-36158757 | 5.37489 | 6.54044 |
| lncRNA47791 | "x" | chr12:47352218-47358594 | 5.93468 | 7.2656 |
| lncRNA12361 | "x" | chr03:43618092-43619038 | 6.66567 | 7.75607 |
| lncRNA18621 | "x" | chr04:24590323-24590991 | 155.336 | 131.177 |
| lncRNA47654 | "x" | chr12:44578158-44581940 | 1.45302 | 0.939978 |
| lncRNA31747 | "x" | chr08:58838679-58845331 | 0.729458 | 0.485149 |
| lncRNA15576 | "x" | chr03:59594179-59595693 | 22.1787 | 28.6705 |
| lncRNA46706 | "x" | chr12:1931970-1934991 | 9.18603 | 4.76733 |
| lncRNA44959 | "x" | chr12:3016035-3020599 | 4.34098 | 6.27656 |
| lncRNA10815 | "x" | chr02:43075014-43078764 | 2.44173 | 1.70434 |
| lncRNA33037 | "u" | chr08:52093350-52094324 | 15.6407 | 17.6629 |
| lncRNA05762 | "x" | chr01:78575598-78579217 | 0.705735 | 0.406544 |
| lncRNA00320 | "x" | chr00:15164227-15165268 | 4.26931 | 2.22171 |
| lncRNA07937 | "u" | chr02:37602875-37603862 | 0.86539 | 1.12339 |
| lncRNA12486 | "x" | chr03:46326979-46330040 | 7.56096 | 8.42292 |
| lncRNA26603 | "x" | chr06:41277399-41284709 | 6.82484 | 8.16318 |
| lncRNA09200 | "x" | chr02:8085545-8097243 | 1.5832 | 1.4092 |
| lncRNA03559 | "x" | chr01:85825318-85831189 | 0.698137 | 1.46955 |
| lncRNA08670 | "x" | chr02:45322489-45329077 | 2.55792 | 1.88655 |
| lncRNA17502 | "x" | chr04:59294994-59304028 | 3.05491 | 4.17503 |
| lncRNA24588 | "u" | chr06:40372207-40373164 | 1.39859 | 1.09482 |
| lncRNA40976 | "x" | chr10:64825438-64829000 | 14.7872 | 20.0087 |
| lncRNA39290 | "x" | chr10:2144876-2151960 | 0.642241 | 0.439352 |
| lncRNA47132 | "x" | chr12:8921449-8935284 | 4.69718 | 6.30793 |
| lncRNA46660 | "x" | chr12:1371354-1375476 | 6.93386 | 5.85355 |
| lncRNA43242 | "x" | chr11:4461422-4472459 | 1.78376 | 2.69532 |
| lncRNA21118 | "x" | chr05:59651298-59658483 | 5.21296 | 3.91107 |
| lncRNA00430 | "x" | chr00:17945600-17948327 | 4.69684 | 4.09329 |
| lncRNA15660 | "x" | chr03:60525013-60526875 | 1.05863 | 1.44418 |
| lncRNA20648 | "x" | chr05:25873695-25879668 | 46.0508 | 61.687 |
| lncRNA46822 | "u" | chr12:3141107-3143469 | 0.98795 | 0.816372 |
| lncRNA33490 | "x" | chr08:58906172-58907645 | 4.40141 | 6.65796 |
| lncRNA20699 | "x" | chr05:28060780-28074769 | 268.836 | 131.93 |
| lncRNA43184 | "x" | chr11:3652322-3654101 | 6.28662 | 8.48715 |
| lncRNA44431 | "x" | chr11:51622745-51623350 | 2.89257 | 3.782 |
| lncRNA47297 | "x" | chr12:23997245-24003471 | 23.1484 | 14.6115 |
| lncRNA38605 | "x" | chr10:60410500-60414275 | 2.52802 | 3.97492 |
| lncRNA04363 | "u" | chr01:6665555-6666523 | 0.852208 | 1.10802 |
| lncRNA15680 | "x" | chr03:60787313-60791258 | 1.17464 | 1.60379 |
| lncRNA21289 | "x" | chr05:62180004-62182916 | 1.86444 | 1.52757 |
| lncRNA20739 | "x" | chr05:30510863-30515887 | 0.721849 | 1.21904 |
| lncRNA32722 | "u" | chr08:31431559-31432357 | 3.12178 | 3.77544 |
| lncRNA40567 | "x" | chr10:61306956-61310447 | 3.59825 | 4.76396 |
| lncRNA05681 | "x" | chr01:77543954-77553574 | 1.52854 | 2.34812 |
| lncRNA21094 | "x" | chr05:58997061-59001737 | 3.02716 | 2.094 |
| lncRNA07091 | "x" | chr02:17855019-17855764 | 14.3258 | 16.4303 |
| lncRNA07884 | "x" | chr02:36971743-36973998 | 5.74686 | 7.1134 |
| lncRNA43769 | "u" | chr11:27429597-27432559 | 0.83255 | 0.99317 |
| lncRNA48092 | "x" | chr12:62982333-62985416 | 12.5857 | 14.9027 |
| lncRNA47376 | "x" | chr12:30766662-30769099 | 11.1831 | 10.0345 |
| lncRNA24903 | "x" | chr06:43563646-43569037 | 0.852752 | 0.441315 |
| lncRNA33752 | "x" | chr08:62016441-62021205 | 11.988 | 8.21473 |
| lncRNA19764 | "x" | chr04:62536298-62548561 | 1.88141 | 3.05054 |
| lncRNA42813 | "x" | chr11:53376248-53382033 | 2.93298 | 4.04015 |
| lncRNA16451 | "x" | chr04:4961927-4964016 | 2.76489 | 2.18917 |
| lncRNA15555 | "x" | chr03:59340663-59343595 | 3.1956 | 4.39023 |
| lncRNA44428 | "x" | chr11:51605381-51608210 | 12.3363 | 14.8141 |
| lncRNA42612 | "x" | chr11:51368840-51371270 | 1.7425 | 1.48127 |
| lncRNA27459 | "x" | chr07:8282474-8294010 | 6.42465 | 8.20146 |
| lncRNA13803 | "x" | chr03:1128701-1130011 | 18.4796 | 13.0565 |
| lncRNA15245 | "x" | chr03:55978824-55983439 | 1.05372 | 1.80864 |
| lncRNA39451 | "x" | chr10:5090004-5094094 | 1.22115 | 0.951571 |
| lncRNA45916 | "x" | chr12:47593772-47597862 | 4.92264 | 4.4025 |
| lncRNA20479 | "u" | chr05:9429969-9431760 | 1.77689 | 2.08495 |
| lncRNA36926 | "x" | chr09:63298830-63303187 | 6.60028 | 3.4517 |
| lncRNA40555 | "x" | chr10:61213282-61216146 | 4.58176 | 5.5579 |
| lncRNA27075 | "x" | chr07:213547-218992 | 0.411373 | 0.623175 |
| lncRNA23841 | "x" | chr06:29766989-29768250 | 2.51316 | 3.47144 |
| lncRNA39967 | "x" | chr10:46393441-46401288 | 6.20769 | 7.71806 |
| lncRNA05817 | "x" | chr01:79261733-79268461 | 1.93834 | 1.46721 |
| lncRNA29860 | "x" | chr07:58613766-58616039 | 0.76644 | 0.926554 |
| lncRNA03380 | "x" | chr01:84064095-84066135 | 11.6154 | 9.01816 |
| lncRNA18578 | "x" | chr04:20486363-20492335 | 2.28983 | 1.42141 |
| lncRNA36905 | "x" | chr09:62880221-62885972 | 22.6766 | 19.7481 |
| lncRNA44705 | "x" | chr12:593192-597651 | 7.96932 | 6.72429 |
| lncRNA17024 | "x" | chr04:50325232-50331365 | 0.671494 | 0.870871 |
| lncRNA19698 | "x" | chr04:61950020-61955010 | 1.01648 | 0.698745 |
| lncRNA33114 | "x" | chr08:53702694-53705401 | 0.882718 | 1.43001 |
| lncRNA42754 | "x" | chr11:52667621-52692241 | 52.1313 | 67.1315 |
| lncRNA47632 | "x" | chr12:43908613-43915165 | 70.9177 | 100.948 |
| lncRNA18306 | "x" | chr04:4722699-4724263 | 0.602975 | 0.392278 |
| lncRNA18330 | "x" | chr04:5087203-5090283 | 1.75215 | 1.51003 |
| lncRNA08281 | "x" | chr02:41311122-41321957 | 1.31507 | 1.90526 |
| lncRNA09094 | "x" | chr02:49748275-49765101 | 0.908729 | 1.28741 |
| lncRNA38146 | "x" | chr10:46856998-46859735 | 1.0991 | 1.57767 |
| lncRNA15424 | "x" | chr03:58105200-58107482 | 0.48169 | 0.7826 |
| lncRNA25277 | "x" | chr06:1716213-1719659 | 22.4239 | 27.615 |
| lncRNA08891 | "u" | chr02:47695980-47696298 | 27.8955 | 21.9432 |
| lncRNA32637 | "x" | chr08:22866364-22867852 | 18.566 | 21.5588 |
| lncRNA06347 | "x" | chr01:85211482-85216375 | 0.93903 | 0.607189 |
| lncRNA35153 | "x" | chr09:62775940-62776597 | 1.49208 | 1.93477 |
| lncRNA23220 | "x" | chr06:692090-695354 | 0.502038 | 0.727794 |
| lncRNA11695 | "x" | chr03:5669211-5673369 | 2.59478 | 3.12517 |
| lncRNA06179 | "x" | chr01:83160061-83163345 | 1.34503 | 1.16064 |
| lncRNA33829 | "x" | chr08:62958392-62969194 | 33.5365 | 25.4948 |
| lncRNA36124 | "x" | chr09:14886227-14887371 | 4.03547 | 3.4079 |
| lncRNA14804 | "x" | chr03:46351732-46357225 | 10.9453 | 15.2819 |
| lncRNA25997 | "x" | chr06:33808185-33811464 | 8.42145 | 6.48559 |
| lncRNA20324 | "x" | chr05:5787613-5790355 | 1.01243 | 0.716528 |
| lncRNA16258 | "x" | chr04:2579018-2581365 | 2.54944 | 4.39782 |
| lncRNA43800 | "x" | chr11:30479838-30483491 | 1.74057 | 1.48569 |
| lncRNA18277 | "u" | chr04:4431311-4435936 | 1.54799 | 1.35745 |
| lncRNA43422 | "x" | chr11:6653733-6662471 | 1.45922 | 0.951041 |
| lncRNA48340 | "x" | chr12:65050824-65051571 | 70.2219 | 80.6881 |
| lncRNA10759 | "x" | chr02:42565244-42567726 | 0.780032 | 1.15449 |
| lncRNA25046 | "x" | chr06:44949968-44952757 | 2.57674 | 3.2423 |
| lncRNA48337 | "x" | chr12:65030398-65035155 | 0.902313 | 1.52302 |
| lncRNA08441 | "x" | chr02:43010118-43017774 | 0.641661 | 0.428425 |
| lncRNA40583 | "x" | chr10:61511727-61512437 | 33.6834 | 41.8581 |
| lncRNA41786 | "x" | chr11:13596865-13599221 | 0.493207 | 0.867639 |
| lncRNA44205 | "x" | chr11:48939579-48940478 | 3.15819 | 3.85758 |
| lncRNA42082 | "x" | chr11:35949958-35955757 | 9.61141 | 11.578 |
| lncRNA15301 | "x" | chr03:56690057-56697829 | 5.32846 | 8.17226 |
| lncRNA25729 | "u" | chr06:27914869-27918479 | 8.83866 | 9.8224 |
| lncRNA00005 | "u" | chr00:1143572-1150355 | 3.78159 | 4.20032 |
| lncRNA42430 | "u" | chr11:49289933-49293239 | 0.689325 | 0.579605 |
| lncRNA11734 | "x" | chr03:6977536-6983383 | 6.15596 | 4.53012 |
| lncRNA37262 | "x" | chr09:67569665-67573987 | 5.72675 | 7.74839 |
| lncRNA26631 | "x" | chr06:41474234-41474903 | 5.89073 | 10.2653 |
| lncRNA41406 | "x" | chr11:4562896-4563457 | 2.60016 | 1.91873 |
| lncRNA36273 | "x" | chr09:30362984-30369236 | 0.595957 | 0.452122 |
| lncRNA06142 | "x" | chr01:82756555-82760521 | 1.83683 | 2.27242 |
| lncRNA45789 | "x" | chr12:44819812-44820619 | 8.93636 | 13.9161 |
| lncRNA02208 | "x" | chr01:68969861-68975207 | 1.14465 | 1.32587 |
| lncRNA28344 | "x" | chr07:60947078-60947975 | 13.5495 | 19.4491 |
| lncRNA02898 | "x" | chr01:78775058-78787916 | 7.82756 | 5.36402 |
| lncRNA05327 | "x" | chr01:72868816-72892972 | 2.10392 | 1.19977 |
| lncRNA43383 | "x" | chr11:5954869-5955636 | 39.0922 | 33.7635 |
| lncRNA05878 | "x" | chr01:79961872-79966303 | 0.518741 | 0.803119 |
| lncRNA38317 | "u" | chr10:52638041-52640614 | 2.03511 | 2.3398 |
| lncRNA46698 | "x" | chr12:1846726-1858737 | 4.4572 | 3.60202 |
| lncRNA02226 | "u" | chr01:69301207-69302971 | 1.83613 | 1.55919 |
| lncRNA05090 | "x" | chr01:68053131-68064366 | 8.2726 | 5.38343 |
| lncRNA10810 | "x" | chr02:42964848-42978097 | 1.38792 | 1.0108 |
| lncRNA35238 | "x" | chr09:64188415-64193786 | 1.26685 | 0.914333 |
| lncRNA36808 | "x" | chr09:61752746-61755104 | 3.54248 | 4.15928 |
| lncRNA24328 | "x" | chr06:37502784-37506620 | 7.56078 | 5.01005 |
| lncRNA35453 | "x" | chr09:66610988-66615970 | 1.79933 | 1.46534 |
| lncRNA42387 | "x" | chr11:48597971-48610372 | 1.85049 | 1.18414 |
| lncRNA13412 | "u" | chr03:61789245-61790066 | 13.0998 | 14.8637 |
| lncRNA30153 | "x" | chr07:62119222-62123881 | 2.75657 | 3.70459 |
| lncRNA27966 | "x" | chr07:55156872-55162253 | 1.89298 | 1.23354 |
| lncRNA45323 | "x" | chr12:11304998-11311216 | 1.80038 | 2.54182 |
| lncRNA43933 | "x" | chr11:36943234-36953509 | 0.981678 | 0.708978 |
| lncRNA16790 | "x" | chr04:30678741-30683513 | 0.725847 | 0.549902 |
| lncRNA15995 | "x" | chr03:64085002-64094618 | 34.1862 | 27.684 |
| lncRNA09709 | "x" | chr02:30427050-30432713 | 0.710769 | 0.919856 |
| lncRNA13172 | "x" | chr03:59287232-59290008 | 1.50313 | 1.18505 |
| lncRNA16019 | "x" | chr03:64358103-64359366 | 0.814735 | 1.45176 |
| lncRNA26669 | "x" | chr06:41997666-42009879 | 3.67719 | 5.44313 |
| lncRNA46163 | "x" | chr12:62489989-62494109 | 0.475803 | 0.772815 |
| lncRNA27711 | "x" | chr07:41645114-41647181 | 1.36768 | 2.36474 |
| lncRNA19606 | "x" | chr04:60978512-60983322 | 1.06854 | 1.56195 |
| lncRNA23271 | "x" | chr06:1320759-1327928 | 1.08615 | 1.2721 |
| lncRNA24409 | "x" | chr06:38517438-38521284 | 1.09393 | 0.778041 |
| lncRNA40600 | "x" | chr10:61621968-61626173 | 2.83222 | 1.63451 |
| lncRNA42259 | "x" | chr11:46309560-46311263 | 8.40204 | 6.5411 |
| lncRNA45391 | "x" | chr12:20128895-20139142 | 3.63454 | 3.24657 |
| lncRNA24530 | "x" | chr06:39792730-39798747 | 19.295 | 26.0365 |
| lncRNA41097 | "x" | chr11:1161597-1163447 | 44.3637 | 34.4614 |
| lncRNA17959 | "u" | chr04:369311-369627 | 29.3809 | 23.4758 |
| lncRNA26459 | "u" | chr06:39699660-39700020 | 14.1339 | 11.283 |
| lncRNA14122 | "u" | chr03:9141982-9145954 | 1.06711 | 1.23077 |
| lncRNA47775 | "u" | chr12:47153149-47154162 | 0.867585 | 0.653989 |
| lncRNA46189 | "x" | chr12:62736018-62742836 | 8.72917 | 11.2674 |
| lncRNA28955 | "u" | chr07:2404900-2405840 | 2.36813 | 1.93179 |
| lncRNA25908 | "u" | chr06:32479011-32479694 | 1.69361 | 1.29157 |
| lncRNA34478 | "u" | chr09:18309236-18309884 | 3.50435 | 2.80642 |
| lncRNA40980 | "x" | chr11:45035-49724 | 9.0531 | 7.14409 |
| lncRNA23815 | "u" | chr06:29255067-29255815 | 1.13194 | 1.48018 |
| lncRNA18775 | "x" | chr04:37836462-37848150 | 1.25814 | 0.924535 |
| lncRNA46776 | "x" | chr12:2671715-2673972 | 2.16568 | 2.5271 |
| lncRNA21346 | "x" | chr05:62901009-62908425 | 10.1088 | 12.8702 |
| lncRNA23451 | "u" | chr06:4553716-4558176 | 9.582 | 10.7919 |
| lncRNA48225 | "x" | chr12:64110248-64114714 | 108.741 | 150.401 |
| lncRNA39006 | "x" | chr10:64091670-64094291 | 4.33558 | 5.00904 |
| lncRNA43081 | "x" | chr11:2495402-2498527 | 4.41037 | 5.19246 |
| lncRNA15100 | "x" | chr03:53122338-53128281 | 3.51326 | 2.84262 |
| lncRNA28646 | "x" | chr07:64179981-64186849 | 1.16383 | 1.76039 |
| lncRNA25920 | "x" | chr06:32754800-32755810 | 4.8791 | 5.80953 |
| lncRNA40481 | "x" | chr10:60536605-60540360 | 0.954064 | 0.530977 |
| lncRNA38765 | "x" | chr10:61851226-61858478 | 34.4106 | 58.517 |
| lncRNA23182 | "x" | chr06:23848-26003 | 2.29985 | 1.50017 |
| lncRNA16958 | "x" | chr04:48422729-48425767 | 5.628 | 7.64797 |
| lncRNA41123 | "x" | chr11:1424638-1425782 | 1.91004 | 2.3575 |
| lncRNA45156 | "x" | chr12:5069062-5074778 | 161.789 | 118.073 |
| lncRNA46487 | "x" | chr12:65284474-65285353 | 5.85666 | 8.0053 |
| lncRNA35023 | "o" | chr09:60861307-60879115 | 2.93392 | 5.67243 |
| lncRNA16890 | "x" | chr04:42201866-42210091 | 3.68895 | 4.28934 |
| lncRNA22015 | "x" | chr05:6766536-6771486 | 7.45351 | 6.38901 |
| lncRNA25015 | "x" | chr06:44670440-44671383 | 30.61 | 22.7925 |
| lncRNA28753 | "x" | chr07:65153195-65157585 | 3.18293 | 4.1339 |
| lncRNA17730 | "x" | chr04:61932458-61945517 | 1.99679 | 1.43314 |
| lncRNA46295 | "x" | chr12:63740294-63748742 | 1.7906 | 1.34633 |
| lncRNA27085 | "x" | chr07:288359-289609 | 1.39302 | 0.901181 |
| lncRNA26785 | "x" | chr06:43294893-43300576 | 10.7119 | 14.3521 |
| lncRNA33461 | "x" | chr08:58397566-58400828 | 1.11321 | 1.37107 |
| lncRNA07401 | "x" | chr02:30847020-30852710 | 10.2319 | 13.2613 |
| lncRNA40695 | "x" | chr10:62426454-62429694 | 7.97133 | 13.871 |
| lncRNA44112 | "x" | chr11:47229605-47242121 | 1.7977 | 2.56332 |
| lncRNA43643 | "u" | chr11:16713611-16715112 | 3.11141 | 2.68644 |
| lncRNA41092 | "x" | chr11:1127407-1134287 | 6.82101 | 8.3287 |
| lncRNA47291 | "x" | chr12:23288024-23292305 | 1.30848 | 1.85411 |
| lncRNA33850 | "x" | chr09:156252-158894 | 1.22638 | 1.59611 |
| lncRNA34979 | "x" | chr09:59863133-59863944 | 3.44637 | 2.2525 |
| lncRNA39955 | "u" | chr10:45933549-45933996 | 19.9316 | 23.2249 |
| lncRNA42535 | "x" | chr11:50446088-50451004 | 8.04217 | 10.5614 |
| lncRNA09702 | "x" | chr02:30328161-30329074 | 34.1131 | 40.8769 |
| lncRNA45493 | "u" | chr12:30710997-30711605 | 1.23464 | 0.916481 |
| lncRNA35147 | "x" | chr09:62718582-62723215 | 1.0554 | 1.24332 |
| lncRNA31975 | "x" | chr08:61360541-61364423 | 33.1941 | 23.3611 |
| lncRNA45728 | "x" | chr12:43448467-43449792 | 4.34055 | 6.28555 |
| lncRNA13523 | "x" | chr03:63010743-63013302 | 1.31461 | 1.04701 |
| lncRNA06263 | "x" | chr01:84215759-84219412 | 1.23865 | 1.76721 |
| lncRNA43189 | "x" | chr11:3691034-3704230 | 19.5281 | 24.7136 |
| lncRNA36551 | "x" | chr09:55885028-55889505 | 3.2408 | 4.85405 |
| lncRNA02325 | "x" | chr01:70996669-71002457 | 0.958144 | 0.532127 |
| lncRNA10583 | "x" | chr02:40814596-40816158 | 8.59413 | 12.3372 |
| lncRNA20414 | "x" | chr05:7410726-7417285 | 27.3712 | 31.9804 |
| lncRNA11720 | "x" | chr03:6806298-6809434 | 2.62363 | 2.06446 |
| lncRNA16562 | "x" | chr04:7355671-7363750 | 7.24649 | 10.1288 |
| lncRNA40533 | "u" | chr10:60999359-61002644 | 13.699 | 12.3977 |
| lncRNA41827 | "x" | chr11:15586200-15590361 | 0.678114 | 0.834134 |
| lncRNA33828 | "x" | chr08:62958392-62969194 | 14.4551 | 11.0609 |
| lncRNA03953 | "x" | chr01:90039525-90058786 | 1.61744 | 2.03547 |
| lncRNA16889 | "u" | chr04:42024678-42025045 | 6.77306 | 8.81538 |
| lncRNA32251 | "x" | chr08:1257743-1264946 | 17.5293 | 13.3522 |
| lncRNA39182 | "x" | chr10:1096396-1098696 | 8.61723 | 9.96413 |
| lncRNA41763 | "u" | chr11:13116298-13118782 | 1.03311 | 0.877385 |
| lncRNA44250 | "x" | chr11:49620492-49626032 | 1.24988 | 1.67823 |
| lncRNA14807 | "x" | chr03:46382483-46389077 | 11.1494 | 9.6512 |
| lncRNA25436 | "u" | chr06:4831270-4834771 | 1.42157 | 1.2355 |
| lncRNA31041 | "x" | chr08:38233772-38235053 | 2.25713 | 2.67415 |
| lncRNA19273 | "x" | chr04:56665496-56679263 | 4.32956 | 3.53421 |
| lncRNA28062 | "x" | chr07:56838601-56840961 | 43.3123 | 36.8532 |
| lncRNA11408 | "x" | chr02:49121178-49125517 | 6.29156 | 4.09648 |
| lncRNA43357 | "x" | chr11:5572668-5592438 | 0.568358 | 0.832136 |
| lncRNA46235 | "x" | chr12:63245145-63248345 | 7.33804 | 8.93661 |
| lncRNA14144 | "x" | chr03:9344332-9346934 | 2.19041 | 3.04161 |
| lncRNA24725 | "x" | chr06:41622095-41629236 | 0.845391 | 0.618141 |
| lncRNA28463 | "j" | chr07:62379497-62387295 | 0.520749 | 1.08675 |
| lncRNA36092 | "x" | chr09:11864123-11869655 | 9.14936 | 8.3125 |
| lncRNA09797 | "u" | chr02:31916918-31919476 | 3.84644 | 3.4057 |
| lncRNA17367 | "x" | chr04:57370861-57376283 | 10.5093 | 9.08856 |
| lncRNA23510 | "u" | chr06:9557312-9557994 | 17.1261 | 15.0488 |
| lncRNA38755 | "x" | chr10:61751884-61754621 | 3.36963 | 4.31096 |
| lncRNA07773 | "u" | chr02:35772879-35774799 | 0.82144 | 0.987138 |
| lncRNA13461 | "x" | chr03:62298628-62303181 | 2.1635 | 2.90412 |
| lncRNA24623 | "x" | chr06:40682292-40686760 | 2.75742 | 3.70611 |
| lncRNA27450 | "x" | chr07:7942966-7945924 | 32.2066 | 26.2294 |
| lncRNA13694 | "x" | chr03:64802904-64811982 | 1.21916 | 0.942326 |
| lncRNA14819 | "x" | chr03:46522037-46533607 | 0.602621 | 0.877969 |
| lncRNA25977 | "x" | chr06:33626365-33627142 | 11.5784 | 16.0444 |
| lncRNA36668 | "x" | chr09:59091344-59094313 | 33.1505 | 29.7083 |
| lncRNA23012 | "x" | chr05:63294452-63299184 | 0.714792 | 1.02081 |
| lncRNA33587 | "x" | chr08:60067528-60071933 | 2.50811 | 3.1539 |
| lncRNA21757 | "x" | chr05:3023477-3033476 | 1.75497 | 1.39199 |
| lncRNA34192 | "x" | chr09:4199566-4209478 | 16.079 | 21.3771 |
| lncRNA21006 | "u" | chr05:56280111-56280919 | 4.29423 | 3.63761 |
| lncRNA33941 | "x" | chr09:978550-981575 | 0.801569 | 1.04099 |
| lncRNA02768 | "x" | chr01:77372506-77374935 | 1.93907 | 2.60976 |
| lncRNA40266 | "x" | chr10:58005212-58008669 | 2.03425 | 2.5875 |
| lncRNA40536 | "x" | chr10:61024274-61027540 | 28.4267 | 22.5322 |
| lncRNA13853 | "x" | chr03:2052866-2056148 | 2.5921 | 3.17737 |
| lncRNA10821 | "u" | chr02:43124094-43124686 | 13.5599 | 11.7179 |
| lncRNA46534 | "x" | chr12:147173-153831 | 5.36713 | 3.80065 |
| lncRNA47413 | "x" | chr12:33685763-33688873 | 5.80883 | 9.05839 |
| lncRNA26358 | "x" | chr06:38545386-38550453 | 4.11633 | 3.27562 |
| lncRNA39008 | "u" | chr10:64095402-64095866 | 13.4054 | 11.4099 |
| lncRNA47136 | "x" | chr12:9071678-9076697 | 39.6673 | 48.3733 |
| lncRNA07293 | "x" | chr02:28534145-28538817 | 2.4 | 1.69295 |
| lncRNA24244 | "x" | chr06:36399869-36402486 | 7.06274 | 7.97306 |
| lncRNA21987 | "x" | chr05:6329769-6332005 | 5.14413 | 3.95922 |
| lncRNA46060 | "x" | chr12:55873691-55882235 | 3.57536 | 2.95248 |
| lncRNA14334 | "u" | chr03:15573434-15573989 | 3.66874 | 4.5196 |
| lncRNA19744 | "x" | chr04:62372756-62376875 | 2.26666 | 1.70632 |
| lncRNA16959 | "x" | chr04:48422729-48425767 | 1.57741 | 1.25678 |
| lncRNA28403 | "x" | chr07:61634118-61644861 | 6.85061 | 9.22986 |
| lncRNA07569 | "x" | chr02:33182050-33183581 | 2.7902 | 3.50704 |
| lncRNA03379 | "x" | chr01:84055797-84057975 | 0.773547 | 0.59862 |
| lncRNA27615 | "x" | chr07:26687213-26688625 | 8.86998 | 6.72395 |
| lncRNA35626 | "x" | chr09:605625-607732 | 0.610603 | 0.528938 |
| lncRNA19783 | "u" | chr04:62872882-62873100 | 366.128 | 296.599 |
| lncRNA28767 | "x" | chr07:134732-140291 | 2.69713 | 3.22066 |
| lncRNA26878 | "x" | chr06:44298356-44301354 | 2.66489 | 3.85422 |
| lncRNA22051 | "x" | chr05:7472079-7476868 | 4.96928 | 3.82916 |
| lncRNA19849 | "x" | chr04:63530073-63539032 | 0.776682 | 0.522629 |
| lncRNA25898 | "x" | chr06:32405536-32407910 | 1.00106 | 0.768662 |
| lncRNA15728 | "x" | chr03:61224741-61239563 | 0.800717 | 0.519318 |
| lncRNA01771 | "x" | chr01:36603557-36621602 | 8.62372 | 12.238 |
| lncRNA36631 | "x" | chr09:57990444-57994976 | 3.49075 | 2.66663 |
| lncRNA19452 | "x" | chr04:58909754-58914890 | 0.750595 | 0.448658 |
| lncRNA25095 | "x" | chr06:45588704-45595285 | 2.11796 | 3.19733 |
| lncRNA40590 | "x" | chr10:61566360-61568335 | 4.9358 | 6.73673 |
| lncRNA42571 | "u" | chr11:50810461-50814021 | 1.09881 | 0.954908 |
| lncRNA37244 | "x" | chr09:67317557-67321196 | 0.686052 | 0.951499 |
| lncRNA11380 | "x" | chr02:48893525-48896917 | 1.01126 | 0.666621 |
| lncRNA31623 | "x" | chr08:57133148-57134844 | 0.901928 | 1.24971 |
| lncRNA18382 | "u" | chr04:5884809-5888621 | 4.61043 | 5.10905 |
| lncRNA20695 | "x" | chr05:28060780-28074769 | 622.313 | 328.512 |
| lncRNA38798 | "x" | chr10:62138486-62141090 | 1.11224 | 0.904915 |
| lncRNA17254 | "x" | chr04:55617931-55632414 | 1.02064 | 0.597422 |
| lncRNA38009 | "x" | chr10:38708745-38714191 | 3.1037 | 2.56653 |
| lncRNA47879 | "x" | chr12:49627021-49631589 | 0.844442 | 0.514395 |
| lncRNA15274 | "x" | chr03:56424948-56427685 | 0.691064 | 1.09543 |
| lncRNA15505 | "x" | chr03:58949925-58952574 | 4.93795 | 6.77813 |
| lncRNA19918 | "x" | chr05:107756-110824 | 1.70601 | 1.19577 |
| lncRNA40329 | "x" | chr10:59189547-59194597 | 4.92526 | 3.53837 |
| lncRNA36621 | "x" | chr09:57800520-57806263 | 3.16924 | 2.33368 |
| lncRNA11062 | "x" | chr02:45506184-45507431 | 10.294 | 7.66747 |
| lncRNA25234 | "x" | chr06:1304941-1310377 | 1.02709 | 1.6403 |
| lncRNA25640 | "x" | chr06:22263030-22264655 | 0.643016 | 0.475529 |
| lncRNA28544 | "x" | chr07:63212971-63221060 | 0.902783 | 1.33315 |
| lncRNA17255 | "x" | chr04:55646757-55648252 | 12.7937 | 9.69262 |
| lncRNA09660 | "u" | chr02:29711092-29711901 | 1.32976 | 1.06217 |
| lncRNA10568 | "u" | chr02:40575323-40576445 | 0.839673 | 0.669672 |
| lncRNA16538 | "x" | chr04:6723020-6728721 | 0.748673 | 1.05487 |
| lncRNA26518 | "x" | chr06:40443789-40450397 | 1.70186 | 2.05892 |
| lncRNA20584 | "u" | chr05:19173690-19176245 | 4.4846 | 3.99937 |
| lncRNA28947 | "x" | chr07:2356041-2360542 | 20.3677 | 14.4594 |
| lncRNA00995 | "x" | chr00:18727879-18730776 | 3.51525 | 2.27857 |
| lncRNA23234 | "u" | chr06:828017-829282 | 3.9758 | 4.55981 |
| lncRNA46429 | "x" | chr12:64861258-64863316 | 3.47281 | 3.07051 |
| lncRNA28476 | "x" | chr07:62463023-62467530 | 14.9235 | 13.0344 |
| lncRNA03936 | "x" | chr01:89817467-89825251 | 4.16675 | 3.36178 |
| lncRNA14824 | "x" | chr03:46577091-46584432 | 1.60903 | 1.37736 |
| lncRNA26282 | "x" | chr06:37721325-37723464 | 7.21341 | 12.9498 |
| lncRNA42739 | "x" | chr11:52550280-52553441 | 5.29713 | 4.2135 |
| lncRNA22848 | "x" | chr05:61145682-61160111 | 1.03632 | 0.62645 |
| lncRNA18289 | "x" | chr04:4541496-4544361 | 14.2148 | 9.99026 |
| lncRNA14735 | "x" | chr03:45462356-45464256 | 7.11753 | 6.22686 |
| lncRNA34540 | "x" | chr09:24061586-24062513 | 3.96707 | 5.58494 |
| lncRNA46839 | "x" | chr12:3340221-3341446 | 6.57372 | 7.81494 |
| lncRNA20335 | "u" | chr05:5840871-5851353 | 1.40256 | 1.03918 |
| lncRNA47524 | "x" | chr12:39219487-39221955 | 5.9879 | 4.13425 |
| lncRNA08442 | "u" | chr02:43018232-43020330 | 1.0425 | 0.879865 |
| lncRNA17224 | "u" | chr04:54990374-54991034 | 2.76331 | 3.39977 |
| lncRNA44450 | "x" | chr11:51745188-51751207 | 2.7442 | 3.55816 |
| lncRNA14900 | "x" | chr03:48166951-48178089 | 0.647966 | 0.838402 |
| lncRNA11262 | "x" | chr02:47576841-47584344 | 2.02048 | 2.87285 |
| lncRNA15139 | "x" | chr03:53624544-53628355 | 1.22378 | 0.908584 |
| lncRNA28153 | "x" | chr07:58387485-58389956 | 8.39948 | 10.8401 |
| lncRNA43926 | "x" | chr11:36633139-36638389 | 1.44434 | 1.17487 |
| lncRNA10507 | "x" | chr02:39870548-39873842 | 4.40069 | 5.44396 |
| lncRNA30219 | "x" | chr07:62742398-62770824 | 10.078 | 12.0393 |
| lncRNA46250 | "j" | chr12:63400153-63405993 | 81.6006 | 93.4811 |
| lncRNA33325 | "x" | chr08:56785410-56792428 | 0.815231 | 0.622455 |
| lncRNA15019 | "x" | chr03:51376490-51382146 | 6.59122 | 5.12865 |
| lncRNA39726 | "x" | chr10:28164989-28167199 | 2.42095 | 2.05928 |
| lncRNA45111 | "x" | chr12:4588155-4591740 | 3.32101 | 2.32367 |
| lncRNA31596 | "x" | chr08:56823208-56825538 | 4.79801 | 3.85641 |
| lncRNA30134 | "x" | chr07:61921992-61925034 | 8.54995 | 6.92632 |
| lncRNA31523 | "x" | chr08:55666614-55667282 | 1.32904 | 0.867802 |
| lncRNA45615 | "u" | chr12:37670839-37688024 | 0.568703 | 1.11561 |
| lncRNA47171 | "u" | chr12:10619412-10622877 | 2.87914 | 3.41746 |
| lncRNA20269 | "x" | chr05:4905125-4906026 | 5.13483 | 4.42219 |
| lncRNA46247 | "x" | chr12:63354422-63364478 | 5.37633 | 12.0022 |
| lncRNA17396 | "x" | chr04:57830786-57838718 | 0.774202 | 1.22267 |
| lncRNA37550 | "x" | chr10:3225901-3226629 | 5.76443 | 9.39457 |
| lncRNA10529 | "x" | chr02:40107111-40113981 | 1.44091 | 1.16969 |
| lncRNA27298 | "x" | chr07:3386407-3389659 | 2.92082 | 3.39037 |
| lncRNA39847 | "x" | chr10:40070147-40074680 | 2.85751 | 2.1651 |
| lncRNA45819 | "x" | chr12:45713473-45716680 | 2.298 | 1.86123 |
| lncRNA17042 | "u" | chr04:50863122-50863765 | 24.8043 | 22.1366 |
| lncRNA31403 | "x" | chr08:53623133-53624739 | 52.4237 | 40.8469 |
| lncRNA44967 | "x" | chr12:3054836-3056142 | 24.9692 | 21.556 |
| lncRNA01188 | "x" | chr01:1430745-1438943 | 1.30073 | 0.802214 |
| lncRNA31212 | "x" | chr08:48833026-48840941 | 1.92342 | 1.4051 |
| lncRNA34453 | "x" | chr09:16842843-16844969 | 8.76573 | 6.49765 |
| lncRNA04023 | "x" | chr01:663323-669843 | 2.23512 | 1.90108 |
| lncRNA04698 | "x" | chr01:37546039-37550895 | 3.43008 | 3.06685 |
| lncRNA23817 | "x" | chr06:29256146-29260955 | 1.03663 | 1.5603 |
| lncRNA44940 | "x" | chr12:2837632-2843798 | 2.29287 | 2.9278 |
| lncRNA13643 | "x" | chr03:64287120-64295654 | 7.67843 | 5.77946 |
| lncRNA42308 | "x" | chr11:47345790-47352691 | 3.40083 | 4.89151 |
| lncRNA46470 | "x" | chr12:65154940-65160628 | 11.126 | 14.8793 |
| lncRNA09263 | "x" | chr02:13199688-13206298 | 1.18671 | 1.02748 |
| lncRNA30255 | "x" | chr07:63154191-63158875 | 2.32219 | 2.93328 |
| lncRNA11854 | "x" | chr03:8851151-8851914 | 11.564 | 7.49579 |
| lncRNA42437 | "x" | chr11:49371321-49397877 | 16.193 | 32.1205 |
| lncRNA09835 | "x" | chr02:32391617-32393160 | 2.77561 | 3.58815 |
| lncRNA10779 | "x" | chr02:42758701-42759696 | 3.91869 | 2.54013 |
| lncRNA12603 | "x" | chr03:48603036-48605620 | 1.27324 | 1.65743 |
| lncRNA37899 | "x" | chr10:28260427-28267239 | 1.42449 | 1.13216 |
| lncRNA38683 | "x" | chr10:61087316-61091045 | 57.3136 | 46.7228 |
| lncRNA11095 | "x" | chr02:45888677-45903696 | 0.696567 | 1.03118 |
| lncRNA20632 | "x" | chr05:22505559-22507232 | 20.482 | 23.314 |
| lncRNA33948 | "x" | chr09:1074959-1077700 | 3.59114 | 3.10182 |
| lncRNA38256 | "u" | chr10:51220183-51220780 | 4.79178 | 5.73785 |
| lncRNA03460 | "x" | chr01:84820986-84826024 | 2.02907 | 2.54961 |
| lncRNA38371 | "x" | chr10:57429081-57430448 | 91.3245 | 67.6141 |
| lncRNA42605 | "x" | chr11:51207833-51211668 | 3.98331 | 2.9742 |
| lncRNA45746 | "x" | chr12:43796572-43805138 | 1.52509 | 2.34859 |
| lncRNA24439 | "x" | chr06:38767649-38769674 | 5.14792 | 6.45501 |
| lncRNA39686 | "x" | chr10:22672166-22682547 | 18.9755 | 22.4795 |
| lncRNA11460 | "u" | chr02:49721740-49721994 | 97.0311 | 119.288 |
| lncRNA23287 | "x" | chr06:1625241-1627604 | 7.739 | 9.40505 |
| lncRNA27174 | "x" | chr07:1547766-1557882 | 1.60009 | 1.21711 |
| lncRNA13258 | "x" | chr03:60253149-60260762 | 1.7935 | 2.32278 |
| lncRNA34994 | "x" | chr09:60238524-60243325 | 0.697512 | 0.517473 |
| lncRNA46752 | "x" | chr12:2409946-2413184 | 4.99498 | 5.70891 |
| lncRNA42994 | "x" | chr11:1576540-1581335 | 9.39109 | 7.1173 |
| lncRNA11382 | "x" | chr02:48901700-48907222 | 1.26609 | 0.920459 |
| lncRNA17174 | "u" | chr04:54316307-54316722 | 4.85126 | 3.76694 |
| lncRNA19066 | "u" | chr04:52882732-52884886 | 1.45995 | 1.13456 |
| lncRNA13197 | "x" | chr03:59555343-59556136 | 4.96723 | 8.11296 |
| lncRNA07527 | "x" | chr02:32627789-32637710 | 3.43993 | 4.45564 |
| lncRNA27471 | "x" | chr07:8937608-8941322 | 0.746502 | 0.578029 |
| lncRNA29847 | "x" | chr07:58467381-58471454 | 8.24188 | 6.17342 |
| lncRNA42268 | "u" | chr11:46526457-46526754 | 73.0842 | 61.838 |
| lncRNA05118 | "j" | chr01:68969861-68975207 | 0.856145 | 1.09542 |
| lncRNA15665 | "x" | chr03:60584573-60591595 | 0.932692 | 1.09832 |
| lncRNA47788 | "x" | chr12:47336795-47341380 | 2.4381 | 2.03835 |
| lncRNA45961 | "x" | chr12:48277686-48280333 | 5.615 | 4.64518 |
| lncRNA44644 | "x" | chr12:112325-116138 | 19.4479 | 11.4573 |
| lncRNA05108 | "x" | chr01:68612560-68622957 | 26.8389 | 33.029 |
| lncRNA24989 | "x" | chr06:44446249-44448616 | 2.43345 | 1.95697 |
| lncRNA11124 | "x" | chr02:46272183-46276233 | 3.80205 | 2.96989 |
| lncRNA18322 | "x" | chr04:4993925-5000772 | 2.10795 | 2.63256 |
| lncRNA15405 | "x" | chr03:57864364-57867465 | 0.822547 | 1.01842 |
| lncRNA47004 | "x" | chr12:5146926-5148878 | 4.86736 | 5.97088 |
| lncRNA38457 | "x" | chr10:58956826-58963034 | 1.05567 | 0.871734 |
| lncRNA45529 | "x" | chr12:32764935-32770977 | 4.9081 | 5.82828 |
| lncRNA38573 | "x" | chr10:60093778-60097061 | 20.1258 | 24.7307 |
| lncRNA01807 | "x" | chr01:40900287-40910083 | 0.726648 | 0.591973 |
| lncRNA05461 | "u" | chr01:74653068-74653453 | 62.0635 | 70.0643 |
| lncRNA21005 | "x" | chr05:56224297-56227509 | 0.785121 | 0.907523 |
| lncRNA10364 | "x" | chr02:38290039-38292921 | 1.12409 | 1.61093 |
| lncRNA31070 | "x" | chr08:42439714-42446002 | 12.5009 | 11.1542 |
| lncRNA45753 | "u" | chr12:44041567-44042650 | 5.96883 | 5.24137 |
| lncRNA41540 | "x" | chr11:6173996-6179893 | 2.57992 | 3.27545 |
| lncRNA34932 | "u" | chr09:59187435-59188243 | 41.231 | 48.9301 |
| lncRNA29587 | "u" | chr07:53542514-53542950 | 10.0662 | 8.35914 |
| lncRNA47373 | "x" | chr12:30748577-30749675 | 0.67564 | 0.528117 |
| lncRNA12550 | "x" | chr03:47262080-47265159 | 1.63554 | 1.21321 |
| lncRNA15872 | "x" | chr03:62869677-62870805 | 14.9997 | 12.4003 |
| lncRNA30937 | "x" | chr08:23558694-23562046 | 0.616668 | 0.717558 |
| lncRNA12614 | "x" | chr03:48962368-48968719 | 6.15719 | 4.38762 |
| lncRNA47213 | "x" | chr12:12860664-12861539 | 8.93923 | 7.88529 |
| lncRNA22271 | "x" | chr05:19345632-19349812 | 8.20387 | 9.66117 |
| lncRNA40109 | "x" | chr10:51598678-51604016 | 2.74679 | 3.45935 |
| lncRNA11573 | "x" | chr03:1179805-1181174 | 1.09039 | 1.79481 |
| lncRNA07840 | "x" | chr02:36440999-36445829 | 1.98414 | 2.3451 |
| lncRNA41178 | "u" | chr11:1861455-1862025 | 10.0459 | 8.67427 |
| lncRNA34400 | "x" | chr09:12702259-12715748 | 0.644627 | 0.510263 |
| lncRNA21145 | "x" | chr05:60214145-60218827 | 2.35825 | 1.73582 |
| lncRNA44117 | "x" | chr11:47316104-47325352 | 19.083 | 15.3081 |
| lncRNA29826 | "x" | chr07:58104788-58107207 | 64.9354 | 57.7145 |
| lncRNA22832 | "x" | chr05:60759276-60763583 | 2.50269 | 2.16768 |
| lncRNA03513 | "x" | chr01:85403788-85409718 | 7.24528 | 8.06004 |
| lncRNA25013 | "x" | chr06:44653365-44654794 | 9.75213 | 13.1276 |
| lncRNA18354 | "u" | chr04:5348266-5349380 | 1.22704 | 1.4955 |
| lncRNA01482 | "u" | chr01:8695482-8695795 | 13.1811 | 17.2449 |
| lncRNA44246 | "x" | chr11:49591440-49596362 | 4.43532 | 5.60876 |
| lncRNA40848 | "x" | chr10:63775138-63780444 | 9.89499 | 12.4673 |
| lncRNA12569 | "u" | chr03:47795983-47796547 | 5.61065 | 4.69246 |
| lncRNA30330 | "x" | chr07:63951469-63952351 | 1.9045 | 2.37725 |
| lncRNA37266 | "u" | chr09:67590656-67591477 | 1.78007 | 2.16155 |
| lncRNA08193 | "u" | chr02:40409693-40409909 | 109.629 | 143.022 |
| lncRNA26834 | "x" | chr06:43828644-43831364 | 6.56472 | 5.05664 |
| lncRNA30789 | "u" | chr08:5900863-5901142 | 52.3019 | 42.0937 |
| lncRNA35619 | "x" | chr09:508766-511015 | 5.56122 | 6.64471 |
| lncRNA41163 | "x" | chr11:1746041-1746613 | 0.960232 | 1.51842 |
| lncRNA44732 | "u" | chr12:754878-755854 | 115.567 | 105.701 |
| lncRNA43029 | "x" | chr11:1850249-1853675 | 4.15805 | 3.43703 |
| lncRNA20647 | "x" | chr05:25812493-25813095 | 15.4956 | 19.9815 |
| lncRNA35808 | "x" | chr09:3063761-3071360 | 0.610442 | 0.398295 |
| lncRNA31603 | "x" | chr08:56903968-56905470 | 4.35876 | 3.02753 |
| lncRNA10384 | "x" | chr02:38540097-38546756 | 3.96638 | 3.04722 |
| lncRNA07550 | "x" | chr02:32844635-32846980 | 1.05799 | 0.856122 |
| lncRNA12214 | "x" | chr03:27437411-27441368 | 0.965268 | 1.25079 |
| lncRNA20511 | "x" | chr05:10767267-10772201 | 3.30368 | 2.53423 |
| lncRNA27385 | "u" | chr07:5234687-5234994 | 41.2809 | 49.1384 |
| lncRNA01101 | "x" | chr01:314641-317493 | 1.81057 | 2.34405 |
| lncRNA48289 | "x" | chr12:64654399-64657657 | 26.118 | 32.3488 |
| lncRNA08571 | "x" | chr02:44343131-44345022 | 0.505802 | 0.715259 |
| lncRNA46742 | "x" | chr12:2266945-2269806 | 1.35209 | 0.882513 |
| lncRNA24013 | "x" | chr06:33294346-33296510 | 5.77051 | 7.40764 |
| lncRNA14703 | "x" | chr03:44318707-44320001 | 8.94829 | 11.8488 |
| lncRNA32543 | "x" | chr08:10229036-10234421 | 12.5231 | 8.62807 |
| lncRNA06912 | "x" | chr02:7760648-7762916 | 1.03975 | 0.732459 |
| lncRNA26530 | "x" | chr06:40596759-40600442 | 0.558704 | 0.795523 |
| lncRNA25131 | "x" | chr06:45874012-45875152 | 2.24281 | 3.21361 |
| lncRNA20757 | "x" | chr05:31472117-31474741 | 6.90513 | 6.28621 |
| lncRNA38780 | "x" | chr10:61962634-61985280 | 31.2512 | 42.7928 |
| lncRNA41149 | "x" | chr11:1616197-1616804 | 1.85165 | 1.44629 |
| lncRNA22223 | "x" | chr05:16034730-16038070 | 8.00134 | 9.29322 |
| lncRNA26587 | "x" | chr06:41145492-41149587 | 1.82926 | 1.25092 |
| lncRNA30273 | "x" | chr07:63306051-63309625 | 24.5268 | 28.4049 |
| lncRNA05629 | "u" | chr01:76842017-76842738 | 0.917753 | 1.18791 |
| lncRNA19361 | "x" | chr04:57708363-57710287 | 2.25409 | 1.652 |
| lncRNA24378 | "u" | chr06:38093058-38093738 | 2.85479 | 2.34659 |
| lncRNA03463 | "u" | chr01:84854565-84855836 | 6.77232 | 7.59085 |
| lncRNA26019 | "x" | chr06:34298757-34314191 | 5.22398 | 6.78073 |
| lncRNA24823 | "x" | chr06:42712797-42713434 | 22.9132 | 14.8845 |
| lncRNA21744 | "u" | chr05:2884444-2885959 | 1.49079 | 1.74689 |
| lncRNA40440 | "x" | chr10:60089816-60093292 | 6.53795 | 5.68047 |
| lncRNA21384 | "x" | chr05:63405115-63408195 | 6.13612 | 5.32164 |
| lncRNA46469 | "x" | chr12:65149593-65154390 | 2.39945 | 1.97253 |
| lncRNA02239 | "x" | chr01:69474664-69480759 | 3.07771 | 2.50176 |
| lncRNA26855 | "x" | chr06:43991429-44000411 | 1.95031 | 2.773 |
| lncRNA08273 | "x" | chr02:41254138-41255995 | 4.58508 | 5.90317 |
| lncRNA43571 | "x" | chr11:12877256-12879053 | 1.33964 | 1.07166 |
| lncRNA21435 | "x" | chr05:64073845-64078030 | 2.62045 | 3.15727 |
| lncRNA26739 | "x" | chr06:42790701-42795143 | 3.31939 | 2.93624 |
| lncRNA27264 | "u" | chr07:2626762-2627652 | 5.66001 | 4.93389 |
| lncRNA40635 | "x" | chr10:61939030-61947948 | 3.08584 | 4.08052 |
| lncRNA36418 | "u" | chr09:46094439-46095408 | 1.28562 | 1.56148 |
| lncRNA42756 | "x" | chr11:52697579-52701602 | 15.2738 | 21.2897 |
| lncRNA06102 | "x" | chr01:82372616-82378174 | 3.01075 | 2.48282 |
| lncRNA23450 | "u" | chr06:4553716-4558176 | 1.65306 | 1.33456 |
| lncRNA41505 | "x" | chr11:5643478-5648033 | 2.63341 | 2.01427 |
| lncRNA47880 | "x" | chr12:49627021-49631589 | 3.87101 | 4.77741 |
| lncRNA15700 | "x" | chr03:60964197-60971730 | 5.32613 | 7.67348 |
| lncRNA44559 | "x" | chr11:52792684-52800589 | 11.3004 | 13.9733 |
| lncRNA16857 | "x" | chr04:38700444-38705951 | 3.69981 | 5.47095 |
| lncRNA06526 | "x" | chr01:87080460-87086437 | 0.923129 | 1.27732 |
| lncRNA33280 | "x" | chr08:56111005-56113627 | 3.87548 | 2.89919 |
| lncRNA34476 | "u" | chr09:18296700-18298102 | 1.10244 | 1.30705 |
| lncRNA33857 | "x" | chr09:183244-189258 | 5.19008 | 3.84491 |
| lncRNA07209 | "u" | chr02:22037318-22040281 | 5.28032 | 5.81383 |
| lncRNA28152 | "x" | chr07:58377402-58384583 | 0.757225 | 1.06955 |
| lncRNA03412 | "x" | chr01:84492006-84495850 | 3.03437 | 2.61268 |
| lncRNA33073 | "x" | chr08:52941304-52948844 | 10.6146 | 8.49297 |
| lncRNA34165 | "u" | chr09:3930561-3934919 | 1.3049 | 0.909157 |
| lncRNA26410 | "u" | chr06:39016762-39017477 | 5.06158 | 4.33351 |
| lncRNA28392 | "x" | chr07:61592601-61597151 | 1.71047 | 0.982091 |
| lncRNA18208 | "x" | chr04:3365036-3378905 | 6.73023 | 8.75515 |
| lncRNA30749 | "x" | chr08:3798490-3804724 | 0.59568 | 0.932768 |
| lncRNA25562 | "u" | chr06:20009303-20009653 | 29.5283 | 25.1513 |
| lncRNA19217 | "u" | chr04:55534046-55538041 | 1.37237 | 1.20852 |
| lncRNA40620 | "x" | chr10:61807054-61812997 | 2.22639 | 1.18356 |
| lncRNA35665 | "x" | chr09:1006614-1020453 | 6.2601 | 8.04441 |
| lncRNA43178 | "x" | chr11:3569231-3572080 | 6.80011 | 8.81789 |
| lncRNA36195 | "u" | chr09:21022259-21057350 | 0.617033 | 0.871812 |
| lncRNA05997 | "x" | chr01:81202876-81206378 | 4.88442 | 5.85161 |
| lncRNA20385 | "x" | chr05:6468235-6474651 | 6.46595 | 7.47668 |
| lncRNA37447 | "x" | chr10:2091467-2102853 | 18.7309 | 22.2311 |
| lncRNA25138 | "x" | chr06:45961421-45964444 | 6.06225 | 6.82219 |
| lncRNA41974 | "x" | chr11:28385874-28389979 | 5.925 | 8.16573 |
| lncRNA11277 | "x" | chr02:47869437-47873230 | 0.613145 | 0.791895 |
| lncRNA13214 | "x" | chr03:59803428-59807944 | 1.78768 | 1.60582 |
| lncRNA01153 | "x" | chr01:819249-827067 | 14.2087 | 12.4924 |
| lncRNA18239 | "u" | chr04:3930661-3935345 | 1.11126 | 1.25582 |
| lncRNA43378 | "x" | chr11:5917303-5932080 | 0.918009 | 0.679578 |
| lncRNA19778 | "x" | chr04:62774087-62778961 | 2.91042 | 2.36731 |
| lncRNA40492 | "x" | chr10:60644105-60649130 | 2.99785 | 2.60903 |
| lncRNA15951 | "x" | chr03:63621076-63623684 | 4.20656 | 3.02806 |
| lncRNA26453 | "x" | chr06:39591190-39595475 | 17.5085 | 23.3438 |
| lncRNA38145 | "x" | chr10:46845066-46847877 | 3.47055 | 4.01349 |
| lncRNA41337 | "x" | chr11:3704385-3706636 | 51.3806 | 41.702 |
| lncRNA11565 | "x" | chr03:1045287-1053451 | 10.317 | 12.4649 |
| lncRNA29159 | "x" | chr07:8737594-8770718 | 1.50905 | 0.985913 |
| lncRNA34060 | "x" | chr09:2443048-2448016 | 29.7899 | 24.2982 |
| lncRNA20035 | "x" | chr05:1607826-1612548 | 2.42118 | 1.94622 |
| lncRNA41546 | "x" | chr11:6331212-6333717 | 1.28224 | 1.519 |
| lncRNA47449 | "u" | chr12:35729923-35731911 | 0.650073 | 0.773867 |
| lncRNA28598 | "x" | chr07:63742297-63744672 | 1.7046 | 1.14412 |
| lncRNA29566 | "u" | chr07:52797346-52797757 | 51.3952 | 57.6393 |
| lncRNA27318 | "x" | chr07:3728617-3747433 | 2.19969 | 1.72382 |
| lncRNA35640 | "x" | chr09:707482-710234 | 1.37419 | 1.77721 |
| lncRNA15222 | "x" | chr03:55754957-55758771 | 2.49732 | 2.96128 |
| lncRNA48099 | "x" | chr12:63072437-63075882 | 1.12598 | 0.813319 |
| lncRNA09323 | "x" | chr02:16012191-16026741 | 5.56886 | 8.42418 |
| lncRNA05123 | "x" | chr01:69081972-69096361 | 3.49184 | 2.6265 |
| lncRNA24223 | "x" | chr06:36178409-36181754 | 0.969377 | 0.664315 |
| lncRNA33534 | "x" | chr08:59396340-59397387 | 2.70083 | 3.30523 |
| lncRNA06311 | "x" | chr01:84828310-84840249 | 0.772 | 0.544157 |
| lncRNA21313 | "x" | chr05:62504703-62510484 | 4.6679 | 3.62586 |
| lncRNA18558 | "x" | chr04:18238047-18251375 | 5.72106 | 3.93116 |
| lncRNA19009 | "u" | chr04:50914593-50916220 | 3.2418 | 2.86148 |
| lncRNA27494 | "u" | chr07:10507173-10508742 | 4.52811 | 4.03258 |
| lncRNA41716 | "x" | chr11:10694770-10702258 | 11.5032 | 10.2593 |
| lncRNA18189 | "x" | chr04:3163516-3167544 | 0.52594 | 0.646962 |
| lncRNA32744 | "x" | chr08:36316244-36320659 | 5.3773 | 6.92473 |
| lncRNA19791 | "u" | chr04:62951303-62951697 | 6.02115 | 4.68604 |
| lncRNA25156 | "x" | chr06:266780-271800 | 3.36905 | 2.81669 |
| lncRNA37008 | "x" | chr09:64287967-64292259 | 0.717553 | 1.30073 |
| lncRNA31077 | "u" | chr08:42819491-42820043 | 15.8614 | 13.9899 |
| lncRNA45909 | "x" | chr12:47483218-47492298 | 13.2736 | 10.4106 |
| lncRNA16835 | "u" | chr04:34400990-34403482 | 2.52267 | 2.25242 |
| lncRNA07938 | "u" | chr02:37604304-37605989 | 1.33505 | 1.54488 |
| lncRNA48372 | "x" | chr12:65253264-65258115 | 2.91572 | 2.18329 |
| lncRNA45720 | "x" | chr12:43228220-43230210 | 2.42409 | 3.80473 |
| lncRNA02645 | "x" | chr01:75450906-75457993 | 21.121 | 30.0229 |
| lncRNA11701 | "x" | chr03:6211092-6212137 | 17.2056 | 19.4177 |
| lncRNA44946 | "x" | chr12:2935791-2938988 | 7.85961 | 6.46598 |
| lncRNA39796 | "u" | chr10:35849443-35850538 | 5.34146 | 4.71984 |
| lncRNA30989 | "x" | chr08:29151559-29152760 | 3.94104 | 4.47613 |
| lncRNA12905 | "x" | chr03:56202186-56204866 | 9.01988 | 7.39844 |
| lncRNA19612 | "x" | chr04:61136135-61141411 | 0.90389 | 1.16607 |
| lncRNA07170 | "x" | chr02:21192405-21201016 | 0.4528 | 0.737375 |
| lncRNA26613 | "x" | chr06:41344912-41347546 | 3.50653 | 4.61495 |
| lncRNA18483 | "x" | chr04:9491988-9495123 | 5.25288 | 4.76056 |
| lncRNA36267 | "u" | chr09:29721303-29722226 | 7.40271 | 8.29911 |
| lncRNA41273 | "x" | chr11:2938050-2950401 | 12.363 | 16.3105 |
| lncRNA47724 | "x" | chr12:46080509-46092167 | 1.9019 | 1.41305 |
| lncRNA41476 | "x" | chr11:5339993-5340663 | 10.0839 | 7.97908 |
| lncRNA07436 | "u" | chr02:31325148-31331693 | 1.00027 | 0.646192 |
| lncRNA01755 | "x" | chr01:33896432-33906524 | 2.7307 | 3.50832 |
| lncRNA32279 | "x" | chr08:1603514-1605680 | 18.182 | 13.2336 |
| lncRNA10192 | "u" | chr02:36297466-36302033 | 19.2292 | 17.9868 |
| lncRNA39073 | "x" | chr10:64674860-64677321 | 26.5802 | 30.7464 |
| lncRNA05753 | "x" | chr01:78463592-78470070 | 0.978426 | 1.7349 |
| lncRNA16217 | "x" | chr04:2113279-2118375 | 1.43066 | 1.2554 |
| lncRNA40087 | "x" | chr10:51141760-51147365 | 65.8733 | 58.3516 |
| lncRNA40819 | "x" | chr10:63516370-63519557 | 4.3575 | 5.55077 |
| lncRNA12608 | "x" | chr03:48931859-48932684 | 15.8324 | 19.0427 |
| lncRNA26338 | "x" | chr06:38277654-38284362 | 33.377 | 24.606 |
| lncRNA11139 | "x" | chr02:46383041-46388070 | 0.487138 | 0.785474 |
| lncRNA40812 | "x" | chr10:63448011-63451661 | 7.59785 | 6.14381 |
| lncRNA41844 | "x" | chr11:16478163-16482708 | 3.33498 | 3.02612 |
| lncRNA06606 | "x" | chr01:87877827-87899995 | 6.62653 | 8.97892 |
| lncRNA17544 | "x" | chr04:59848287-59850129 | 5.02439 | 6.06895 |
| lncRNA06327 | "x" | chr01:84970756-84973065 | 0.853871 | 0.999206 |
| lncRNA08333 | "x" | chr02:41829797-41904084 | 4.90564 | 5.89501 |
| lncRNA47268 | "u" | chr12:19962141-19964047 | 1.63742 | 1.86974 |
| lncRNA10480 | "x" | chr02:39610382-39613685 | 3.41055 | 2.80537 |
| lncRNA17423 | "x" | chr04:58163010-58168188 | 2.92675 | 2.13049 |
| lncRNA47149 | "u" | chr12:9918833-9920053 | 3.50091 | 3.06483 |
| lncRNA25808 | "u" | chr06:30136521-30137207 | 11.4905 | 10.1707 |
| lncRNA19806 | "u" | chr04:63077812-63078983 | 1.36746 | 1.6198 |
| lncRNA22762 | "x" | chr05:59599094-59601524 | 18.0923 | 25.1066 |
| lncRNA34197 | "x" | chr09:4321586-4325542 | 1.29266 | 1.96488 |
| lncRNA30844 | "u" | chr08:9368950-9369993 | 0.958439 | 1.16646 |
| lncRNA31351 | "x" | chr08:52512629-52530812 | 8.83171 | 6.14875 |
| lncRNA29418 | "u" | chr07:40744465-40745873 | 1.75022 | 2.02473 |
| lncRNA28673 | "x" | chr07:64453634-64454971 | 25.5199 | 20.7383 |
| lncRNA43891 | "u" | chr11:35862327-35864196 | 4.50315 | 4.03967 |
| lncRNA27755 | "x" | chr07:46787807-46800500 | 2.37429 | 1.40101 |
| lncRNA30567 | "x" | chr08:1188324-1206669 | 2.92215 | 2.48411 |
| lncRNA35635 | "x" | chr09:677638-679595 | 1.85634 | 2.68903 |
| lncRNA46938 | "x" | chr12:4393941-4396972 | 3.03348 | 4.02825 |
| lncRNA17871 | "x" | chr04:63606781-63614167 | 12.3853 | 15.0042 |
| lncRNA31201 | "u" | chr08:48345970-48347163 | 1.12187 | 1.33104 |
| lncRNA41754 | "x" | chr11:12820897-12827341 | 2.74108 | 1.96796 |
| lncRNA36799 | "x" | chr09:61585602-61586182 | 25.833 | 36.4254 |
| lncRNA22186 | "x" | chr05:12223809-12238169 | 7.78746 | 5.22887 |
| lncRNA38797 | "x" | chr10:62138486-62141090 | 1.0037 | 1.19996 |
| lncRNA12167 | "x" | chr03:23398708-23400858 | 2.34054 | 2.62874 |
| lncRNA30202 | "x" | chr07:62593041-62595310 | 10.3632 | 7.99205 |
| lncRNA38970 | "x" | chr10:63850346-63854136 | 17.4192 | 22.5901 |
| lncRNA42543 | "x" | chr11:50509917-50513076 | 16.5522 | 18.0637 |
| lncRNA44128 | "x" | chr11:47420743-47430541 | 2.14382 | 1.85701 |
| lncRNA28330 | "x" | chr07:60764316-60768090 | 4.45855 | 3.9984 |
| lncRNA03187 | "u" | chr01:81837017-81837993 | 1.06289 | 0.851767 |
| lncRNA34644 | "x" | chr09:41844926-41847439 | 11.1669 | 6.90219 |
| lncRNA34819 | "u" | chr09:56005256-56006134 | 1.17617 | 0.943189 |
| lncRNA42081 | "x" | chr11:35949958-35955757 | 3.80944 | 5.40612 |
| lncRNA45044 | "u" | chr12:3717297-3717595 | 28.7058 | 23.1423 |
| lncRNA02785 | "x" | chr01:77606606-77612796 | 2.01523 | 2.43824 |
| lncRNA19261 | "x" | chr04:56303468-56307353 | 3.13563 | 2.43925 |
| lncRNA40251 | "u" | chr10:57874355-57874591 | 107.319 | 87.1379 |
| lncRNA40479 | "u" | chr10:60519027-60519455 | 5.50658 | 4.47268 |
| lncRNA35539 | "x" | chr09:67447769-67454617 | 6.06969 | 5.20017 |
| lncRNA12289 | "x" | chr03:33354361-33370213 | 25.9871 | 34.3254 |
| lncRNA16879 | "x" | chr04:41657944-41671566 | 1.00447 | 1.20819 |
| lncRNA18272 | "x" | chr04:4361228-4366187 | 1.32273 | 1.10937 |
| lncRNA11393 | "x" | chr02:48994051-48996956 | 6.39798 | 8.43448 |
| lncRNA19076 | "x" | chr04:53237130-53239123 | 0.801092 | 1.2311 |
| lncRNA32636 | "u" | chr08:22830435-22831899 | 2.18496 | 2.48868 |
| lncRNA44547 | "x" | chr11:52664922-52667491 | 1.08469 | 0.840871 |
| lncRNA27070 | "x" | chr07:176823-181975 | 3.20117 | 3.69976 |
| lncRNA44183 | "x" | chr11:48570084-48574651 | 4.46107 | 5.09983 |
| lncRNA20326 | "o" | chr05:5799500-5800416 | 14.3523 | 8.38719 |
| lncRNA28154 | "x" | chr07:58390689-58399838 | 12.4112 | 15.7805 |
| lncRNA34347 | "u" | chr09:8629546-8631376 | 2.98363 | 2.1714 |
| lncRNA08427 | "x" | chr02:42877425-42883768 | 0.736071 | 0.646944 |
| lncRNA43048 | "x" | chr11:2056743-2064575 | 4.78555 | 3.79463 |
| lncRNA10735 | "x" | chr02:42327853-42330617 | 1.5187 | 1.89221 |
| lncRNA21436 | "u" | chr05:64083262-64085071 | 7.40112 | 8.13827 |
| lncRNA48117 | "x" | chr12:63240061-63245063 | 9.29278 | 12.2894 |
| lncRNA33457 | "x" | chr08:58373700-58385976 | 6.83611 | 5.38082 |
| lncRNA01855 | "u" | chr01:46534416-46535607 | 3.51185 | 3.08899 |
| lncRNA39671 | "x" | chr10:21887499-21902534 | 5.48953 | 6.9806 |
| lncRNA38887 | "x" | chr10:63201227-63204679 | 1.91931 | 2.64208 |
| lncRNA32696 | "x" | chr08:28490018-28503056 | 0.814025 | 0.646064 |
| lncRNA07793 | "x" | chr02:35942238-35951338 | 2.48824 | 3.51463 |
| lncRNA01619 | "u" | chr01:22596852-22597209 | 5.70083 | 7.46405 |
| lncRNA36351 | "x" | chr09:41660834-41663619 | 0.90099 | 1.32074 |
| lncRNA41803 | "u" | chr11:14683277-14688445 | 1.76651 | 1.5973 |
| lncRNA30161 | "x" | chr07:62185204-62189970 | 1.4909 | 1.23789 |
| lncRNA09279 | "x" | chr02:13959589-13961208 | 10.3528 | 13.1575 |
| lncRNA42119 | "x" | chr11:36553142-36555518 | 1.45476 | 1.27601 |
| lncRNA48307 | "x" | chr12:64786483-64789002 | 1.23491 | 1.50283 |
| lncRNA07956 | "x" | chr02:37785909-37796329 | 17.5924 | 24.2365 |
| lncRNA15970 | "x" | chr03:63857884-63862728 | 1.20175 | 1.76931 |
| lncRNA23822 | "x" | chr06:29366137-29368048 | 26.5363 | 32.9526 |
| lncRNA30798 | "x" | chr08:6476388-6486552 | 3.46128 | 2.7179 |
| lncRNA12846 | "x" | chr03:54207945-54209199 | 2.3902 | 3.76201 |
| lncRNA47439 | "x" | chr12:35197505-35216182 | 82.7326 | 61.6476 |
| lncRNA14255 | "x" | chr03:11762615-11763329 | 126.976 | 146.207 |
| lncRNA37594 | "x" | chr10:4259618-4260333 | 5.4355 | 7.10245 |
| lncRNA21732 | "x" | chr05:2707861-2717543 | 14.4098 | 11.9003 |
| lncRNA37611 | "u" | chr10:4700128-4700575 | 5.36954 | 4.36658 |
| lncRNA09989 | "x" | chr02:34096945-34101392 | 1.90323 | 1.28515 |
| lncRNA30239 | "x" | chr07:62960748-62962489 | 2.08201 | 3.01891 |
| lncRNA12606 | "x" | chr03:48897779-48901345 | 2.39366 | 2.15448 |
| lncRNA16831 | "x" | chr04:33412304-33414560 | 3.83833 | 4.75949 |
| lncRNA14616 | "x" | chr03:37712280-37720423 | 1.26521 | 1.002 |
| lncRNA20787 | "x" | chr05:36887238-36890935 | 4.88724 | 4.01612 |
| lncRNA35065 | "x" | chr09:61655002-61662278 | 2.16041 | 2.88582 |
| lncRNA19239 | "x" | chr04:55854785-55859981 | 1.24632 | 1.07169 |
| lncRNA31633 | "x" | chr08:57304882-57314726 | 2.71365 | 1.88389 |
| lncRNA37882 | "x" | chr10:27598549-27603127 | 3.43315 | 3.8549 |
| lncRNA46989 | "x" | chr12:4997778-4999879 | 6.82814 | 7.48023 |
| lncRNA18726 | "x" | chr04:31424455-31427145 | 1.87784 | 1.67026 |
| lncRNA18543 | "x" | chr04:17399320-17400397 | 28.8205 | 21.4555 |
| lncRNA41376 | "x" | chr11:4209975-4214584 | 17.0304 | 18.9364 |
| lncRNA22071 | "x" | chr05:8012501-8015024 | 0.863827 | 0.614575 |
| lncRNA44899 | "x" | chr12:2340212-2343067 | 0.901524 | 1.17261 |
| lncRNA38734 | "x" | chr10:61576687-61581704 | 9.81005 | 15.7521 |
| lncRNA43499 | "x" | chr11:9318757-9323559 | 12.3839 | 8.6765 |
| lncRNA16444 | "x" | chr04:4872991-4878203 | 1.19289 | 0.840845 |
| lncRNA17187 | "x" | chr04:54444394-54449597 | 1.00473 | 0.728907 |
| lncRNA45479 | "x" | chr12:29752533-29754365 | 20.3191 | 23.9103 |
| lncRNA04427 | "u" | chr01:10289643-10292045 | 3.69132 | 4.08025 |
| lncRNA21233 | "x" | chr05:61591817-61598511 | 1.66007 | 1.26331 |
| lncRNA38535 | "x" | chr10:59742410-59744990 | 7.38553 | 10.2619 |
| lncRNA02243 | "u" | chr01:69582274-69583395 | 0.922103 | 1.1138 |
| lncRNA16074 | "x" | chr04:195807-199205 | 56.6981 | 42.4804 |
| lncRNA42670 | "x" | chr11:51936686-51942097 | 6.2838 | 5.04979 |
| lncRNA45721 | "x" | chr12:43267878-43268699 | 1.22137 | 1.58852 |
| lncRNA11374 | "x" | chr02:48823829-48828106 | 0.867519 | 0.603078 |
| lncRNA17503 | "x" | chr04:59294994-59304028 | 1.93779 | 2.56004 |
| lncRNA31496 | "x" | chr08:55196941-55214508 | 0.690304 | 0.969039 |
| lncRNA01330 | "x" | chr01:2948573-2955890 | 0.718135 | 0.399629 |
| lncRNA21946 | "x" | chr05:5805481-5808679 | 0.951556 | 1.27719 |
| lncRNA35637 | "x" | chr09:685040-686018 | 22.2138 | 19.9486 |
| lncRNA20153 | "x" | chr05:3110784-3115464 | 2.11761 | 1.72633 |
| lncRNA11643 | "x" | chr03:2146444-2154565 | 25.556 | 20.2732 |
| lncRNA29974 | "x" | chr07:60012266-60019147 | 1.2081 | 1.79315 |
| lncRNA07489 | "x" | chr02:32182791-32187185 | 0.765825 | 0.583208 |
| lncRNA35844 | "x" | chr09:3540686-3545497 | 1.42344 | 1.68888 |
| lncRNA33446 | "x" | chr08:58267855-58273968 | 27.0166 | 31.3612 |
| lncRNA35442 | "x" | chr09:66517948-66522458 | 0.774332 | 0.54789 |
| lncRNA38538 | "x" | chr10:59753767-59758728 | 6.96789 | 5.1385 |
| lncRNA41140 | "x" | chr11:1546570-1548837 | 38.1211 | 24.9047 |
| lncRNA43883 | "u" | chr11:35825135-35828631 | 18.3886 | 22.6297 |
| lncRNA46445 | "x" | chr12:64969738-64984594 | 2.94135 | 3.54329 |
| lncRNA10706 | "x" | chr02:42008367-42020239 | 1.32887 | 1.48819 |
| lncRNA35051 | "x" | chr09:61435841-61436867 | 3.73271 | 4.49417 |
| lncRNA41499 | "x" | chr11:5557816-5562111 | 22.096 | 17.9562 |
| lncRNA42566 | "u" | chr11:50754057-50754664 | 33.647 | 36.9845 |
| lncRNA44982 | "x" | chr12:3165220-3165759 | 1.46292 | 1.90859 |
| lncRNA46109 | "x" | chr12:61996547-62000973 | 1.5216 | 1.73276 |
| lncRNA21404 | "x" | chr05:63692189-63694635 | 1.44557 | 1.67598 |
| lncRNA44362 | "x" | chr11:50759435-50763354 | 109.639 | 142.72 |
| lncRNA37378 | "x" | chr10:1210904-1214025 | 1.89463 | 2.30962 |
| lncRNA46655 | "x" | chr12:1329789-1334904 | 5.44195 | 6.39191 |
| lncRNA46941 | "x" | chr12:4397879-4403188 | 8.52994 | 10.3711 |
| lncRNA06188 | "x" | chr01:83288461-83291680 | 0.72362 | 0.560022 |
| lncRNA11147 | "x" | chr02:46475666-46479466 | 1.70366 | 1.44267 |
| lncRNA24935 | "x" | chr06:43901231-43906883 | 7.38197 | 8.61096 |
| lncRNA25359 | "x" | chr06:2668953-2670429 | 0.91356 | 1.08308 |
| lncRNA46360 | "x" | chr12:64230351-64238790 | 4.86286 | 5.74974 |
| lncRNA32586 | "x" | chr08:16760674-16774732 | 0.780607 | 0.663468 |
| lncRNA44944 | "x" | chr12:2891036-2892240 | 6.66269 | 5.96074 |
| lncRNA41139 | "x" | chr11:1546570-1548837 | 4.62111 | 3.23366 |
| lncRNA04344 | "u" | chr01:5877254-5880947 | 2.66566 | 2.41495 |
| lncRNA37845 | "x" | chr10:23106628-23108786 | 2.12621 | 2.39552 |
| lncRNA33588 | "u" | chr08:60111038-60116116 | 1.66101 | 1.49555 |
| lncRNA10710 | "x" | chr02:42021136-42030203 | 1.55962 | 1.74313 |
| lncRNA27138 | "x" | chr07:1047065-1047703 | 5.04565 | 6.57224 |
| lncRNA09303 | "x" | chr02:15374577-15384944 | 0.944611 | 0.767859 |
| lncRNA27464 | "x" | chr07:8648611-8649396 | 76.0328 | 67.5148 |
| lncRNA06221 | "x" | chr01:83739840-83746742 | 1.68599 | 2.81749 |
| lncRNA40938 | "u" | chr10:64536507-64537948 | 9.22283 | 10.1419 |
| lncRNA20729 | "u" | chr05:30168382-30169342 | 8.39318 | 9.34016 |
| lncRNA32309 | "x" | chr08:2038690-2043280 | 1.92401 | 2.63127 |
| lncRNA08705 | "x" | chr02:45671699-45676268 | 2.97782 | 2.39883 |
| lncRNA06642 | "x" | chr01:88321478-88322795 | 1178.32 | 1061.13 |
| lncRNA17253 | "x" | chr04:55617931-55632414 | 0.741148 | 0.926733 |
| lncRNA11995 | "x" | chr03:11887805-11899902 | 2.60592 | 2.13059 |
| lncRNA42679 | "x" | chr11:52002517-52004953 | 13.2369 | 16.5454 |
| lncRNA43144 | "x" | chr11:3164930-3166346 | 1.79428 | 1.40414 |
| lncRNA45706 | "x" | chr12:42890822-42893651 | 1.74509 | 2.00187 |
| lncRNA29176 | "u" | chr07:9800179-9801782 | 0.920373 | 1.08209 |
| lncRNA20212 | "x" | chr05:3973338-3980949 | 10.1906 | 12.5276 |
| lncRNA36282 | "x" | chr09:30608810-30610202 | 5.53508 | 6.52888 |
| lncRNA47047 | "x" | chr12:5860246-5866862 | 6.02149 | 4.79271 |
| lncRNA46831 | "x" | chr12:3266696-3268481 | 112.465 | 136.076 |
| lncRNA41624 | "u" | chr11:8172865-8192556 | 0.785422 | 0.525105 |
| lncRNA44293 | "x" | chr11:50049702-50053095 | 1.98306 | 2.45765 |
| lncRNA15085 | "x" | chr03:52917016-52920423 | 2.36027 | 1.89727 |
| lncRNA19197 | "x" | chr04:55133072-55140024 | 0.973512 | 0.68505 |
| lncRNA35921 | "x" | chr09:4703920-4707518 | 9.79228 | 7.46367 |
| lncRNA37077 | "x" | chr09:65304825-65309530 | 3.4438 | 3.11156 |
| lncRNA09962 | "x" | chr02:33842724-33851382 | 0.689655 | 0.471775 |
| lncRNA19889 | "x" | chr04:63861453-63864472 | 3.85489 | 2.99057 |
| lncRNA32875 | "u" | chr08:46825012-46825235 | 371.656 | 314.731 |
| lncRNA39526 | "x" | chr10:8707026-8709841 | 3.99093 | 4.60599 |
| lncRNA43887 | "u" | chr11:35828783-35829309 | 6.72341 | 5.70097 |
| lncRNA46739 | "x" | chr12:2227328-2232351 | 0.983681 | 1.21315 |
| lncRNA46880 | "x" | chr12:3661120-3669941 | 14.2297 | 11.9461 |
| lncRNA20345 | "u" | chr05:5970535-5972112 | 13.0522 | 14.2159 |
| lncRNA33214 | "x" | chr08:55131302-55135662 | 0.640426 | 0.862947 |
| lncRNA33623 | "x" | chr08:60536401-60540654 | 1.60677 | 2.24286 |
| lncRNA15557 | "x" | chr03:59364980-59368703 | 24.9709 | 19.8605 |
| lncRNA24696 | "x" | chr06:41366589-41370323 | 6.69879 | 8.68076 |
| lncRNA40859 | "x" | chr10:63867299-63871612 | 2.30672 | 1.90628 |
| lncRNA10191 | "x" | chr02:36297466-36302033 | 33.8799 | 38.4441 |
| lncRNA12412 | "u" | chr03:45062156-45069845 | 1.40088 | 1.54328 |
| lncRNA14978 | "u" | chr03:49948397-49948822 | 10.7973 | 9.09329 |
| lncRNA21486 | "x" | chr05:64530653-64535425 | 6.66498 | 9.86586 |
| lncRNA23992 | "x" | chr06:32942712-32944228 | 1.66203 | 2.18746 |
| lncRNA03810 | "x" | chr01:88383746-88387401 | 3.46215 | 4.30458 |
| lncRNA37042 | "x" | chr09:64900264-64902631 | 5.7202 | 7.66152 |
| lncRNA47472 | "u" | chr12:36127009-36136643 | 3.16574 | 3.4309 |
| lncRNA10722 | "u" | chr02:42142005-42142376 | 103.299 | 93.6525 |
| lncRNA21623 | "x" | chr05:1195250-1197463 | 5.23283 | 7.1458 |
| lncRNA31881 | "x" | chr08:60318784-60323467 | 35.7321 | 25.7511 |
| lncRNA40152 | "x" | chr10:52626789-52631217 | 8.43544 | 7.00715 |
| lncRNA36239 | "x" | chr09:27230672-27232263 | 12.6561 | 17.5983 |
| lncRNA15679 | "x" | chr03:60787313-60791258 | 3.58122 | 2.70135 |
| lncRNA09027 | "x" | chr02:49084875-49088035 | 1.9119 | 1.4478 |
| lncRNA12782 | "x" | chr03:53235915-53245445 | 5.98138 | 7.55233 |
| lncRNA14073 | "u" | chr03:8414971-8416343 | 1.25789 | 1.06246 |
| lncRNA44273 | "x" | chr11:49843260-49848948 | 10.5194 | 9.43504 |
| lncRNA11263 | "x" | chr02:47688582-47692021 | 2.19351 | 2.58433 |
| lncRNA31652 | "x" | chr08:57594646-57603672 | 1.6539 | 3.22663 |
| lncRNA32287 | "x" | chr08:1737351-1739951 | 0.811623 | 0.52093 |
| lncRNA06426 | "x" | chr01:86040019-86047935 | 1.11358 | 0.816485 |
| lncRNA29636 | "x" | chr07:54807978-54814690 | 3.26299 | 2.50126 |
| lncRNA32424 | "x" | chr08:3679125-3682484 | 1.98286 | 2.31504 |
| lncRNA38416 | "x" | chr10:58109247-58135952 | 10.3005 | 5.23489 |
| lncRNA32035 | "x" | chr08:62081442-62086597 | 3.85368 | 3.00611 |
| lncRNA01946 | "x" | chr01:53799877-53802653 | 0.725127 | 0.625449 |
| lncRNA19588 | "x" | chr04:60707370-60710275 | 203.112 | 288.963 |
| lncRNA42961 | "x" | chr11:1357489-1361277 | 17.7445 | 16.096 |
| lncRNA41294 | "x" | chr11:3191063-3196487 | 7.73163 | 9.71369 |
| lncRNA25288 | "x" | chr06:1784006-1785855 | 1.59394 | 1.15754 |
| lncRNA14891 | "x" | chr03:47874480-47880681 | 1.52001 | 0.984764 |
| lncRNA42557 | "x" | chr11:50641327-50644510 | 0.712415 | 0.503196 |
| lncRNA03445 | "x" | chr01:84682629-84685312 | 8.98352 | 7.56107 |
| lncRNA13100 | "x" | chr03:58573164-58583411 | 3.54432 | 4.72222 |
| lncRNA27721 | "x" | chr07:42878617-42882589 | 16.972 | 13.7417 |
| lncRNA46779 | "x" | chr12:2704556-2706846 | 2.55797 | 2.11342 |
| lncRNA47588 | "x" | chr12:43016136-43018624 | 4.76711 | 3.51031 |
| lncRNA42045 | "x" | chr11:34840446-34854035 | 20.5089 | 16.0876 |
| lncRNA46238 | "x" | chr12:63259974-63269260 | 7.71813 | 5.98619 |
| lncRNA44783 | "x" | chr12:1220889-1223936 | 5.65483 | 7.12693 |
| lncRNA40575 | "x" | chr10:61391471-61394174 | 0.96531 | 0.716663 |
| lncRNA06794 | "x" | chr01:89896493-89898383 | 4.7986 | 3.19055 |
| lncRNA45197 | "x" | chr12:5718432-5721319 | 3.64791 | 4.38758 |
| lncRNA48261 | "x" | chr12:64455003-64460140 | 17.5498 | 22.5188 |
| lncRNA38603 | "x" | chr10:60394336-60396960 | 1.53677 | 1.17107 |
| lncRNA08191 | "x" | chr02:40404203-40408880 | 13.4385 | 16.485 |
| lncRNA25083 | "x" | chr06:45374747-45377192 | 0.833157 | 1.02957 |
| lncRNA42344 | "x" | chr11:47901622-47906481 | 5.86865 | 5.15253 |
| lncRNA43274 | "x" | chr11:4740522-4743362 | 1.05027 | 0.769763 |
| lncRNA28994 | "x" | chr07:3121462-3128220 | 13.5708 | 16.9927 |
| lncRNA11446 | "x" | chr02:49544949-49547904 | 4.28221 | 5.22097 |
| lncRNA14800 | "x" | chr03:46322575-46326863 | 2.87403 | 2.28069 |
| lncRNA11656 | "u" | chr03:2377483-2378548 | 1.4279 | 1.68183 |
| lncRNA14365 | "u" | chr03:17147647-17148191 | 1.23639 | 1.62274 |
| lncRNA27360 | "u" | chr07:4493387-4493761 | 3.58846 | 4.69895 |
| lncRNA20194 | "x" | chr05:3767464-3769326 | 10.9588 | 7.91079 |
| lncRNA46489 | "x" | chr12:65289533-65290834 | 1.27533 | 1.66757 |
| lncRNA14675 | "x" | chr03:42385874-42391566 | 8.17082 | 6.10191 |
| lncRNA43296 | "x" | chr11:4951032-4953962 | 3.55408 | 4.64476 |
| lncRNA35150 | "x" | chr09:62765358-62766136 | 6.70451 | 8.44577 |
| lncRNA35625 | "x" | chr09:605625-607732 | 2.7314 | 2.26638 |
| lncRNA37942 | "u" | chr10:34429206-34429566 | 12.4914 | 10.4015 |
| lncRNA26806 | "u" | chr06:43490938-43491292 | 4.67943 | 6.04852 |
| lncRNA25910 | "u" | chr06:32487710-32488351 | 7.35668 | 8.31927 |
| lncRNA34515 | "u" | chr09:21215899-21217060 | 8.45442 | 9.32709 |
| lncRNA14809 | "x" | chr03:46405572-46407538 | 1.02357 | 0.825636 |
| lncRNA25004 | "u" | chr06:44613691-44623029 | 40.4745 | 24.7636 |
| lncRNA36773 | "x" | chr09:61055488-61061294 | 2.79097 | 2.36036 |
| lncRNA30455 | "x" | chr07:65212625-65216103 | 1.49965 | 1.80336 |
| lncRNA06305 | "x" | chr01:84759758-84766136 | 9.9725 | 8.18993 |
| lncRNA29375 | "u" | chr07:35815126-35835301 | 7.04621 | 5.74243 |
| lncRNA45537 | "x" | chr12:32996287-32997087 | 2.71793 | 2.3252 |
| lncRNA46775 | "x" | chr12:2619473-2623849 | 0.57382 | 0.721702 |
| lncRNA28241 | "x" | chr07:59554357-59557127 | 1.30186 | 1.51139 |
| lncRNA23623 | "x" | chr06:20656437-20659051 | 3.49896 | 3.15322 |
| lncRNA15670 | "x" | chr03:60710386-60715329 | 1.3198 | 1.00176 |
| lncRNA21378 | "x" | chr05:63355134-63356447 | 0.60029 | 0.781765 |
| lncRNA08674 | "x" | chr02:45399100-45405782 | 8.90477 | 6.9281 |
| lncRNA16495 | "x" | chr04:5520044-5526092 | 2.89437 | 4.22842 |
| lncRNA40641 | "x" | chr10:61992121-61996358 | 2.76605 | 2.21627 |
| lncRNA11409 | "x" | chr02:49121178-49125517 | 18.0103 | 16.1283 |
| lncRNA11759 | "x" | chr03:7291650-7299172 | 9.19891 | 7.25917 |
| lncRNA33712 | "u" | chr08:61559264-61560433 | 2.88652 | 2.53278 |
| lncRNA35256 | "x" | chr09:64404040-64405561 | 0.497969 | 0.776303 |
| lncRNA46795 | "x" | chr12:2852171-2860231 | 28.3967 | 23.6442 |
| lncRNA08996 | "x" | chr02:48797709-48801198 | 2.27127 | 2.82212 |
| lncRNA36697 | "x" | chr09:59692999-59697548 | 0.971661 | 0.769066 |
| lncRNA05584 | "x" | chr01:76371765-76376193 | 1.09383 | 0.841362 |
| lncRNA13946 | "x" | chr03:6792277-6799634 | 1.14062 | 1.4717 |
| lncRNA19913 | "u" | chr05:31566-32406 | 3.47537 | 3.03286 |
| lncRNA33281 | "x" | chr08:56111005-56113627 | 6.60552 | 5.35003 |
| lncRNA48378 | "x" | chr12:65314111-65317211 | 1.3707 | 1.04825 |
| lncRNA14826 | "x" | chr03:46584787-46592143 | 2.45723 | 2.25913 |
| lncRNA16186 | "x" | chr04:1674661-1680865 | 13.4561 | 11.553 |
| lncRNA08541 | "x" | chr02:44111326-44119374 | 6.84171 | 7.97058 |
| lncRNA11466 | "x" | chr02:49807398-49821989 | 19.3853 | 16.1386 |
| lncRNA28751 | "x" | chr07:65118759-65130514 | 2.74194 | 2.09476 |
| lncRNA23512 | "x" | chr06:9564693-9565135 | 27.5043 | 34.0755 |
| lncRNA45008 | "x" | chr12:3429272-3438938 | 4.31696 | 5.53432 |
| lncRNA30908 | "u" | chr08:17666273-17667765 | 6.5385 | 7.19271 |
| lncRNA39243 | "x" | chr10:1705973-1710748 | 8.30885 | 7.7086 |
| lncRNA29161 | "x" | chr07:8779430-8791044 | 0.733211 | 0.592312 |
| lncRNA40271 | "x" | chr10:58049175-58060447 | 17.6416 | 22.6574 |
| lncRNA42733 | "x" | chr11:52494532-52497678 | 17.8358 | 14.216 |
| lncRNA08183 | "x" | chr02:40332072-40334019 | 0.671644 | 0.936259 |
| lncRNA28223 | "u" | chr07:59262057-59262816 | 2.57236 | 2.17439 |
| lncRNA41436 | "x" | chr11:4926574-4931762 | 8.88994 | 11.5918 |
| lncRNA44170 | "x" | chr11:48417850-48421332 | 3.89964 | 4.66374 |
| lncRNA35184 | "x" | chr09:63441328-63442395 | 1.00962 | 1.2223 |
| lncRNA32221 | "x" | chr08:945013-953304 | 1.48246 | 0.965058 |
| lncRNA12759 | "x" | chr03:52900673-52904929 | 2.14911 | 2.78383 |
| lncRNA44321 | "x" | chr11:50339389-50347382 | 2.36392 | 1.93499 |
| lncRNA02439 | "x" | chr01:72979535-72985606 | 1.84341 | 1.49983 |
| lncRNA29155 | "x" | chr07:8626399-8628434 | 11.004 | 14.8615 |
| lncRNA31796 | "x" | chr08:59430889-59434544 | 2.94654 | 2.35967 |
| lncRNA01416 | "x" | chr01:5033710-5037078 | 2.25963 | 2.68739 |
| lncRNA20718 | "u" | chr05:29293194-29293689 | 28.4494 | 31.5312 |
| lncRNA38579 | "x" | chr10:60143695-60149387 | 5.93739 | 6.91104 |
| lncRNA30087 | "x" | chr07:61188418-61189958 | 1.85005 | 2.3965 |
| lncRNA13603 | "x" | chr03:63953874-63956934 | 3.23746 | 3.68106 |
| lncRNA27754 | "x" | chr07:46785995-46786882 | 4.01076 | 4.6554 |
| lncRNA00475 | "x" | chr00:19656546-19657203 | 2.42665 | 2.14917 |
| lncRNA40263 | "u" | chr10:57986987-57988436 | 8.55833 | 9.37737 |
| lncRNA02876 | "x" | chr01:78524705-78530438 | 2.56575 | 2.15746 |
| lncRNA44735 | "u" | chr12:756222-761422 | 69.9538 | 60.2417 |
| lncRNA40148 | "u" | chr10:52560679-52561387 | 8.79414 | 9.83968 |
| lncRNA46620 | "u" | chr12:878023-878568 | 33.6983 | 30.5843 |
| lncRNA16941 | "u" | chr04:47184860-47185676 | 6.47345 | 5.77962 |
| lncRNA07329 | "x" | chr02:29468110-29477847 | 2.98587 | 2.35966 |
| lncRNA25605 | "x" | chr06:21351565-21354557 | 3.24638 | 2.67771 |
| lncRNA38521 | "x" | chr10:59627067-59628608 | 0.517324 | 0.674572 |
| lncRNA44552 | "x" | chr11:52713557-52728072 | 1.10706 | 0.851521 |
| lncRNA43443 | "x" | chr11:7820294-7844282 | 207.747 | 163.235 |
| lncRNA44891 | "x" | chr12:2271797-2280172 | 3.70412 | 4.79115 |
| lncRNA29255 | "x" | chr07:19453608-19456895 | 9.43609 | 7.17724 |
| lncRNA39267 | "x" | chr10:1911957-1918842 | 0.519761 | 0.722854 |
| lncRNA04189 | "x" | chr01:2485164-2492775 | 4.96875 | 3.67497 |
| lncRNA24674 | "x" | chr06:41210979-41212001 | 4.55268 | 4.05736 |
| lncRNA22058 | "x" | chr05:7795702-7801867 | 14.4319 | 13.154 |
| lncRNA11051 | "u" | chr02:45440641-45443060 | 7.23626 | 7.87555 |
| lncRNA17652 | "x" | chr04:61103129-61107755 | 1.18301 | 0.838119 |
| lncRNA48263 | "x" | chr12:64466749-64471027 | 2.36865 | 1.92026 |
| lncRNA01111 | "x" | chr01:406974-409234 | 3.01183 | 2.39905 |
| lncRNA02236 | "x" | chr01:69454141-69456938 | 2.06193 | 2.77457 |
| lncRNA09095 | "x" | chr02:49748275-49765101 | 0.758716 | 0.905168 |
| lncRNA45996 | "x" | chr12:49708562-49714242 | 7.76131 | 9.5404 |
| lncRNA23174 | "u" | chr05:64980160-64980515 | 46.1529 | 40.9577 |
| lncRNA46864 | "x" | chr12:3517599-3519456 | 8.63417 | 6.9718 |
| lncRNA28297 | "u" | chr07:60297034-60300742 | 2.16471 | 1.96532 |
| lncRNA38601 | "x" | chr10:60389905-60392568 | 21.0341 | 24.1472 |
| lncRNA32395 | "x" | chr08:3060067-3061635 | 3.39672 | 4.12107 |
| lncRNA17791 | "u" | chr04:62731728-62732855 | 1.61639 | 1.39549 |
| lncRNA02580 | "x" | chr01:74601560-74609120 | 13.625 | 16.0073 |
| lncRNA01434 | "x" | chr01:5507331-5510230 | 0.606895 | 0.782755 |
| lncRNA08145 | "x" | chr02:40004457-40006288 | 1.25511 | 0.998943 |
| lncRNA16617 | "x" | chr04:9609623-9610848 | 1.90279 | 2.17728 |
| lncRNA38575 | "x" | chr10:60110733-60114570 | 1.25285 | 0.843188 |
| lncRNA43100 | "x" | chr11:2758332-2764075 | 4.27313 | 5.23414 |
| lncRNA39362 | "x" | chr10:3151103-3152540 | 143.128 | 107.302 |
| lncRNA27687 | "x" | chr07:39099381-39103804 | 1.75547 | 2.09906 |
| lncRNA30764 | "x" | chr08:4408026-4415047 | 3.52567 | 4.45827 |
| lncRNA25880 | "x" | chr06:32080857-32092835 | 2.30219 | 3.49638 |
| lncRNA40986 | "x" | chr11:112866-127081 | 6.60438 | 8.2276 |
| lncRNA07668 | "x" | chr02:34557085-34566850 | 32.3058 | 39.818 |
| lncRNA27276 | "x" | chr07:2853128-2858349 | 2.1211 | 2.55218 |
| lncRNA42975 | "x" | chr11:1435169-1438740 | 0.832277 | 0.724246 |
| lncRNA24938 | "x" | chr06:43936689-43937316 | 61.8698 | 75.3313 |
| lncRNA12399 | "x" | chr03:44778617-44782582 | 3.52477 | 2.95659 |
| lncRNA09042 | "x" | chr02:49202210-49205167 | 4.82589 | 3.92815 |
| lncRNA40687 | "x" | chr10:62377261-62380722 | 25.6524 | 21.1941 |
| lncRNA45056 | "x" | chr12:3877746-3880867 | 1.78918 | 1.45197 |
| lncRNA25001 | "u" | chr06:44612329-44612806 | 7.2538 | 6.20861 |
| lncRNA37291 | "u" | chr10:245126-246374 | 1.88463 | 2.17129 |
| lncRNA01035 | "u" | chr00:20538399-20540505 | 1.82479 | 1.62938 |
| lncRNA06957 | "x" | chr02:11977399-11978995 | 15.6975 | 19.2534 |
| lncRNA29377 | "u" | chr07:35815126-35835301 | 81.2335 | 43.7217 |
| lncRNA38320 | "x" | chr10:52669436-52671015 | 5.34447 | 4.01459 |
| lncRNA25034 | "u" | chr06:44839123-44840481 | 1.78573 | 2.03394 |
| lncRNA40947 | "x" | chr10:64600275-64604692 | 0.738957 | 0.955775 |
| lncRNA05350 | "x" | chr01:73030236-73033351 | 8.09695 | 6.0227 |
| lncRNA10850 | "x" | chr02:43514318-43520636 | 56.1426 | 42.7792 |
| lncRNA46691 | "x" | chr12:1741358-1746064 | 1.4605 | 1.89336 |
| lncRNA32526 | "u" | chr08:7997533-7998311 | 2.19294 | 1.86362 |
| lncRNA36634 | "x" | chr09:58080450-58088531 | 5.37494 | 4.12732 |
| lncRNA33286 | "x" | chr08:56198769-56206553 | 10.5346 | 9.76133 |
| lncRNA16242 | "x" | chr04:2424176-2425648 | 1.31277 | 0.994023 |
| lncRNA42262 | "x" | chr11:46404536-46410373 | 3.35447 | 2.42675 |
| lncRNA39793 | "x" | chr10:35783788-35806501 | 7.81791 | 10.1595 |
| lncRNA09496 | "u" | chr02:23637828-23639482 | 7.44746 | 6.80251 |
| lncRNA38932 | "x" | chr10:63557448-63558575 | 45.1572 | 51.875 |
| lncRNA35144 | "x" | chr09:62631865-62639953 | 1.93764 | 1.51106 |
| lncRNA25049 | "x" | chr06:45010505-45012800 | 6.39377 | 5.68025 |
| lncRNA41652 | "x" | chr11:8634312-8640219 | 1.52267 | 1.37484 |
| lncRNA07751 | "x" | chr02:35468991-35471461 | 13.8316 | 10.2827 |
| lncRNA40174 | "u" | chr10:53498775-53500057 | 1.36686 | 1.17234 |
| lncRNA46194 | "x" | chr12:62767796-62772259 | 5.80711 | 7.19775 |
| lncRNA09012 | "x" | chr02:48947509-48952106 | 3.69702 | 3.11029 |
| lncRNA21195 | "x" | chr05:61101696-61108223 | 13.9178 | 17.7208 |
| lncRNA43379 | "x" | chr11:5932338-5933563 | 1.76485 | 2.06993 |
| lncRNA18026 | "x" | chr04:1157354-1158028 | 2.55563 | 2.17087 |
| lncRNA31140 | "x" | chr08:46828225-46831672 | 29.5055 | 24.98 |
| lncRNA18600 | "u" | chr04:22238435-22239233 | 3.29534 | 3.7517 |
| lncRNA10004 | "x" | chr02:34281464-34286083 | 5.97746 | 4.83621 |
| lncRNA16923 | "x" | chr04:45455419-45471463 | 0.639778 | 0.383288 |
| lncRNA47886 | "u" | chr12:50047512-50049908 | 2.8358 | 3.13187 |
| lncRNA01612 | "o" | chr01:22059383-22060856 | 0.543168 | 0.645976 |
| lncRNA07308 | "x" | chr02:28951770-28955056 | 1.29709 | 1.51306 |
| lncRNA35370 | "x" | chr09:65605317-65639030 | 1.33823 | 1.11775 |
| lncRNA33513 | "x" | chr08:59116807-59118652 | 4.01492 | 2.97339 |
| lncRNA02353 | "x" | chr01:71475302-71486951 | 5.49674 | 6.5159 |
| lncRNA16222 | "x" | chr04:2185453-2191242 | 0.9538 | 0.730478 |
| lncRNA44384 | "x" | chr11:51043075-51047264 | 0.946641 | 1.20345 |
| lncRNA42413 | "x" | chr11:49098931-49102726 | 22.5976 | 26.3966 |
| lncRNA03693 | "x" | chr01:87151556-87155525 | 7.16951 | 7.97555 |
| lncRNA29784 | "x" | chr07:57410328-57416583 | 1.59473 | 1.96348 |
| lncRNA25396 | "x" | chr06:3284451-3287075 | 3.43458 | 4.72249 |
| lncRNA40766 | "u" | chr10:63072566-63073172 | 4.44448 | 5.12124 |
| lncRNA10927 | "x" | chr02:44330412-44335237 | 1.34071 | 1.66242 |
| lncRNA44797 | "x" | chr12:1348237-1352431 | 5.13912 | 6.58845 |
| lncRNA06375 | "x" | chr01:85417029-85427501 | 0.84357 | 0.614986 |
| lncRNA27216 | "x" | chr07:1995884-1997283 | 2.20052 | 2.86725 |
| lncRNA12251 | "x" | chr03:30527779-30530150 | 0.646736 | 0.740969 |
| lncRNA43026 | "x" | chr11:1807763-1810656 | 9.59547 | 8.25774 |
| lncRNA43174 | "x" | chr11:3499438-3506198 | 14.6765 | 16.6584 |
| lncRNA08083 | "x" | chr02:39294369-39295565 | 16.3431 | 11.3518 |
| lncRNA38532 | "x" | chr10:59704816-59706809 | 30.8816 | 33.318 |
| lncRNA48241 | "x" | chr12:64267673-64273580 | 3.41422 | 2.31915 |
| lncRNA30735 | "x" | chr08:3514106-3519633 | 0.600602 | 0.776122 |
| lncRNA17432 | "x" | chr04:58301850-58305291 | 1.13721 | 0.829982 |
| lncRNA44282 | "x" | chr11:49907175-49913103 | 3.05904 | 3.82198 |
| lncRNA02551 | "x" | chr01:74319148-74336096 | 1.74727 | 1.36404 |
| lncRNA14120 | "x" | chr03:9134532-9135005 | 235.275 | 179.729 |
| lncRNA24929 | "x" | chr06:43843182-43849644 | 1.05258 | 0.775825 |
| lncRNA47453 | "u" | chr12:35917534-35920173 | 2.88067 | 3.16262 |
| lncRNA06240 | "x" | chr01:83978627-83987203 | 1.42061 | 0.916143 |
| lncRNA33614 | "x" | chr08:60411765-60417957 | 4.23365 | 5.53508 |
| lncRNA13850 | "x" | chr03:1986225-1991553 | 2.38351 | 1.87145 |
| lncRNA46424 | "x" | chr12:64789172-64795487 | 3.48253 | 4.21387 |
| lncRNA17096 | "u" | chr04:52683329-52685463 | 1.68775 | 1.50495 |
| lncRNA39234 | "x" | chr10:1522517-1526065 | 30.4318 | 27.0814 |
| lncRNA21453 | "x" | chr05:64191535-64194770 | 3.41134 | 3.1296 |
| lncRNA33779 | "u" | chr08:62332560-62335439 | 2.41413 | 2.19476 |
| lncRNA11328 | "u" | chr02:48328718-48329465 | 6.00395 | 5.33728 |
| lncRNA41541 | "x" | chr11:6185689-6211407 | 15.2339 | 19.7509 |
| lncRNA38738 | "x" | chr10:61602429-61606823 | 10.8017 | 8.67917 |
| lncRNA45813 | "x" | chr12:45579296-45590307 | 14.8144 | 17.1073 |
| lncRNA17929 | "x" | chr04:146130-148224 | 26.9222 | 17.6401 |
| lncRNA38544 | "x" | chr10:59792042-59795246 | 14.1481 | 10.4949 |
| lncRNA43056 | "x" | chr11:2181482-2185868 | 24.625 | 34.1513 |
| lncRNA06016 | "x" | chr01:81450574-81454548 | 0.810467 | 0.904058 |
| lncRNA46871 | "x" | chr12:3591088-3593379 | 24.901 | 27.7344 |
| lncRNA01381 | "x" | chr01:4122411-4126861 | 1.49875 | 1.1985 |
| lncRNA09691 | "x" | chr02:30219292-30220400 | 6.88879 | 5.6751 |
| lncRNA33453 | "x" | chr08:58353283-58359554 | 0.707139 | 0.992762 |
| lncRNA45787 | "x" | chr12:44759598-44772028 | 25.7162 | 30.8539 |
| lncRNA01722 | "u" | chr01:29989602-29990062 | 6.00804 | 5.10438 |
| lncRNA27868 | "x" | chr07:53018870-53019624 | 3.5081 | 2.88455 |
| lncRNA12265 | "u" | chr03:31775882-31776449 | 9.84765 | 8.543 |
| lncRNA12516 | "x" | chr03:46666654-46668863 | 18.8452 | 16.1298 |
| lncRNA38705 | "x" | chr10:61262270-61265938 | 1.02613 | 1.33343 |
| lncRNA01025 | "u" | chr00:20195736-20196642 | 0.945966 | 0.786075 |
| lncRNA28367 | "u" | chr07:61222960-61223699 | 1.40439 | 1.16165 |
| lncRNA28752 | "x" | chr07:65149017-65152954 | 14.7027 | 11.4878 |
| lncRNA41650 | "x" | chr11:8631105-8633554 | 71.4262 | 64.5778 |
| lncRNA46994 | "x" | chr12:5057439-5062841 | 7.99902 | 6.58457 |
| lncRNA11222 | "x" | chr02:47205093-47212067 | 2.06324 | 1.73336 |
| lncRNA39215 | "x" | chr10:1322843-1333280 | 1.65134 | 1.29656 |
| lncRNA39871 | "x" | chr10:41635719-41638245 | 1.57429 | 1.30655 |
| lncRNA41833 | "x" | chr11:15646306-15654641 | 8.00252 | 9.82295 |
| lncRNA27054 | "x" | chr06:45950565-45959677 | 1.22391 | 0.958984 |
| lncRNA37449 | "x" | chr10:2108444-2114253 | 9.34408 | 11.2049 |
| lncRNA28727 | "x" | chr07:64876217-64880415 | 7.17681 | 6.14184 |
| lncRNA47990 | "x" | chr12:61900822-61904696 | 3.23586 | 3.71725 |
| lncRNA19595 | "x" | chr04:60818555-60824311 | 2.65219 | 3.30522 |
| lncRNA36268 | "u" | chr09:29745929-29748190 | 0.810296 | 0.919095 |
| lncRNA48188 | "x" | chr12:63777455-63782513 | 10.3897 | 7.76983 |
| lncRNA37231 | "x" | chr09:67113260-67115149 | 3.11123 | 3.81041 |
| lncRNA48362 | "x" | chr12:65186152-65189023 | 4.45742 | 6.39256 |
| lncRNA44113 | "x" | chr11:47257119-47269249 | 20.6311 | 24.4975 |
| lncRNA11221 | "x" | chr02:47205093-47212067 | 2.45178 | 2.02522 |
| lncRNA33746 | "x" | chr08:61956448-61961285 | 1.67795 | 1.51303 |
| lncRNA08994 | "x" | chr02:48797709-48801198 | 3.52677 | 4.1307 |
| lncRNA38667 | "x" | chr10:60937230-60942101 | 4.40868 | 3.9055 |
| lncRNA19975 | "x" | chr05:697947-703061 | 2.09479 | 2.36799 |
| lncRNA38080 | "x" | chr10:42607235-42609593 | 1.42433 | 1.57923 |
| lncRNA48078 | "x" | chr12:62792417-62795041 | 7.47696 | 9.34949 |
| lncRNA26278 | "x" | chr06:37689193-37694087 | 6.56145 | 5.74463 |
| lncRNA33296 | "x" | chr08:56343139-56348032 | 1.04278 | 1.19443 |
| lncRNA42659 | "x" | chr11:51816643-51817331 | 0.675438 | 0.530145 |
| lncRNA18719 | "x" | chr04:31105831-31110158 | 2.13068 | 1.69856 |
| lncRNA23572 | "x" | chr06:16057616-16067362 | 0.872239 | 1.0722 |
| lncRNA38676 | "x" | chr10:61048316-61049928 | 4.17631 | 3.42429 |
| lncRNA04823 | "u" | chr01:53934382-53938203 | 3.59506 | 3.89152 |
| lncRNA22883 | "x" | chr05:61604829-61607750 | 2.79523 | 3.26864 |
| lncRNA07794 | "x" | chr02:35961398-35967753 | 5.5631 | 6.53187 |
| lncRNA37239 | "x" | chr09:67260445-67276224 | 0.640219 | 1.06137 |
| lncRNA40192 | "x" | chr10:55831366-55836837 | 4.1332 | 3.33577 |
| lncRNA01113 | "x" | chr01:420215-426321 | 3.21522 | 2.55603 |
| lncRNA20606 | "x" | chr05:20210741-20216936 | 2.18968 | 1.69073 |
| lncRNA13866 | "x" | chr03:2206117-2210847 | 0.683662 | 0.509473 |
| lncRNA18311 | "x" | chr04:4813268-4815928 | 5.77977 | 6.8674 |
| lncRNA28122 | "x" | chr07:58021167-58022058 | 9.91257 | 8.33913 |
| lncRNA14965 | "x" | chr03:49617530-49620534 | 5.63736 | 8.18818 |
| lncRNA40648 | "x" | chr10:62050069-62055930 | 4.14487 | 3.64429 |
| lncRNA18658 | "x" | chr04:25914557-25919982 | 16.8641 | 20.1687 |
| lncRNA24301 | "x" | chr06:37127111-37132449 | 7.52725 | 6.75637 |
| lncRNA25272 | "x" | chr06:1678624-1682578 | 7.08825 | 6.10068 |
| lncRNA41108 | "x" | chr11:1295170-1295937 | 31.1489 | 37.0363 |
| lncRNA35706 | "x" | chr09:1460748-1461251 | 1.38369 | 1.80288 |
| lncRNA16086 | "x" | chr04:345253-350535 | 1.62523 | 1.99786 |
| lncRNA03107 | "x" | chr01:81028898-81034087 | 1.14908 | 0.966595 |
| lncRNA35345 | "x" | chr09:65379327-65382647 | 1.17356 | 0.945561 |
| lncRNA38995 | "x" | chr10:64002632-64006740 | 19.4713 | 23.3589 |
| lncRNA44344 | "x" | chr11:50533810-50538251 | 4.75283 | 3.85241 |
| lncRNA11834 | "x" | chr03:8556667-8557992 | 22.3414 | 18.5418 |
| lncRNA11843 | "x" | chr03:8630448-8632868 | 1.77644 | 1.40668 |
| lncRNA14825 | "x" | chr03:46577091-46584432 | 1.43409 | 1.15774 |
| lncRNA33822 | "x" | chr08:62847855-62855113 | 3.42862 | 3.99606 |
| lncRNA26461 | "x" | chr06:39700221-39703137 | 18.0843 | 25.0356 |
| lncRNA39138 | "x" | chr10:646208-650932 | 10.3013 | 9.3714 |
| lncRNA24401 | "u" | chr06:38435719-38437764 | 0.769185 | 0.881153 |
| lncRNA03397 | "x" | chr01:84289856-84292557 | 23.9267 | 29.813 |
| lncRNA33523 | "x" | chr08:59218202-59235848 | 37.2747 | 32.3443 |
| lncRNA14991 | "x" | chr03:50295024-50295921 | 11.9572 | 8.81406 |
| lncRNA35242 | "x" | chr09:64208344-64217419 | 1.57277 | 1.1995 |
| lncRNA38704 | "u" | chr10:61259492-61259863 | 9.33572 | 11.1667 |
| lncRNA40941 | "x" | chr10:64540172-64543143 | 76.4827 | 95.4728 |
| lncRNA01091 | "x" | chr01:241002-245649 | 6.7157 | 9.31898 |
| lncRNA37224 | "x" | chr09:67011460-67019762 | 9.44929 | 6.683 |
| lncRNA46359 | "u" | chr12:64229169-64229808 | 4.73217 | 5.35493 |
| lncRNA22143 | "x" | chr05:10037525-10073316 | 12.7793 | 15.8246 |
| lncRNA22401 | "u" | chr05:29170738-29171125 | 49.3654 | 54.3638 |
| lncRNA36829 | "u" | chr09:62024843-62049150 | 0.99835 | 0.610497 |
| lncRNA44468 | "x" | chr11:51930685-51936424 | 11.3364 | 13.2909 |
| lncRNA25132 | "x" | chr06:45879869-45887797 | 0.876891 | 0.628152 |
| lncRNA20506 | "x" | chr05:10597443-10598938 | 10.6671 | 9.76894 |
| lncRNA46924 | "x" | chr12:4182958-4191205 | 1.66969 | 1.99117 |
| lncRNA29694 | "x" | chr07:55999418-56004983 | 3.59151 | 2.73015 |
| lncRNA35241 | "x" | chr09:64208344-64217419 | 0.647122 | 0.971243 |
| lncRNA40852 | "x" | chr10:63813409-63821534 | 6.61993 | 10.1613 |
| lncRNA45402 | "x" | chr12:21200022-21227856 | 1.12939 | 0.717091 |
| lncRNA05343 | "x" | chr01:72994132-72995987 | 3.53706 | 2.79421 |
| lncRNA42126 | "o" | chr11:36827249-36828057 | 11.1351 | 12.3262 |
| lncRNA04160 | "u" | chr01:2159289-2160607 | 3.83645 | 4.24562 |
| lncRNA12226 | "u" | chr03:27966633-27966984 | 4.09443 | 5.26128 |
| lncRNA36089 | "x" | chr09:11381641-11399733 | 0.939479 | 0.608616 |
| lncRNA13649 | "x" | chr03:64326045-64328123 | 4.49102 | 3.60211 |
| lncRNA45123 | "x" | chr12:4745672-4750635 | 3.94462 | 4.78633 |
| lncRNA46213 | "x" | chr12:63040158-63043542 | 2.18151 | 1.85037 |
| lncRNA48304 | "x" | chr12:64775509-64777201 | 2.2568 | 2.7351 |
| lncRNA35501 | "x" | chr09:67115281-67115869 | 11.5871 | 14.947 |
| lncRNA40939 | "x" | chr10:64540172-64543143 | 5.00921 | 3.50729 |
| lncRNA39284 | "x" | chr10:2104478-2107696 | 1.33638 | 1.08284 |
| lncRNA28600 | "x" | chr07:63760373-63763458 | 7.76983 | 6.30291 |
| lncRNA45195 | "x" | chr12:5686940-5695832 | 7.55885 | 8.67045 |
| lncRNA09210 | "x" | chr02:9171366-9179385 | 5.04575 | 4.15445 |
| lncRNA08956 | "x" | chr02:48380826-48386599 | 49.902 | 64.8405 |
| lncRNA14459 | "x" | chr03:24990380-24996067 | 1.92417 | 1.7769 |
| lncRNA22714 | "x" | chr05:58163572-58166944 | 15.7579 | 13.0184 |
| lncRNA30301 | "u" | chr07:63637265-63638328 | 1.54844 | 1.33203 |
| lncRNA42446 | "x" | chr11:49482461-49486050 | 32.8903 | 37.743 |
| lncRNA11066 | "u" | chr02:45565270-45567410 | 5.91245 | 5.4519 |
| lncRNA30872 | "x" | chr08:12012697-12014514 | 2.82233 | 2.16295 |
| lncRNA26914 | "j" | chr06:44656999-44661486 | 299.422 | 278.637 |
| lncRNA22255 | "x" | chr05:18466137-18469886 | 1.17882 | 1.05856 |
| lncRNA29903 | "u" | chr07:59215840-59216202 | 10.1703 | 12.0521 |
| lncRNA07269 | "x" | chr02:28070019-28072510 | 1.14412 | 0.940125 |
| lncRNA36141 | "u" | chr09:16513188-16514932 | 6.36677 | 5.85239 |
| lncRNA37896 | "x" | chr10:28141049-28144088 | 12.0824 | 10.8131 |
| lncRNA39688 | "x" | chr10:22692983-22694958 | 4.71577 | 5.15628 |
| lncRNA20064 | "x" | chr05:2106440-2110793 | 4.63869 | 3.7003 |
| lncRNA40248 | "x" | chr10:57850649-57852507 | 12.3167 | 10.8954 |
| lncRNA08619 | "x" | chr02:44892469-44892843 | 2.16825 | 2.81942 |
| lncRNA07951 | "x" | chr02:37699861-37704464 | 1.38836 | 1.08943 |
| lncRNA01282 | "x" | chr01:2357663-2360678 | 7.60549 | 9.07688 |
| lncRNA44298 | "x" | chr11:50079732-50085326 | 3.49735 | 4.19165 |
| lncRNA12945 | "x" | chr03:56740612-56742221 | 6.83027 | 7.50953 |
| lncRNA22773 | "x" | chr05:59787254-59789919 | 3.18724 | 3.81929 |
| lncRNA38937 | "x" | chr10:63600247-63603604 | 5.53993 | 4.65943 |
| lncRNA36129 | "u" | chr09:15036820-15037257 | 2.02534 | 1.58127 |
| lncRNA35124 | "x" | chr09:62428367-62432417 | 114.791 | 131.36 |
| lncRNA17734 | "x" | chr04:61960872-61965969 | 2.61031 | 2.05493 |
| lncRNA39992 | "u" | chr10:47133950-47135566 | 3.48543 | 3.83209 |
| lncRNA07438 | "x" | chr02:31384427-31385950 | 3.91781 | 5.10016 |
| lncRNA43904 | "x" | chr11:36267508-36275960 | 8.85911 | 10.6257 |
| lncRNA17569 | "x" | chr04:60136811-60140004 | 2.15536 | 2.59857 |
| lncRNA32977 | "x" | chr08:50635833-50639226 | 1.59567 | 1.94386 |
| lncRNA27409 | "x" | chr07:6515310-6518394 | 0.981665 | 1.24424 |
| lncRNA05498 | "x" | chr01:75196199-75198194 | 1.02402 | 0.797128 |
| lncRNA26829 | "x" | chr06:43763434-43767681 | 0.591948 | 0.729834 |
| lncRNA08307 | "x" | chr02:41552361-41555731 | 0.736472 | 0.952792 |
| lncRNA40534 | "x" | chr10:61005070-61007824 | 4.68894 | 3.73371 |
| lncRNA43338 | "x" | chr11:5357145-5365824 | 15.0882 | 17.5553 |
| lncRNA06403 | "x" | chr01:85768996-85775706 | 1.05771 | 1.2857 |
| lncRNA34454 | "x" | chr09:16842843-16844969 | 4.97513 | 6.62727 |
| lncRNA22949 | "x" | chr05:62466098-62470232 | 2.3019 | 1.75234 |
| lncRNA43098 | "x" | chr11:2700330-2707136 | 1.38227 | 1.74073 |
| lncRNA06874 | "x" | chr02:4366065-4366881 | 1.91339 | 2.21058 |
| lncRNA36380 | "x" | chr09:44128043-44131083 | 2.41426 | 2.21101 |
| lncRNA22824 | "x" | chr05:60637188-60647088 | 2.53491 | 1.94483 |
| lncRNA34671 | "x" | chr09:43665437-43678768 | 0.956387 | 1.18516 |
| lncRNA26517 | "x" | chr06:40430999-40433125 | 1.24928 | 1.57009 |
| lncRNA18089 | "x" | chr04:2012437-2014971 | 11.2287 | 8.47637 |
| lncRNA34652 | "x" | chr09:42251199-42261835 | 0.558536 | 0.609079 |
| lncRNA22202 | "u" | chr05:13935528-13936519 | 0.881618 | 1.04918 |
| lncRNA01121 | "x" | chr01:477866-480386 | 33.2524 | 29.4497 |
| lncRNA11857 | "x" | chr03:8893580-8895465 | 1.96594 | 1.59482 |
| lncRNA26049 | "x" | chr06:34944044-34947287 | 0.786555 | 0.702174 |
| lncRNA18225 | "x" | chr04:3595815-3596666 | 0.874226 | 0.714023 |
| lncRNA34379 | "u" | chr09:11054920-11057500 | 11.8675 | 12.7301 |
| lncRNA18743 | "x" | chr04:32819447-32828520 | 2.02181 | 1.75218 |
| lncRNA03308 | "x" | chr01:83279473-83284682 | 1.57703 | 2.08507 |
| lncRNA24314 | "x" | chr06:37261408-37270075 | 4.7382 | 4.29462 |
| lncRNA14644 | "x" | chr03:41236975-41237761 | 56.3256 | 48.4689 |
| lncRNA20698 | "u" | chr05:28060780-28074769 | 32.3284 | 21.8643 |
| lncRNA44386 | "x" | chr11:51054006-51059536 | 22.8896 | 26.4553 |
| lncRNA22012 | "x" | chr05:6718425-6723728 | 3.79899 | 2.97977 |
| lncRNA27718 | "x" | chr07:42662204-42667542 | 2.04805 | 2.40868 |
| lncRNA35852 | "u" | chr09:3668781-3672749 | 7.90296 | 8.46888 |
| lncRNA40788 | "x" | chr10:63236087-63238063 | 9.06325 | 7.75068 |
| lncRNA40372 | "x" | chr10:59546047-59551523 | 4.48552 | 5.4953 |
| lncRNA06406 | "u" | chr01:85799177-85800547 | 0.740935 | 0.877328 |
| lncRNA22704 | "x" | chr05:57979169-57979768 | 19.0806 | 20.9771 |
| lncRNA30441 | "u" | chr07:65096101-65096889 | 1.24251 | 1.46874 |
| lncRNA32193 | "u" | chr08:624802-625481 | 1.6343 | 1.93635 |
| lncRNA16141 | "x" | chr04:1065838-1072643 | 2.26939 | 2.70784 |
| lncRNA14126 | "u" | chr03:9184028-9184614 | 1.52906 | 1.24169 |
| lncRNA46219 | "x" | chr12:63107292-63112866 | 5.5367 | 4.73768 |
| lncRNA38168 | "x" | chr10:47332776-47335943 | 0.70253 | 0.851042 |
| lncRNA03480 | "u" | chr01:84988957-84989458 | 2.22697 | 1.81783 |
| lncRNA18288 | "x" | chr04:4541496-4544361 | 1.38201 | 1.78497 |
| lncRNA16009 | "u" | chr03:64212198-64212528 | 50.1447 | 44.4402 |
| lncRNA10377 | "x" | chr02:38449862-38458570 | 2.28193 | 3.16723 |
| lncRNA09090 | "x" | chr02:49731299-49733909 | 0.88299 | 1.07264 |
| lncRNA15589 | "x" | chr03:59695831-59701517 | 1.44906 | 1.02023 |
| lncRNA38842 | "x" | chr10:62693485-62705391 | 9.18348 | 7.67928 |
| lncRNA25408 | "o" | chr06:3492430-3495217 | 1.6301 | 1.39053 |
| lncRNA14190 | "x" | chr03:9966348-9968429 | 2.8914 | 2.47869 |
| lncRNA32896 | "x" | chr08:47962816-47973284 | 3.83575 | 2.90145 |
| lncRNA20011 | "x" | chr05:1214285-1219211 | 0.872778 | 0.665945 |
| lncRNA28675 | "x" | chr07:64455533-64463398 | 4.67643 | 3.779 |
| lncRNA37299 | "x" | chr10:482054-489351 | 1.89535 | 1.49849 |
| lncRNA27104 | "x" | chr07:586533-588407 | 3.87297 | 3.45581 |
| lncRNA44648 | "x" | chr12:131154-136091 | 0.673056 | 0.555283 |
| lncRNA07731 | "x" | chr02:35217703-35225700 | 2.75376 | 2.28091 |
| lncRNA27838 | "u" | chr07:52215459-52219908 | 4.70616 | 4.27843 |
| lncRNA08761 | "x" | chr02:46255508-46256124 | 83.3902 | 60.9194 |
| lncRNA44812 | "x" | chr12:1537179-1541140 | 16.2765 | 13.4063 |
| lncRNA14153 | "x" | chr03:9500783-9507893 | 10.2931 | 12.4495 |
| lncRNA17885 | "x" | chr04:63835557-63843020 | 3.53223 | 2.71492 |
| lncRNA39625 | "x" | chr10:17993427-17995316 | 28.225 | 22.9095 |
| lncRNA21462 | "x" | chr05:64293195-64296760 | 2.66694 | 1.88231 |
| lncRNA22991 | "x" | chr05:63100529-63103607 | 3.12089 | 4.17068 |
| lncRNA42642 | "x" | chr11:51669431-51673400 | 1.19546 | 0.925582 |
| lncRNA28001 | "x" | chr07:55699387-55703727 | 5.91332 | 5.51639 |
| lncRNA16646 | "u" | chr04:13495632-13495860 | 39.6241 | 50.9096 |
| lncRNA20701 | "u" | chr05:28084355-28084680 | 4.45801 | 5.71519 |
| lncRNA42740 | "x" | chr11:52554793-52557686 | 3.08783 | 3.67981 |
| lncRNA39875 | "x" | chr10:41753578-41755101 | 29.616 | 35.9487 |
| lncRNA33937 | "x" | chr09:964900-970853 | 1.27057 | 1.48692 |
| lncRNA02964 | "x" | chr01:79604630-79610949 | 1.7264 | 2.09816 |
| lncRNA19984 | "x" | chr05:814492-815389 | 14.9218 | 17.5107 |
| lncRNA24525 | "u" | chr06:39753815-39754156 | 13.5138 | 15.9149 |
| lncRNA33753 | "x" | chr08:62016441-62021205 | 2.19147 | 1.54126 |
| lncRNA30730 | "u" | chr08:3361334-3361992 | 9.28088 | 10.227 |
| lncRNA20507 | "u" | chr05:10685259-10687877 | 3.50128 | 3.2349 |
| lncRNA41794 | "o" | chr11:13927801-13931184 | 32.0964 | 29.0753 |
| lncRNA48179 | "x" | chr12:63715795-63718275 | 2.57814 | 2.97127 |
| lncRNA42402 | "x" | chr11:48838044-48839089 | 36.4974 | 47.1245 |
| lncRNA34002 | "x" | chr09:1612471-1615497 | 3.89235 | 4.65885 |
| lncRNA38401 | "x" | chr10:57945111-57956476 | 10.4723 | 9.35626 |
| lncRNA14258 | "u" | chr03:11824292-11825840 | 0.75752 | 0.656248 |
| lncRNA14464 | "u" | chr03:25208577-25209074 | 5.37348 | 4.64136 |
| lncRNA18012 | "u" | chr04:989519-990315 | 1.99384 | 1.72348 |
| lncRNA11188 | "x" | chr02:46898347-46906437 | 6.42323 | 7.77877 |
| lncRNA09630 | "x" | chr02:29072684-29074553 | 12.1872 | 13.2537 |
| lncRNA02417 | "x" | chr01:72686530-72695319 | 6.56184 | 7.60132 |
| lncRNA45796 | "u" | chr12:44972328-44973373 | 1.40883 | 1.15695 |
| lncRNA46890 | "x" | chr12:3807526-3810933 | 4.81514 | 5.83294 |
| lncRNA13262 | "x" | chr03:60309684-60310623 | 0.963455 | 1.14782 |
| lncRNA31347 | "x" | chr08:52455965-52463301 | 4.71994 | 5.35509 |
| lncRNA32715 | "u" | chr08:30707980-30710376 | 1.61301 | 1.77288 |
| lncRNA21854 | "x" | chr05:4453321-4454407 | 21.6454 | 18.4923 |
| lncRNA12609 | "x" | chr03:48931859-48932684 | 398.554 | 508.803 |
| lncRNA13752 | "x" | chr03:590057-595854 | 1.06169 | 0.843152 |
| lncRNA47012 | "x" | chr12:5441220-5446999 | 25.6778 | 34.9092 |
| lncRNA08915 | "u" | chr02:47948479-47950574 | 21.0508 | 22.7318 |
| lncRNA29855 | "x" | chr07:58532610-58534185 | 11.1253 | 9.56846 |
| lncRNA28661 | "x" | chr07:64364297-64368103 | 1.71418 | 1.47886 |
| lncRNA34936 | "x" | chr09:59228853-59238380 | 1.23623 | 1.85012 |
| lncRNA25581 | "x" | chr06:20648200-20654157 | 0.718181 | 0.877955 |
| lncRNA02247 | "x" | chr01:69651824-69658418 | 0.849192 | 1.09766 |
| lncRNA43241 | "x" | chr11:4461422-4472459 | 1.03238 | 0.762161 |
| lncRNA47509 | "x" | chr12:37959034-37959856 | 6.09852 | 6.75663 |
| lncRNA46265 | "x" | chr12:63527616-63533470 | 9.72756 | 12.4515 |
| lncRNA36796 | "x" | chr09:61524969-61527952 | 0.877816 | 0.709746 |
| lncRNA47963 | "x" | chr12:59474711-59480399 | 10.2718 | 12.0281 |
| lncRNA28636 | "x" | chr07:64080740-64083209 | 0.873931 | 1.13054 |
| lncRNA33645 | "x" | chr08:60845203-60847982 | 11.8734 | 10.5419 |
| lncRNA27999 | "x" | chr07:55658107-55669122 | 15.7105 | 19.4195 |
| lncRNA40970 | "x" | chr10:64798023-64800186 | 3.38689 | 2.88213 |
| lncRNA43649 | "x" | chr11:17049554-17054156 | 12.0589 | 10.0302 |
| lncRNA07924 | "u" | chr02:37471491-37473110 | 1.94619 | 1.74162 |
| lncRNA29092 | "x" | chr07:5625014-5634447 | 5.77808 | 4.7089 |
| lncRNA40571 | "u" | chr10:61333309-61336909 | 2.69162 | 2.48679 |
| lncRNA14179 | "x" | chr03:9732697-9739046 | 0.701111 | 0.609845 |
| lncRNA36144 | "x" | chr09:16517926-16519922 | 1.48509 | 1.95911 |
| lncRNA42831 | "x" | chr11:147567-156637 | 16.6691 | 14.3123 |
| lncRNA15822 | "x" | chr03:62238371-62239920 | 37.0798 | 47.4857 |
| lncRNA27790 | "x" | chr07:49305576-49311893 | 1.01752 | 0.817885 |
| lncRNA35592 | "x" | chr09:253407-269376 | 2.367 | 1.75578 |
| lncRNA46635 | "u" | chr12:1097707-1098325 | 6.80483 | 6.07661 |
| lncRNA08533 | "x" | chr02:44045071-44057225 | 7.80094 | 9.4105 |
| lncRNA20440 | "x" | chr05:8115884-8118432 | 7.06144 | 8.3566 |
| lncRNA36831 | "x" | chr09:62024843-62049150 | 4.19751 | 2.62828 |
| lncRNA27055 | "x" | chr06:45950565-45959677 | 3.24487 | 2.77538 |
| lncRNA47870 | "x" | chr12:48542023-48543957 | 32.4627 | 25.0577 |
| lncRNA17673 | "x" | chr04:61268367-61273656 | 4.9038 | 3.41648 |
| lncRNA17359 | "x" | chr04:57222953-57227125 | 56.2876 | 45.9367 |
| lncRNA26109 | "x" | chr06:35698536-35703436 | 0.942799 | 0.813755 |
| lncRNA35464 | "x" | chr09:66826616-66829521 | 3.92579 | 4.49357 |
| lncRNA13440 | "x" | chr03:62122427-62126103 | 8.85319 | 10.1818 |
| lncRNA04866 | "x" | chr01:57853718-57859489 | 1.2488 | 1.65394 |
| lncRNA25374 | "x" | chr06:2965667-2967884 | 12.9734 | 11.1986 |
| lncRNA16062 | "x" | chr04:71632-77723 | 0.652716 | 0.904574 |
| lncRNA37184 | "x" | chr09:66645387-66647714 | 1.8329 | 1.53958 |
| lncRNA36059 | "u" | chr09:8780509-8783093 | 2.17325 | 2.37389 |
| lncRNA43165 | "x" | chr11:3380695-3387891 | 10.2262 | 8.30861 |
| lncRNA11081 | "x" | chr02:45792080-45808025 | 14.9223 | 20.9583 |
| lncRNA16250 | "x" | chr04:2511228-2514610 | 18.1909 | 13.4987 |
| lncRNA21518 | "x" | chr05:64926414-64939085 | 8.13384 | 6.74896 |
| lncRNA38507 | "u" | chr10:59467083-59467907 | 1.12857 | 1.32654 |
| lncRNA22133 | "x" | chr05:9724619-9743820 | 9.64928 | 12.5081 |
| lncRNA30999 | "x" | chr08:30537368-30544347 | 3.55309 | 4.30476 |
| lncRNA19692 | "x" | chr04:61896305-61898185 | 8.79334 | 11.525 |
| lncRNA41292 | "x" | chr11:3181785-3190461 | 33.8725 | 37.4073 |
| lncRNA39012 | "x" | chr10:64123014-64129787 | 2.62064 | 2.12301 |
| lncRNA45184 | "x" | chr12:5599855-5603988 | 1.3556 | 1.6861 |
| lncRNA17850 | "x" | chr04:63373842-63378319 | 1.11438 | 0.938968 |
| lncRNA26121 | "x" | chr06:35809188-35813216 | 3.54365 | 3.09925 |
| lncRNA30649 | "x" | chr08:2251357-2271409 | 2.04261 | 2.28292 |
| lncRNA12921 | "x" | chr03:56519078-56524988 | 3.37426 | 4.13248 |
| lncRNA39373 | "x" | chr10:3406751-3409405 | 17.8759 | 16.1544 |
| lncRNA03656 | "x" | chr01:86763244-86767272 | 6.99431 | 6.30327 |
| lncRNA33938 | "x" | chr09:964900-970853 | 1.15807 | 0.949352 |
| lncRNA44724 | "x" | chr12:712641-715942 | 42.1172 | 35.4156 |
| lncRNA40984 | "x" | chr11:69258-73275 | 10.0109 | 8.43758 |
| lncRNA11295 | "x" | chr02:48020173-48023471 | 2.20569 | 1.89973 |
| lncRNA33230 | "x" | chr08:55300248-55316472 | 9.14598 | 11.4662 |
| lncRNA02210 | "x" | chr01:68976195-68994801 | 1.09994 | 0.826526 |
| lncRNA40467 | "x" | chr10:60349061-60350855 | 5.99959 | 8.30273 |
| lncRNA26267 | "u" | chr06:37556536-37556765 | 312.04 | 275.799 |
| lncRNA00492 | "x" | chr00:20647169-20649279 | 11.0417 | 12.8824 |
| lncRNA29080 | "x" | chr07:5197541-5201173 | 2.97009 | 2.48577 |
| lncRNA43486 | "x" | chr11:8721427-8727227 | 5.27488 | 6.26467 |
| lncRNA03572 | "u" | chr01:85878926-85891837 | 2.85801 | 1.90435 |
| lncRNA13783 | "x" | chr03:909633-914360 | 2.15052 | 2.40433 |
| lncRNA14005 | "x" | chr03:7634617-7636770 | 0.697688 | 0.905452 |
| lncRNA23303 | "x" | chr06:1748100-1752394 | 5.06916 | 4.40019 |
| lncRNA26755 | "x" | chr06:43050785-43051546 | 38.3078 | 47.6709 |
| lncRNA45144 | "x" | chr12:4931011-4933969 | 5.97753 | 7.79459 |
| lncRNA24514 | "x" | chr06:39675107-39681903 | 3.29381 | 3.67857 |
| lncRNA40416 | "x" | chr10:59880693-59883090 | 7.59124 | 6.38342 |
| lncRNA02533 | "x" | chr01:74108627-74114909 | 2.71278 | 3.00439 |
| lncRNA36949 | "x" | chr09:63534871-63547345 | 1.20662 | 0.994575 |
| lncRNA31025 | "x" | chr08:36781140-36786963 | 7.65888 | 6.56916 |
| lncRNA12799 | "x" | chr03:53530484-53532501 | 16.6471 | 13.2839 |
| lncRNA30637 | "x" | chr08:2125106-2129164 | 2.4063 | 1.83562 |
| lncRNA36477 | "u" | chr09:50318530-50320335 | 9.53285 | 8.90221 |
| lncRNA07730 | "x" | chr02:35206515-35213915 | 7.7105 | 7.48222 |
| lncRNA41460 | "x" | chr11:5135778-5138781 | 4.78049 | 6.77841 |
| lncRNA01940 | "x" | chr01:53410401-53419907 | 2.17138 | 2.59093 |
| lncRNA38506 | "x" | chr10:59464366-59465746 | 9.1143 | 7.74889 |
| lncRNA06444 | "u" | chr01:86215909-86217583 | 1.44406 | 1.61199 |
| lncRNA15083 | "x" | chr03:52909980-52914569 | 5.63083 | 7.17504 |
| lncRNA06976 | "x" | chr02:13103670-13107547 | 1.19719 | 1.09386 |
| lncRNA46253 | "x" | chr12:63432450-63438226 | 2.89338 | 3.78907 |
| lncRNA11227 | "x" | chr02:47258560-47259331 | 5.48304 | 4.15327 |
| lncRNA12483 | "x" | chr03:46244595-46250538 | 5.40085 | 4.3655 |
| lncRNA42349 | "x" | chr11:47939418-47944116 | 4.82717 | 4.5147 |
| lncRNA43373 | "x" | chr11:5872662-5874436 | 1.77368 | 2.1952 |
| lncRNA16794 | "x" | chr04:30736507-30739594 | 4.89838 | 4.18089 |
| lncRNA28004 | "x" | chr07:55724939-55728323 | 5.78728 | 4.83529 |
| lncRNA05771 | "x" | chr01:78658132-78669039 | 2.07862 | 1.72388 |
| lncRNA23821 | "x" | chr06:29366137-29368048 | 19.245 | 14.858 |
| lncRNA45016 | "x" | chr12:3471049-3475830 | 22.6381 | 33.8613 |
| lncRNA20166 | "x" | chr05:3208285-3211740 | 3.4431 | 2.90232 |
| lncRNA27793 | "u" | chr07:49494164-49495676 | 2.9838 | 3.26525 |
| lncRNA42508 | "x" | chr11:50223483-50227393 | 0.98385 | 0.76333 |
| lncRNA45865 | "u" | chr12:46647446-46647667 | 197.904 | 169.264 |
| lncRNA46984 | "x" | chr12:4918491-4923579 | 4.01865 | 3.38775 |
| lncRNA42814 | "x" | chr11:53376248-53382033 | 1.90787 | 2.30745 |
| lncRNA42529 | "u" | chr11:50399599-50401208 | 4.68076 | 4.30526 |
| lncRNA44545 | "x" | chr11:52658480-52664137 | 13.9673 | 11.1207 |
| lncRNA26513 | "x" | chr06:40335708-40344949 | 11.1402 | 9.11831 |
| lncRNA39707 | "x" | chr10:24529173-24538113 | 13.1321 | 16.149 |
| lncRNA26805 | "x" | chr06:43486754-43490197 | 1.83258 | 2.47398 |
| lncRNA33609 | "x" | chr08:60327091-60332049 | 5.08979 | 5.80727 |
| lncRNA08766 | "x" | chr02:46288184-46290290 | 1.83184 | 1.66585 |
| lncRNA40781 | "x" | chr10:63196189-63199261 | 3.71523 | 4.68683 |
| lncRNA42449 | "x" | chr11:49512561-49521520 | 15.8679 | 14.4015 |
| lncRNA31777 | "x" | chr08:59236251-59238091 | 8.36026 | 6.72418 |
| lncRNA44488 | "x" | chr11:52183259-52204381 | 5.97514 | 4.79057 |
| lncRNA34728 | "x" | chr09:49797126-49800218 | 5.3509 | 5.72832 |
| lncRNA34922 | "x" | chr09:59066134-59066793 | 6.07683 | 4.39815 |
| lncRNA03647 | "u" | chr01:86624119-86624464 | 9.77875 | 11.5263 |
| lncRNA21702 | "x" | chr05:2281080-2283542 | 6.26889 | 5.24803 |
| lncRNA36984 | "u" | chr09:64000941-64002524 | 4.635 | 5.03427 |
| lncRNA45062 | "x" | chr12:3970953-3974056 | 4.31064 | 3.38001 |
| lncRNA15748 | "u" | chr03:61431614-61435542 | 5.85337 | 6.24101 |
| lncRNA22437 | "u" | chr05:30892562-30903761 | 0.495756 | 0.71637 |
| lncRNA10963 | "x" | chr02:44652276-44654950 | 2.63008 | 3.66996 |
| lncRNA05743 | "x" | chr01:78322389-78325890 | 7.81816 | 7.16861 |
| lncRNA25875 | "x" | chr06:32066257-32066685 | 1.39166 | 1.20541 |
| lncRNA47269 | "x" | chr12:19964774-19965146 | 11.8874 | 10.2871 |
| lncRNA08436 | "x" | chr02:42949518-42961984 | 31.8201 | 36.6702 |
| lncRNA39761 | "u" | chr10:32355015-32356051 | 2.33685 | 2.08615 |
| lncRNA40465 | "x" | chr10:60309914-60318533 | 14.6453 | 17.0829 |
| lncRNA02669 | "x" | chr01:75848646-75866839 | 14.711 | 13.0848 |
| lncRNA42773 | "x" | chr11:52950434-52955286 | 1.04661 | 0.813492 |
| lncRNA17495 | "x" | chr04:59214455-59217833 | 6.03582 | 6.43452 |
| lncRNA46729 | "x" | chr12:2134018-2140549 | 2.33104 | 2.1343 |
| lncRNA28097 | "x" | chr07:57380030-57384945 | 0.462762 | 0.63367 |
| lncRNA31198 | "x" | chr08:48317884-48318642 | 1.3287 | 1.14782 |
| lncRNA09910 | "x" | chr02:33172763-33180223 | 2.77288 | 2.496 |
| lncRNA26062 | "u" | chr06:35093239-35093937 | 3.0357 | 3.41533 |
| lncRNA33855 | "x" | chr09:175106-180103 | 1.53892 | 1.27133 |
| lncRNA45838 | "x" | chr12:46073128-46073982 | 11.2447 | 14.5668 |
| lncRNA43712 | "x" | chr11:21504135-21524244 | 0.784242 | 0.684585 |
| lncRNA15653 | "x" | chr03:60457108-60459160 | 22.891 | 21.3284 |
| lncRNA13076 | "u" | chr03:58245875-58248612 | 1.88718 | 1.72875 |
| lncRNA15170 | "x" | chr03:54032355-54043045 | 3.52329 | 2.86326 |
| lncRNA23807 | "x" | chr06:28975747-28981918 | 1.93374 | 2.33803 |
| lncRNA06184 | "o" | chr01:83252022-83258088 | 1.89847 | 1.57801 |
| lncRNA30075 | "u" | chr07:61034688-61035563 | 5.40711 | 5.93086 |
| lncRNA23055 | "x" | chr05:63884548-63891458 | 3.66001 | 2.97576 |
| lncRNA33649 | "x" | chr08:60871158-60878316 | 3.05389 | 3.60355 |
| lncRNA46129 | "x" | chr12:62198316-62200434 | 2.01541 | 2.46508 |
| lncRNA10820 | "x" | chr02:43115436-43123905 | 2.19178 | 1.94126 |
| lncRNA46571 | "x" | chr12:428786-434597 | 5.66599 | 6.39281 |
| lncRNA33065 | "x" | chr08:52806346-52807980 | 0.655958 | 0.848154 |
| lncRNA01861 | "u" | chr01:46770738-46777003 | 1.24711 | 1.15257 |
| lncRNA39652 | "x" | chr10:20931188-20937092 | 6.84159 | 8.49307 |
| lncRNA47506 | "u" | chr12:37901514-37902267 | 14.2137 | 13.1295 |
| lncRNA24512 | "x" | chr06:39647266-39651761 | 0.828028 | 0.984269 |
| lncRNA44279 | "x" | chr11:49886800-49894730 | 8.7494 | 6.90913 |
| lncRNA48284 | "x" | chr12:64645084-64648815 | 3.10048 | 3.7363 |
| lncRNA19784 | "u" | chr04:62873452-62874833 | 2.07454 | 1.8713 |
| lncRNA06509 | "x" | chr01:86915523-86922302 | 27.9054 | 30.6734 |
| lncRNA13109 | "x" | chr03:58636756-58640560 | 0.803273 | 1.07388 |
| lncRNA35521 | "x" | chr09:67260445-67276224 | 13.0065 | 11.791 |
| lncRNA17227 | "x" | chr04:55013507-55018537 | 31.5945 | 23.7395 |
| lncRNA44287 | "x" | chr11:49978028-49981767 | 25.5245 | 20.203 |
| lncRNA15020 | "x" | chr03:51376490-51382146 | 11.8771 | 10.6578 |
| lncRNA27928 | "u" | chr07:54254505-54255007 | 24.771 | 26.9614 |
| lncRNA08807 | "x" | chr02:46801720-46805514 | 1.18609 | 1.42678 |
| lncRNA19732 | "x" | chr04:62236179-62240094 | 0.715212 | 0.588728 |
| lncRNA35546 | "x" | chr09:67491032-67496208 | 2.17723 | 2.81315 |
| lncRNA09271 | "u" | chr02:13648541-13650891 | 0.732054 | 0.818312 |
| lncRNA04020 | "x" | chr01:642517-648118 | 10.2389 | 9.22999 |
| lncRNA04182 | "x" | chr01:2385891-2404877 | 14.5939 | 16.9619 |
| lncRNA47950 | "u" | chr12:57400054-57400719 | 2.31893 | 2.00384 |
| lncRNA35698 | "u" | chr09:1399528-1399831 | 54.2208 | 47.84 |
| lncRNA47043 | "x" | chr12:5811999-5817521 | 13.3069 | 16.5864 |
| lncRNA23322 | "x" | chr06:2042759-2050873 | 1.4955 | 1.21929 |
| lncRNA31379 | "x" | chr08:53185862-53191168 | 5.56806 | 6.10408 |
| lncRNA11535 | "x" | chr03:628730-631838 | 0.567731 | 0.731089 |
| lncRNA20134 | "x" | chr05:2893044-2899996 | 1.05561 | 0.874136 |
| lncRNA28969 | "x" | chr07:2660219-2667792 | 1.70811 | 1.29462 |
| lncRNA35611 | "x" | chr09:437632-442917 | 1.17807 | 0.971316 |
| lncRNA09708 | "x" | chr02:30419777-30425448 | 6.165 | 6.88617 |
| lncRNA36196 | "u" | chr09:21022259-21057350 | 0.974885 | 0.774917 |
| lncRNA16188 | "x" | chr04:1682463-1697247 | 1.14282 | 0.955954 |
| lncRNA17752 | "x" | chr04:62208524-62213336 | 0.712228 | 0.570593 |
| lncRNA39817 | "x" | chr10:37072999-37077766 | 4.6946 | 6.43128 |
| lncRNA13535 | "x" | chr03:63078677-63079354 | 7.27474 | 5.72907 |
| lncRNA47800 | "x" | chr12:47466155-47469512 | 5.46892 | 6.42922 |
| lncRNA19011 | "x" | chr04:50976968-50977970 | 0.742852 | 0.647927 |
| lncRNA30698 | "x" | chr08:2920288-2922665 | 0.846993 | 0.678019 |
| lncRNA31872 | "x" | chr08:60166482-60170216 | 36.1461 | 41.518 |
| lncRNA30116 | "x" | chr07:61564272-61568561 | 11.4816 | 9.80298 |
| lncRNA30236 | "x" | chr07:62940487-62946443 | 0.732964 | 0.907496 |
| lncRNA39080 | "x" | chr10:64782861-64789180 | 3.64123 | 4.36866 |
| lncRNA28605 | "x" | chr07:63833708-63836015 | 1.24211 | 1.40617 |
| lncRNA44857 | "x" | chr12:1974429-1979725 | 17.3919 | 16.0439 |
| lncRNA18037 | "x" | chr04:1283913-1287795 | 10.048 | 13.2176 |
| lncRNA48205 | "x" | chr12:63929226-63931184 | 1.26241 | 1.65071 |
| lncRNA03181 | "x" | chr01:81801836-81804990 | 13.0337 | 15.7044 |
| lncRNA14694 | "u" | chr03:43732578-43739980 | 2.03704 | 2.22279 |
| lncRNA06105 | "x" | chr01:82378815-82399452 | 12.4477 | 9.75714 |
| lncRNA24160 | "x" | chr06:35475233-35482560 | 77.8561 | 83.1352 |
| lncRNA10921 | "x" | chr02:44301839-44304082 | 0.726929 | 0.945549 |
| lncRNA17570 | "x" | chr04:60142815-60148275 | 17.5551 | 13.9108 |
| lncRNA39634 | "u" | chr10:19037138-19040239 | 8.75093 | 9.31267 |
| lncRNA05651 | "x" | chr01:77112342-77117902 | 8.75786 | 9.85081 |
| lncRNA11156 | "x" | chr02:46579721-46580497 | 1.83839 | 1.50083 |
| lncRNA16265 | "x" | chr04:2618406-2627468 | 11.2885 | 9.8238 |
| lncRNA44540 | "u" | chr11:52629542-52629860 | 55.6981 | 61.7138 |
| lncRNA28451 | "x" | chr07:62208937-62213598 | 10.2919 | 14.1875 |
| lncRNA44145 | "x" | chr11:47743983-47745573 | 21.692 | 25.2438 |
| lncRNA33628 | "x" | chr08:60651001-60652375 | 15.7945 | 10.7728 |
| lncRNA03242 | "x" | chr01:82514404-82519439 | 0.78643 | 0.947035 |
| lncRNA06496 | "u" | chr01:86805164-86805790 | 10.2313 | 12.403 |
| lncRNA07529 | "x" | chr02:32649783-32651779 | 1.15662 | 1.37072 |
| lncRNA12665 | "x" | chr03:50349407-50352048 | 0.780652 | 0.607094 |
| lncRNA09862 | "x" | chr02:32643741-32648213 | 2.03052 | 1.79996 |
| lncRNA43890 | "x" | chr11:35859362-35861393 | 1.5479 | 1.17132 |
| lncRNA39007 | "x" | chr10:64091670-64094291 | 2.81906 | 2.27847 |
| lncRNA19314 | "u" | chr04:57154878-57157897 | 2.45152 | 2.23949 |
| lncRNA36329 | "x" | chr09:37798401-37814494 | 9.13238 | 7.69824 |
| lncRNA36436 | "x" | chr09:48306436-48308072 | 6.27817 | 7.28357 |
| lncRNA24944 | "x" | chr06:43971026-43974405 | 17.5274 | 19.8465 |
| lncRNA09308 | "x" | chr02:15496663-15497253 | 13.6913 | 10.7155 |
| lncRNA05387 | "x" | chr01:73577958-73585260 | 1.11282 | 0.926276 |
| lncRNA32204 | "x" | chr08:749598-755094 | 6.8905 | 7.63029 |
| lncRNA44257 | "x" | chr11:49725869-49731382 | 24.2416 | 22.2971 |
| lncRNA29393 | "x" | chr07:37921416-37924651 | 2.32246 | 2.50447 |
| lncRNA08974 | "x" | chr02:48550848-48557510 | 9.1988 | 8.29541 |
| lncRNA18803 | "u" | chr04:39845900-39846388 | 5.98904 | 5.27625 |
| lncRNA46373 | "x" | chr12:64335039-64335699 | 12.7702 | 10.4845 |
| lncRNA09195 | "x" | chr02:7820671-7827566 | 7.19731 | 6.08108 |
| lncRNA37270 | "x" | chr09:67638252-67650510 | 5.18831 | 4.21001 |
| lncRNA23670 | "x" | chr06:21801050-21802904 | 1.80736 | 1.65043 |
| lncRNA13046 | "x" | chr03:57875337-57880089 | 1.69591 | 1.42815 |
| lncRNA00238 | "u" | chr00:12632396-12635202 | 1.28711 | 1.44808 |
| lncRNA40985 | "x" | chr11:91924-105206 | 12.7213 | 14.4987 |
| lncRNA37251 | "u" | chr09:67418886-67419369 | 7.48648 | 6.62663 |
| lncRNA00398 | "x" | chr00:16688347-16690038 | 4.65374 | 4.26402 |
| lncRNA15192 | "x" | chr03:54371524-54378426 | 2.14127 | 1.85467 |
| lncRNA07780 | "x" | chr02:35875448-35876126 | 2.67392 | 3.04272 |
| lncRNA22778 | "u" | chr05:59862614-59864962 | 0.709662 | 0.633011 |
| lncRNA37515 | "x" | chr10:2859727-2865652 | 1.16154 | 0.992052 |
| lncRNA46695 | "x" | chr12:1799024-1803857 | 10.7447 | 12.8115 |
| lncRNA44553 | "x" | chr11:52713557-52728072 | 2.70043 | 2.2158 |
| lncRNA46343 | "x" | chr12:64120809-64145028 | 17.9855 | 22.8846 |
| lncRNA02637 | "x" | chr01:75364499-75365497 | 2.66595 | 3.43019 |
| lncRNA26619 | "x" | chr06:41386284-41388620 | 2.76217 | 2.41727 |
| lncRNA14346 | "u" | chr03:16112595-16116855 | 2.252 | 2.09925 |
| lncRNA23333 | "x" | chr06:2204253-2213239 | 0.76437 | 0.610429 |
| lncRNA24051 | "x" | chr06:33725467-33726480 | 2.56771 | 2.05949 |
| lncRNA36181 | "x" | chr09:19509016-19518675 | 3.35636 | 3.5618 |
| lncRNA14114 | "x" | chr03:8924939-8927974 | 15.5651 | 14.5302 |
| lncRNA45139 | "x" | chr12:4893798-4896737 | 12.3009 | 15.1841 |
| lncRNA21576 | "x" | chr05:665863-672056 | 1.07129 | 0.907755 |
| lncRNA27829 | "u" | chr07:51498330-51502159 | 1.08704 | 0.984648 |
| lncRNA40281 | "x" | chr10:58210794-58211355 | 761.003 | 910.965 |
| lncRNA40537 | "x" | chr10:61024274-61027540 | 37.4457 | 31.7046 |
| lncRNA07615 | "x" | chr02:33823777-33827793 | 1.09697 | 0.945384 |
| lncRNA15444 | "x" | chr03:58358467-58361072 | 3.23469 | 3.61409 |
| lncRNA24656 | "x" | chr06:41032665-41034660 | 3.71337 | 3.04693 |
| lncRNA45051 | "x" | chr12:3792735-3796034 | 0.650964 | 0.78126 |
| lncRNA02510 | "x" | chr01:73779681-73780394 | 12.3786 | 15.0376 |
| lncRNA06507 | "x" | chr01:86905656-86914166 | 4.20613 | 4.57117 |
| lncRNA40446 | "x" | chr10:60140567-60143266 | 2.60653 | 2.16758 |
| lncRNA47797 | "x" | chr12:47434717-47441368 | 0.777306 | 0.939383 |
| lncRNA06068 | "x" | chr01:82009420-82017397 | 0.870664 | 0.633509 |
| lncRNA18762 | "u" | chr04:35108877-35110149 | 30.1122 | 28.3756 |
| lncRNA16005 | "x" | chr03:64181625-64184025 | 26.887 | 20.788 |
| lncRNA45176 | "x" | chr12:5447835-5455709 | 1.5854 | 1.15557 |
| lncRNA46744 | "x" | chr12:2291561-2297088 | 0.651953 | 0.845409 |
| lncRNA02956 | "u" | chr01:79485096-79485470 | 31.5315 | 34.6265 |
| lncRNA44931 | "x" | chr12:2726503-2729795 | 22.2174 | 17.9733 |
| lncRNA30364 | "x" | chr07:64301710-64304910 | 192.104 | 211.279 |
| lncRNA40570 | "x" | chr10:61327017-61332526 | 5.19728 | 6.07933 |
| lncRNA08699 | "u" | chr02:45633302-45634059 | 5.14764 | 5.65187 |
| lncRNA46046 | "x" | chr12:54592267-54595142 | 0.736866 | 0.600549 |
| lncRNA25065 | "x" | chr06:45132078-45133391 | 6.693 | 5.37414 |
| lncRNA41643 | "x" | chr11:8383114-8384626 | 2.49596 | 2.82905 |
| lncRNA13037 | "x" | chr03:57759331-57764210 | 51.0957 | 59.7912 |
| lncRNA45594 | "u" | chr12:36375550-36375904 | 12.4396 | 10.8569 |
| lncRNA09385 | "x" | chr02:18822313-18837732 | 1.34295 | 1.12415 |
| lncRNA43934 | "x" | chr11:36943234-36953509 | 0.649838 | 0.788382 |
| lncRNA30400 | "u" | chr07:64625742-64626503 | 3.18448 | 3.54764 |
| lncRNA30414 | "x" | chr07:64812286-64813068 | 59.8365 | 46.3866 |
| lncRNA14399 | "u" | chr03:19835585-19850837 | 0.983809 | 1.28131 |
| lncRNA24350 | "x" | chr06:37749766-37753662 | 16.2608 | 21.0016 |
| lncRNA25949 | "x" | chr06:33310349-33315158 | 9.72428 | 10.9551 |
| lncRNA46167 | "x" | chr12:62515509-62516650 | 15.3905 | 14.4203 |
| lncRNA33238 | "u" | chr08:55401272-55402461 | 1.5581 | 1.38915 |
| lncRNA03085 | "x" | chr01:80838432-80843744 | 2.71395 | 2.13914 |
| lncRNA21713 | "x" | chr05:2500500-2503202 | 0.899459 | 1.1738 |
| lncRNA19306 | "x" | chr04:57113107-57114937 | 3.48004 | 4.11389 |
| lncRNA03418 | "x" | chr01:84540079-84547356 | 1.89975 | 1.75586 |
| lncRNA41485 | "x" | chr11:5405456-5414005 | 7.34477 | 6.25317 |
| lncRNA47353 | "x" | chr12:29606520-29617960 | 6.12334 | 5.08353 |
| lncRNA43459 | "x" | chr11:8083497-8090336 | 36.6249 | 30.5174 |
| lncRNA10683 | "x" | chr02:41788683-41792962 | 8.57453 | 7.13336 |
| lncRNA32510 | "x" | chr08:7319082-7320550 | 0.715452 | 0.815478 |
| lncRNA22010 | "x" | chr05:6701172-6717388 | 9.58252 | 8.8304 |
| lncRNA22505 | "x" | chr05:40181137-40182704 | 2.38655 | 3.08881 |
| lncRNA00490 | "x" | chr00:20640675-20641779 | 7.17711 | 8.76883 |
| lncRNA30846 | "x" | chr08:9926690-9928295 | 5.56996 | 4.3548 |
| lncRNA11202 | "x" | chr02:47041609-47052491 | 12.8105 | 11.8426 |
| lncRNA27394 | "x" | chr07:6057149-6060363 | 17.6103 | 15.9997 |
| lncRNA30599 | "x" | chr08:1636604-1641994 | 0.800336 | 0.668944 |
| lncRNA38698 | "x" | chr10:61217212-61220722 | 9.50122 | 11.4527 |
| lncRNA07519 | "x" | chr02:32564785-32569827 | 1.44339 | 1.25794 |
| lncRNA19607 | "x" | chr04:61007604-61010109 | 7.8807 | 9.28112 |
| lncRNA31659 | "x" | chr08:57665993-57670136 | 1.48623 | 1.22426 |
| lncRNA11894 | "x" | chr03:9384617-9391549 | 6.86099 | 6.1593 |
| lncRNA37520 | "x" | chr10:2924760-2931466 | 0.899774 | 0.720168 |
| lncRNA07467 | "x" | chr02:31872390-31878126 | 13.4449 | 11.1576 |
| lncRNA38445 | "x" | chr10:58747523-58751931 | 2.25733 | 2.92259 |
| lncRNA11876 | "x" | chr03:9164129-9168788 | 0.984005 | 0.772279 |
| lncRNA20302 | "x" | chr05:5539413-5545737 | 1.66681 | 1.38638 |
| lncRNA48006 | "x" | chr12:62151781-62155714 | 2.07527 | 2.38664 |
| lncRNA31494 | "x" | chr08:55196941-55214508 | 3.36912 | 4.37733 |
| lncRNA40005 | "x" | chr10:47515077-47516650 | 19.7942 | 21.2602 |
| lncRNA19158 | "x" | chr04:54590861-54606991 | 21.5357 | 25.2426 |
| lncRNA39462 | "x" | chr10:5462082-5471231 | 0.618957 | 0.9221 |
| lncRNA03759 | "x" | chr01:87837114-87862936 | 1.20228 | 1.45917 |
| lncRNA10055 | "x" | chr02:34799314-34801862 | 19.838 | 18.5573 |
| lncRNA34222 | "x" | chr09:4650627-4658170 | 20.0118 | 23.115 |
| lncRNA06459 | "x" | chr01:86400109-86411982 | 3.22071 | 2.95665 |
| lncRNA17448 | "x" | chr04:58565791-58569731 | 29.3984 | 26.228 |
| lncRNA43216 | "x" | chr11:4040696-4041434 | 2.05496 | 1.67772 |
| lncRNA44633 | "x" | chr12:22129-27629 | 2.66422 | 2.02556 |
| lncRNA44756 | "x" | chr12:1001030-1005831 | 8.78531 | 7.43103 |
| lncRNA30077 | "x" | chr07:61075075-61080659 | 1.38688 | 1.13604 |
| lncRNA32885 | "x" | chr08:47399953-47404859 | 1.02383 | 0.781841 |
| lncRNA03203 | "x" | chr01:82041781-82043997 | 1.16995 | 1.57512 |
| lncRNA41623 | "u" | chr11:8172865-8192556 | 0.947115 | 1.35058 |
| lncRNA23517 | "u" | chr06:10136725-10137976 | 5.07597 | 5.49654 |
| lncRNA06454 | "x" | chr01:86350776-86353911 | 0.70631 | 0.590804 |
| lncRNA39579 | "u" | chr10:13620877-13625459 | 1.41339 | 1.24011 |
| lncRNA40768 | "x" | chr10:63078127-63084428 | 0.809135 | 0.656469 |
| lncRNA36552 | "x" | chr09:55885028-55889505 | 12.1411 | 10.4837 |
| lncRNA42738 | "x" | chr11:52536327-52540732 | 5.68109 | 6.2899 |
| lncRNA15823 | "x" | chr03:62238371-62239920 | 9.9686 | 11.7511 |
| lncRNA20369 | "x" | chr05:6222132-6227060 | 0.895121 | 1.15678 |
| lncRNA14749 | "x" | chr03:45669592-45670961 | 15.0027 | 17.1159 |
| lncRNA23924 | "x" | chr06:31765485-31778043 | 0.675758 | 0.924272 |
| lncRNA35754 | "x" | chr09:2160718-2165701 | 7.12745 | 8.43248 |
| lncRNA46186 | "x" | chr12:62694891-62698109 | 7.7673 | 6.98582 |
| lncRNA05565 | "x" | chr01:75966083-75971749 | 1.22939 | 1.07965 |
| lncRNA37415 | "x" | chr10:1772963-1779740 | 2.68521 | 2.35942 |
| lncRNA02659 | "x" | chr01:75702634-75713819 | 3.00005 | 3.55613 |
| lncRNA27644 | "x" | chr07:30224651-30226244 | 6.48197 | 5.83705 |
| lncRNA41209 | "x" | chr11:2210850-2215951 | 100.823 | 89.1653 |
| lncRNA24918 | "x" | chr06:43757023-43762616 | 6.30138 | 5.93063 |
| lncRNA12077 | "x" | chr03:16499875-16502714 | 1.80058 | 1.66134 |
| lncRNA25128 | "x" | chr06:45853962-45856385 | 4.13482 | 3.53198 |
| lncRNA44443 | "x" | chr11:51717239-51722947 | 3.36265 | 2.55327 |
| lncRNA39321 | "u" | chr10:2633252-2634611 | 2.43344 | 2.65889 |
| lncRNA04233 | "x" | chr01:3067812-3075245 | 15.2945 | 17.9121 |
| lncRNA30461 | "x" | chr08:2177-5917 | 6.1151 | 7.49387 |
| lncRNA34401 | "x" | chr09:12702259-12715748 | 2.6157 | 2.35605 |
| lncRNA08762 | "x" | chr02:46267019-46268130 | 24.8228 | 26.3981 |
| lncRNA18393 | "x" | chr04:6028485-6033726 | 27.4309 | 23.2028 |
| lncRNA43045 | "x" | chr11:2017934-2025276 | 11.0811 | 9.76392 |
| lncRNA22270 | "x" | chr05:19336285-19344234 | 7.688 | 10.3088 |
| lncRNA23955 | "x" | chr06:32373840-32381031 | 8.18799 | 9.85932 |
| lncRNA27205 | "x" | chr07:1914599-1915161 | 13.0863 | 10.255 |
| lncRNA38402 | "x" | chr10:57945111-57956476 | 1.6864 | 1.36626 |
| lncRNA32264 | "x" | chr08:1415280-1421980 | 8.6304 | 7.20339 |
| lncRNA00556 | "x" | chr00:5786752-5791417 | 2.44514 | 2.15971 |
| lncRNA15940 | "x" | chr03:63498194-63499734 | 1.74954 | 1.37929 |
| lncRNA06012 | "x" | chr01:81429272-81435009 | 13.2276 | 12.2976 |
| lncRNA28197 | "x" | chr07:58933883-58937799 | 9.71471 | 9.15164 |
| lncRNA08534 | "x" | chr02:44045071-44057225 | 0.778168 | 0.583883 |
| lncRNA11354 | "x" | chr02:48615201-48626949 | 22.3206 | 25.1366 |
| lncRNA22951 | "x" | chr05:62536960-62539494 | 0.692716 | 0.853521 |
| lncRNA16997 | "x" | chr04:50020619-50028376 | 3.95134 | 3.39715 |
| lncRNA33971 | "x" | chr09:1332196-1335760 | 6.26006 | 7.46854 |
| lncRNA43417 | "x" | chr11:6542920-6549944 | 0.922496 | 0.801164 |
| lncRNA05502 | "x" | chr01:75240885-75259743 | 2.47068 | 2.95342 |
| lncRNA21967 | "x" | chr05:6060861-6062102 | 3.14149 | 3.56343 |
| lncRNA26708 | "x" | chr06:42443319-42445004 | 7.72859 | 6.05939 |
[truncated: 264,832 more chars]
